# Temporal trends in inequalities of the burden of cardiovascular disease across 186 countries and territories

**DOI:** 10.1186/s12939-023-01988-2

**Published:** 2023-08-24

**Authors:** Penghong Deng, Yu Fu, Mingsheng Chen, Dong Wang, Lei Si

**Affiliations:** 1 School of Health Policy & Management, Nanjing Medical University, Nanjing, China; 2 Nanjing Drum Tower Hospital, Nanjing, China; 3 Center for Global Health, Nanjing Medical University, Nanjing, China; 4 School of Public Administration, Nanjing University of Finance and Economics, Qixia District, Nanjing, 210023 China; 5 School of Health Sciences, Western Sydney University, Campbelltown, NSW Australia; 6 Translational Health Research Institute, Western Sydney University, Penrith, NSW Australia

**Keywords:** Cardiovascular disease, Global burden of disease, Disability adjusted life years, Socioeconomic inequalities, Concentration index

## Abstract

**Background:**

Cardiovascular disease (CVD) is a leading cause of morbidity and mortality globally. The extent to which CVD affects the population’s health varies across countries. Moreover, quantitative estimates of the trend of inequalities in CVD burden remain unclear. The objective of our study was to assess the socioeconomic inequalities and temporal trends of CVD burden across 186 countries and territories from 2000 to 2019.

**Methods:**

We extracted data from the Global Burden of Disease, Injuries, and Risk Factors Study (GBD) 2019, and conducted a cross-national time-series analysis. Age-standardized disability-adjusted life-year (DALY) rates were used to measure the burden of CVDs, and gross national income (GNI) per capita was used to approximate the socioeconomic development. Concentration curves and concentration indexes (CIs) were generated to evaluate the cross-national socioeconomic inequality of CVD burden. A joinpoint regression analysis was used to quantify the changes in trends in socioeconomic inequality of CVD burden from 2000 to 2019.

**Results:**

The age-standardized DALY rates of CVDs decreased in 170 (91%) of 186 countries from 2000 to 2019. The concentration curves of the age-standardized DALY rates of CVDs were above the equality line from 2000 to 2019, indicating a disproportional distribution of CVD burden in low-income countries. The CIs declined from − 0.091 (95% CI: −0.128 to − 0.054) in 2000 to − 0.151 (95% CI: −0.190 to − 0.112) in 2019, indicating worsened pro-poor inequality distributions of CVD burden worldwide. A four-phase trend of changes in the CIs of age-standardized DALY rates for CVD was observed from 2000 to 2019, with an average annual percentage change (AAPC) of − 2.7% (95% CI: −3.0 to − 2.4). Decreasing trends in CIs were observed in all CVD subcategories but endocarditis, with AAPC ranging from − 6.6% (95% CI: −7.3 to − 5.9) for ischemic heart disease to − 0.2% (95% CI: −0.4 to − 0.1) for hypertensive heart disease.

**Conclusions:**

Globally, the burden of CVD has decreased in more than 90% of countries over the past two decades, accompanied by an increasing trend of cross-country inequalities. Moreover, the overall burden of CVD continues to fall primarily on low-income countries.

**Supplementary Information:**

The online version contains supplementary material available at 10.1186/s12939-023-01988-2.

## Background

Cardiovascular disease (CVD) is the most prevalent chronic disease, with 11 major subcategories such as rheumatic heart disease, ischemic heart disease, stroke, and hypertensive heart disease. It is a leading cause of death and disability worldwide [1, 2]. According to the World Health Organization (WHO), approximately 17.9 million people die from CVDs each year, accounting for one-third of all global deaths [2, 3]. More than three-quarters of CVD deaths occur in low- and middle-income countries (LMICs) and continue to rise over decades [2, 3]. The increasing prevalence of CVDs and non-communicable diseases (NCDs) can lead to severe and enduring economic impacts on individuals and their families, particularly in low-resource settings [4]. Numerous individuals with CVDs often face continuous out-of-pocket costs as they adhere to long-term healthcare, which can push their families into poverty and even result in catastrophic healthcare expenses. Despite the significant advances made in CVD prevention and control, CVD continues to impose a massive health and economic burden on individuals, healthcare systems, and society [2].

The burden of CVD is the result of a synergistic combination of unhealthy lifestyles and poor access to and performance of healthcare systems. In response to the rapidly increasing burden of CVD due to the epidemiological transition, the WHO launched the 25 × 25 Global Action Plan in 2013 [5]. The aim of the program is to reduce CVD-related premature mortality and the prevalence of raised blood pressure by 25% by 2025 worldwide [5]. In addition, WHO has put forth a set of cost-effective interventions referred to as “best buys”. These interventions encompass measures such as curtailing tobacco use, addressing the detrimental consumption of alcohol, countering unhealthy dietary habits, and promoting physical activity, which were advocated for the integration of these interventions into the fundamental primary health care package to propel forward the global health agenda [6]. Countries and territories have focused on the prevention and management of CVD risk factors through enhanced investment and international cooperation [6], with the commitment to deliver equitable access to primary health care and sustained economic safeguards. These ambitious goals are grounded in robust health systems, universal CVD prevention education, and medical technology support. However, most LMICs have insufficient health financing, fragile health systems, and poor health literacy, which limit their capacities to achieve the above-mentioned targets [7]. Significant disparities exist between LMICs in response to the health burden of CVD. Therefore, scrutiny of the current global burden of CVD and related inequalities is essential for the timely reorientation or enhancement of future strategies.

The Global Burden of Disease, Injuries, and Risk Factors Study (GBD) evaluated the burden of CVDs in 204 countries and territories worldwide [1], providing a valuable resource to measure changes in global cardiovascular health. A secondary study from GBD 2019 analyzed the global trends, and national and regional differences in CVD incidence and mortality between 1990 and 2019. However, this study failed to quantify to what extent the inequalities in CVD burden were distributed across countries [8]. Previous studies have documented widespread inequalities in CVD incidence, mortality, risk factors, care, and awareness by gender, race, and income, particularly in impoverished areas [9–11]. However, an assessment of cross-country inequalities and trends in the global burden of CVD and its subcategories has not yet been conducted.

In line with the Sustainable Development Goals (SDGs) to improve global health, measuring socioeconomic inequalities in CVD burden and the temporal trends in response to the ever-increasing population with CVD is crucial to ensure the equity and effectiveness of CVD prevention [12]. This has been particularly notable since the Coronavirus Disease 2019 (COVID-19) pandemic, as it has increased direct and indirect mortality of CVD and fueled social inequality, placing a challenge on already limited cardiovascular care resources [13]. In this study, we aimed to quantify the changes and socioeconomic inequalities in the burden of CVD and its subcategories, and the temporal trends in inequalities in 186 countries and territories from 2000 to 2019.

## Methods

### Study characteristics

This study is characterized as a cross-national, observational, and cross-sectional investigation. The study collected data on the socioeconomic development and CVD burden from 186 countries/territories spanning the years 2000 to 2019 with an aim to assess inequalities and temporal trends in CVDs and its 11 subgroups.

### Data sources

GBD 2019 provides comprehensive, multi-national, and multi-institutional global collaborative epidemiological research for estimating the burden of 369 diseases and injuries by sex and age group for 204 countries and territories from 1990 to 2019 [1, 14]. We used the data over 2000–2019 to estimate the changes in disease burden of CVD and its 11 subtypes: rheumatic heart disease, ischemic heart disease, stroke, hypertensive heart disease, non-rheumatic valvular heart disease, cardiomyopathy and myocarditis, atrial fibrillation and flutter, aortic aneurysm, peripheral artery disease, endocarditis, and other cardiovascular and circulatory diseases. Disability-adjusted life years (DALYs) were used to quantify the burden of CVD by measuring the healthy loss from both fatal and nonfatal outcomes, including the years of life lost (YLLs) due to premature mortality and years lived with disability (YLDs) [15, 16]. Considering the rapid population growth and changing age composition, we used age-standardized DALY rates in the analyses [17]. Detailed methods for calculating age-standardized DALY rates are presented in GBD publications [18].

### Socioeconomic development across nations

Gross National Income (GNI) per capita was used as a proxy for national socioeconomic development. GNI has been recognized as a good indicator to measure the country’s economic strengths, needs, and the general living standard of its average citizens [19]. We collected GNI per capita data for each country from the website of the World Bank [20]. GNI is the sum of value added by all resident producers plus any product taxes not included in the output valuation plus net receipts of primary income from abroad, calculated in national currency and converted to current US dollars according to the World Bank Atlas method, and divided by the midyear population [21]. To account for non-linearity due to marginal utility, we took a logarithmic transformation of the GNI per capita [22].

### Measures of health inequality

We used the concentration curve and concentration index (CI) [23] to quantify the health inequality of CVD and its 11 subcategories among countries from 2000 to 2019. The concentration curve mapped the distribution of inequality in the cumulative fraction of the age-standardized DALY rates against the cumulative country proportion ranked by national socioeconomic development (i.e., GNI per capita). If the curve lies above the line of equality (45-degree line), the health outcome variable (age-standardized DALY rates) is more prevalent among low-income countries, and vice versa [23]. The CI is derived from the concentration curve, which quantifies the relative socioeconomic inequality in health and is equal to twice the area between the concentration curve and line of equality. The CI ranges from − 1 to 1, where a negative value indicates that age-standardized DALY rate is concentrated more in low-income countries, whereas a positive value indicates a concentration of disease burden in high-income countries [23].

### Statistical analysis

For this study, we did an international, time-series secondary analysis of GBD 2019. To characterize the cross-country burden of CVD and its 11 subcategories, a descriptive analysis was done. We calculated the change rate of age-standardized DALY rates from 2000 to 2019, and divided countries into four groups: disease burden increased by 15% or over, increased by less than 15%, decreased by 15% or below, and decreased by more than 15%. Linear regression analysis was used to investigate the relationship between GNI per capita and age-standardized DALY rates of CVD and its 11 subcategories. A joinpoint regression analysis was used to access significant trends in CIs from 2000 to 2019 by estimating annual percentage change (APC) and average annual percentage change (AAPC). The APC for the segments and the AAPC is a summary measure of the trend, which expresses the weighted average of APC for the overall period [17, 22]. The Monte Carlo permutation method [24] was used to assess the significant changes in the CIs over time and estimate the 95% confidence interval (CI) and *p*-value; the statistical significance level is corrected by the Bonferroni method for greater consistency in the *p*-value. Values are considered statistically significant if APC and AAPC are different from zero at an alpha of 0.05.

All statistical analyses were conducted using SPSS v25.0 (SPSS Inc., Chicago, IL, USA) and Stata v13.0 (Stata Corp., College Station, TX, USA). All joinpoint analyses were performed using Joinpoint Statistical Software (Joinpoint Regression Program, Version 4.8.0.1-April 2020, National Cancer Institute, Bethesda, MD, USA; Statistical Methodology and Applications Branch, Surveillance Research Program, National Cancer Institute).

## Results

### Global burden of CVD with age-standardized DALY rates

**Fig. 1:**
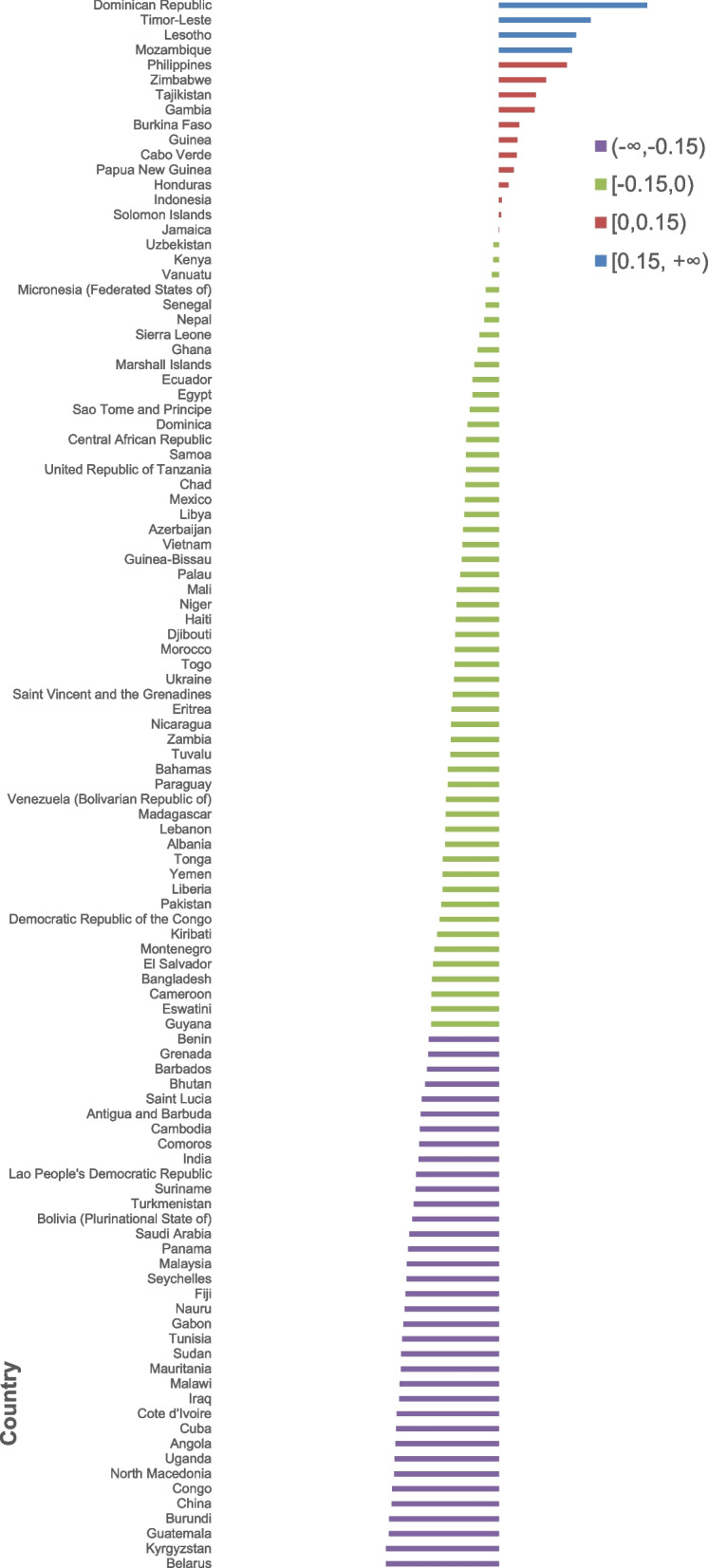
The change rate of age-standardized DALY rates for CVD from 2000 to 2019

The change rate of age-standardized DALY rates for CVD from 2000 to 2019 across 186 countries is presented in Fig. 1. Globally, the overall age-standardized DALY rates for CVD decreased by 23.89%: from 6390.56 to 2000 to 4863.64 in 2019 per 100,000 people. The age-standardized DALY rates of CVD decreased in 170 (91%) of 186 countries from 2000 to 2019, of which 117 (69%) countries decreased by more than 15%.

Among all subcategories of CVD, more than half of countries had a decrease in age-standardized DALY rates from 2000 to 2019 apart from atrial fibrillation and flutter and peripheral artery disease, ranging from 57% for endocarditis to 98% for rheumatic heart disease (Additional file 1: Figs. S1–11). In addition, for rheumatic heart disease, ischemic heart disease, stroke, and cardiomyopathy and myocarditis, the age-standardized DALY rates decreased (i.e., more than a 15% reduction) in about 94%, 60%, 72%, and 52% of countries from 2000 to 2019, respectively (Additional file 1: Figs. S1–3, Fig. S6).

### Socioeconomic inequality in the burden of CVD

As illustrated by the concentration curves, during the period 2000–2019, all curves lay above the equality line, indicating that the geographic distribution of age-standardized DALY rates due to CVD was concentrated in poor countries/territories. Moreover, the distances above the equality line were increasingly far between 2000 and 2019, with the CI decreasing from − 0.091 (95% CI: −0.128 to − 0.054) in 2000 to − 0.151 (95% CI: −0.190 to − 0.112) in 2019 (Figs. 2, 3 and 4, Additional file 1: Figs. S12–29).

**Fig. 2:**
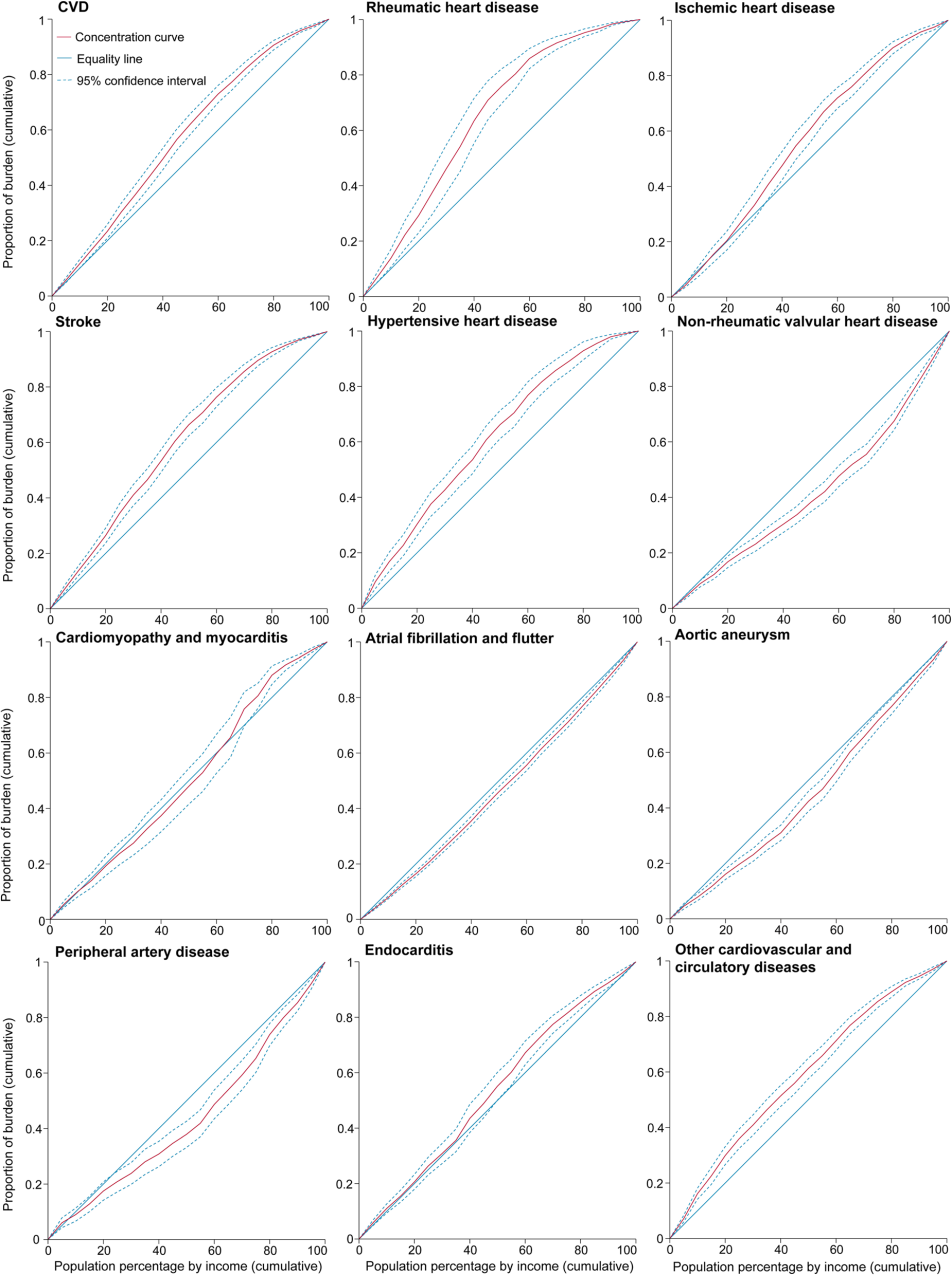
Concentration curves of age-standardized DALY rates for CVD and its subcategories in 2000

By subcategories, the concentration curves of age-standardized DALY rates of non-rheumatic valvular heart disease, atrial fibrillation and flutter, aortic aneurysm, and peripheral artery disease lay below the equality line from 2000 to 2019, suggesting that the burdens of these subcategories were more endemic among rich countries/territories. The distances below the equality line grew increasingly close, with the CI decreasing from 0.180 (95% CI: 0.135 to 0.224) in 2000 to 0.159 (95% CI: 0.116 to 0.201) in 2019 for non-rheumatic valvular heart disease, from 0.081 (95% CI: 0.059 to 0.104) in 2000 to 0.065 (95% CI: 0.043 to 0.086) in 2019 for atrial fibrillation and flutter, from 0.147 (95% CI: 0.105 to 0.190) in 2000 to 0.092 (95% CI: 0.051 to 0.134) in 2019 for aortic aneurysm, and from 0.170 (95% CI: 0.113 to 0.227) in 2000 to 0.128 (95% CI: 0.066 to 0.189) in 2019 for peripheral artery disease (Figs. 2, 3 and 4, Additional file 1: Figs. S102–119, Figs. S138–191).

**Fig. 3:**
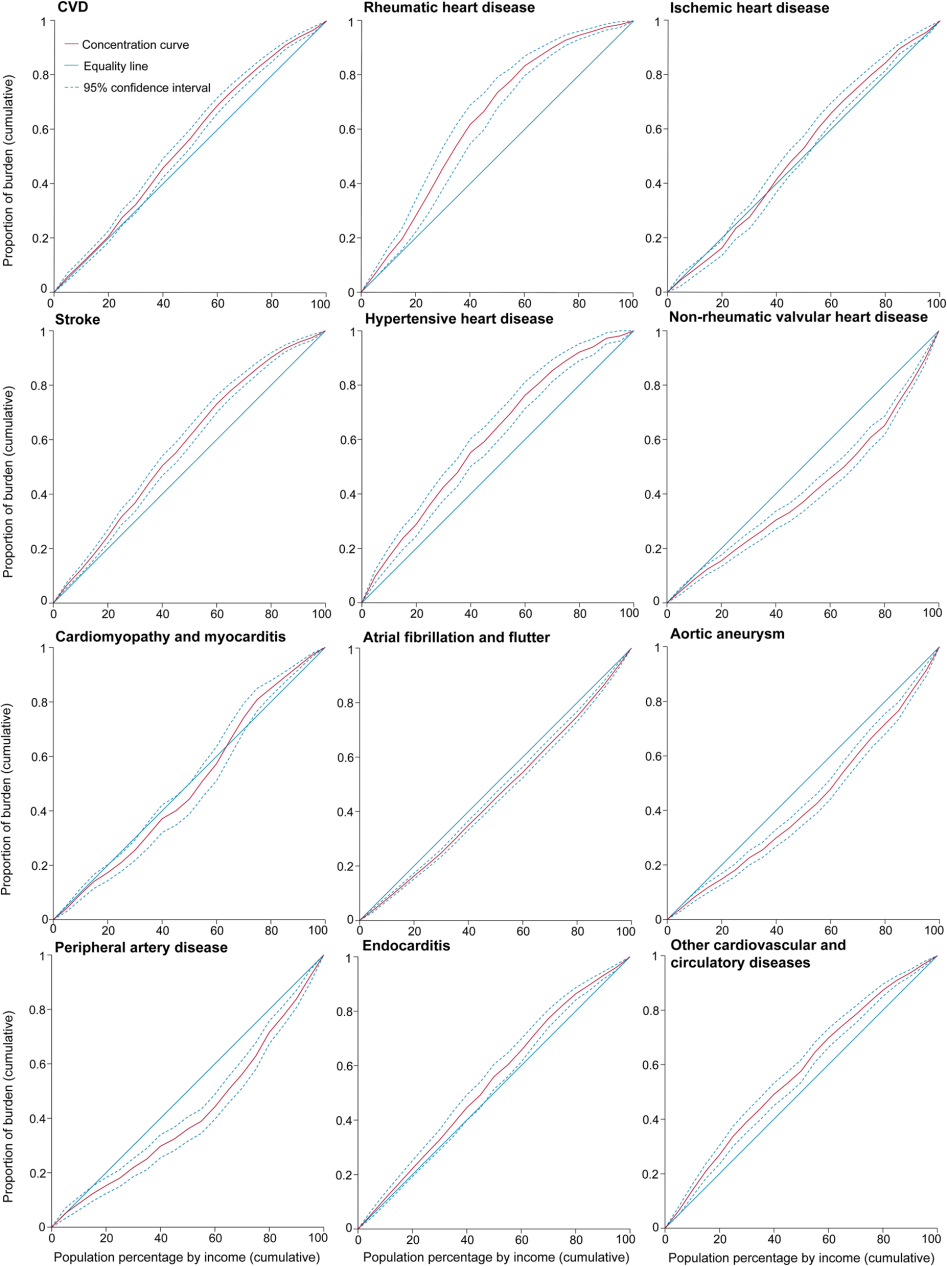
Concentration curves of age-standardized DALY rates for CVD and its subcategories in 2019

For rheumatic heart disease, ischemic heart disease, stroke, hypertensive heart disease, and other cardiovascular and circulatory diseases, the concentration curves were above the equality line, that is, poor countries/territories were sharing the major burden. However, the distances above the equality line became increasingly large, with the CI decreasing from − 0.275 (95% CI: −0.355 to − 0.196) in 2000 to − 0.301 (95% CI: −0.391 to − 0.210) in 2019 for rheumatic heart disease, from − 0.035 (95% CI: −0.085 to 0.015) in 2000 to − 0.119 (95% CI: −0.172 to − 0.066) in 2019 for ischemic heart disease, from − 0.154 (95% CI: −0.191 to − 0.116) in 2000 to − 0.206 (95% CI: −0.247 to − 0.166) in 2019 for stroke, from − 0.218 (95% CI: −0.273 to − 0.164) in 2000 to − 0.229 (95% CI: −0.281 to − 0.177) in 2019 for hypertensive heart disease, and from − 0.133 (95% CI: −0.180 to − 0.086) in 2000 to − 0.173 (95% CI: −0.214 to − 0.131) in 2019 for other cardiovascular and circulatory diseases. In addition, the concentration curves of age-standardized DALY rates for endocarditis lay above the equality line, and the distances above the equality line grew increasingly close, with the CI increasing from − 0.077 (95% CI: −0.131 to − 0.022) in 2000 to − 0.067 (95% CI: −0.124 to − 0.009) in 2019 (Figs. 2, 3 and 4, Additional file 1: Figs. S30–101, Figs. S192–227).

**Fig. 4:**
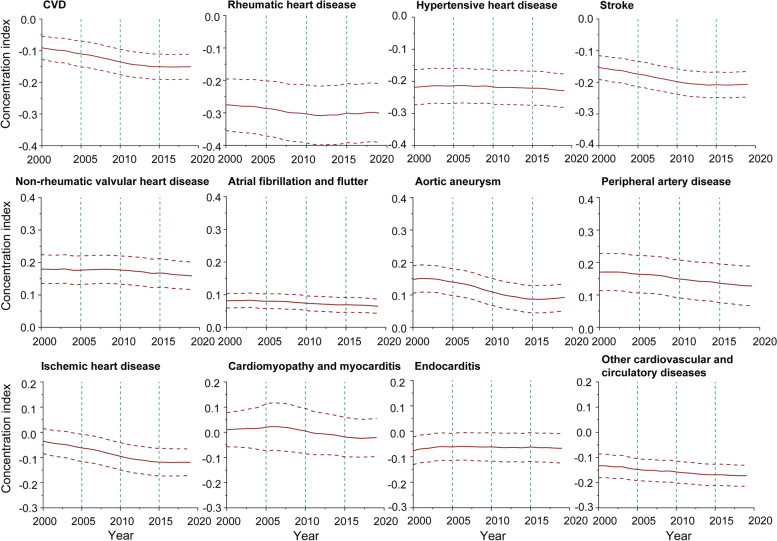
Concentration index of age-standardized DALY rates for CVD and its subcategories from 2000 to 2019

### Trends in socioeconomic inequality of CVD burden

Globally, the CIs of global age-standardized DALY rates of CVD fell, with an AAPC of − 2.7% (95% CI: −3.0 to − 2.4) from 2000 to 2019. The negative CIs demonstrated that the pro-poor inequality distribution of the overall burden of CVD was increasing over time. The decline in the CIs of age-standardized DALY rates of CVD changed over time in four phases: accelerating from 2000 to 2006 (− 3.8%), further accelerating from 2006 to 2011 (− 4.4%), and decelerating from 2011 to 2014 (− 2.1%) and 2014–2019 (− 0.1%), where the first three periods were with significant reductions (Table 1).

**Table 1:** Trends in concentration index of age-standardized DALY rates for CVD from 2000 to 2019

Segment	Period	APC/AAPC (%)	95% CI		*p-*value
Trend 1	2000–2006	−3.8^a^	−4.1 to − 3.5		< 0.001
Trend 2	2006–2011	−4.4^a^	−5.0 to − 3.8		< 0.001
Trend 3	2011–2014	−2.1^a^	−4.0 to − 0.3		0.029
Trend 4	2014–2019	−0.1	−0.5 to 0.3		0.465
Total	2000–2019	−2.7^a^	−3.0 to − 2.4		< 0.001

*APC *Annual percentage change, *AAPC *Average annual percentage change, *CI *Confidence interval

^a^Significant at alpha = 0.05

Between 2000 and 2019, the CIs of age-standardized DALY rates decreased significantly for all subcategories except endocarditis, irrespective of whether the CIs were positive or negative, with the AAPC ranging from − 6.6% (95% CI: −7.3 to − 5.9) for ischemic heart disease to − 0.2% (95% CI: −0.4 to − 0.1) for hypertensive heart disease (Table 2). Given that the CIs for age-standardized DALY rates of non-rheumatic valvular disease, atrial fibrillation and flutter, aortic aneurysm, and peripheral artery disease were positive, the pro-rich inequality distributions in the burden of these dieaseswere progressively narrowing worldwide. Also, the gap in the burden of endocarditis was declining across countries with different levels of socioeconomic development because of the negative CIs of the age-standardized DALY rates, with an increase in AAPC of 0.7% (95% CI: 0.3 to 1.0). However, given that the CIs were negative, together with the decreased AAPC, the pro-poor inequality in the global burden of rheumatic heart disease, ischemic heart disease, stroke, hypertensive heart disease, and other cardiovascular and circulatory diseases was worsening over time (Additional file 1: Tables S1–11).

**Table 2:** AAPC of concentration index of age-standardized DALY rates for subcategories from 2000 to 2019

Subcategories	Period	AAPC (%)	95% CI	*p*-value
Rheumatic heart disease	2000–2019	−0.4^a^	−0.6 to − 0.2	< 0.001
Ischemic heart disease	2000–2019	−6.6^a^	−7.3 to − 5.9	< 0.001
Stroke	2000–2019	−1.6^a^	−1.8 to − 1.4	< 0.001
Hypertensive heart disease	2000–2019	−0.2^a^	−0.4 to − 0.1	< 0.001
Non-rheumatic valvular heart disease	2000–2019	−0.6^a^	−0.7 to − 0.5	< 0.001
Cardiomyopathy and myocarditis^b^	2000–2010	−6.3^a^	−9.0 to − 3.4	< 0.001
	2011–2019	−27.7^a^	−35.1 to − 20.6	< 0.001
Atrial fibrillation and flutter	2000–2019	−1.1^a^	−1.6 to − 0.7	< 0.001
Aortic aneurysm	2000–2019	−2.5^a^	−2.9 to − 2.0	< 0.001
Peripheral artery disease	2000–2019	−1.5^a^	−1.8 to − 1.3	< 0.001
Endocarditis	2000–2019	0.7^a^	0.3 to 1.0	< 0.001
Other cardiovascular and circulatory diseases	2000–2019	−1.4^a^	−1.6 to − 1.2	< 0.001

*APC *Annual percentage change, *AAPC *Average annual percentage change, *CI *Confidence interval

^a^Significant at alpha = 0.05

^b^The CIs of cardiomyopathy and myocarditis varied in positive and negative values across years, and the overall trend in the CIs was divided into two segments in 2000–2019 due to the limitation of the consistency of positive data in the joinpoint regression

## Discussion

This study reports the socioeconomic inequalities, trends, and the changes in age-standardized DALY rates due to CVD from 2000 to 2019. Our results suggested that the overall burden of CVD has decreased. However, countries with lower socioeconomic development continue to share a higher CVD burden, and between-country pro-poor inequalities in the CVD burden showed an increasing trend from 2000 to 2019. This trend varied, with an accelerating trend over the 2000–2014 interval followed by a slowdown in the rate of increase over 2014–2019. A decreasing trend of inequality was observed in 5 of the 11 subcategories—non-rheumatic valvular heart disease, atrial fibrillation and flutter, aortic aneurysm, peripheral artery disease, and endocarditis—from 2000 to 2019.

The results indicated that there was a worldwide downward trend in the overall burden of CVD from 2000 to 2019, and, at the national level, the burden of CVD decreased in more than 90% of countries in this period. There are few studies examining the long-term trend of global CVD burden using age-standardized DALY rates, which are adjusted for population size and age structure and are considered to be a more proper metric for comparison across countries than crude DALY rates or YLDs [25], and some researchers were limited to investigating CVD burden trends in a single geographic location [14]. For example, a worldwide study by Roth et al. indicated that the YLDs of CVD showed a decreasing trend from 1990 to 2019 [2]. One study reported a decreasing trend in YLDs of CVD in the United States from 1990 to 2017, also finding that among CVD subtypes, rheumatic heart disease had the sharpest downward slope [26], which was similar to our finding that the burden of rheumatic heart disease decreased by more than 15% in about 94% of countries. The reduced global burden of CVD was attributed to a combination of efforts, including global adoption of healthy lifestyles, controlling related risk factors, improving public health awareness and use of emergency medical services, and rapid progress in access to prevention, care, and treatment [2, 14, 15, 27–30].

Our study found that countries with higher levels of socioeconomic development were more likely to bear low CVD burden. In previous studies, most wealthier regions had relatively lower incidence and mortality of CVD than poorer ones, and CVD burden was inversely related to the socioeconomic development levels of the countries [2, 3, 31–33]. Wealth or income has been identified as a core social determinant in population health, and its distributional inequalities and imbalances may exert a substantially adverse impact on health financing, access to healthcare, health insurance coverage, access to education, and health outcomes [34]. Developed countries are more capable of responding to and managing CVDs by depending on well-established and accessible healthcare systems, which contributes significantly to improve the diagnosis and treatment rates of CVD. However, in many countries with lower levels of socioeconomic development, poor access to primary health care and an absence of professional guidelines, medicines, and caregivers lead to low rates of CVD diagnosis and treatment. CVDs can interfere with other conditions in multiple ways, potentially leading to a worse outcome. In addition, poverty, overcrowding, unhealthy diets, contaminated alcohol, and large populations persist in most low-income countries and are driving up the prevalence of CVDs [35], which could further explain the geographic disparities in CVD burden.

We also found significant disparities in the distribution of CVD burden across CVD subgroups from 2000 to 2019. Of note, the burdens of non-rheumatic heart disease, atrial fibrillation and flutter, aortic aneurysm, and peripheral artery disease were concentrated in countries with higher levels of socioeconomic development. As previously reported, the incidence and prevalence of atrial fibrillation and flutter, aortic aneurysm, and peripheral arterial disease are greater in developed countries than in developing ones [36–38]. One study indicated that a higher level of European ancestry was associated with increased susceptibility to atrial fibrillation, which may be one plausible explanation [39]. However, the high degree of aging, increased life expectancy, and comprehensive disease surveillance information in developed countries may also be responsible for these geographic variations in disease burden [36].

Our discovery of significantly increasing pro-poor inequalities in the overall burden of CVD among countries over time is concerning, as it suggests an inadequate and uneven control of risk factors geographically. Unfortunately, global cardiovascular health is experiencing adverse trends. The concentration curves of the CVD burden increasingly deviating from the diagonal line over time also corroborated such trends, especially in 2000–2014 with a pronounced unequal tendency, which was associated with an early response of robust health systems in developed countries to a sudden and dramatic increase in CVDs incidence. The absence of NCDs from the original Millennium Development Goals (MDGs) has resulted in a dearth of significant policy support and concern for CVDs since 2000 [12]. Many low-income countries still struggled to tackle the epidemic of infectious diseases, and CVDs received few health resources for more than a decade [12], which may partially explain the significantly accelerated trend of increasing CVD inequality from 2000 to 2014. In 2014, the United Nations (UN) launched the SDGs and placed the control of NCDs on the agenda [12]. Nations and initiatives committed to scaling up resources and funding for CVDs and other NCDs to address the rapidly rising prevalence and burden of the latter. Thus, cross-country inequalities in the burden of CVDs have eased in recent years, but the landscape remains challenging.

It is encouraging that, in the current study, inequalities in the global burden of non-rheumatic valvular disease, atrial fibrillation and flutter, aortic aneurysm, peripheral artery disease, and endocarditis have progressively decreased over the past 20 years. Although the exact reasons for these positive trends are uncertain, of importance is the recognition of the diseases as a major public health problem in countries worldwide, and considerable resources have been invested in the surveillance and control of the risk factors, such as smoking cessation and hypertension management. In developed countries and regions, effective low-cost screening, advanced treatment technology, and better health awareness reduced the prevalence and mortality of these CVDs [40–42], as evidenced by previous studies, that is, countries with higher levels of social development have better quality of CVDs care [41, 43]. In particular, previous studies indicated that a major increase in the global incidence of endocarditis occurred in developed countries, which was attributed to increases in prosthetic valves, intravenous drug use, and cardiac devices, potentially narrowing the gap in the burden of endocarditis for developing countries [44]. However, changes in equality distribution for some CVDs require special attention. For instance, pro-poor inequalities in rheumatic heart disease, ischemic heart disease, stroke, hypertensive heart disease, and other cardiovascular and circulatory diseases have escalated over the past two decades. This is particularly pronounced in the case of ischemic heart disease. These conditions collectively stand as the foremost causes of death, demanding immediate and resolute action to mitigate the unfavorable trends. Yet, addressing these challenges poses a formidable task for LMICs characterized by vast territories and widespread health disparities. The complexities lie in the substantial efforts required to establish and fortify control programs, fundamental prevention and educational initiatives, surgical interventions, and the provision of sustained treatment for advanced-stage diseases. Furthermore, the LMICs have undergone rapid economic transitions, industrialization, urbanization, and globalization over the last two decades, inducing drastic shifts in lifestyles and diets. These changes have significantly contributed to the surge in ischemic heart disease, largely driven by metabolic risk factors [45]. 

Our analysis of the temporal trends in inequalities of CVD burden and its subcategories is beneficial for illustrating global patterns of CVD inequality, which may contribute to policy and strategy development worldwide. Further expansion of health assistance and universal health coverage for lower socioeconomic development countries, such as much of the sub-Saharan and Pacific Island nations, is warranted as a priority to curb the progress of CVD inequities. The need for early detection and treatment cannot be overemphasized, especially for regions with restricted health resources. Given that most CVDs can be prevented by targeting behavioral risk factors, future research on the control strategies of CVD is needed to continuously focus on the intervention and evaluation of these factors, which must be improved. One open-label, cluster-randomized trial (Salt Substitute and Stroke Study, SSaSS) demonstrated that salt substitution could lower the risk of cardiovascular events [46], and a modeling study in China projected that 461,000 cardiovascular deaths and 743,000 nonfatal cardiovascular events could be avoided each year by implementing salt substitution [47]. Salt substitution, which is the only salt-reduction intervention with grade-one evidence [48] and with cost-effectiveness, can be considered by all countries who are planning or implementing early prevention of CVDs. For CVDs that exhibit well-managed inequality, such as non-rheumatic valvular disease, atrial fibrillation and flutter, aortic aneurysm, peripheral arterial disease, and endocarditis, the responsive strategies and advanced approaches to surveillance, screening, and treatment in developed nations have effectively curtailed disparities [41, 42]. Drawing insights from the successful endeavors of these countries and comprehending the social determinants that underlie variations across nations are imperative for enhancing regional performance and extending support to nations that are trailing behind. Furthermore, the timely revelations of this study necessitate a renewed commitment to reversing the glaring trends of inequality in rheumatic heart disease, ischemic heart disease, stroke, and hypertensive heart disease. It urges the development of interventions that are both time-efficient and cost-effective, while being accessible to LMICs. In these nations, a pivotal starting point involves targeting urgent risk factors, such as unhealthy lifestyles, cardiometabolic variables, air pollution, and healthcare inequities [2]. This approach ensures the optimal allocation of limited resources and funding. Thus, a pressing mandate exists for a well-coordinated, cross-sectoral, and multi-tiered collaboration involving policy makers, healthcare providers, and researchers. Such collaboration is essential for the effective implementation of policies, prevention measures, and management strategies, facilitating the expansion of interventions and research on a broader scale.

### Strength and limitations

One strength of the current study is the comprehensive population-based assessment and comparison of trends in socioeconomic inequalities of CVD burden worldwide based on nations’ income levels, which adds to the evidence base for the development and implementation of strategies on the control and management of CVD and its subcategories. However, there are several limitations to be considered. First, as with other GBD studies, the accuracy of estimates in our study depends on the quality and quantity of data sources, and these are associated with detection techniques, incomplete case-reports, and data collection and encoding methods used in different countries. Second, the absence of epidemiological surveys in certain regions may result in a hidden incidence, particularly in low-income countries, which means that inequality of CVD burden is potentially underestimated. Third, it’s important to acknowledge that the joinpoint regression method used for the secondary data analysis did not take into account the uncertainty measure in the GBD raw data. This omission might lead to an underestimation of the uncertainty associated with the trends in AAPC. Fourth, caution is warranted when interpreting the results, as correlated evidence from other studies does not necessarily indicate causality. Furthermore, the policy implications presented in the study may not be universally applicable to countries with distinct socio-economic and healthcare contexts. Fifth, this study is a secondary analysis grounded in GBD epidemiologic data, inherently lacks the control of potential confounders, including the influence of epidemiological transition across countries. Finally, our study is cross-national, which may introduce bias due to a lack of understanding of the disparities that exist between regions within countries.

## Conclusion

Globally, the burden of CVD has decreased in 170 (91%) of 186 countries over the past 20 years. Low-income countries continue to share the major burden of CVD. Socioeconomic inequality in global burden of CVD has worsened over this period, and inequalities vary across the different subcategories. Effective and cost-effective interventions for preventing CVD are crucial in curbing the progress of CVD inequalities.

### Supplementary Information

**Additional file 1: Fig S1.** The change rate of age-standardized DALY rates for rheumatic heart disease from 2000 to 2019. **Fig S2.** The change rate of age-standardized DALY rates for ischemic heart disease from 2000 to 2019. **Fig S3.** The change rate of age-standardized DALY rates for stroke from 2000 to 2019. **Fig S4.** The change rate of age-standardized DALY rates for hypertensive heart disease from 2000 to 2019. **Fig S5.** The change rate of age-standardized DALY rates for non-rheumatic valvular heart disease from 2000 to 2019. **Fig S6.** The change rate of age-standardized DALY rates for cardiomyopathy and myocarditis from 2000 to 2019. **Fig S7.** The change rate of age-standardized DALY rates for atrial fibrillation and flutter from 2000 to 2019. **Fig S8.** The change rate of age-standardized DALY rates for aortic aneurysm from 2000 to 2019. **Fig S9.** The change rate of age-standardized DALY rates for peripheral artery disease from 2000 to 2019. **Fig S10.** The change rate of age-standardized DALY rates for endocarditis from 2000 to 2019. **Fig S11.** The change rate of age-standardized DALY rates for other cardiovascular and circulatory diseases from 2000 to 2019. **Figs S12-29.** Concentration curves of age-standardized DALY rates for CVD from 2001 to 2018. **Figs S30-47.** Concentration curves of age-standardized DALY rates for rheumatic heart disease from 2001 to 2018. **Figs S48-65.** Concentration curves of age-standardized DALY rates for ischemic heart disease from 2001 to 2018. **Figs S66-83.** Concentration curves of age-standardized DALY rates for stroke from 2001 to 2018. **Figs S84-101.** Concentration curves of age-standardized DALY rates for hypertensive heart disease from 2001 to 2018. **Figs S102-119.** Concentration curves of age-standardized DALY rates for non-rheumatic valvular heart disease from 2001 to 2018. **Figs S120-137.** Concentration curves of age-standardized DALY rates for cardiomyopathy and myocarditis from 2001 to 2018. **Figs S138-155.** Concentration curves of age-standardized DALY rates for atrial fibrillation and flutter from 2001 to 2018. **Figs S156-173.** Concentration curves of age-standardized DALY rates for aortic aneurysm from 2001 to 2018. **Figs S174-191.** Concentration curves of age-standardized DALY rates for peripheral artery disease from 2001 to 2018. **Figs S192-209.** Concentration curves of age-standardized DALY rates for endocarditis from 2001 to 2018. **Figs S210-227.** Concentration curves of age-standardized DALY rates for other cardiovascular and circulatory diseases from 2001 to 2018. **Table S1.** Trends in concentration index of age-standardized DALY rates for rheumatic heart disease from 2000-2019. **Table S2.** Trends in concentration index of age-standardized DALY rates for ischemic heart disease from 2000-2019. **Table S3.** Trends in concentration index of age-standardized DALY rates for stroke from 2000-2019. **Table S4.** Trends in concentration index of age-standardized DALY rates for hypertensive heart disease from 2000-2019. **Table S5.** Trends in concentration index of age-standardized DALY rates for non-rheumatic valvular heart disease from 2000-2019. **Table S6.** Trends in concentration index of age-standardized DALY rates for cardiomyopathy and myocarditis from 2000-2019. **Table S7.** Trends in concentration index of age-standardized DALY rates for atrial fibrillation and flutter from 2000-2019. **Table S8.** Trends in concentration index of age-standardized DALY rates for aortic aneurysm from 2000-2019. **Table S9.** Trends in concentration index of age-standardized DALY rates for peripheral artery disease from 2000-2019. **Table S10.** Trends in concentration index of age-standardized DALY rates for endocarditis from 2000-2019. **Table S11.** Trends in concentration index of age-standardized DALY rates for other cardiovascular and circulatory diseases from 2000-2019. 

## Data Availability

Data are publicly available. The datasets generated and analysed during the current study are available in the Global Health Data Exchange GBD 2019 data resources (https://ghdx.healthdata.org/gbd-2019) and the databank of the World Bank (https://databank.worldbank.org/home).

## Abbreviations

CVD: Cardiovascular disease
GBD: The Global Burden of Disease, Injuries, and Risk Factors Study
DALY: Disability-adjusted life-year
GNI: Gross national income
CI: Concentration index
APC: Annual percentage change
AAPC: Average annual percentage change
WHO: World Health Organization
LMICs: Low- and middle-income countries
SDGs: Sustainable Development Goals
COVID-19: Coronavirus Disease 2019
YLLs: Years of life lost
YLDs: Years lived with disability
NCDs: non-communicable diseases
MDGs: Millennium Development Goals
UN: the United Nations
SSaSS: Salt Substitute and Stroke Study
