# Supplementary material for: Temporal trends in inequalities of the burden of cardiovascular disease across 186 countries and territories
Source: Int J Equity Health. 2023 Aug 24;22:164. doi: 10.1186/s12939-023-01988-2 (PMC10463977; doi:10.1186/s12939-023-01988-2)
Supplement: Supplementary file 1 — Additional file 1: Fig S1. The change rate of age-standardized DALY rates for rheumatic heart disease from 2000 to 2019. Fig S2. The change rate of age-standardized DALY rates for ischemic heart disease from 2000 to 2019. Fig S3. The change rate of age-standardized DALY rates for stroke from 2000 to 2019. Fig S4. The change rate of age-standardized DALY rates for hypertensive heart disease from 2000 to 2019. Fig S5. The change rate of age-standardized DALY rates for non-rheumatic valvular heart disease from 2000 to 2019. Fig S6. The change rate of age-standardized DALY rates for cardiomyopathy and myocarditis from 2000 to 2019. Fig S7. The change rate of age-standardized DALY rates for atrial fibrillation and flutter from 2000 to 2019. Fig S8. The change rate of age-standardized DALY rates for aortic aneurysm from 2000 to 2019. Fig S9. The change rate of age-standardized DALY rates for peripheral artery disease from 2000 to 2019. Fig S10. The change rate of age-standardized DALY rates for endocarditis from 2000 to 2019. Fig S11. The change rate of age-standardized DALY rates for other cardiovascular and circulatory diseases from 2000 to 2019. Figs S12-29. Concentration curves of age-standardized DALY rates for CVD from 2001 to 2018. Figs S30-47. Concentration curves of age-standardized DALY rates for rheumatic heart disease from 2001 to 2018. Figs S48-65. Concentration curves of age-standardized DALY rates for ischemic heart disease from 2001 to 2018. Figs S66-83. Concentration curves of age-standardized DALY rates for stroke from 2001 to 2018. Figs S84-101. Concentration curves of age-standardized DALY rates for hypertensive heart disease from 2001 to 2018. Figs S102-119. Concentration curves of age-standardized DALY rates for non-rheumatic valvular heart disease from 2001 to 2018. Figs S120-137. Concentration curves of age-standardized DALY rates for cardiomyopathy and myocarditis from 2001 to 2018. Figs S138-155. Concentration curves of age-standardized DALY rate [file 12939_2023_1988_MOESM1_ESM.pdf]

Additional file 1

Fig. S1 The change rate of age-standardized DALY rates for rheumatic heart disease from 2000 to 2019

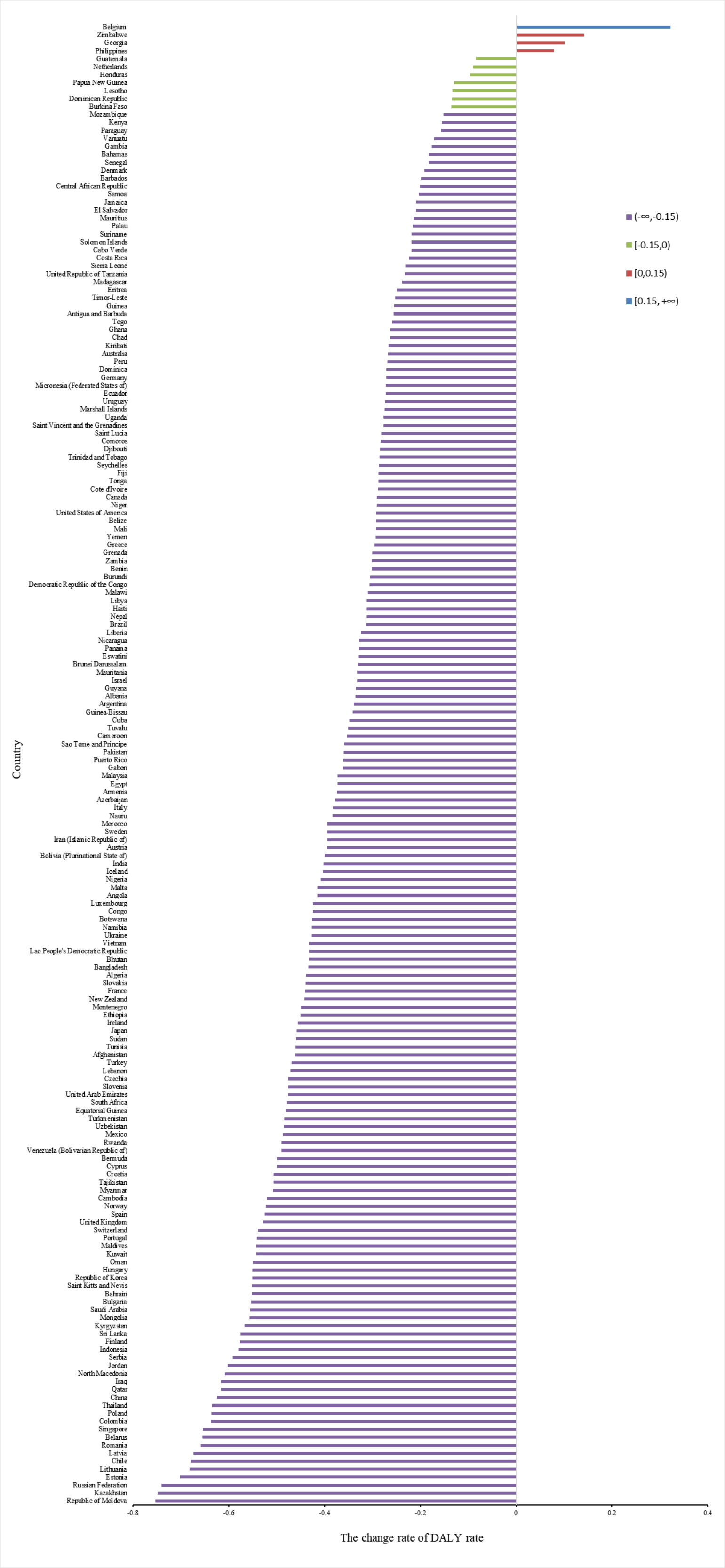

Fig. S2 The change rate of age-standardized DALY rates for ischemic heart disease from 2000 to 2019

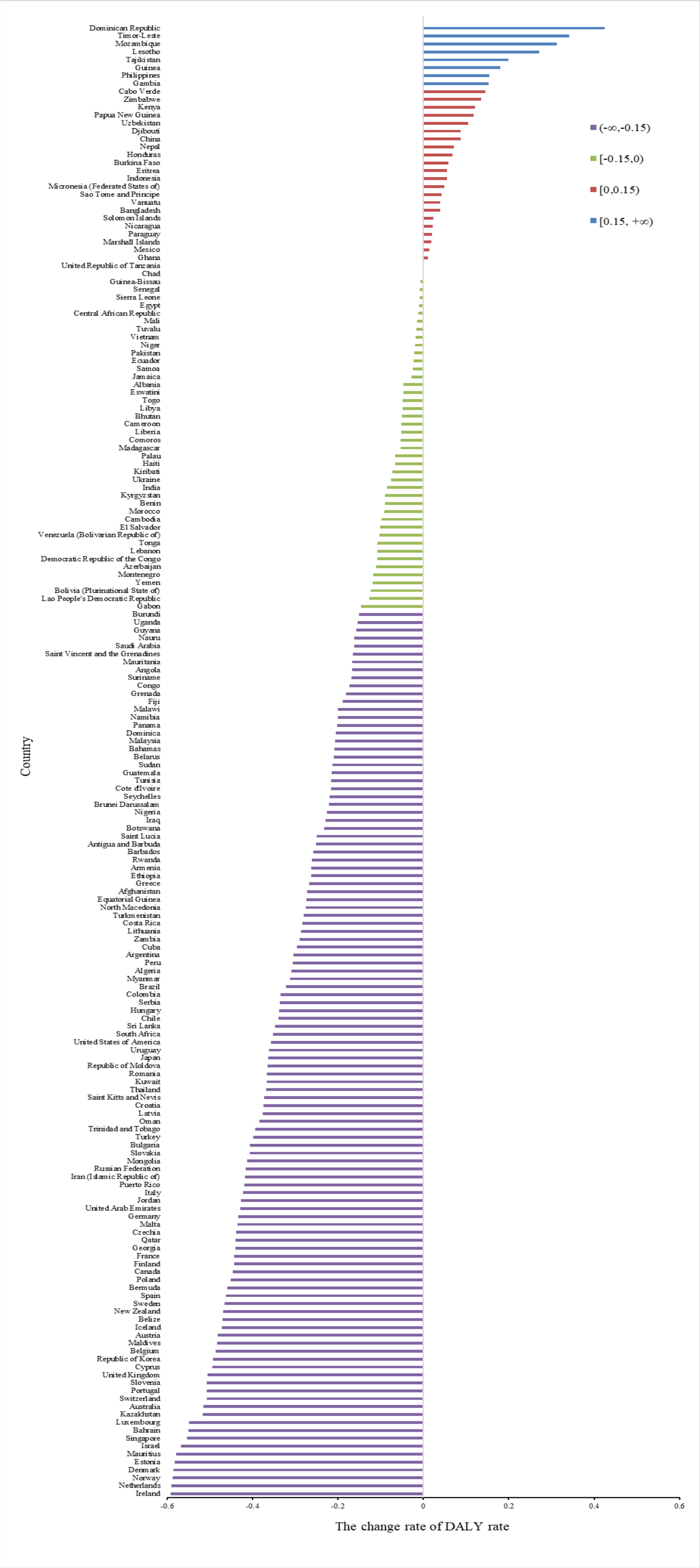

Fig. S3 The change rate of age-standardized DALY rates for stroke from 2000 to 2019

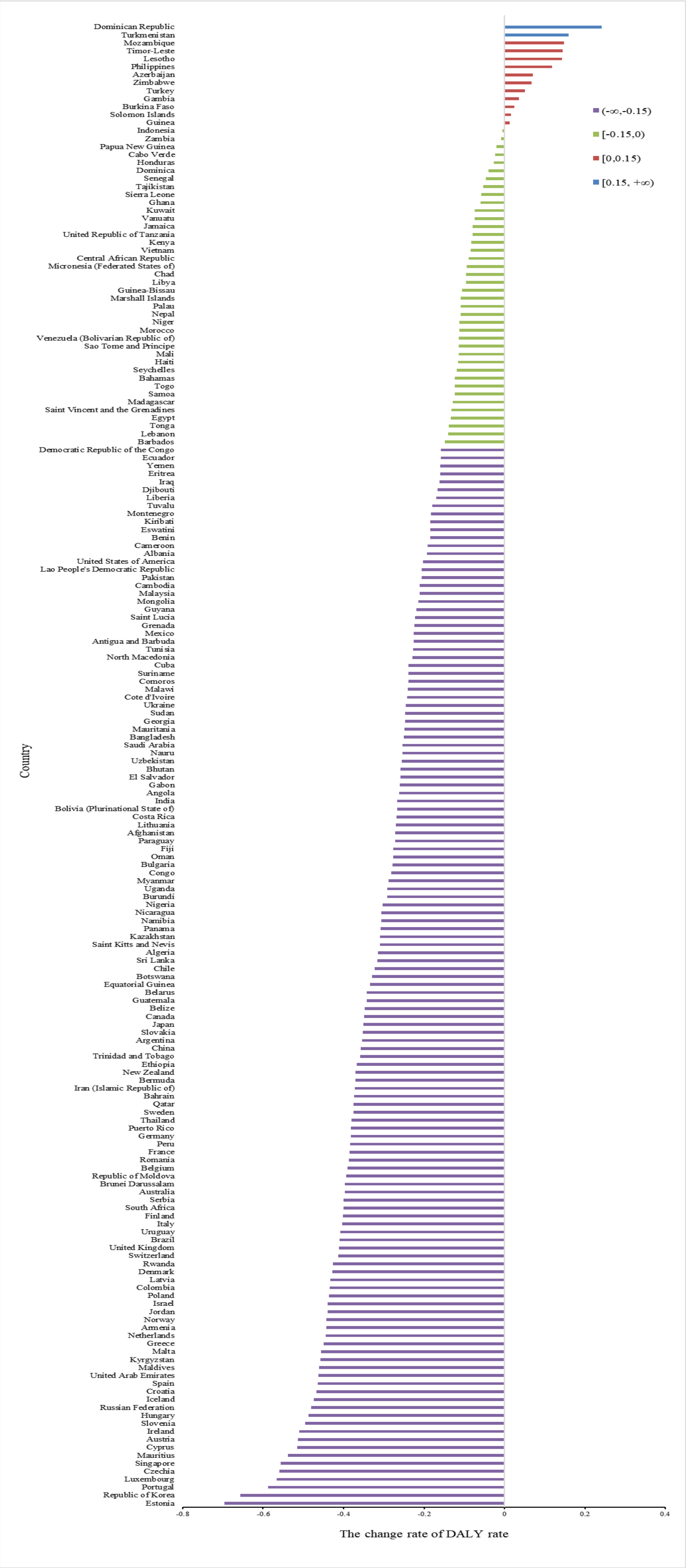

Fig. S4 The change rate of age-standardized DALY rates for hypertensive heart disease from 2000 to 2019

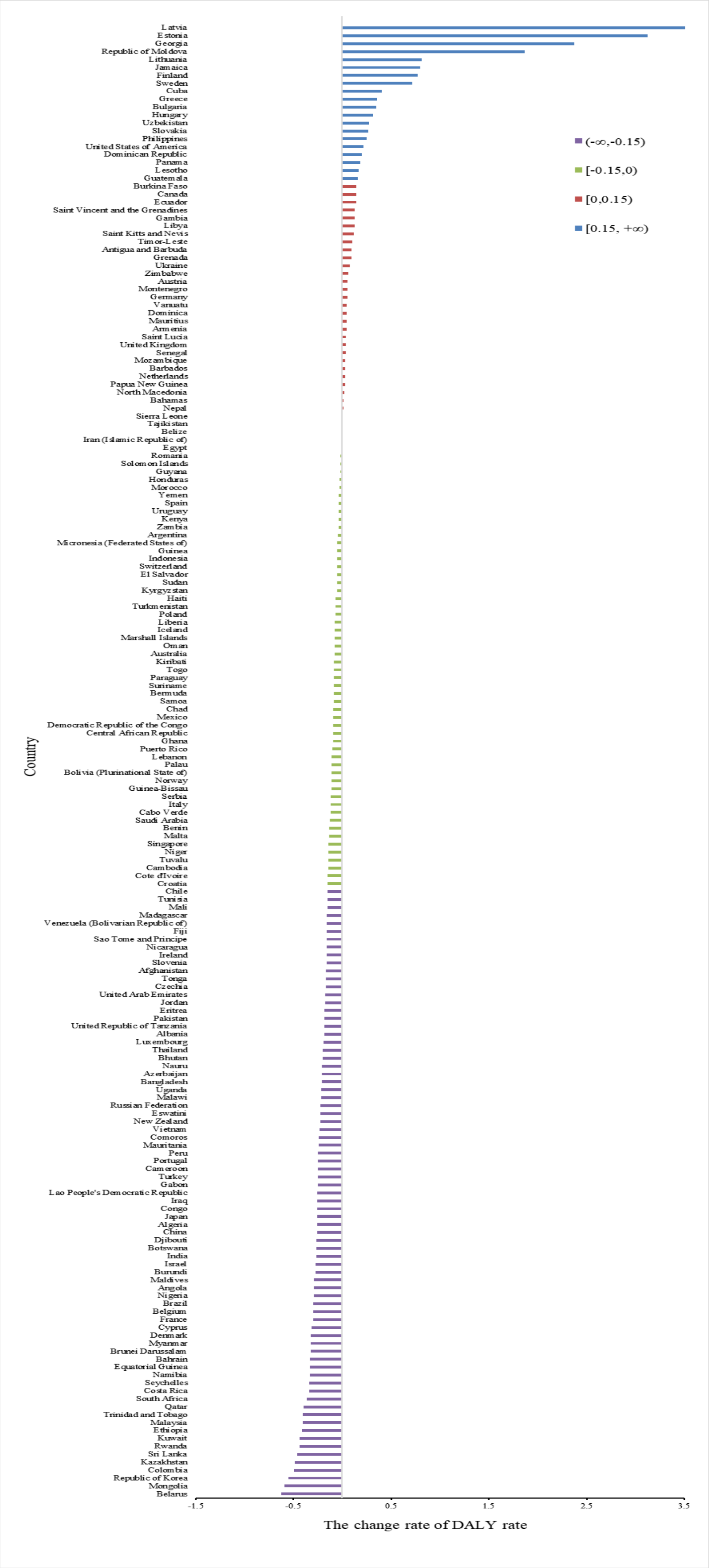

Fig. S5 The change rate of age-standardized DALY rates for non-rheumatic valvular heart disease from 2000 to 2019

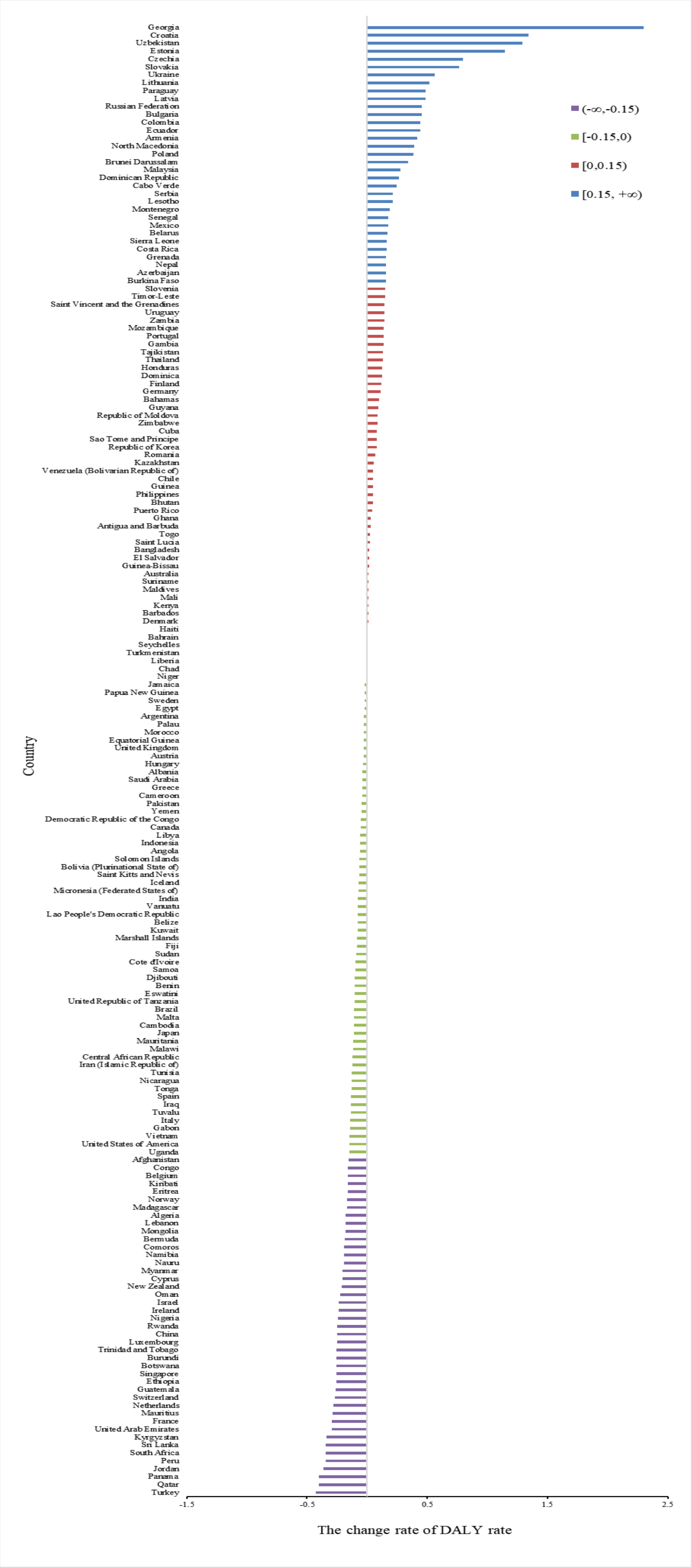

Fig. S6 The change rate of age-standardized DALY rates for cardiomyopathy and myocarditis from 2000 to 2019

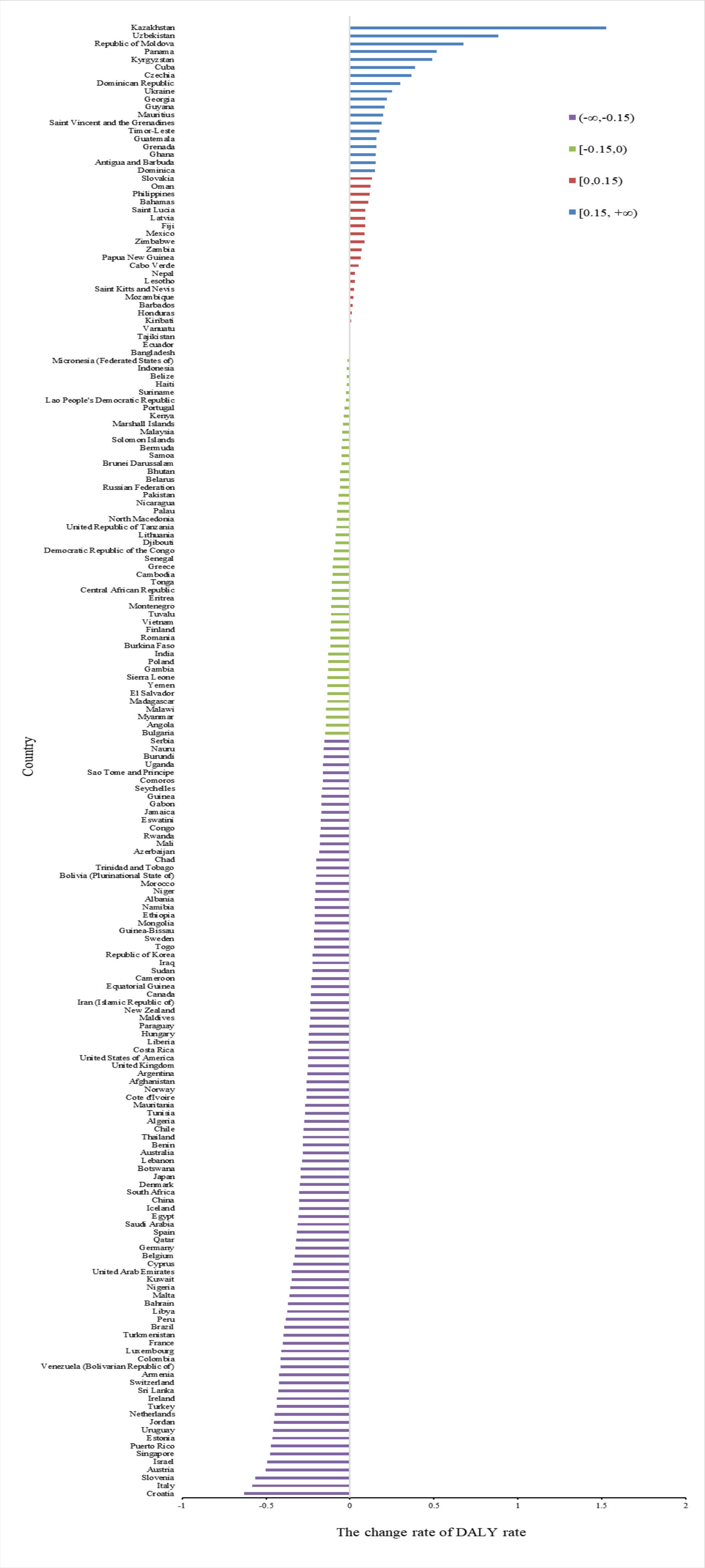

Fig. S7 The change rate of age-standardized DALY rates for atrial fibrillation and flutter from 2000 to 2019

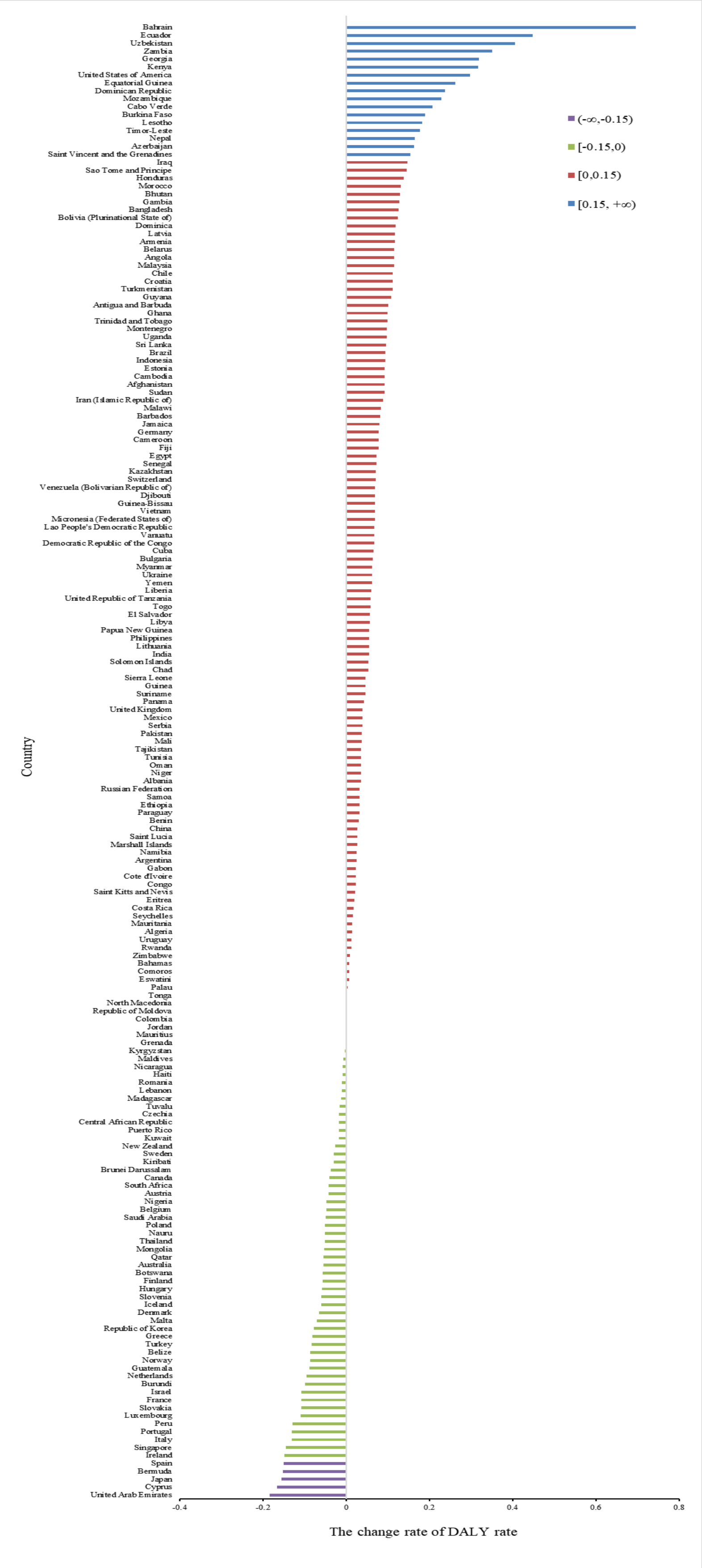

Fig. S8 The change rate of age-standardized DALY rates for aortic aneurysm from 2000 to 2019

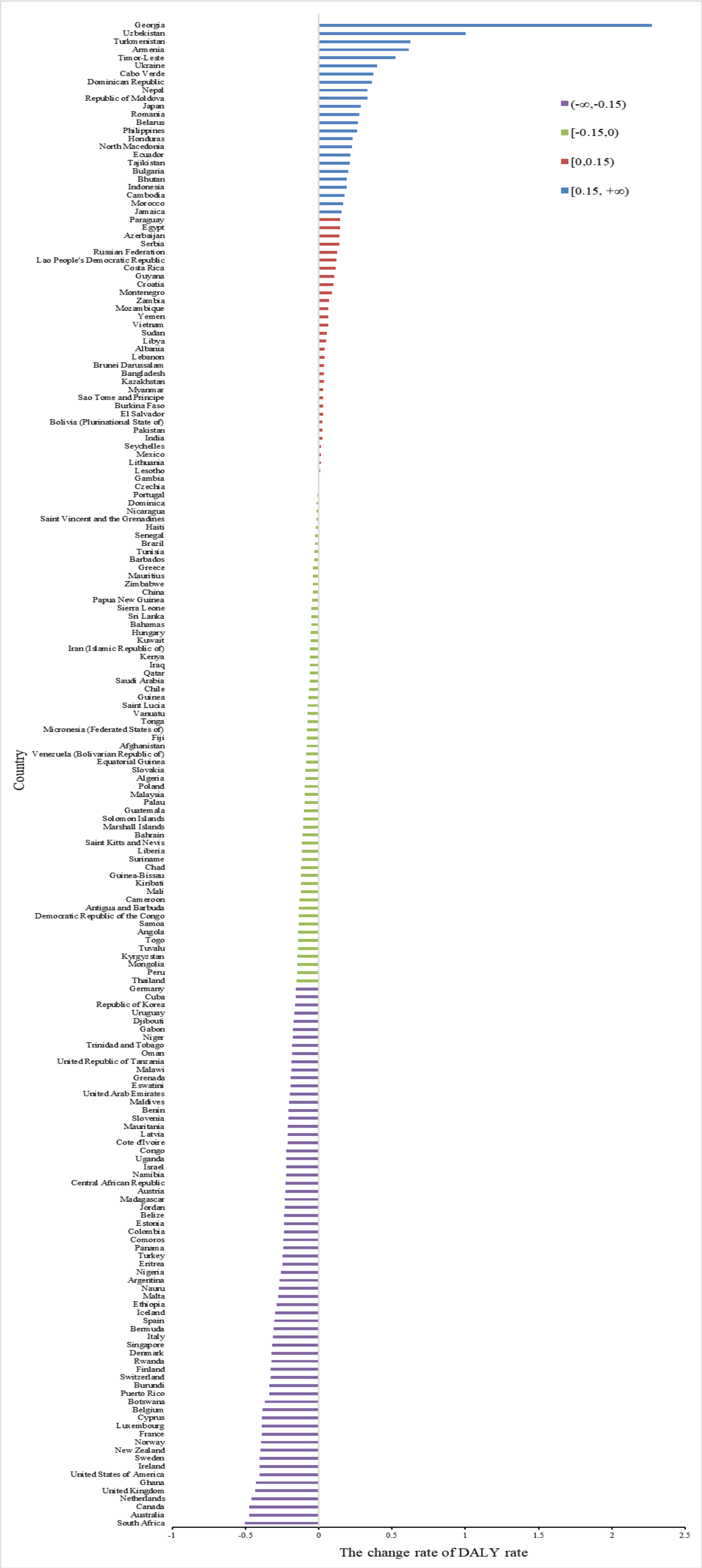

Fig. S9 The change rate of age-standardized DALY rates for peripheral artery disease from 2000 to 2019

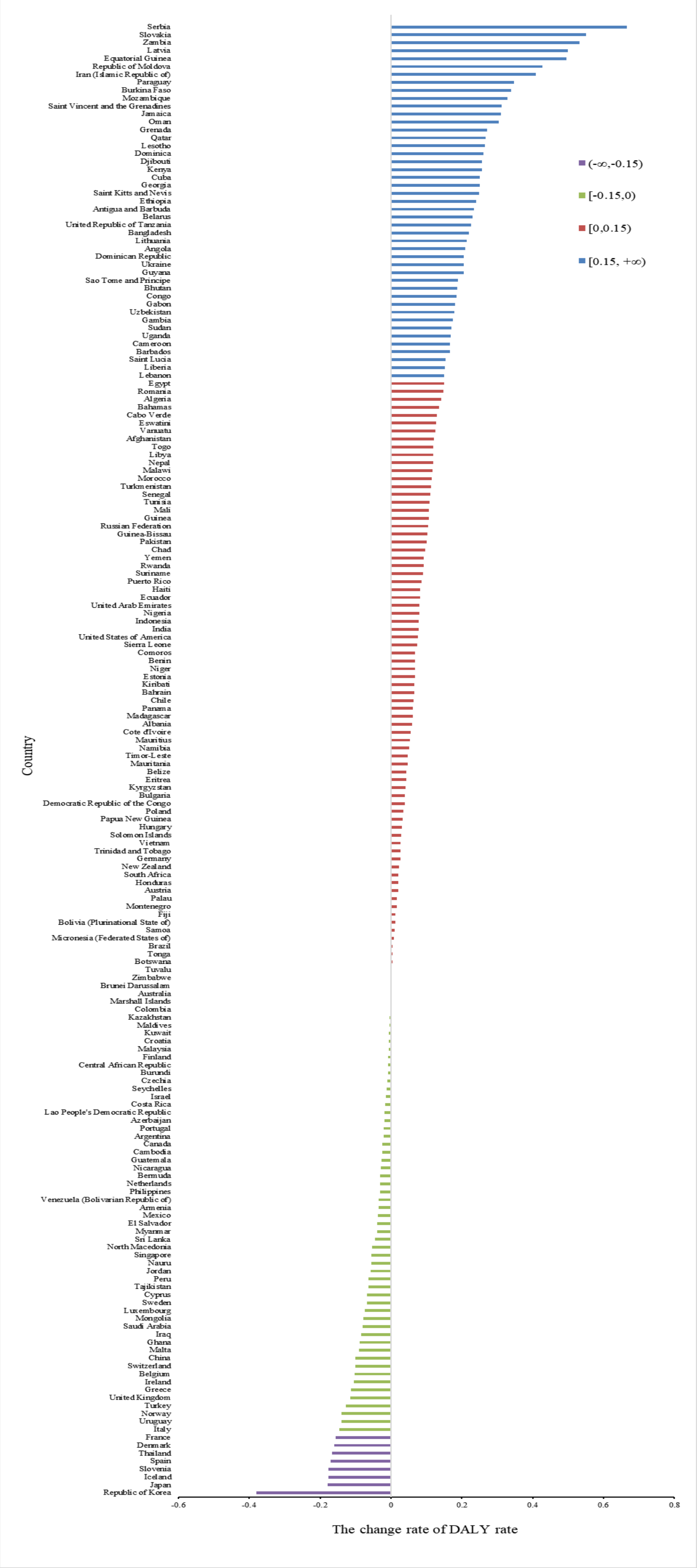

Fig. S10 The change rate of age-standardized DALY rates for endocarditis from 2000 to 2019

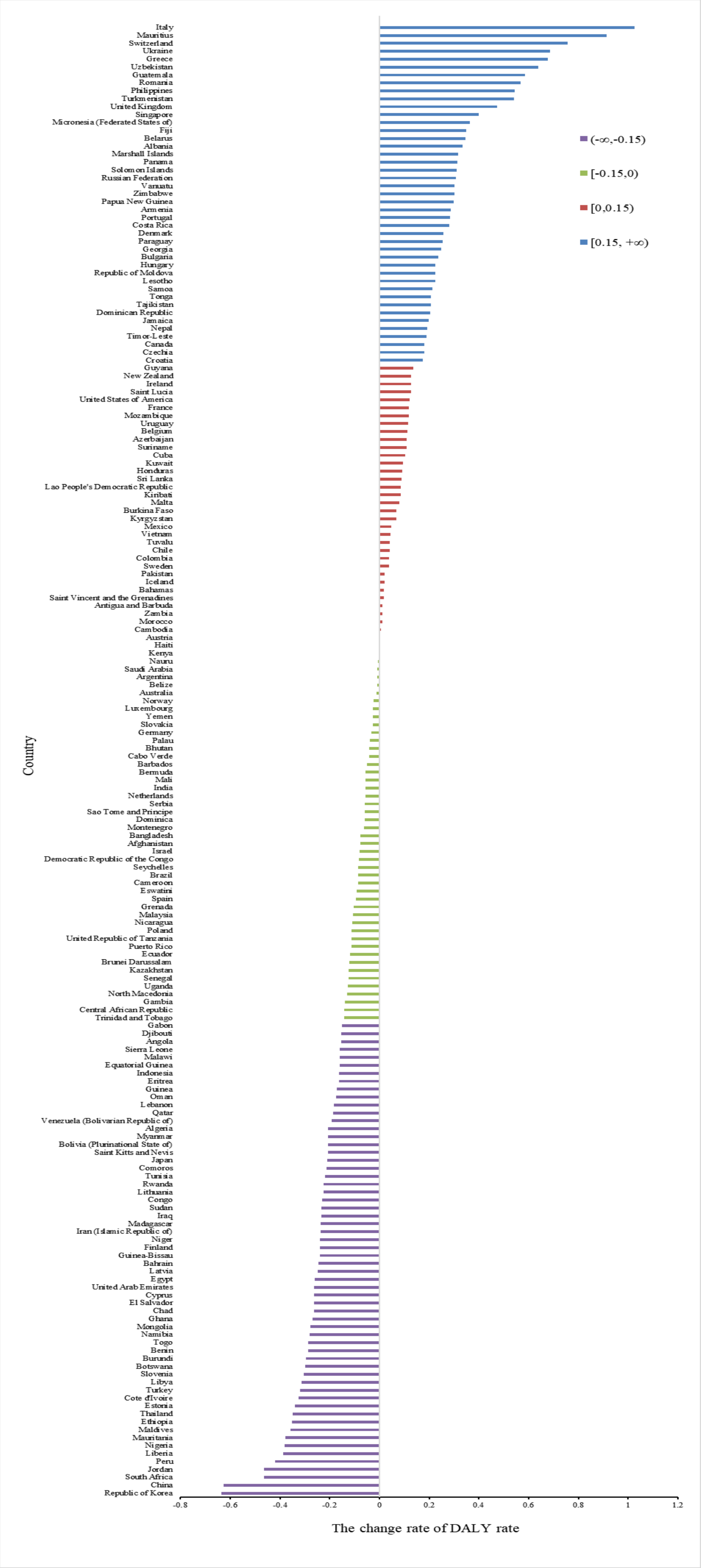

Fig. S11 The change rate of age-standardized DALY rates for other cardiovascular and circulatory diseases from 2000 to 2019

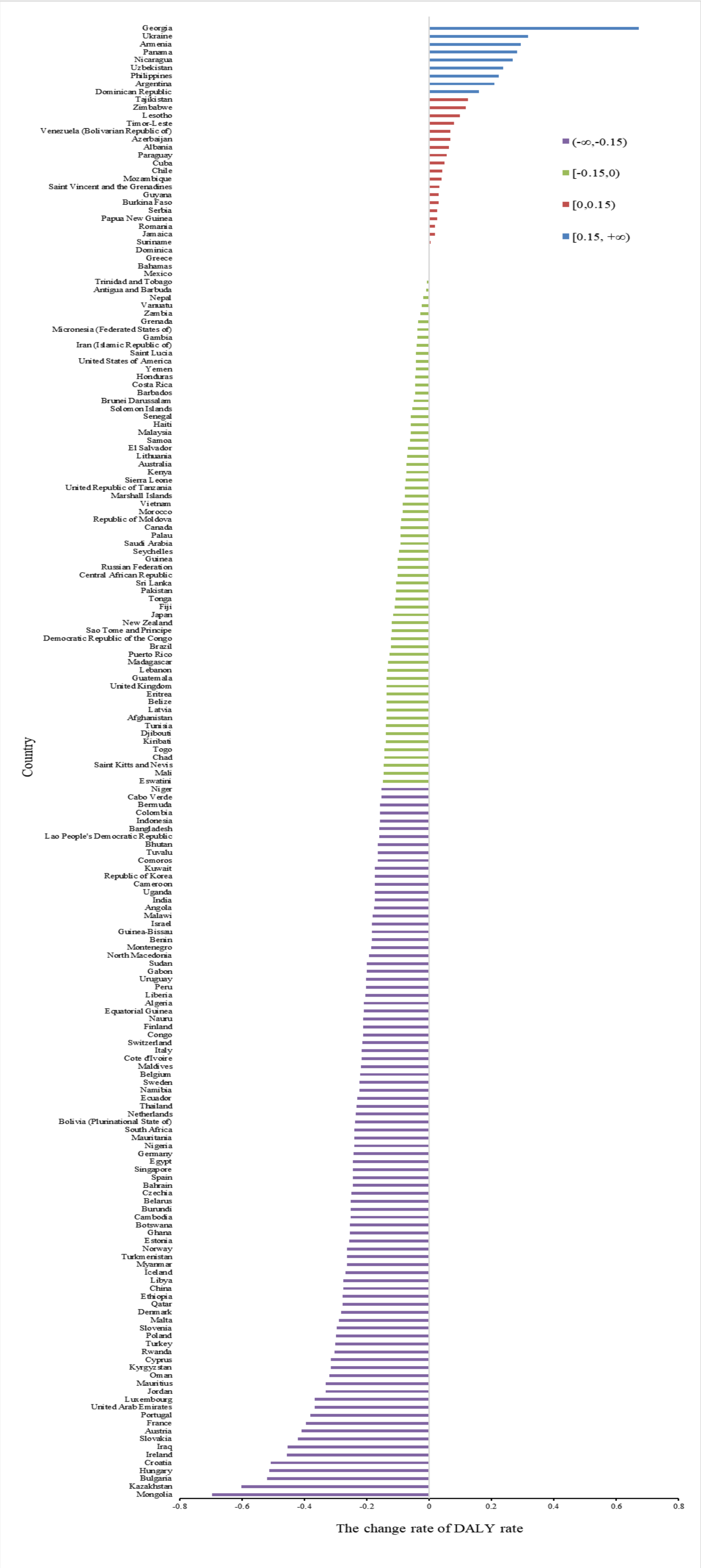

Figs. S12-29 Concentration curves of age-standardized DALY rates for CVD from 2001 to 2018

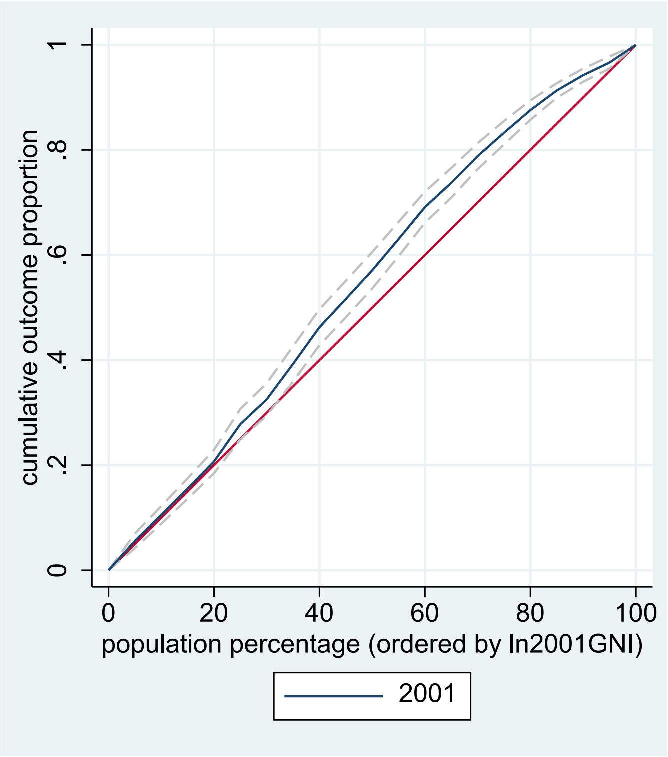

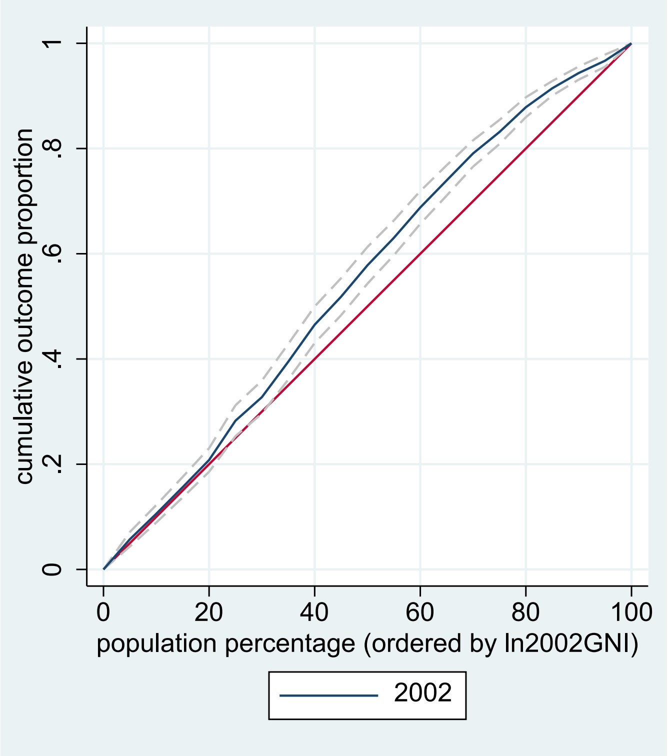

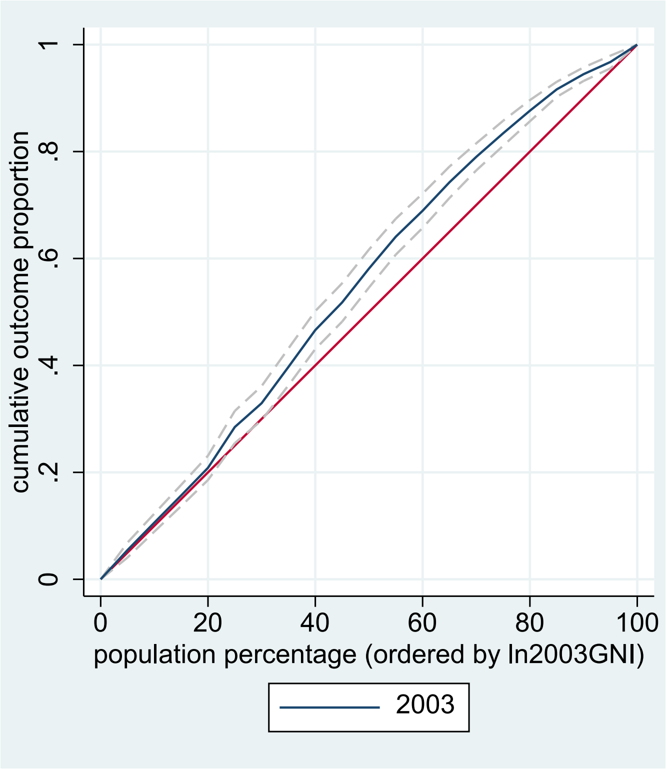
cumulative outcome proportion
0
.2
.4
.6
.8
1
0
20
40
60
80
100
population percentage (ordered by ln2003GNI)
2003

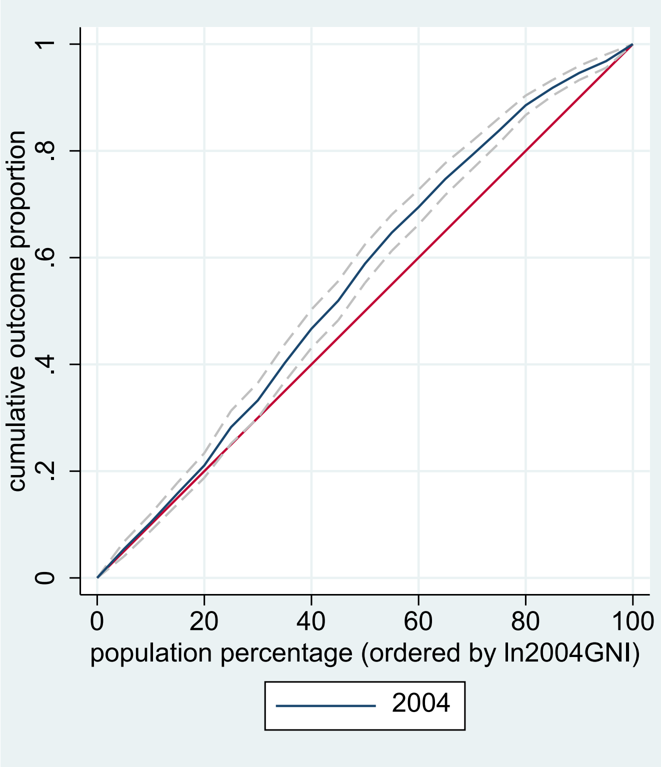
cumulative outcome proportion
0
.2
.4
.6
.8
1
0
20
40
60
80
100
population percentage (ordered by ln2004GNI)
2004

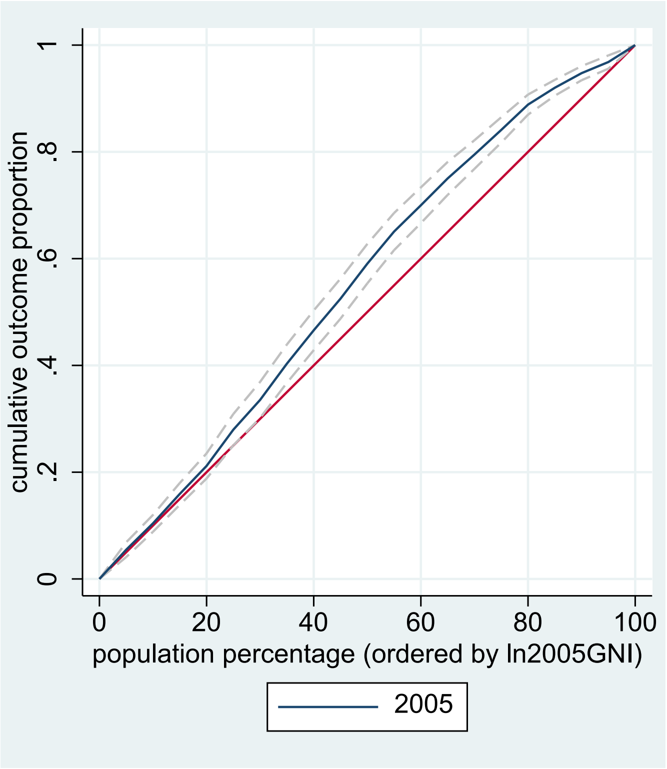

cumulative outcome proportion
0
.2
.4
.6
.8
1
0
20
40
60
80
100
population percentage (ordered by ln2005GNI)
2005

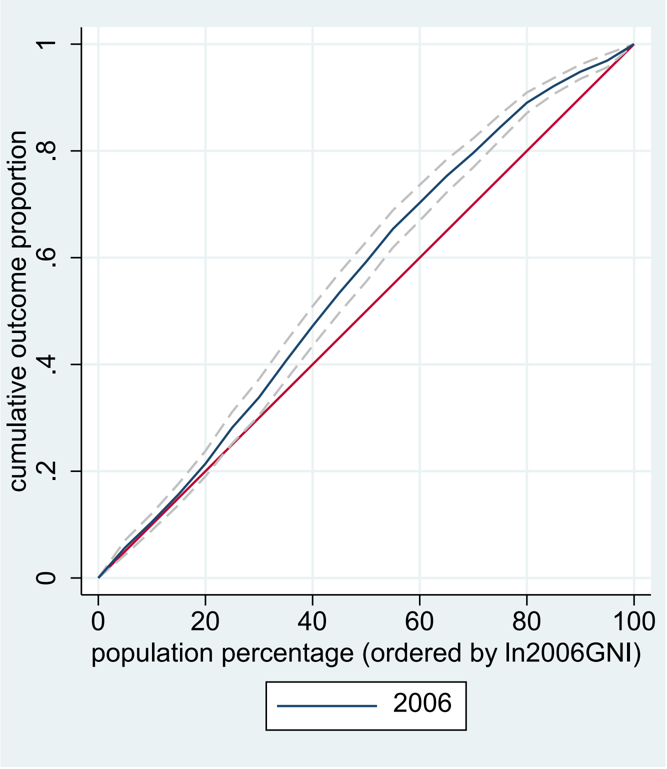

cumulative outcome proportion
0
.2
.4
.6
.8
1
0
20
40
60
80
100
population percentage (ordered by ln2006GNI)
2006

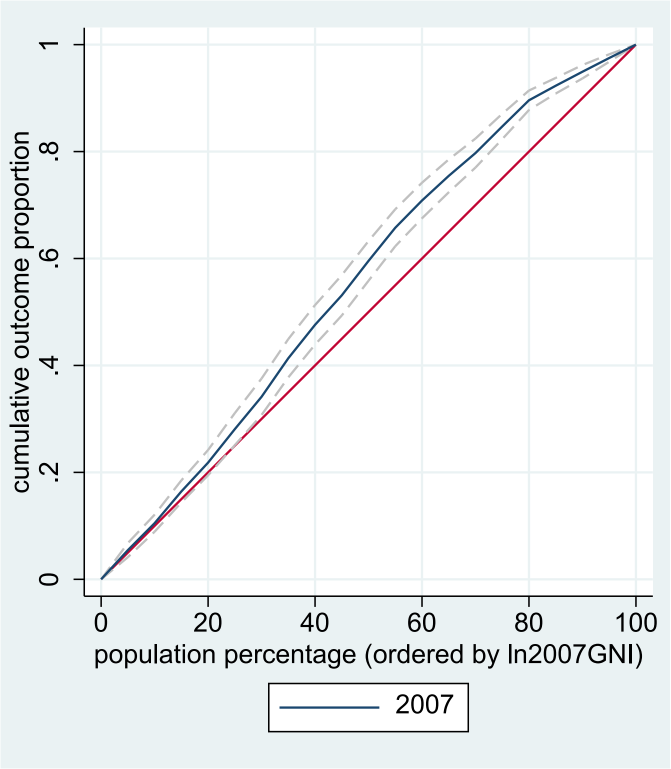

cumulative outcome proportion
0
.2
.4
.6
.8
1
0
20
40
60
80
100
population percentage (ordered by ln2007GNI)
2007

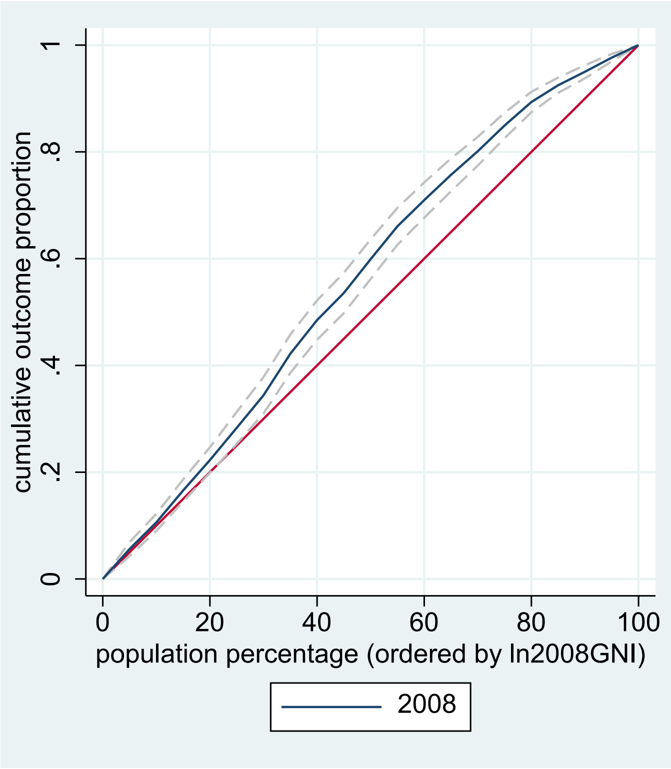

cumulative outcome proportion
0
.2
.4
.6
.8
1
0
20
40
60
80
100
population percentage (ordered by ln2008GNI)
2008

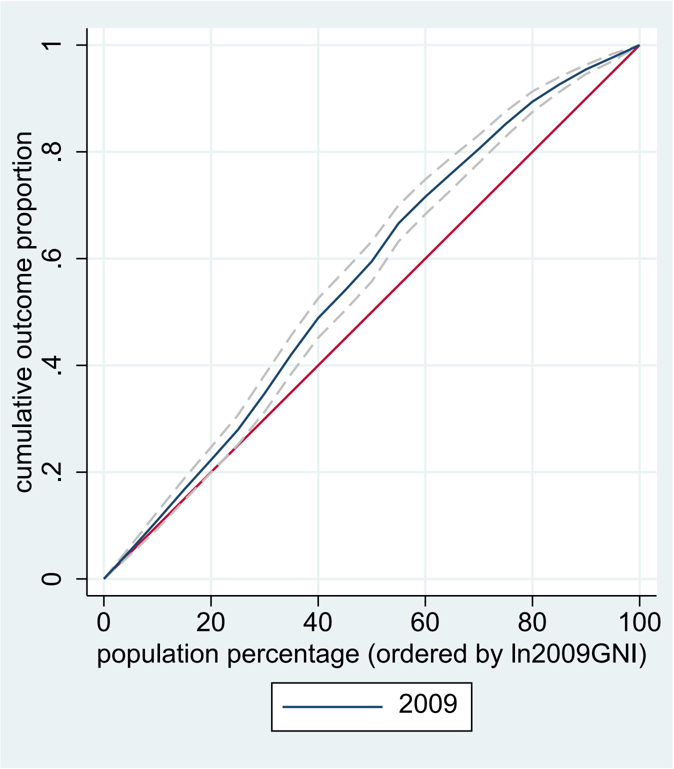
cumulative outcome proportion
0
.2
.4
.6
.8
1
0
20
40
60
80
100
population percentage (ordered by ln2009GNI)
2009

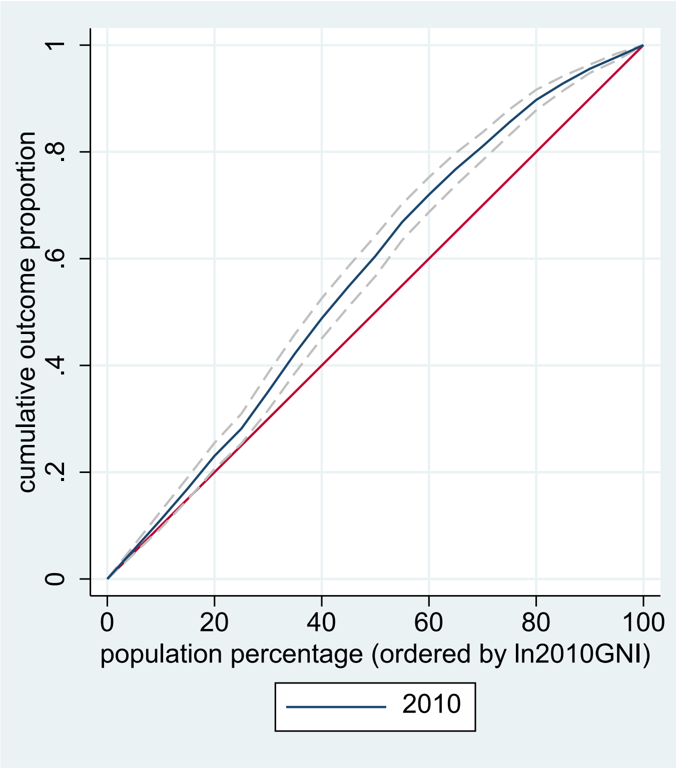
cumulative outcome proportion
0
.2
.4
.6
.8
1
0
20
40
60
80
100
population percentage (ordered by ln2010GNI)
2010

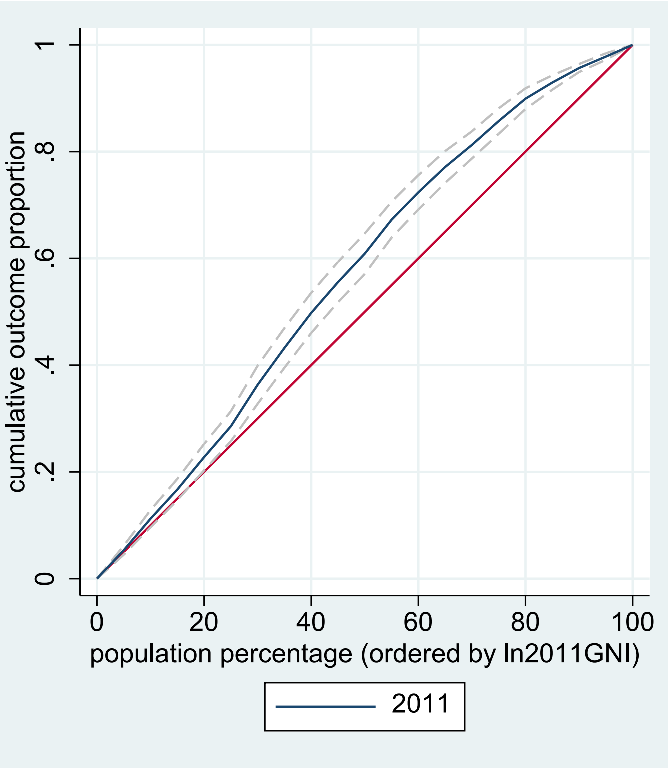
cumulative outcome proportion
0
.2
.4
.6
.8
1
0
20
40
60
80
100
population percentage (ordered by ln2011GNI)
2011

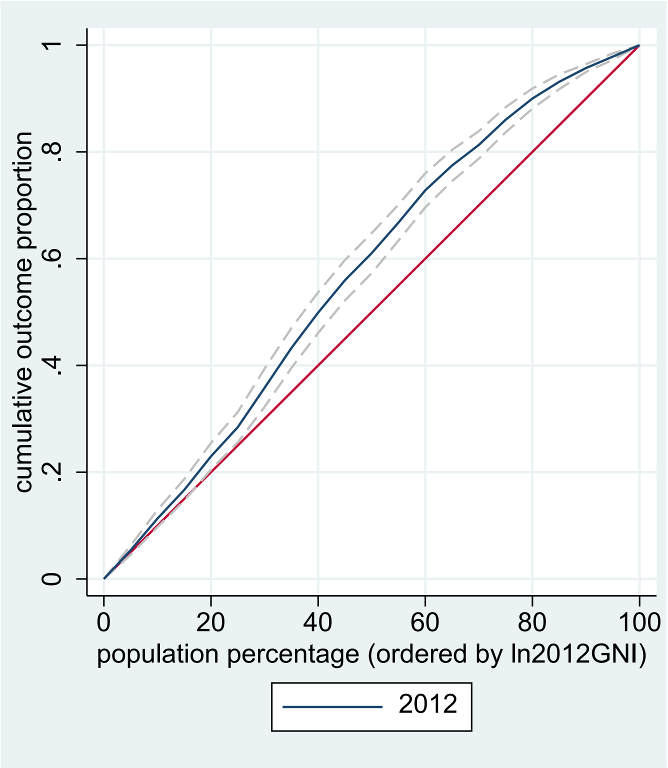
cumulative outcome proportion
0
.2
.4
.6
.8
1
0
20
40
60
80
100
population percentage (ordered by ln2012GNI)
2012

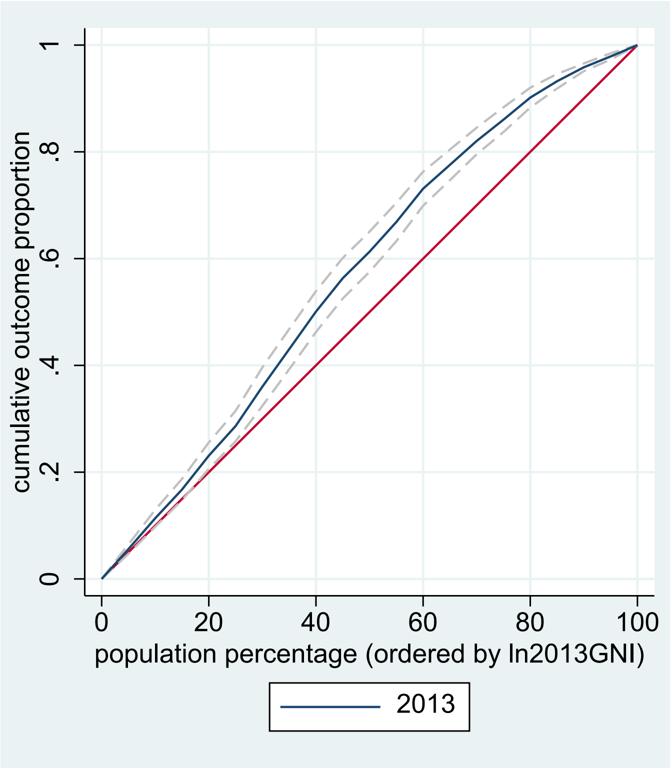
cumulative outcome proportion
0
.2
.4
.6
.8
1
0
20
40
60
80
100
population percentage (ordered by ln2013GNI)
2013

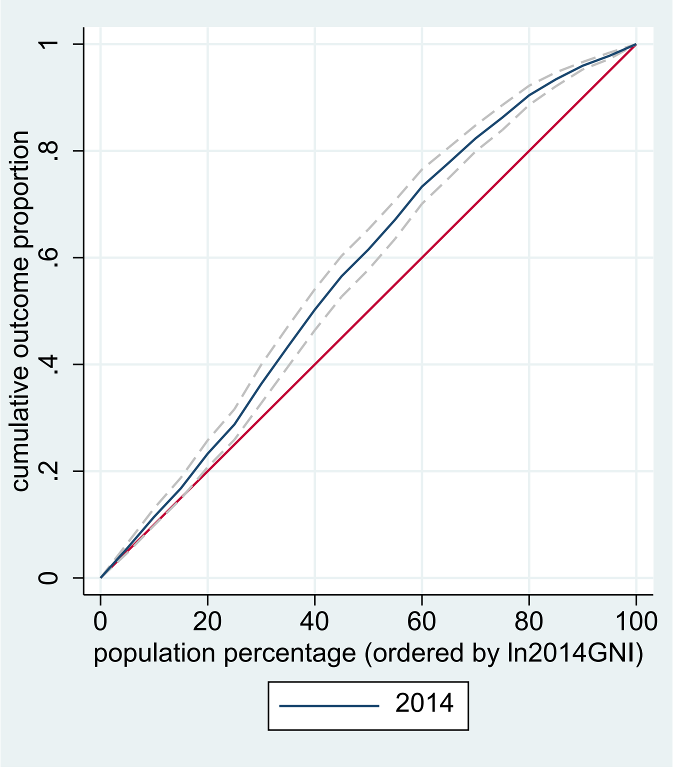
cumulative outcome proportion
0
.2
.4
.6
.8
1
0
20
40
60
80
100
population percentage (ordered by ln2014GNI)
2014

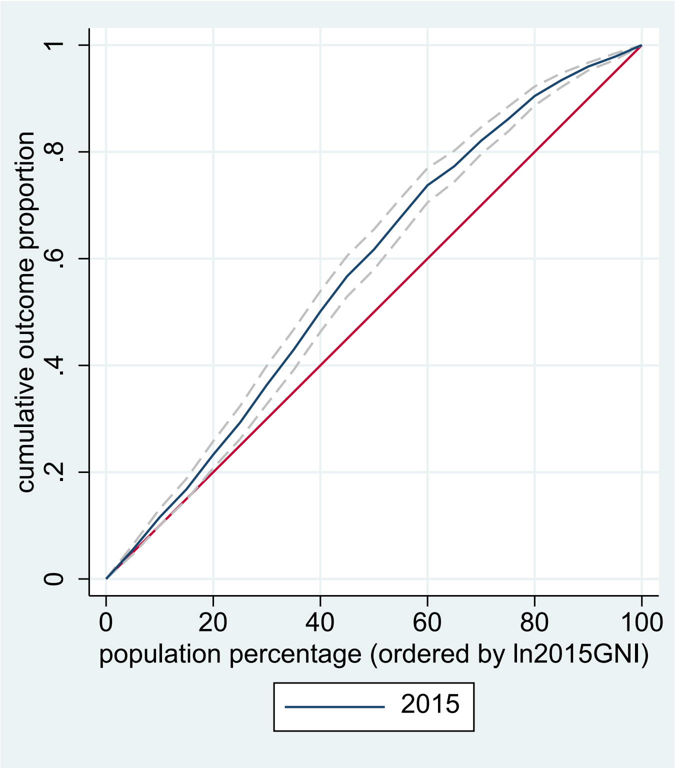
cumulative outcome proportion
0
.2
.4
.6
.8
1
0
20
40
60
80
100
population percentage (ordered by ln2015GNI)
2015

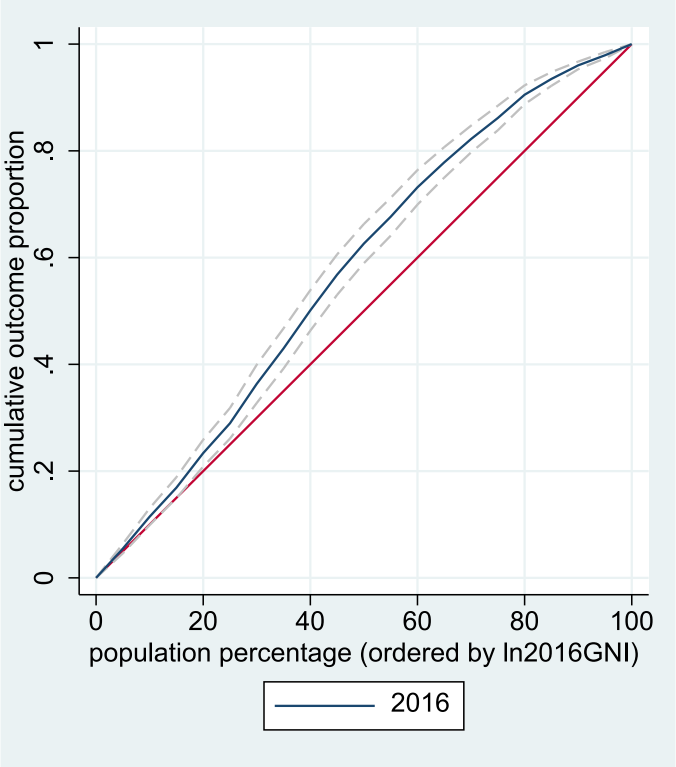
cumulative outcome proportion
0
.2
.4
.6
.8
1
0
20
40
60
80
100
population percentage (ordered by ln2016GNI)
2016

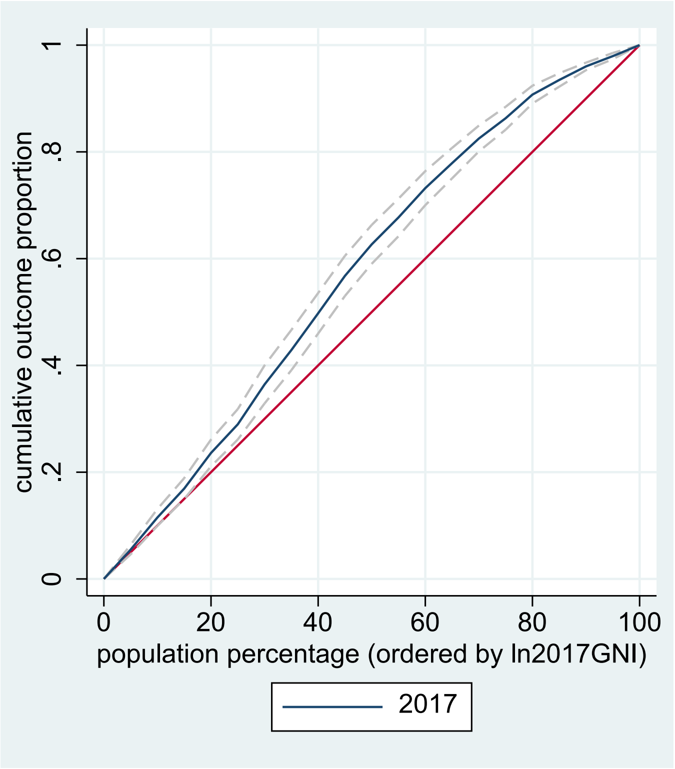
cumulative outcome proportion
0
.2
.4
.6
.8
1
0
20
40
60
80
100
population percentage (ordered by ln2017GNI)
2017

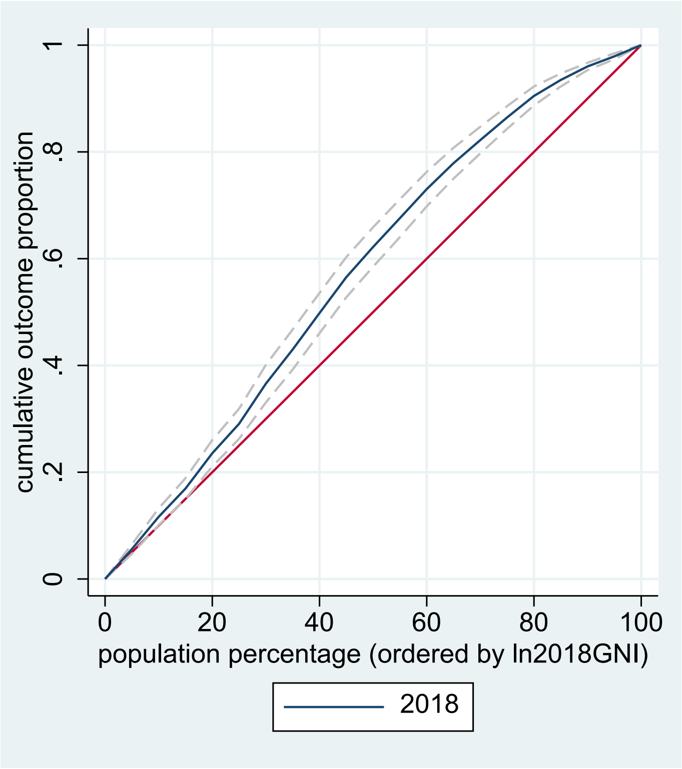
cumulative outcome proportion
0
.2
.4
.6
.8
1
0
20
40
60
80
100
population percentage (ordered by ln2018GNI)
2018

Figs. S30-47 Concentration curves of age-standardized DALY rates for rheumatic heart disease from 2001 to 2018

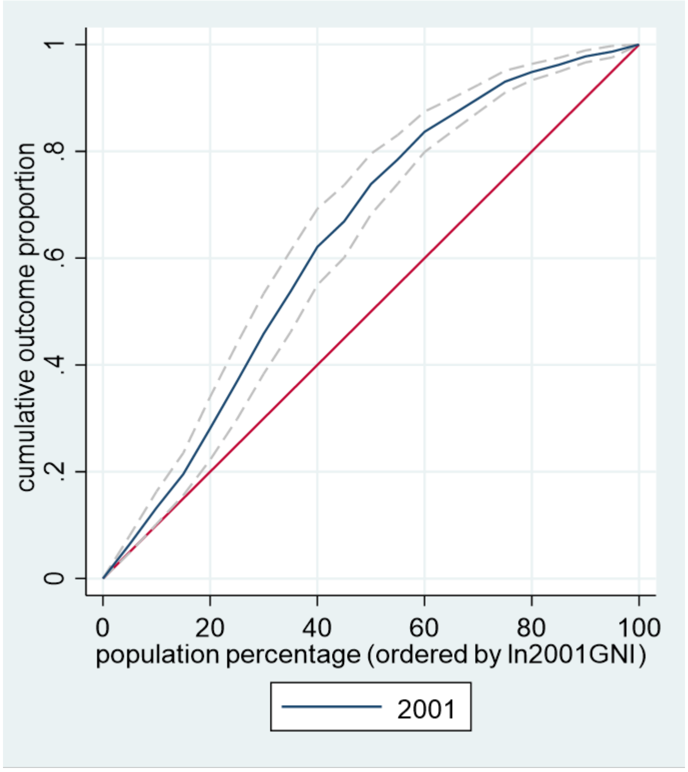

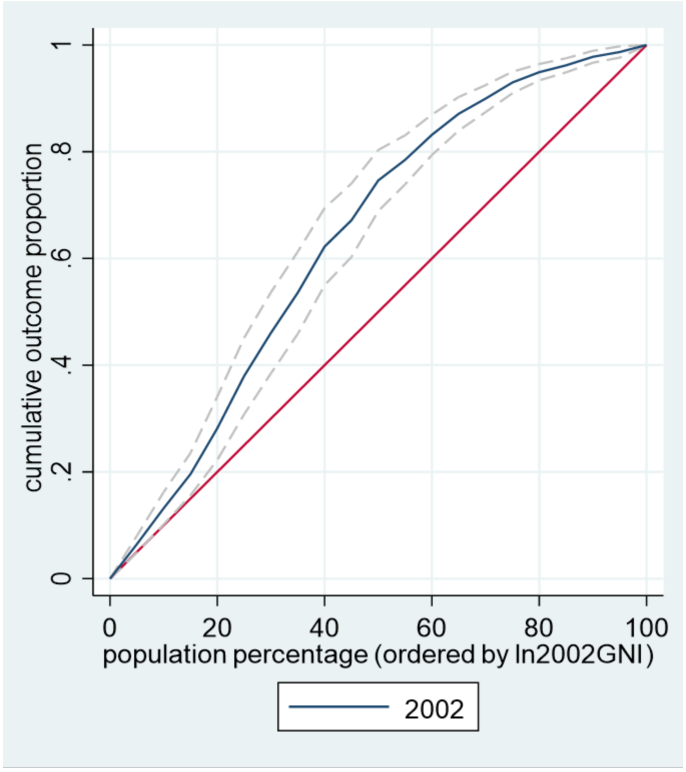

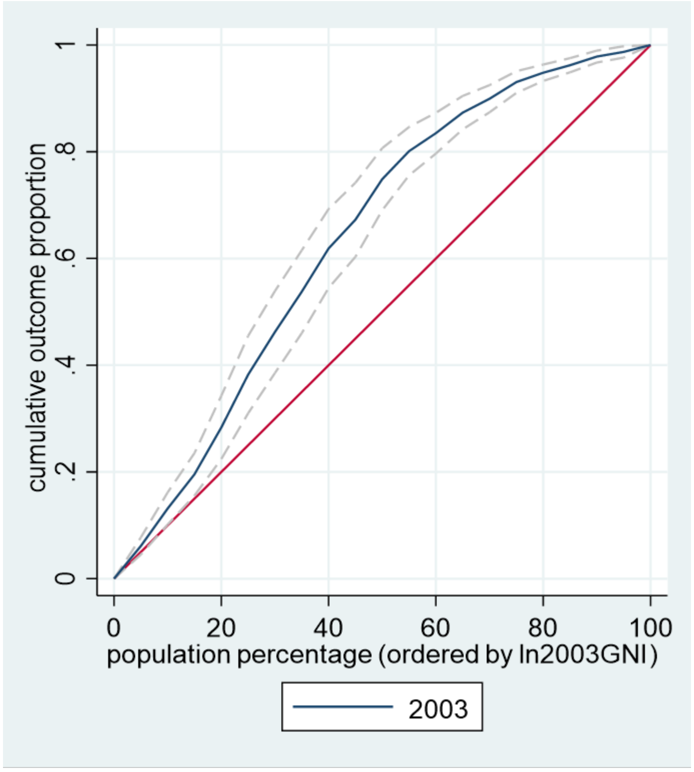
cumulative outcome proportion
0
.2
.4
.6
.8
1
0
20
40
60
80
100
population percentage (ordered by ln2003GNI)
2003

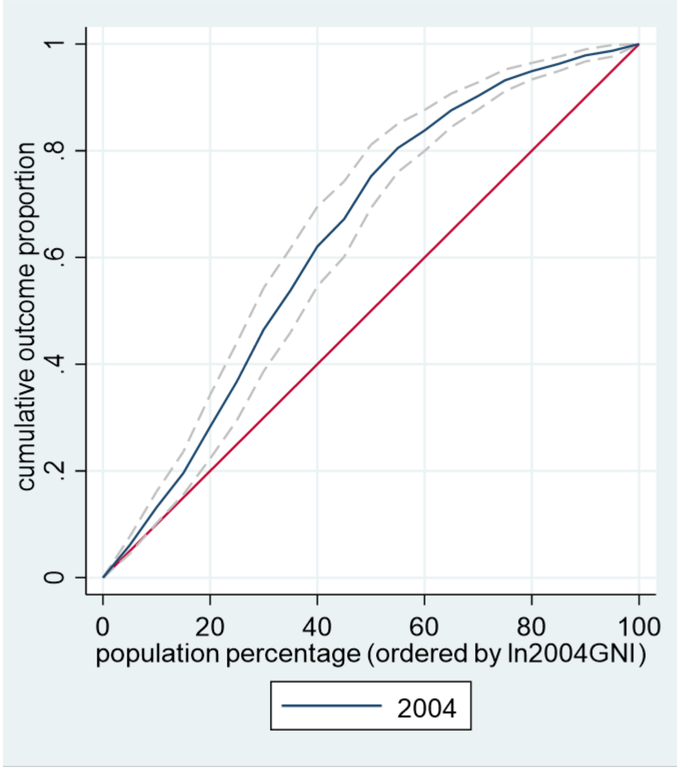
cumulative outcome proportion
0
.2
.4
.6
.8
1
0
20
40
60
80
100
population percentage (ordered by ln2004GNI)
2004

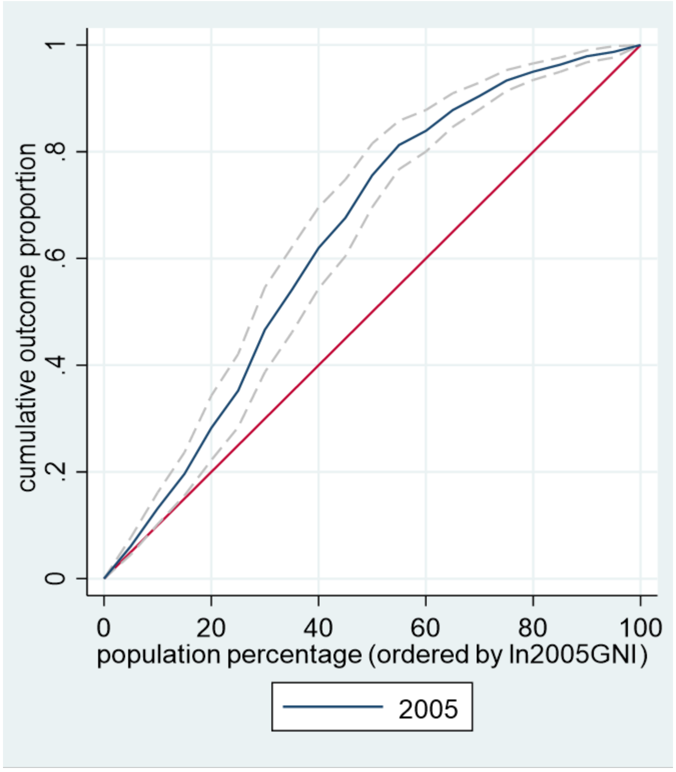
cumulative outcome proportion
0
.2
.4
.6
.8
1
0
20
40
60
80
100
population percentage (ordered by ln2005GNI)
2005

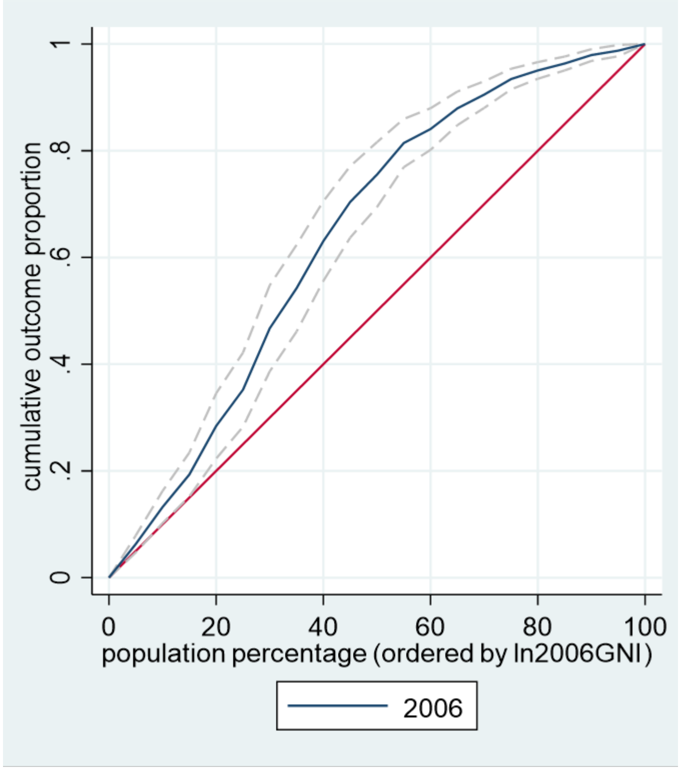
cumulative outcome proportion
0
.2
.4
.6
.8
1
0
20
40
60
80
100
population percentage (ordered by ln2006GNI)
2006

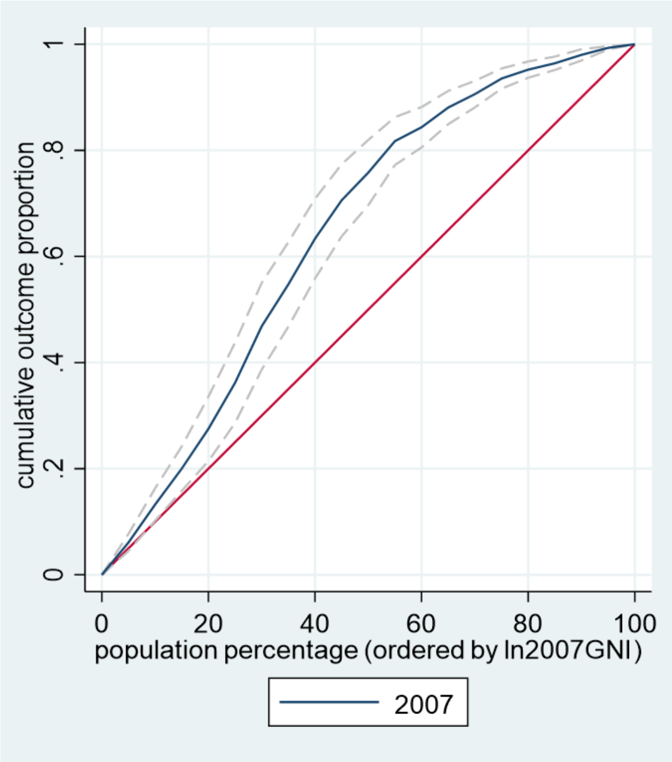
cumulative outcome proportion
0
.2
.4
.6
.8
1
0
20
40
60
80
100
population percentage (ordered by ln2007GNI)
2007

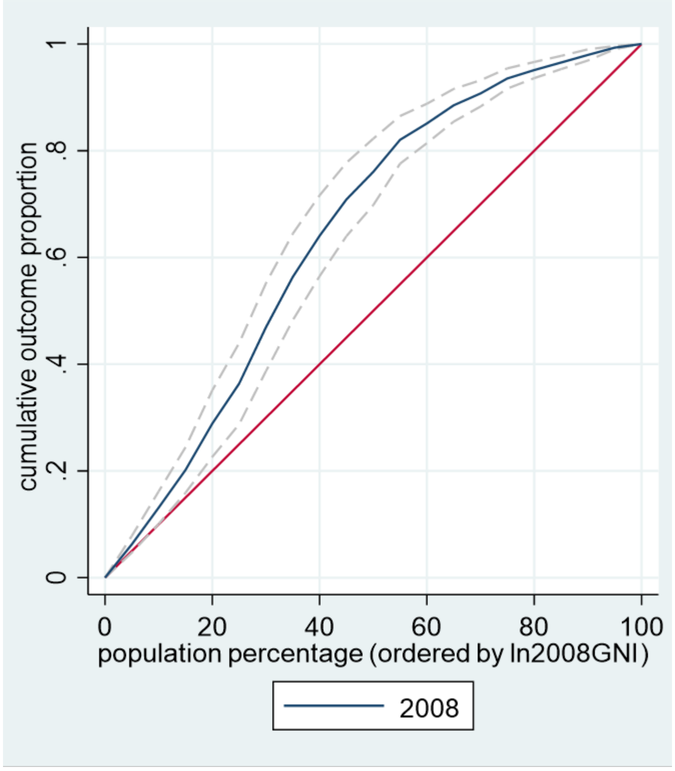
cumulative outcome proportion
0
.2
.4
.6
.8
1
0
20
40
60
80
100
population percentage (ordered by ln2008GNI)
2008

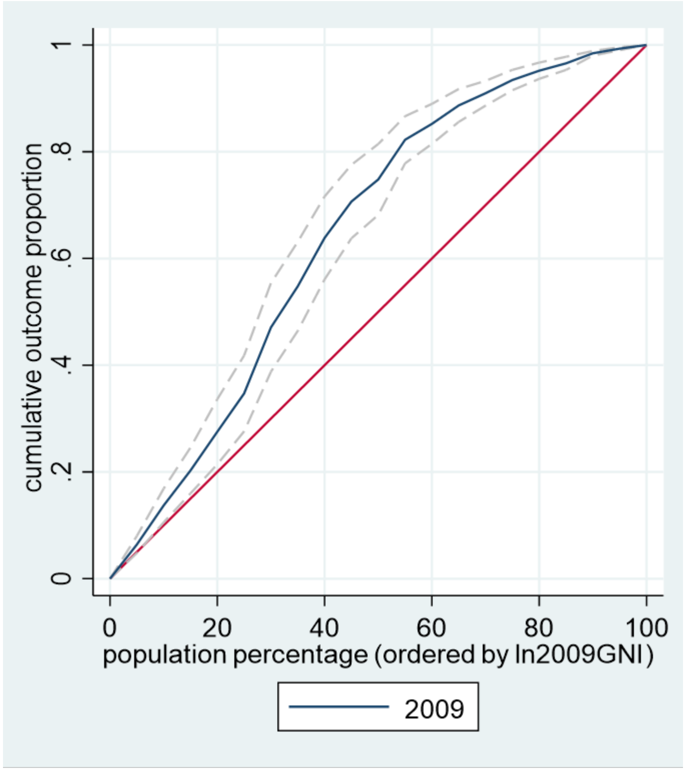
cumulative outcome proportion
0
.2
.4
.6
.8
1
0
20
40
60
80
100
population percentage (ordered by ln2009GNI)
2009

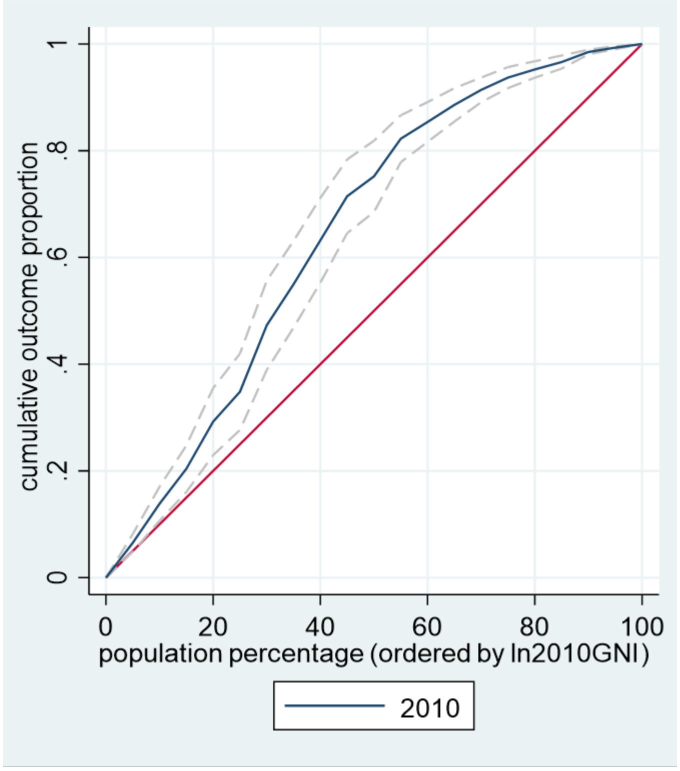
cumulative outcome proportion
0
.2
.4
.6
.8
1
0
20
40
60
80
100
population percentage (ordered by ln2010GNI)
2010

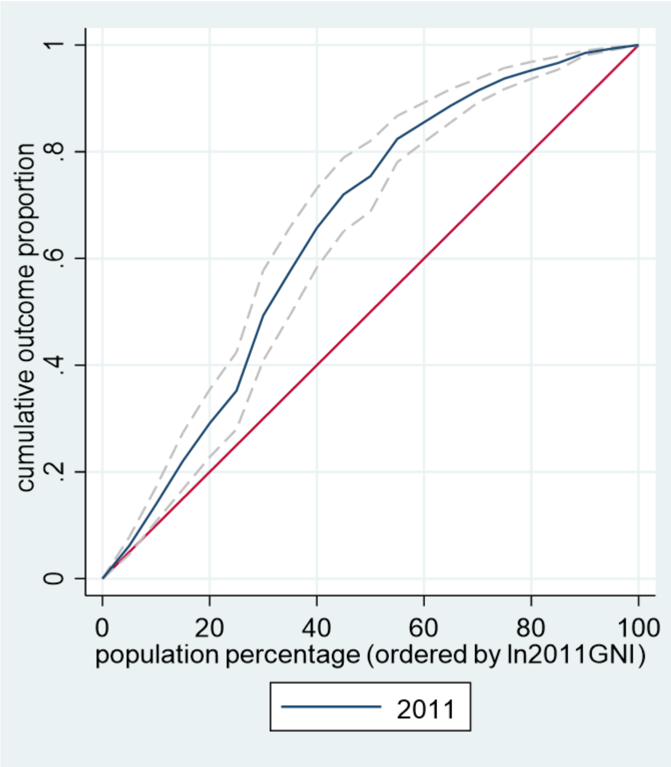

cumulative outcome proportion
0
.2
.4
.6
.8
1
0
20
40
60
80
100
population percentage (ordered by ln2011GNI)
2011

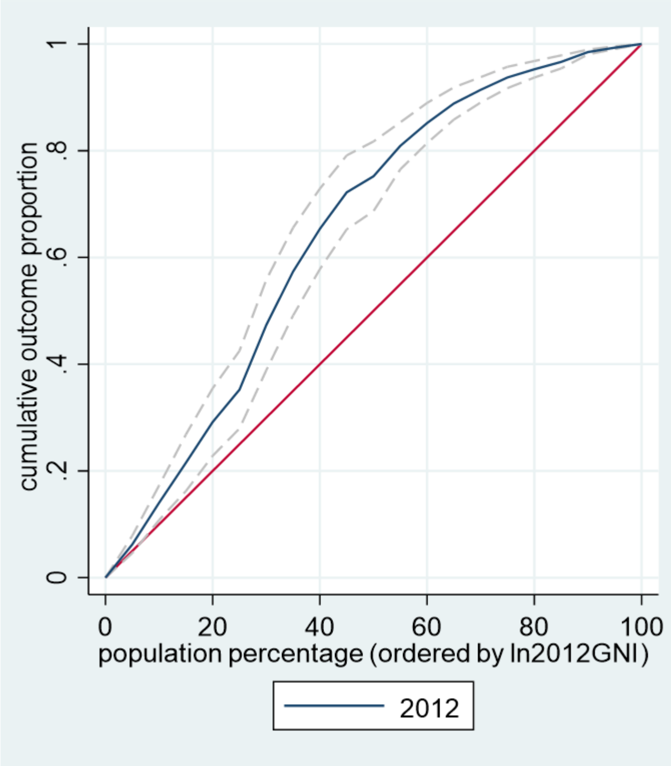

cumulative outcome proportion
0
.2
.4
.6
.8
1
0
20
40
60
80
100
population percentage (ordered by ln2012GNI)
2012

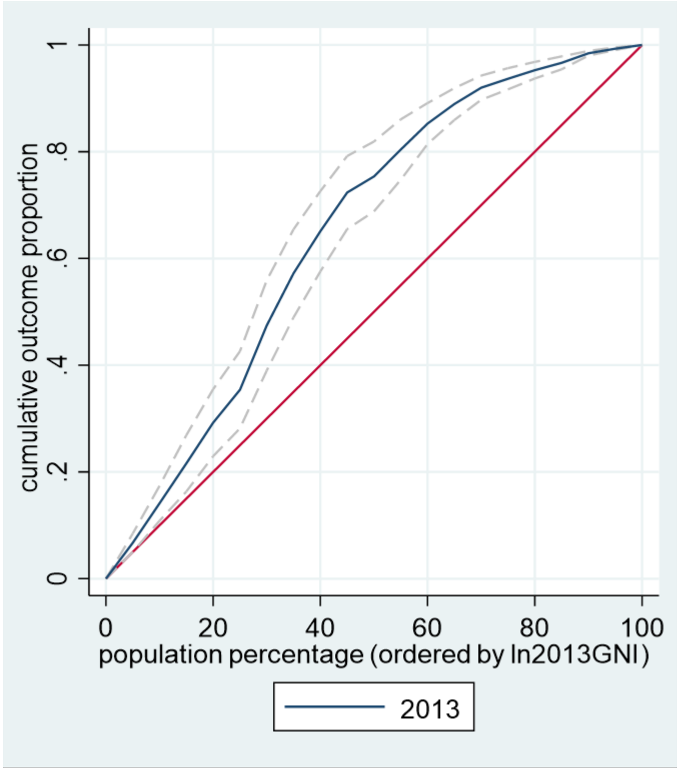

cumulative outcome proportion
0
.2
.4
.6
.8
1
0
20
40
60
80
100
population percentage (ordered by ln2013GNI)
2013

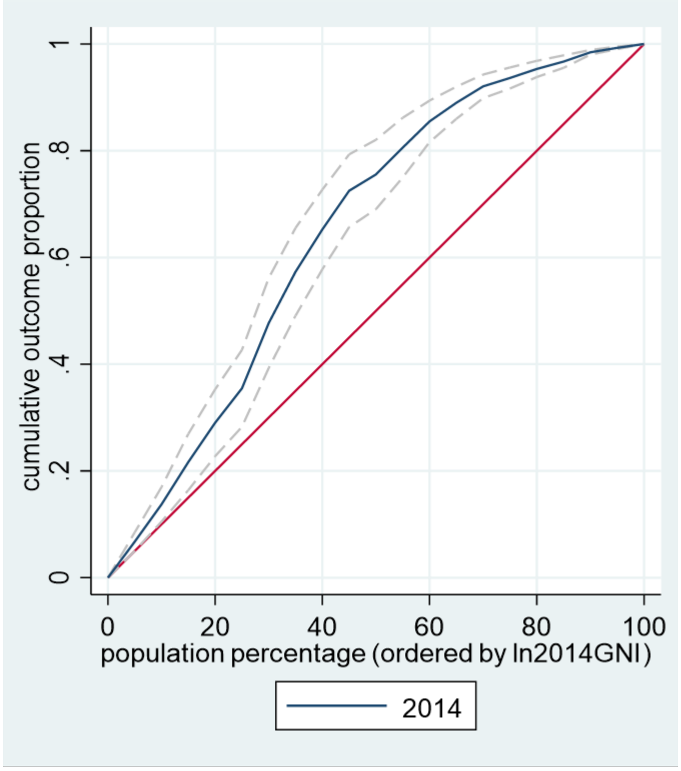

cumulative outcome proportion
0
.2
.4
.6
.8
1
0
20
40
60
80
100
population percentage (ordered by ln2014GNI)
2014

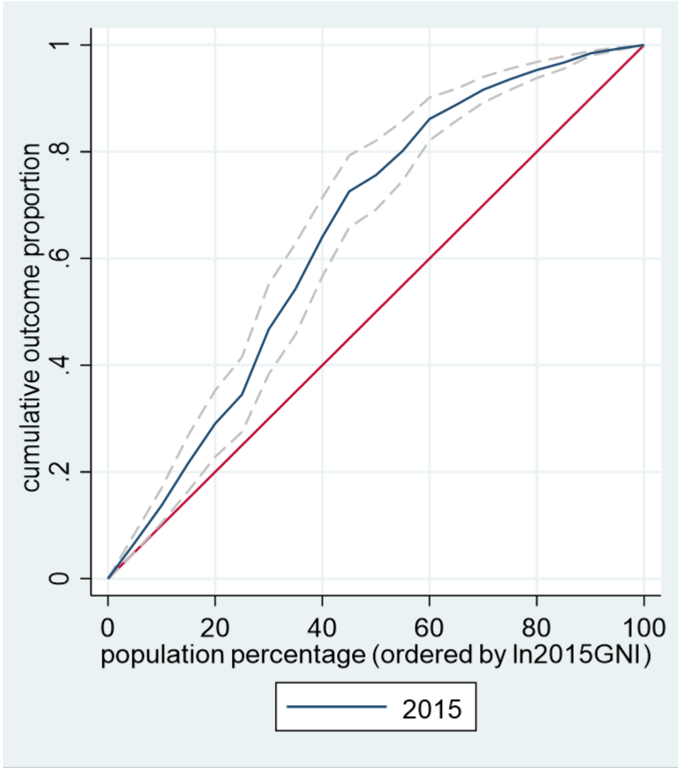
cumulative outcome proportion
0
.2
.4
.6
.8
1
0
20
40
60
80
100
population percentage (ordered by ln2015GNI)
2015

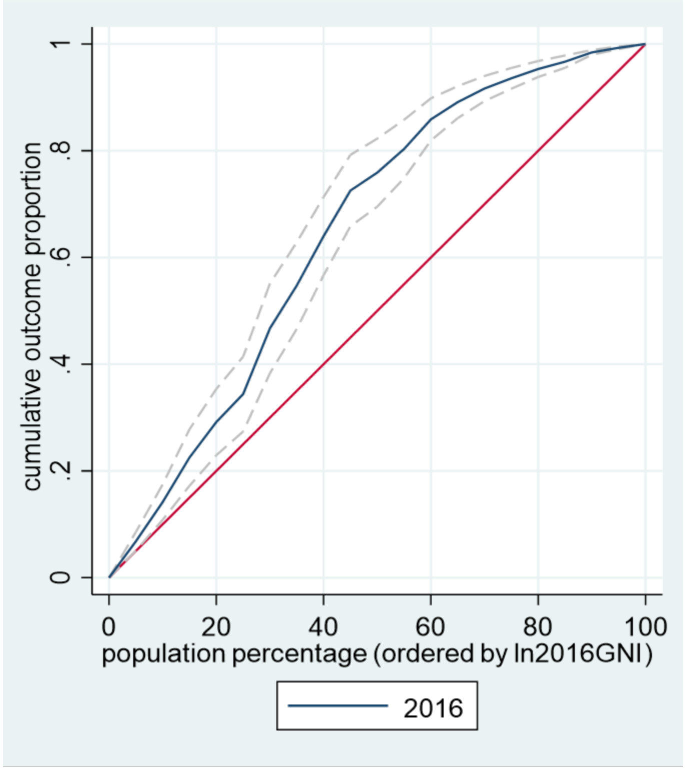
cumulative outcome proportion
0
.2
.4
.6
.8
1
0
20
40
60
80
100
population percentage (ordered by ln2016GNI)
2016

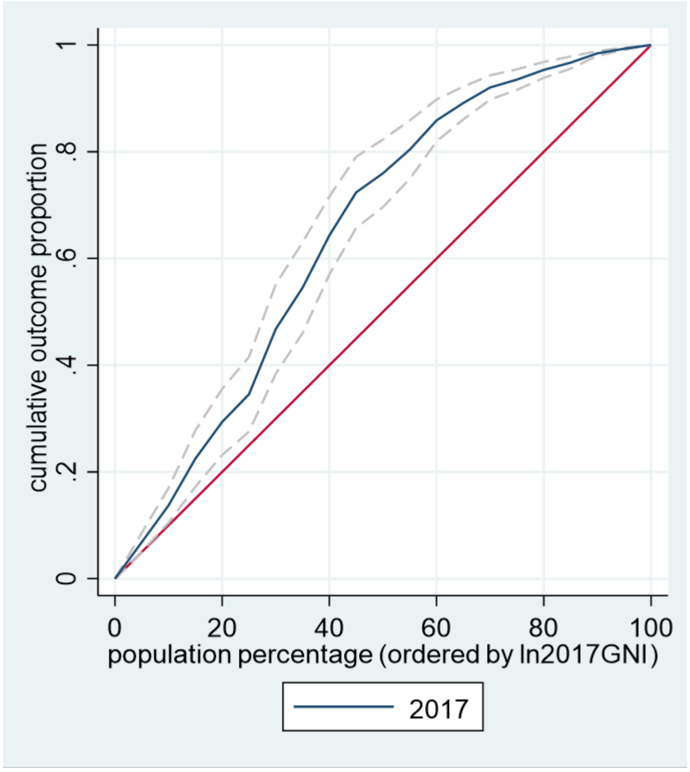

cumulative outcome proportion
0
.2
.4
.6
.8
1
0
20
40
60
80
100
population percentage (ordered by ln2017GNI)
2017

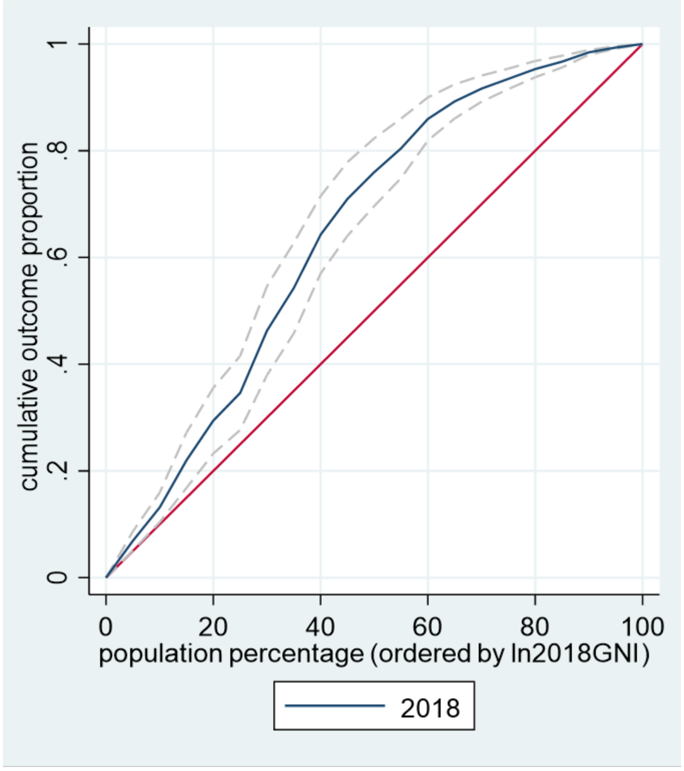

cumulative outcome proportion
0
.2
.4
.6
.8
1
0
20
40
60
80
100
population percentage (ordered by ln2018GNI)
2018

Figs. S48-65 Concentration curves of age-standardized DALY rates for ischemic heart disease from 2001 to 2018

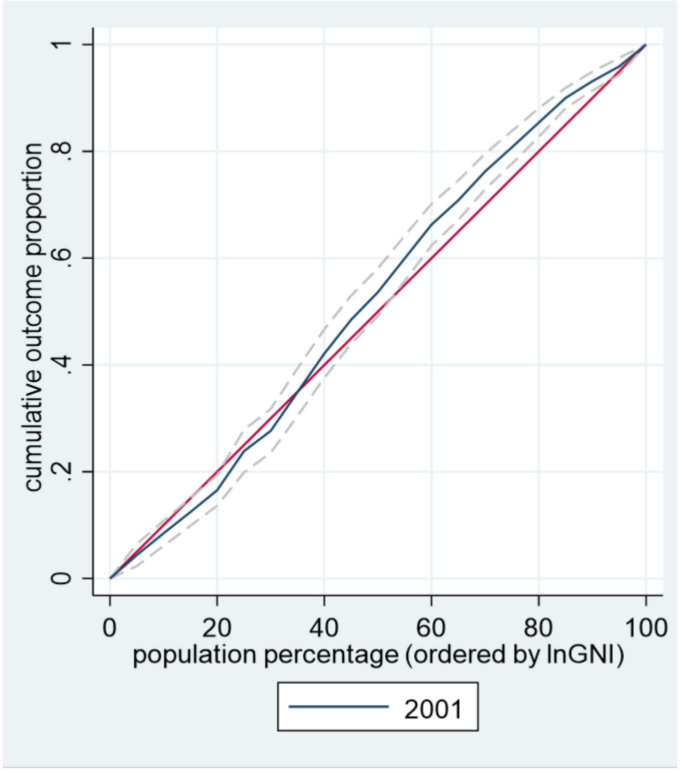

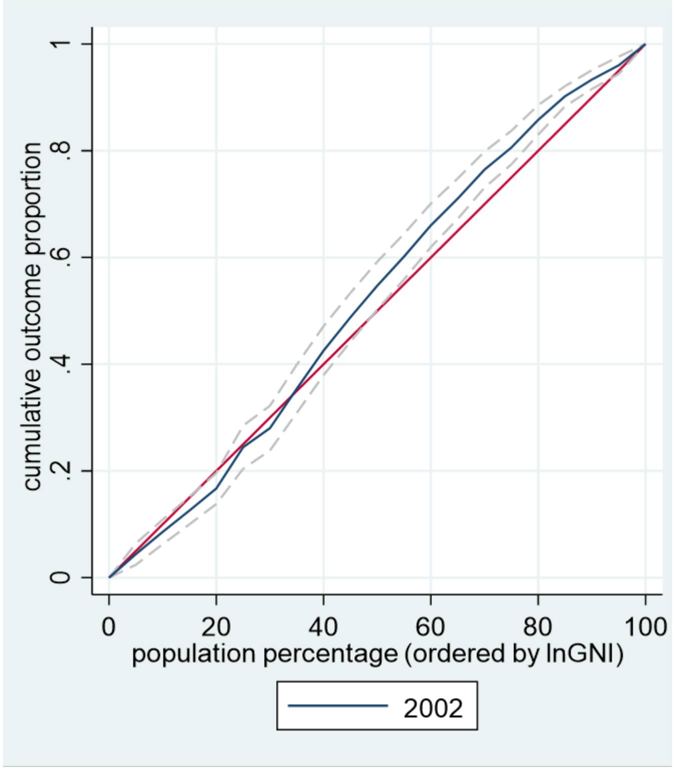

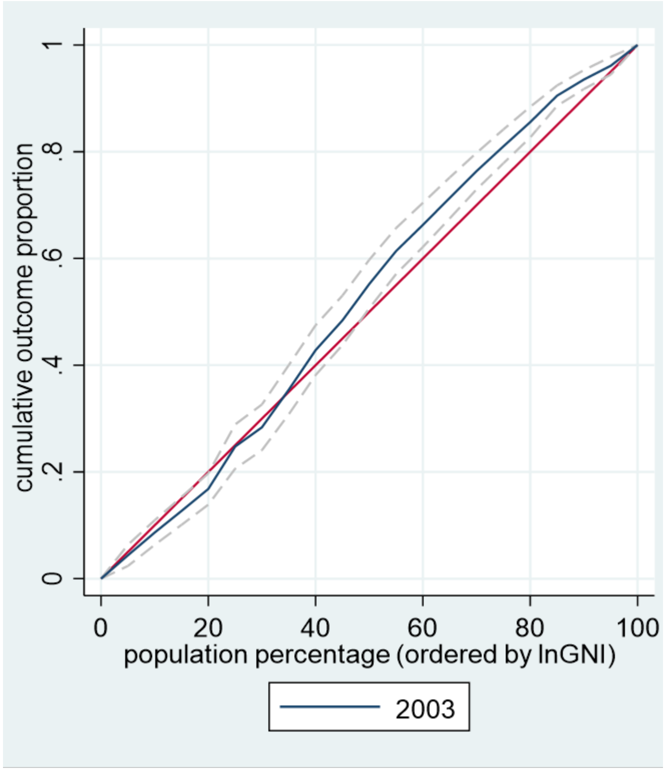

cumulative outcome proportion
0
.2
.4
.6
.8
1
0
20
40
60
80
100
population percentage (ordered by lnGNI)
2003

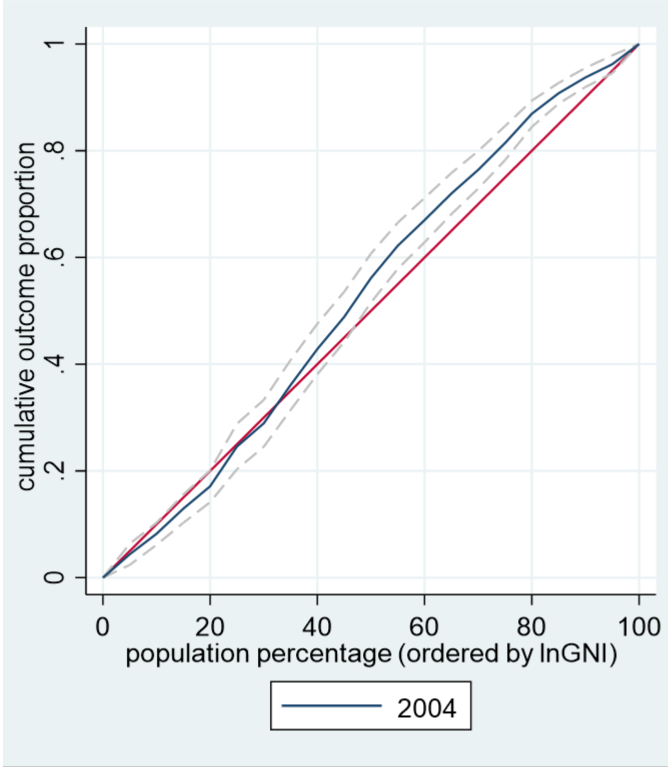

cumulative outcome proportion
0
.2
.4
.6
.8
1
0
20
40
60
80
100
population percentage (ordered by lnGNI)
2004

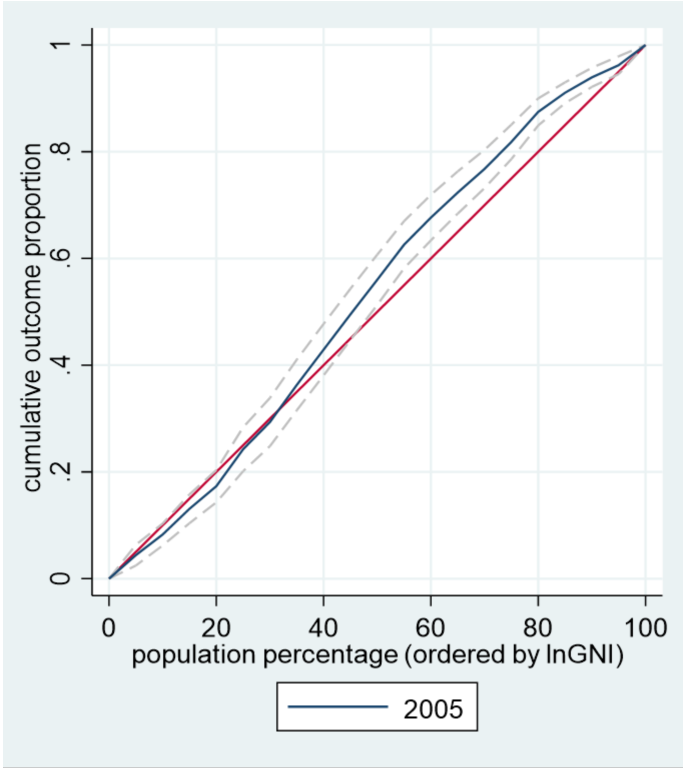
cumulative outcome proportion
0
.2
.4
.6
.8
1
0
20
40
60
80
100
population percentage (ordered by lnGNI)
2005

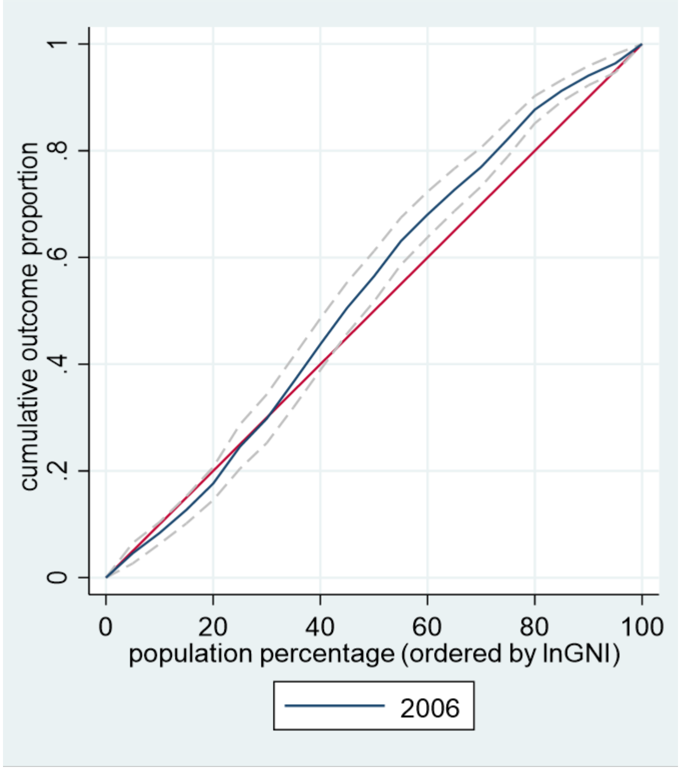
cumulative outcome proportion
0
.2
.4
.6
.8
1
0
20
40
60
80
100
population percentage (ordered by lnGNI)
2006

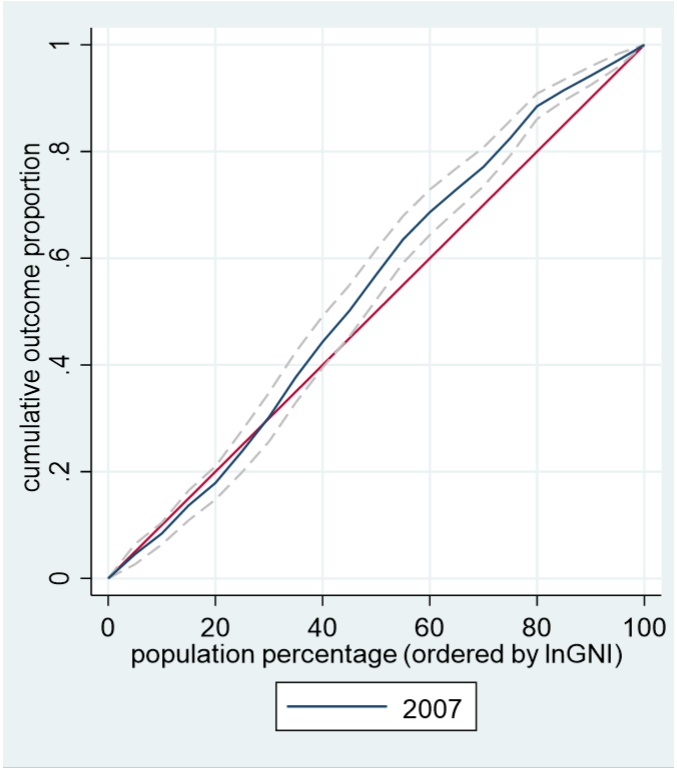

cumulative outcome proportion
0
.2
.4
.6
.8
1
0
20
40
60
80
100
population percentage (ordered by lnGNI)
2007

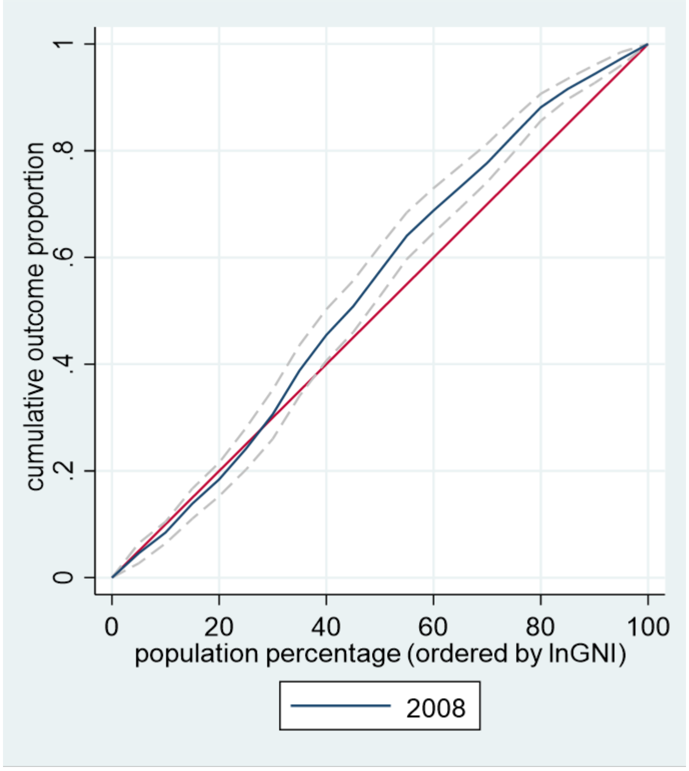

cumulative outcome proportion
0
.2
.4
.6
.8
1
0
20
40
60
80
100
population percentage (ordered by lnGNI)
2008

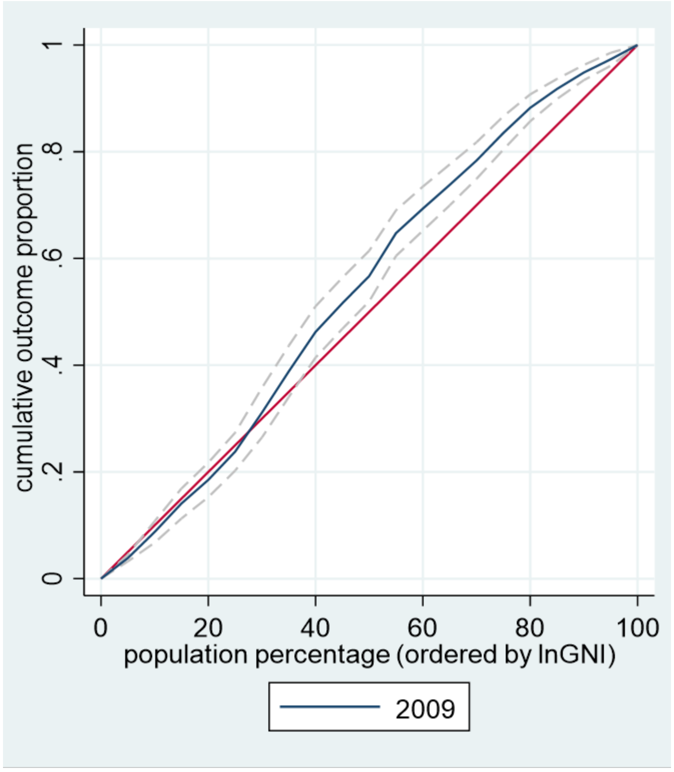
cumulative outcome proportion
0
.2
.4
.6
.8
1
0
20
40
60
80
100
population percentage (ordered by lnGNI)
2009

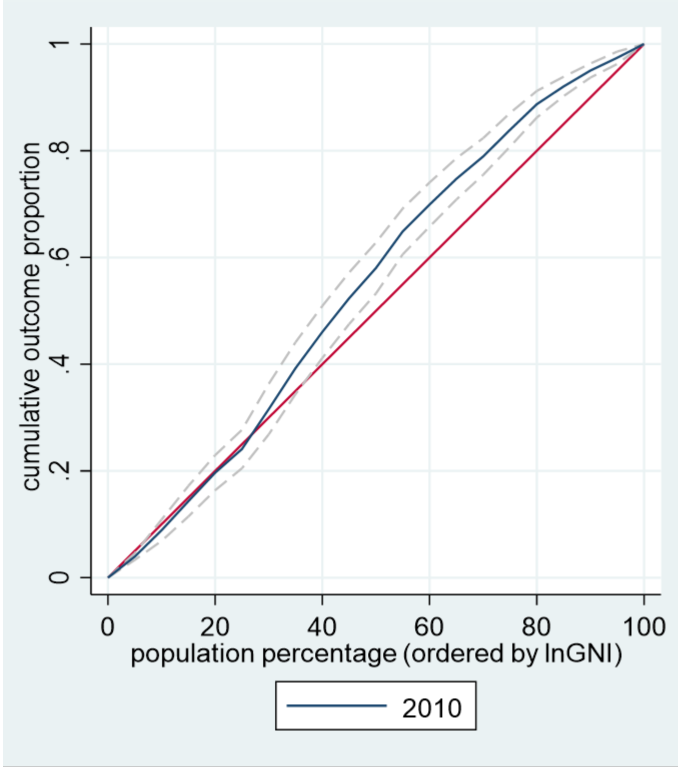
cumulative outcome proportion
0
.2
.4
.6
.8
1
0
20
40
60
80
100
population percentage (ordered by lnGNI)
2010

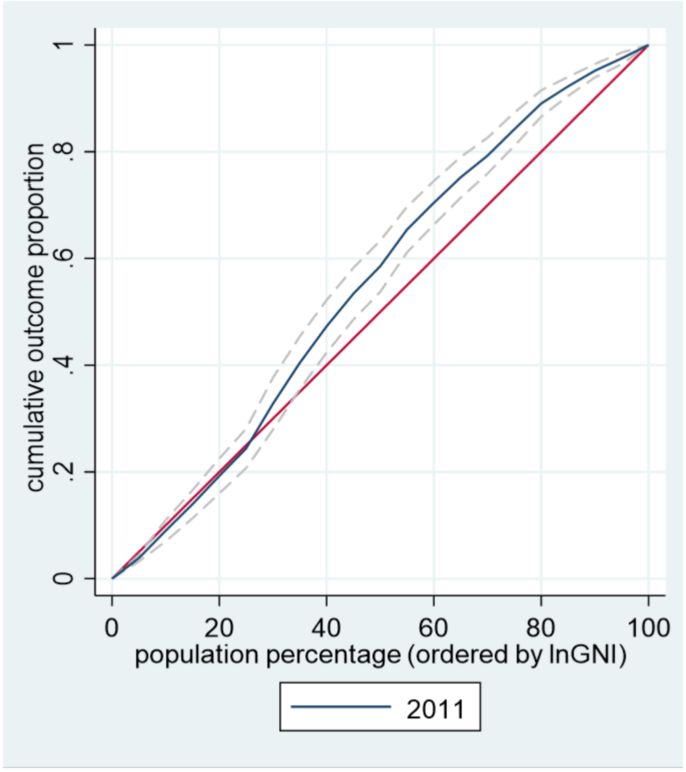

cumulative outcome proportion
0
.2
.4
.6
.8
1
0
20
40
60
80
100
population percentage (ordered by lnGNI)
2011

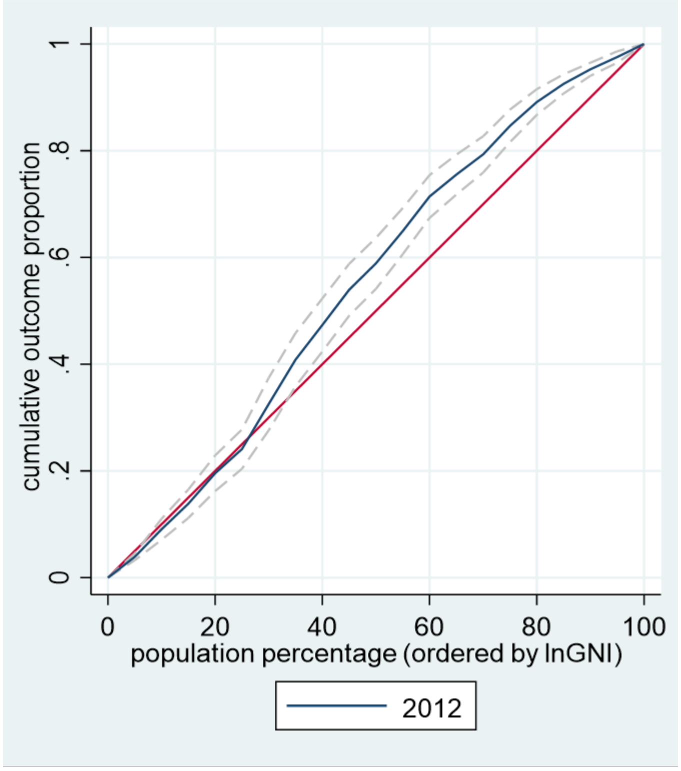

cumulative outcome proportion
0
.2
.4
.6
.8
1
0
20
40
60
80
100
population percentage (ordered by lnGNI)
2012

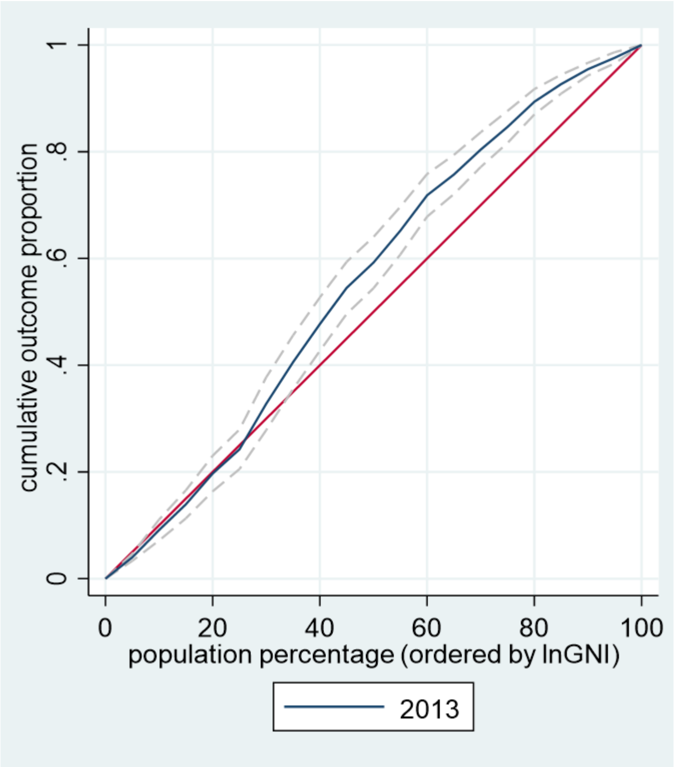
cumulative outcome proportion
0
.2
.4
.6
.8
1
0
20
40
60
80
100
population percentage (ordered by lnGNI)
2013

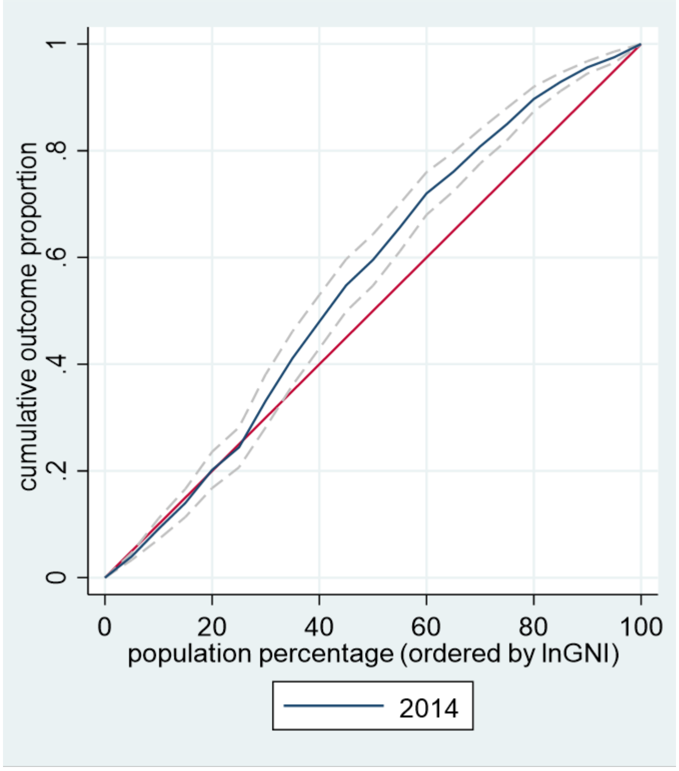
cumulative outcome proportion
0
.2
.4
.6
.8
1
0
20
40
60
80
100
population percentage (ordered by lnGNI)
2014

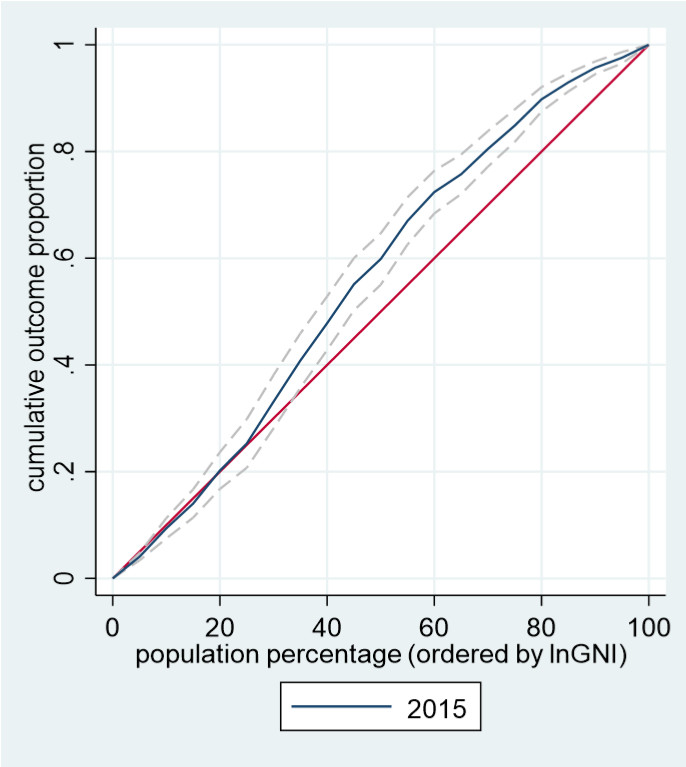
cumulative outcome proportion
0
.2
.4
.6
.8
1
0
20
40
60
80
100
population percentage (ordered by lnGNI)
2015

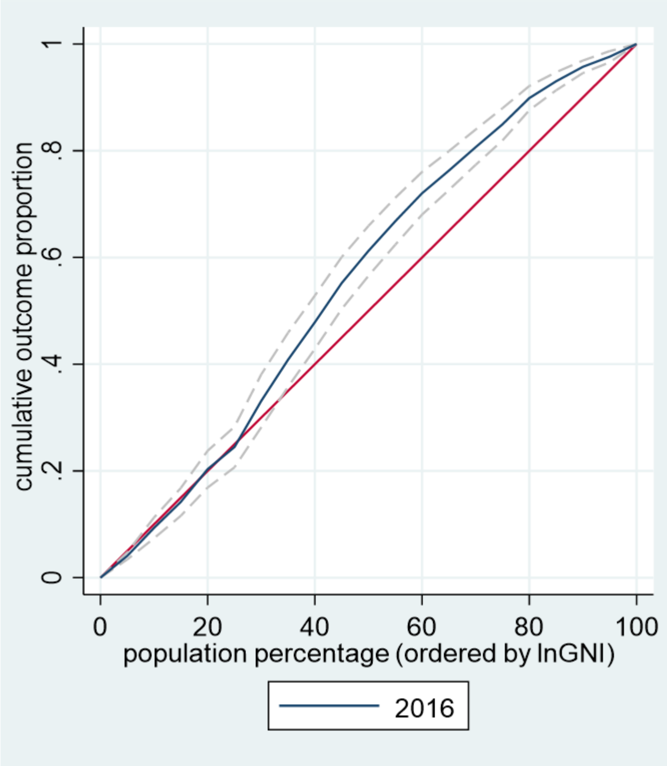
cumulative outcome proportion
0
.2
.4
.6
.8
1
0
20
40
60
80
100
population percentage (ordered by lnGNI)
2016

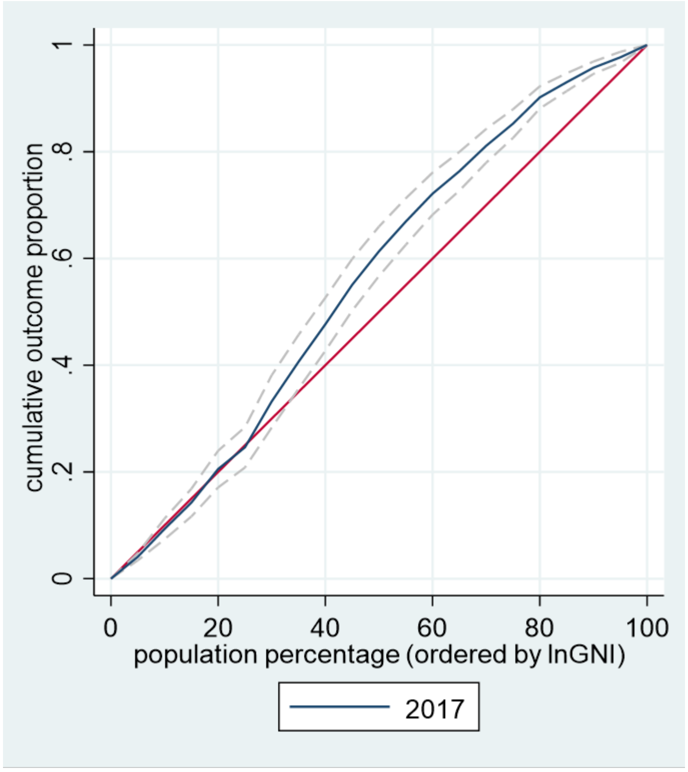
cumulative outcome proportion
0
.2
.4
.6
.8
1
0
20
40
60
80
100
population percentage (ordered by lnGNI)
2017

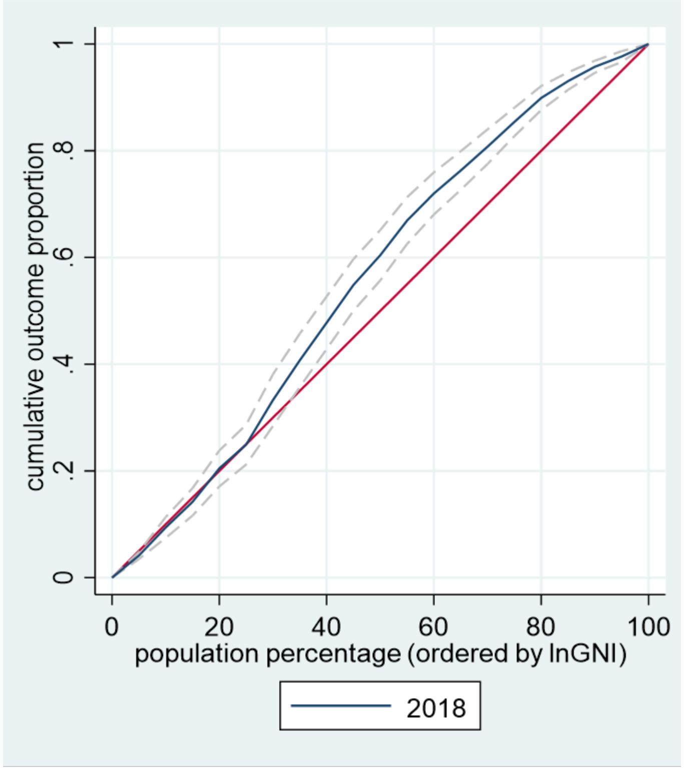
cumulative outcome proportion
0
.2
.4
.6
.8
1
0
20
40
60
80
100
population percentage (ordered by lnGNI)
2018

Figs. S66-83 Concentration curves of age-standardized DALY rates for stroke from 2001 to 2018

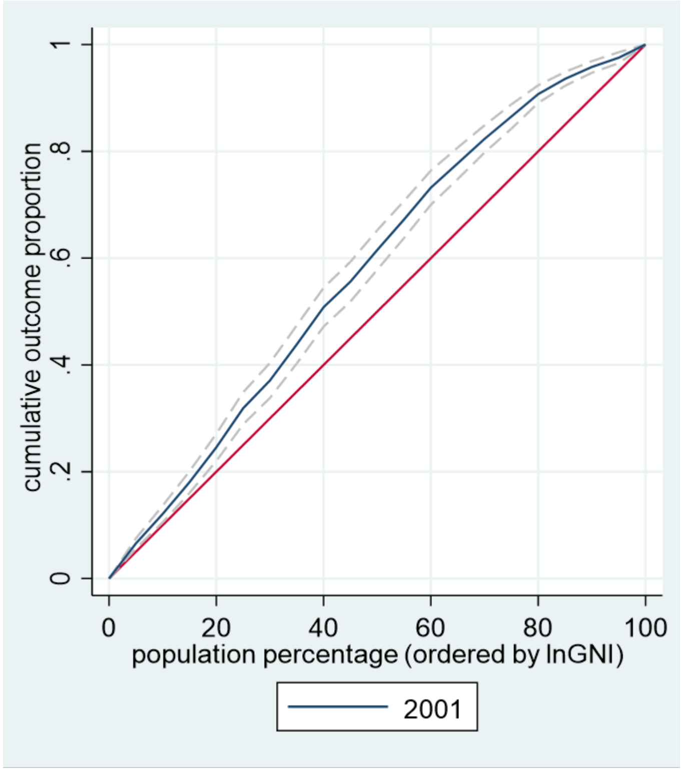

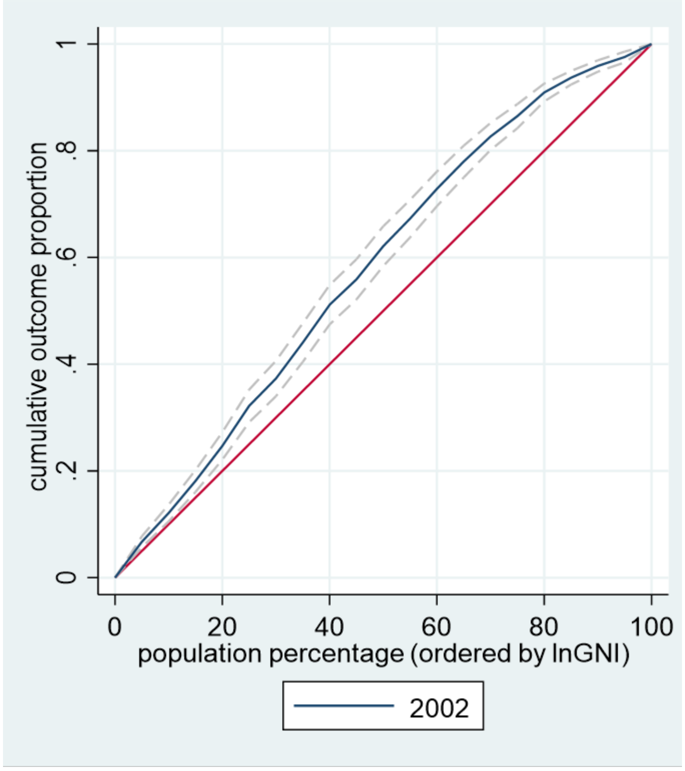

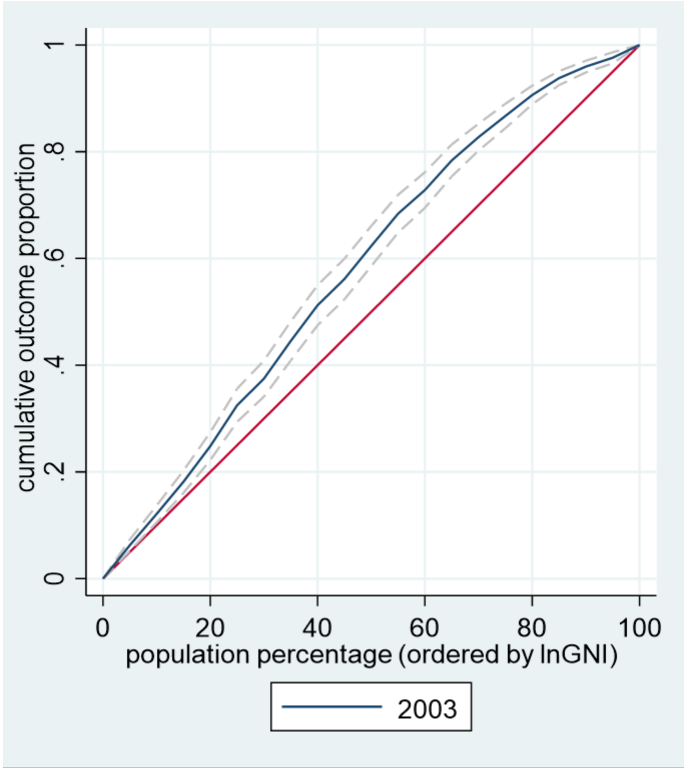

cumulative outcome proportion
0
.2
.4
.6
.8
1
0
20
40
60
80
100
population percentage (ordered by lnGNI)
2003

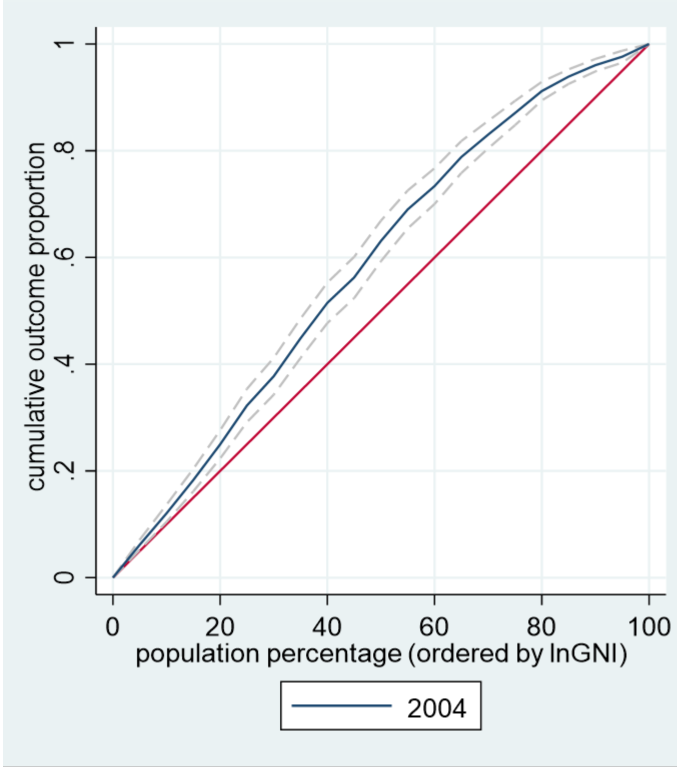

cumulative outcome proportion
0
.2
.4
.6
.8
1
0
20
40
60
80
100
population percentage (ordered by lnGNI)
2004

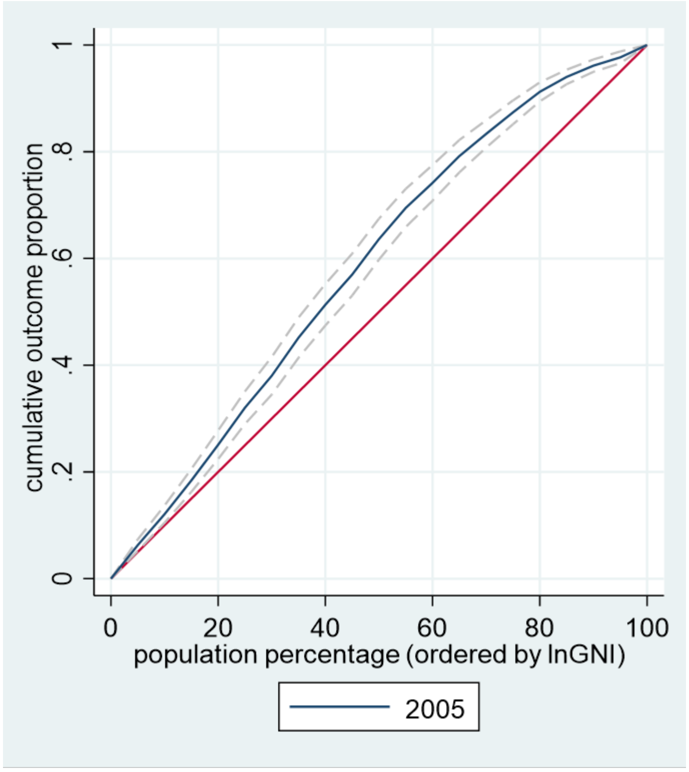
cumulative outcome proportion
0
.2
.4
.6
.8
1
0
20
40
60
80
100
population percentage (ordered by lnGNI)
2005

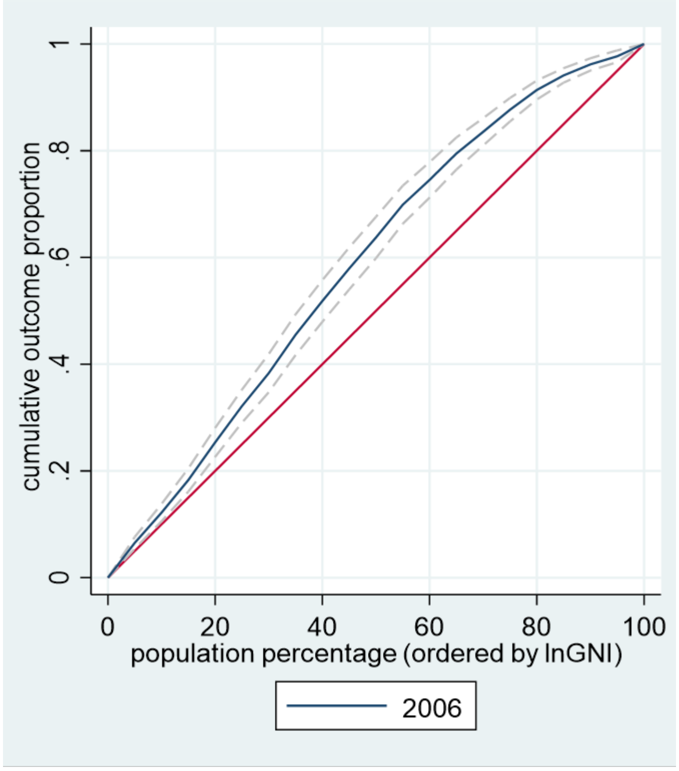
cumulative outcome proportion
0
.2
.4
.6
.8
1
0
20
40
60
80
100
population percentage (ordered by lnGNI)
2006

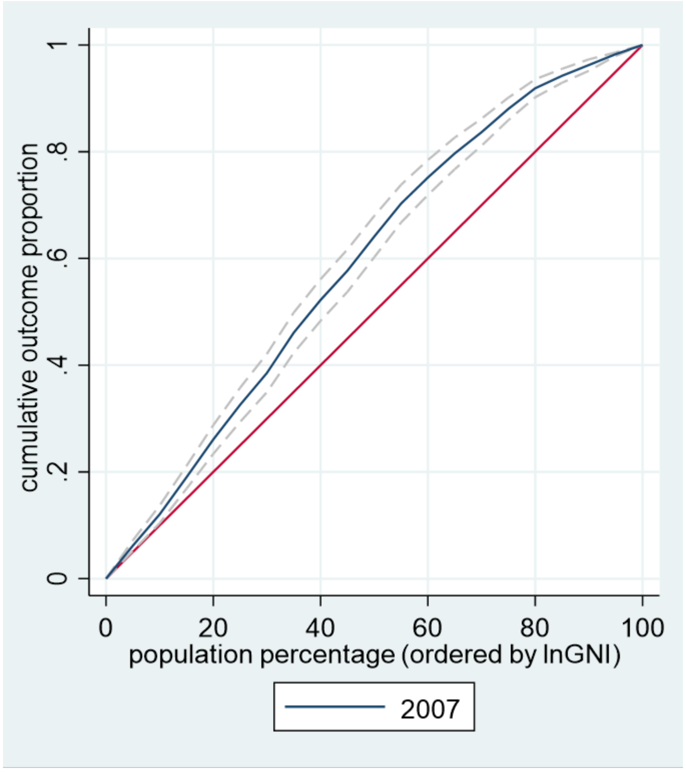
cumulative outcome proportion
0
.2
.4
.6
.8
1
0
20
40
60
80
100
population percentage (ordered by lnGNI)
2007

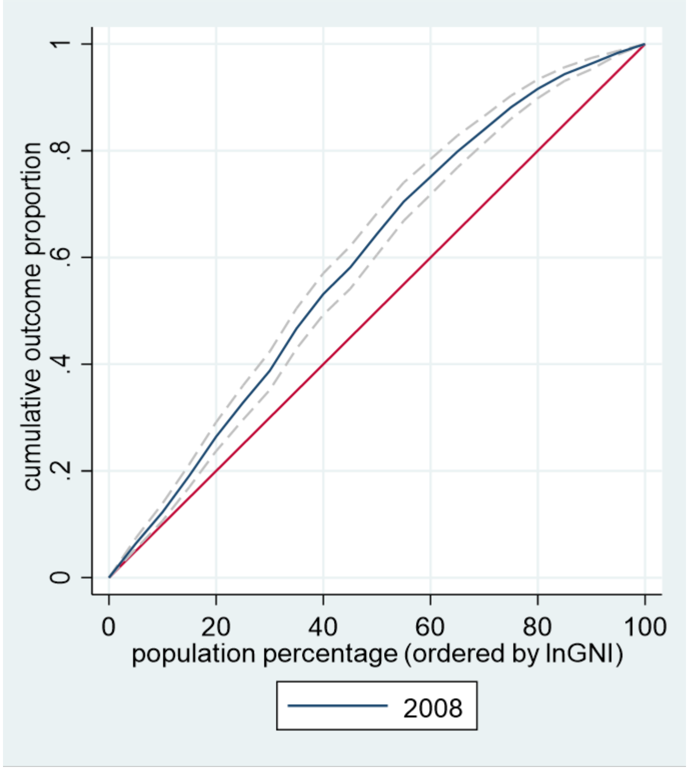
cumulative outcome proportion
0
.2
.4
.6
.8
1
0
20
40
60
80
100
population percentage (ordered by lnGNI)
2008

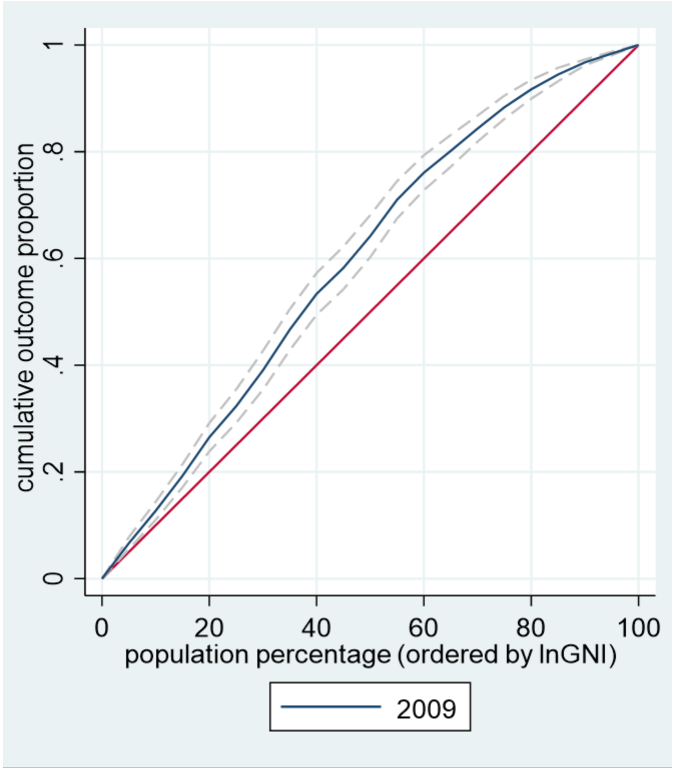

cumulative outcome proportion
0
.2
.4
.6
.8
1
0
20
40
60
80
100
population percentage (ordered by lnGNI)
2009

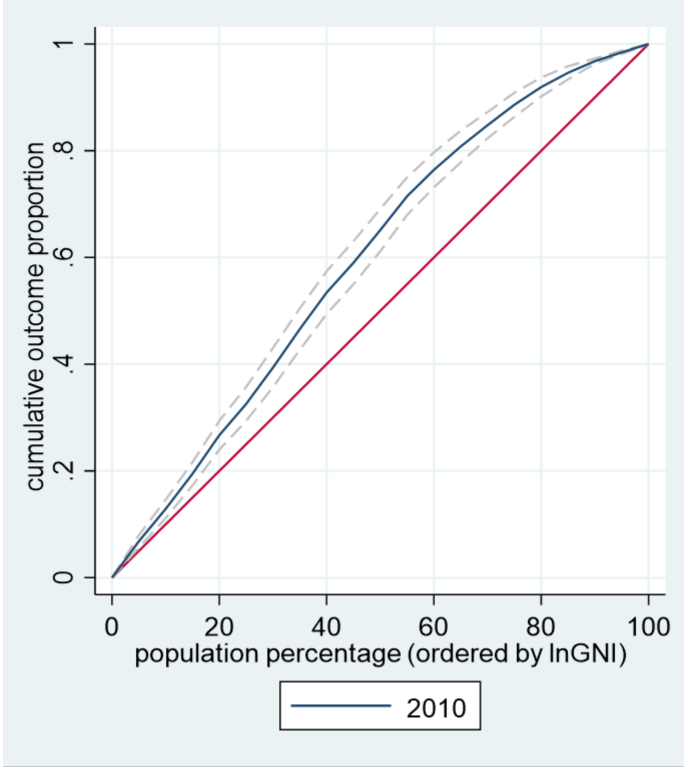

cumulative outcome proportion
0
.2
.4
.6
.8
1
0
20
40
60
80
100
population percentage (ordered by lnGNI)
2010

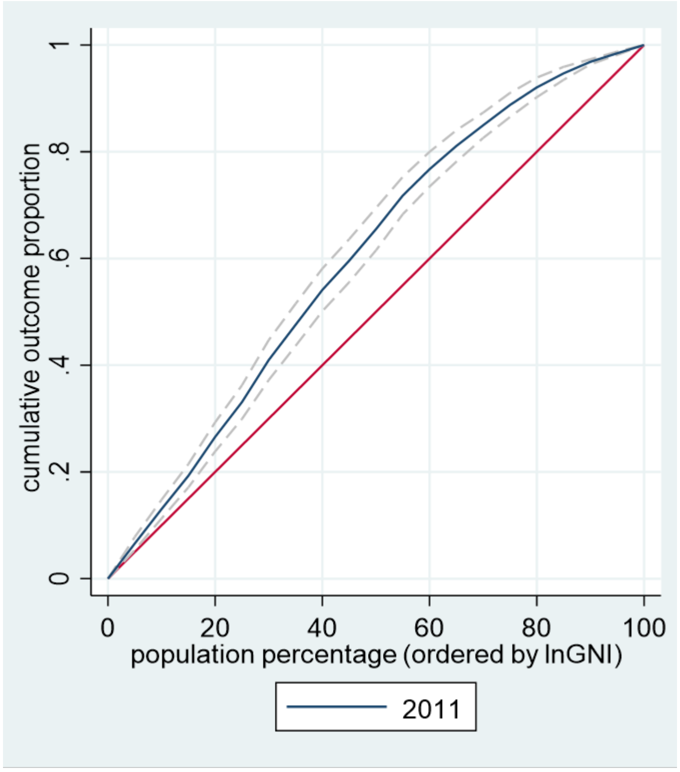
cumulative outcome proportion
0
.2
.4
.6
.8
1
0
20
40
60
80
100
population percentage (ordered by lnGNI)
2011

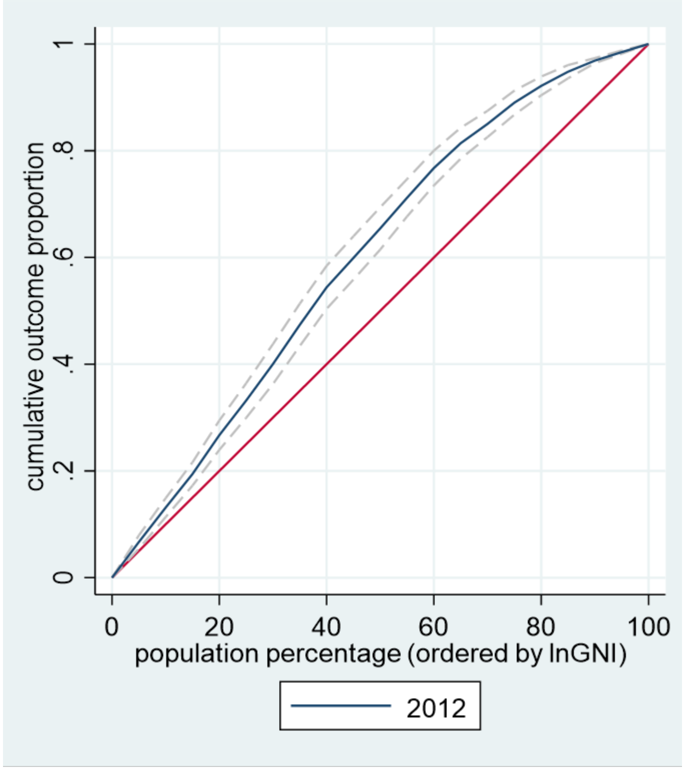
cumulative outcome proportion
0
.2
.4
.6
.8
1
0
20
40
60
80
100
population percentage (ordered by lnGNI)
2012

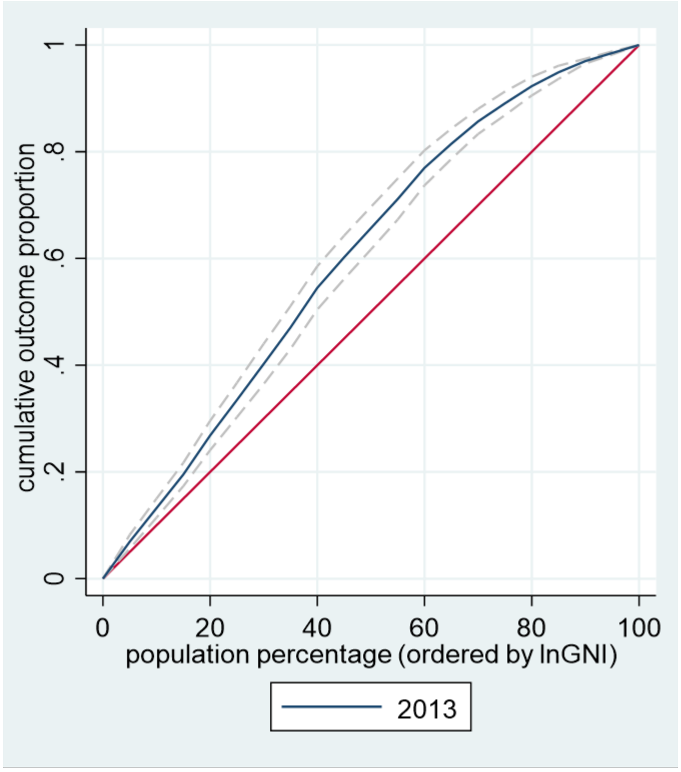
cumulative outcome proportion
0
.2
.4
.6
.8
1
0
20
40
60
80
100
population percentage (ordered by lnGNI)
2013

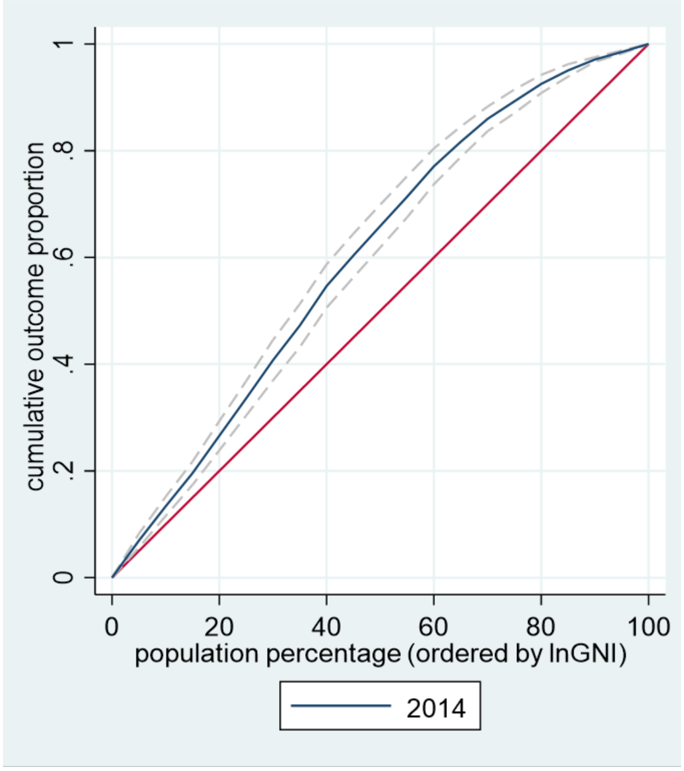
cumulative outcome proportion
0
.2
.4
.6
.8
1
0
20
40
60
80
100
population percentage (ordered by lnGNI)
2014

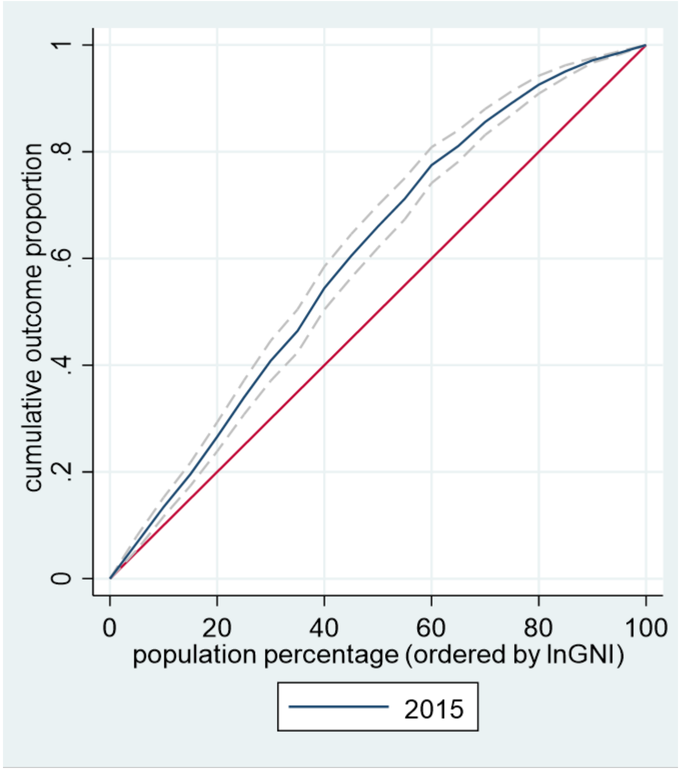

cumulative outcome proportion
0
.2
.4
.6
.8
1
0
20
40
60
80
100
population percentage (ordered by lnGNI)
2015

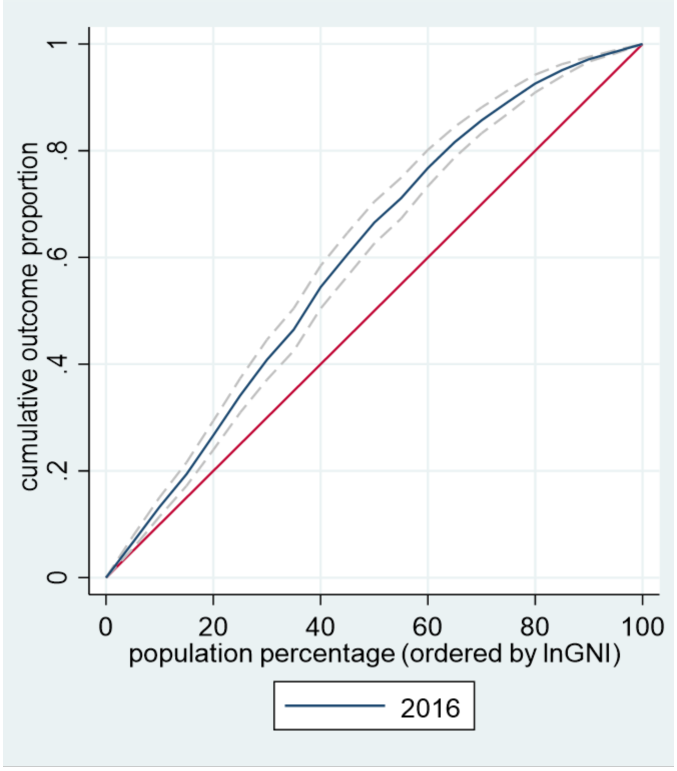

cumulative outcome proportion
0
.2
.4
.6
.8
1
0
20
40
60
80
100
population percentage (ordered by lnGNI)
2016

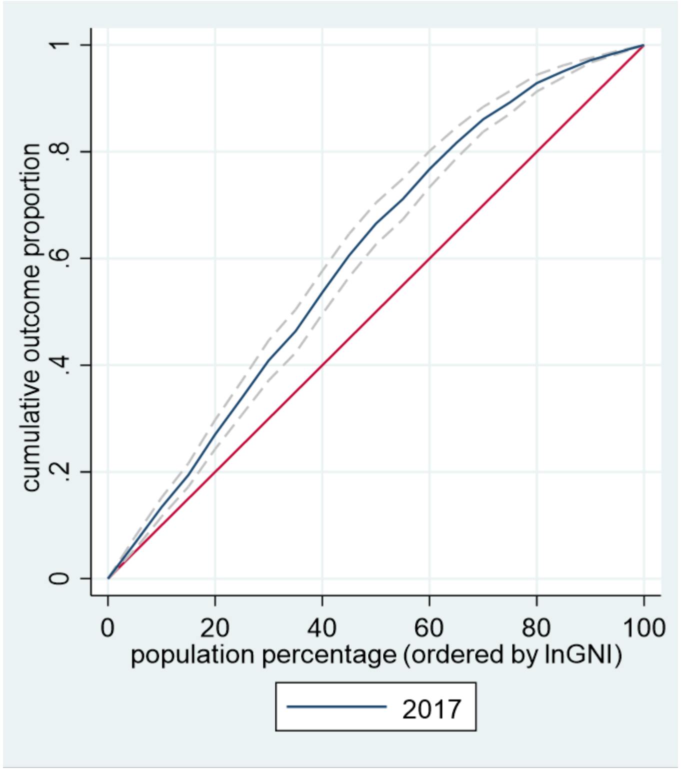
cumulative outcome proportion
0
.2
.4
.6
.8
1
0
20
40
60
80
100
population percentage (ordered by lnGNI)
2017

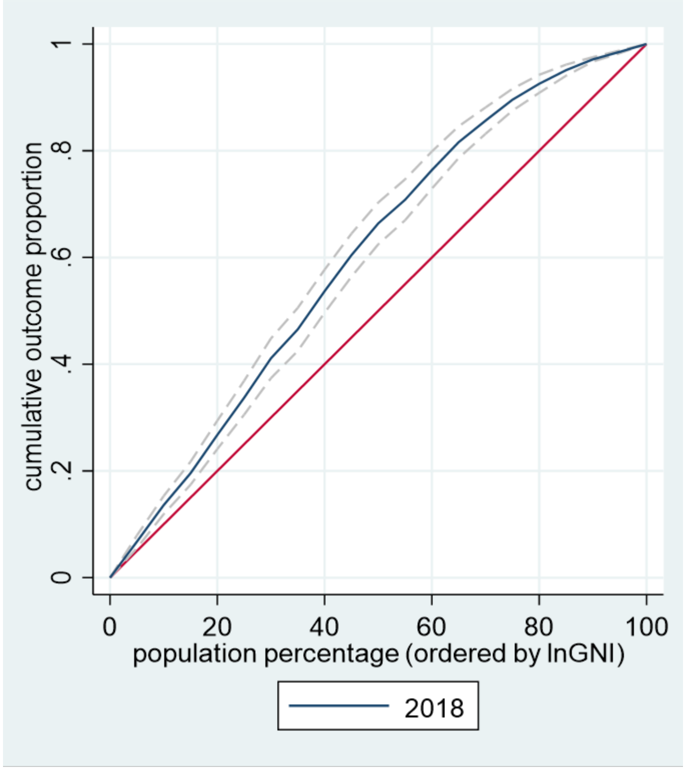
cumulative outcome proportion
0
.2
.4
.6
.8
1
0
20
40
60
80
100
population percentage (ordered by lnGNI)
2018

Figs. S84-101 Concentration curves of age-standardized DALY rates for hypertensive heart disease from 2001 to 2018

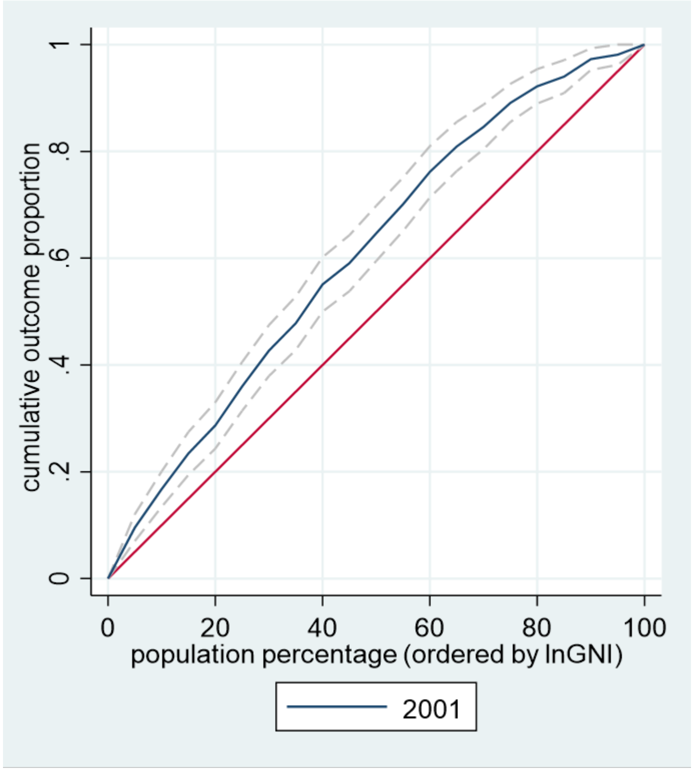

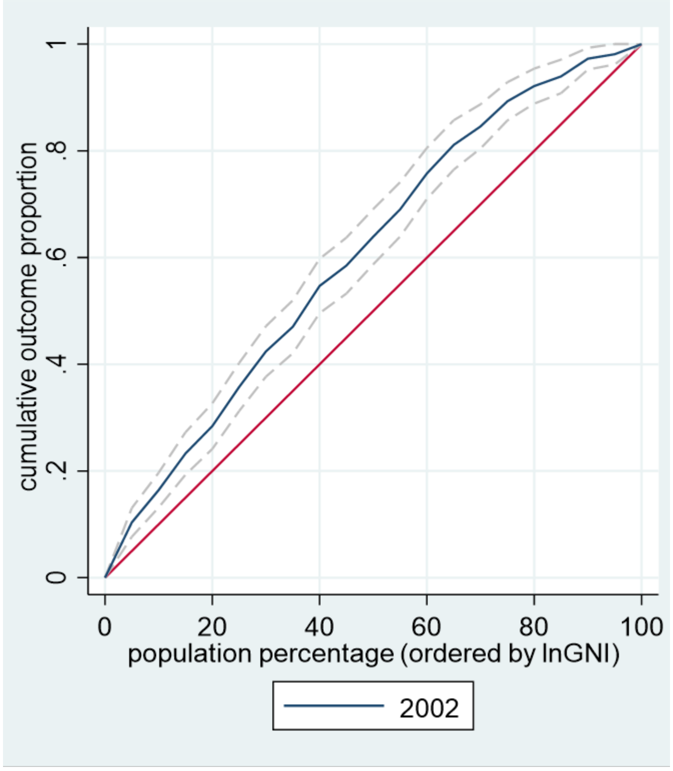

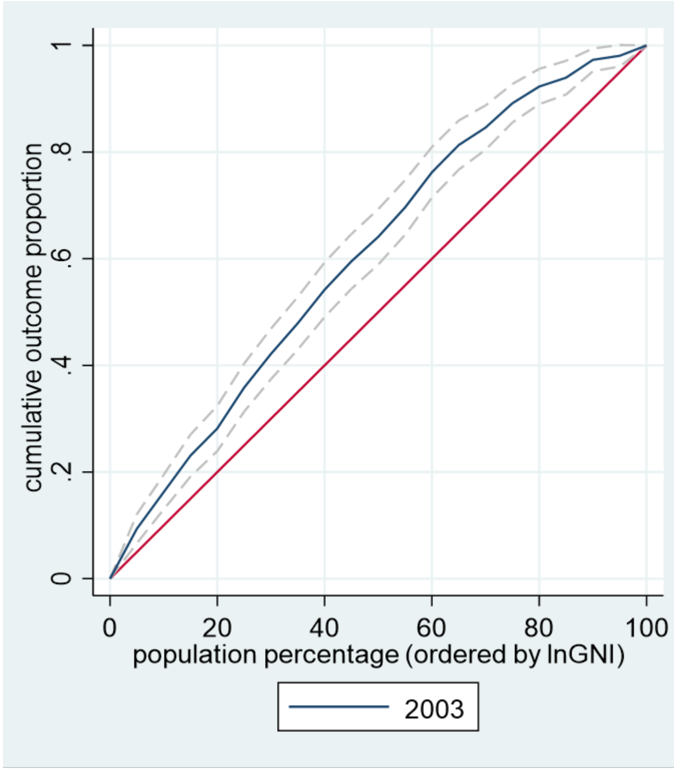
cumulative outcome proportion
0
.2
.4
.6
.8
1
0
20
40
60
80
100
population percentage (ordered by lnGNI)
2003

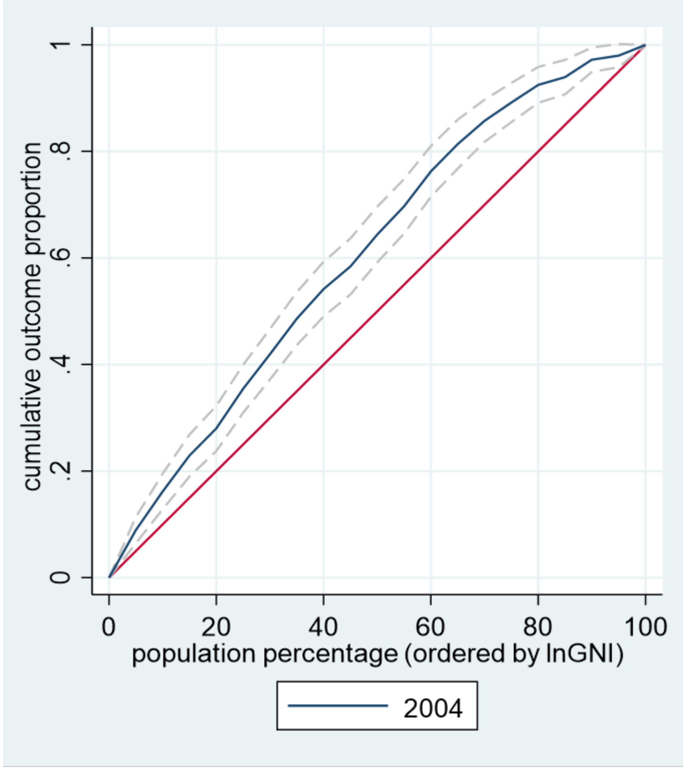
cumulative outcome proportion
0
.2
.4
.6
.8
1
0
20
40
60
80
100
population percentage (ordered by lnGNI)
2004

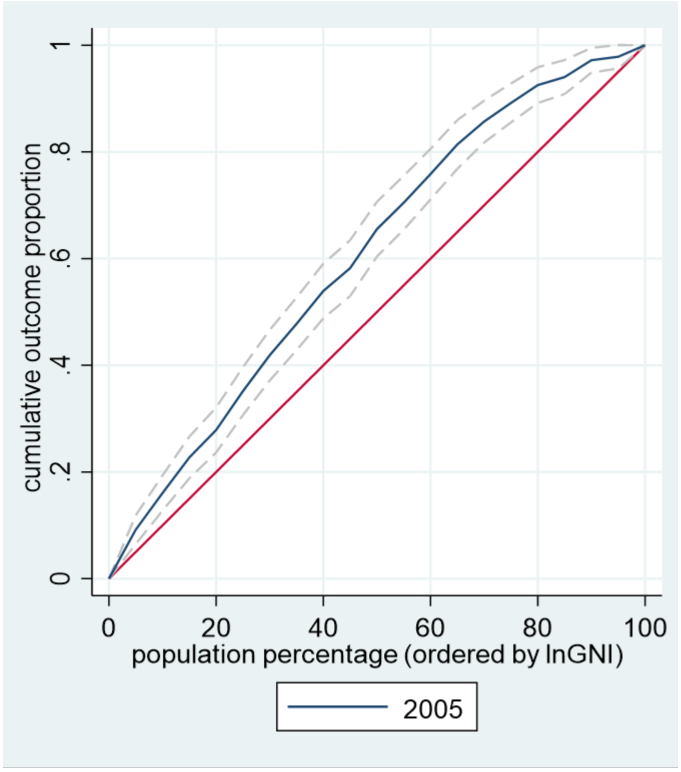

cumulative outcome proportion
0
.2
.4
.6
.8
1
0
20
40
60
80
100
population percentage (ordered by lnGNI)
2005

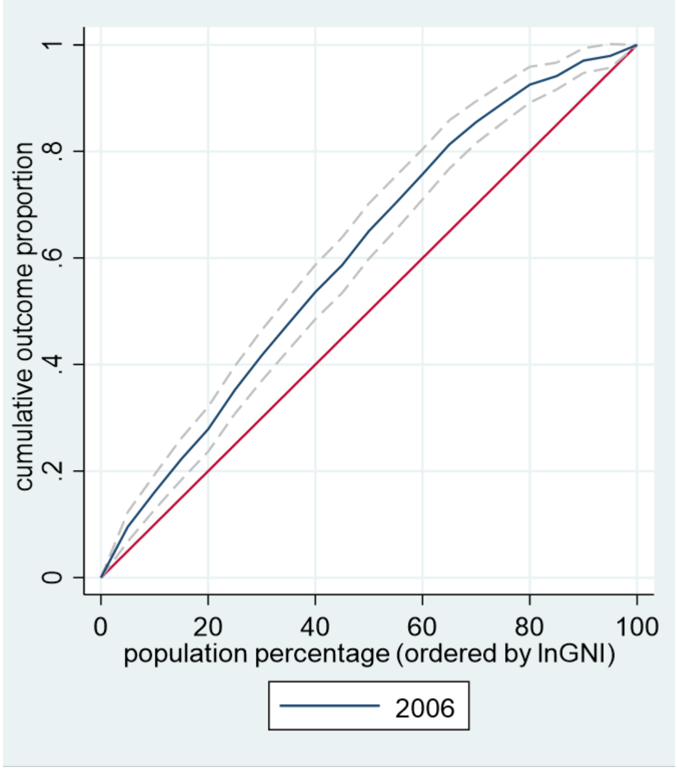

cumulative outcome proportion
0
.2
.4
.6
.8
1
0
20
40
60
80
100
population percentage (ordered by lnGNI)
2006

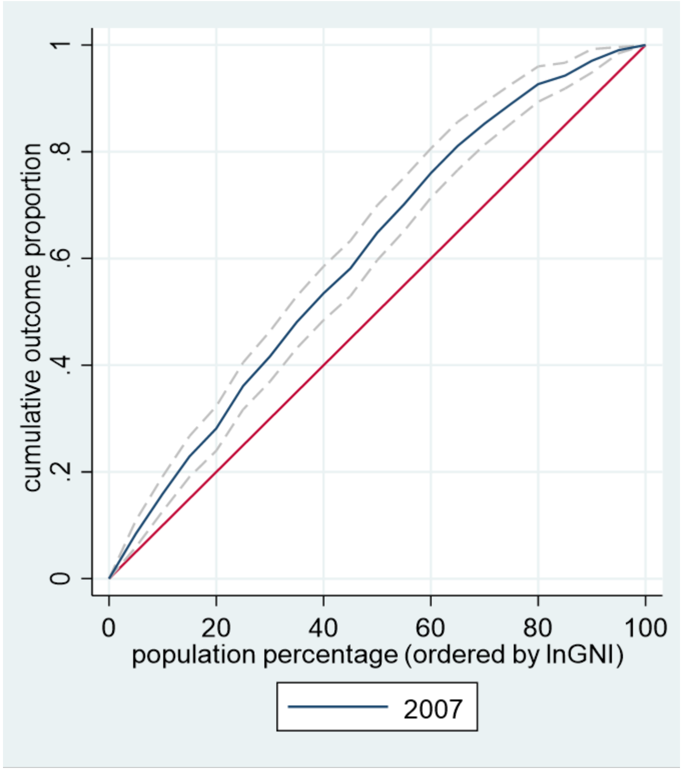

cumulative outcome proportion
0
.2
.4
.6
.8
1
0
20
40
60
80
100
population percentage (ordered by lnGNI)
2007

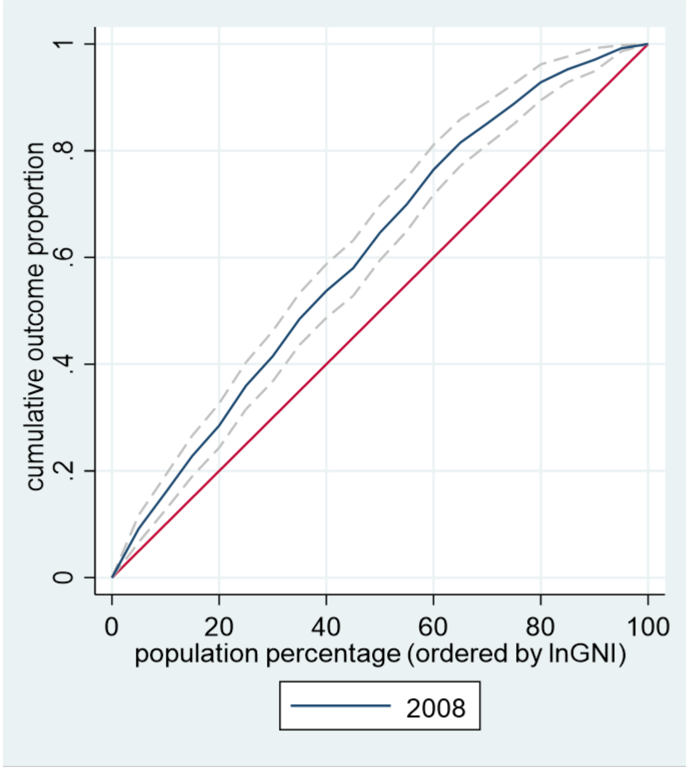

cumulative outcome proportion
0
.2
.4
.6
.8
1
0
20
40
60
80
100
population percentage (ordered by lnGNI)
2008

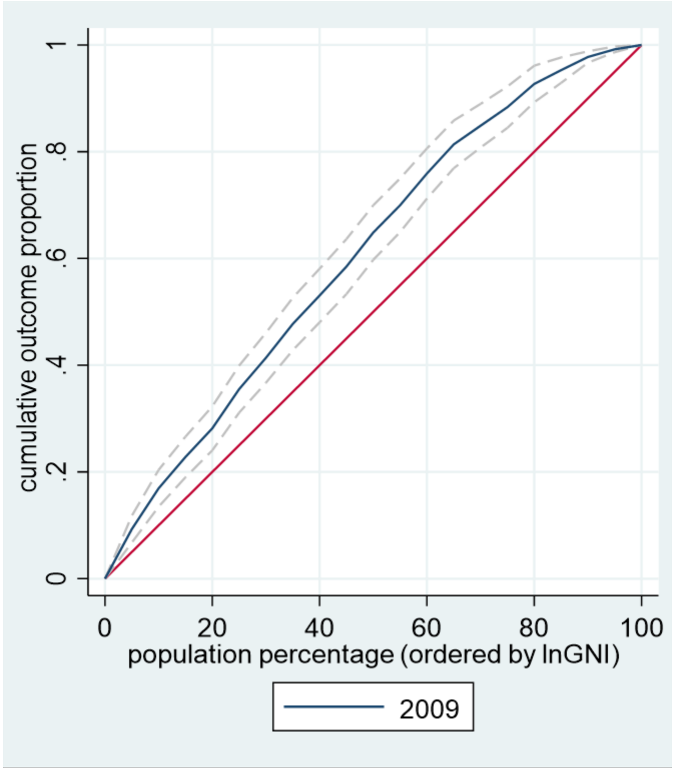
cumulative outcome proportion
0
.2
.4
.6
.8
1
0
20
40
60
80
100
population percentage (ordered by lnGNI)
2009

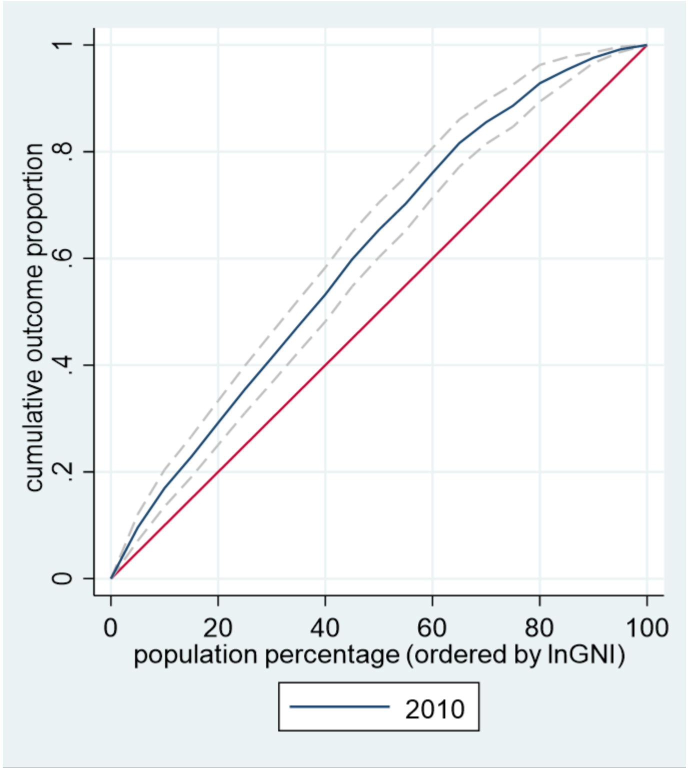
cumulative outcome proportion
0
.2
.4
.6
.8
1
0
20
40
60
80
100
population percentage (ordered by lnGNI)
2010

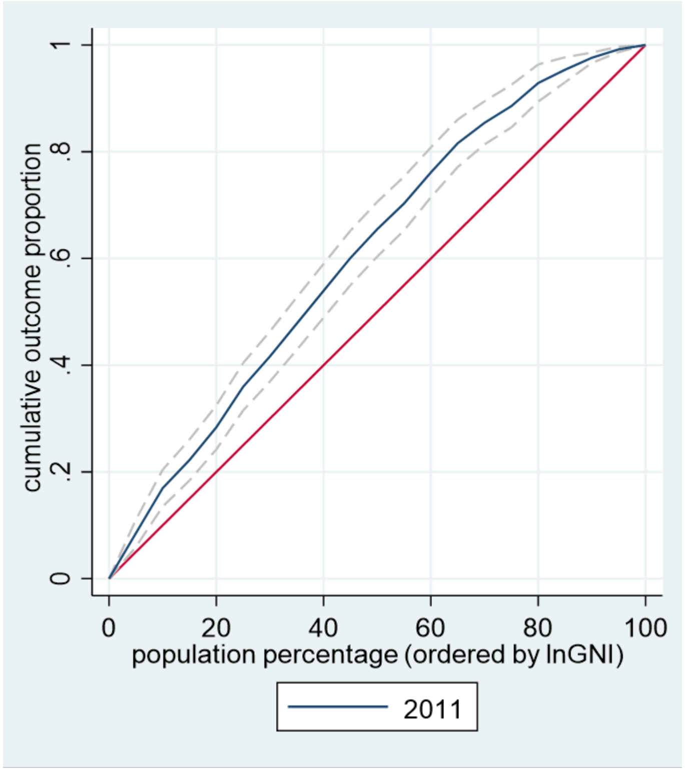
cumulative outcome proportion
0
.2
.4
.6
.8
1
0
20
40
60
80
100
population percentage (ordered by lnGNI)
2011

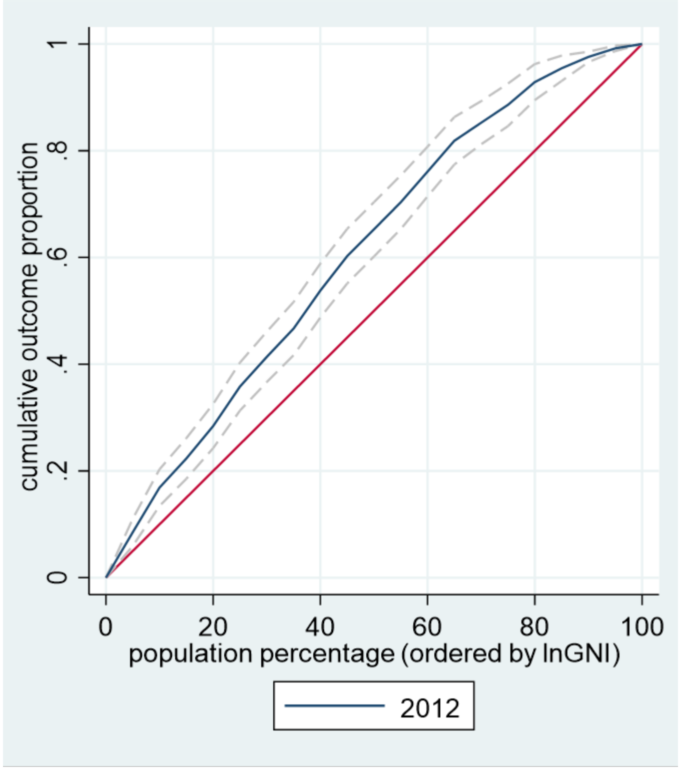
cumulative outcome proportion
0
.2
.4
.6
.8
1
0
20
40
60
80
100
population percentage (ordered by lnGNI)
2012

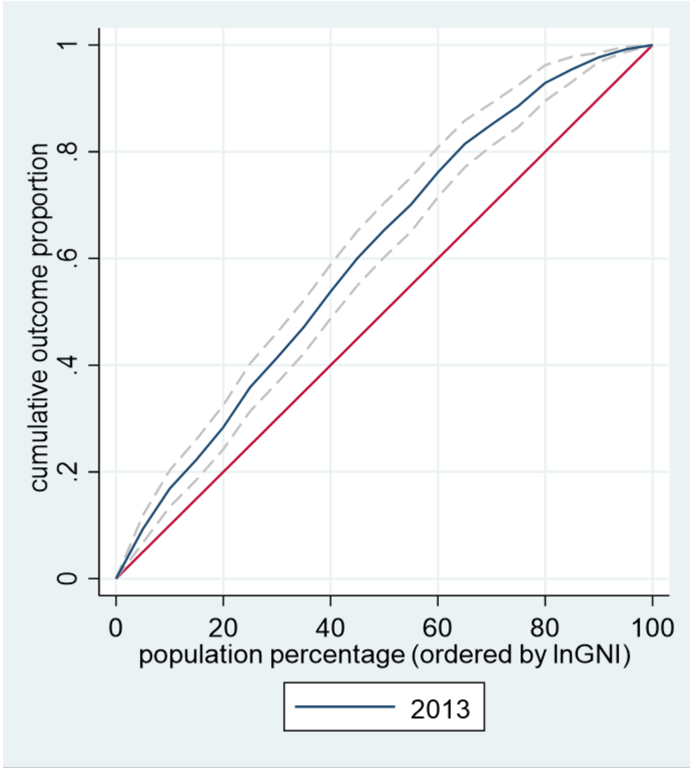

cumulative outcome proportion
0
.2
.4
.6
.8
1
0
20
40
60
80
100
population percentage (ordered by lnGNI)
2013

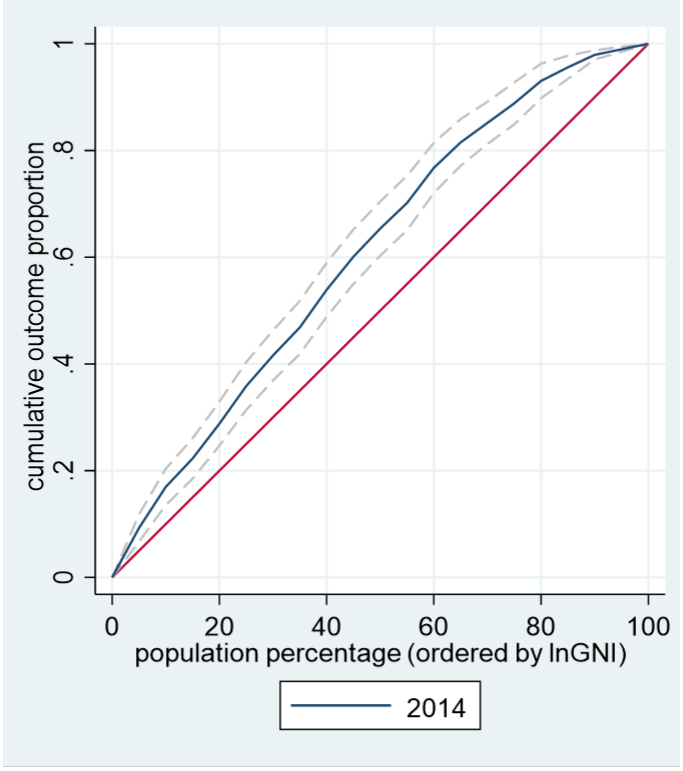

cumulative outcome proportion
0
.2
.4
.6
.8
1
0
20
40
60
80
100
population percentage (ordered by lnGNI)
2014

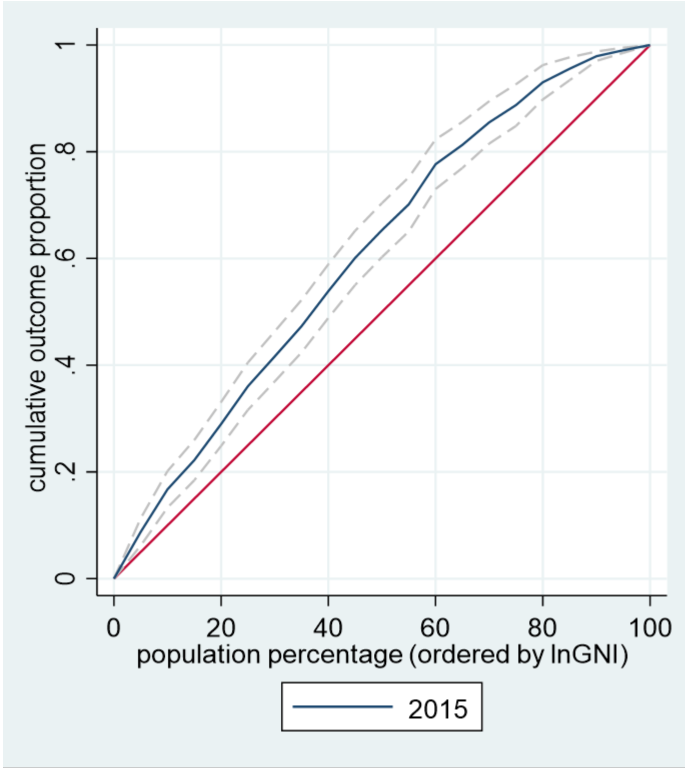

cumulative outcome proportion
0
.2
.4
.6
.8
1
0
20
40
60
80
100
population percentage (ordered by lnGNI)
2015

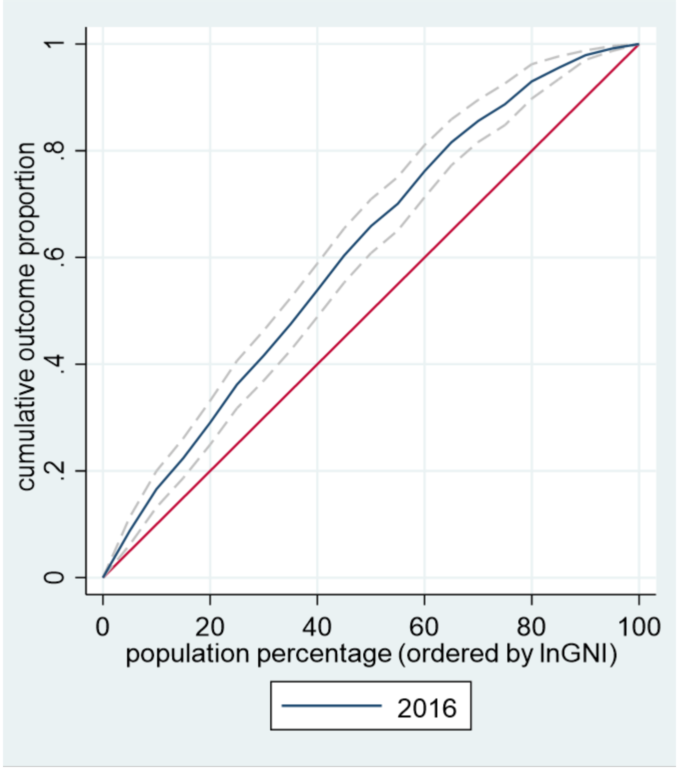

cumulative outcome proportion
0
.2
.4
.6
.8
1
0
20
40
60
80
100
population percentage (ordered by lnGNI)
2016

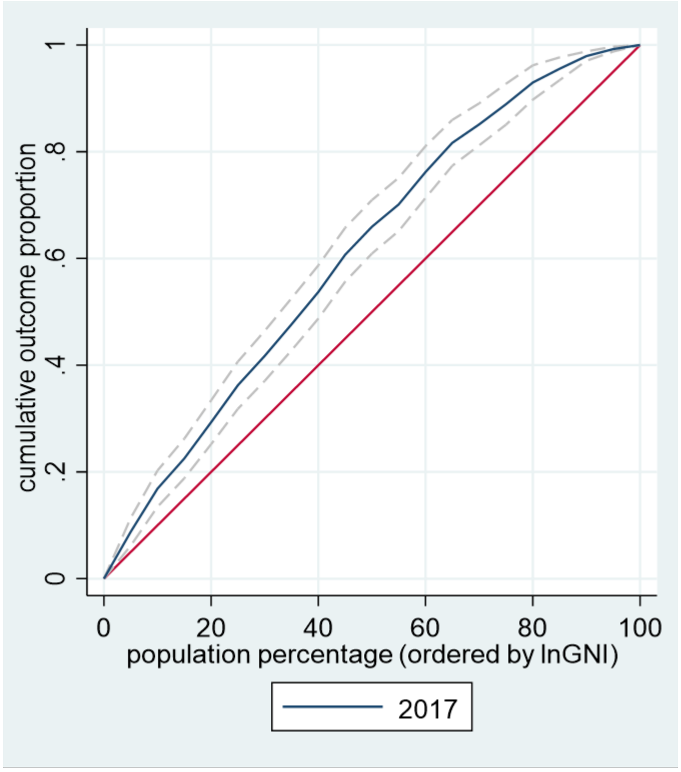

cumulative outcome proportion
0
.2
.4
.6
.8
1
0
20
40
60
80
100
population percentage (ordered by lnGNI)
2017

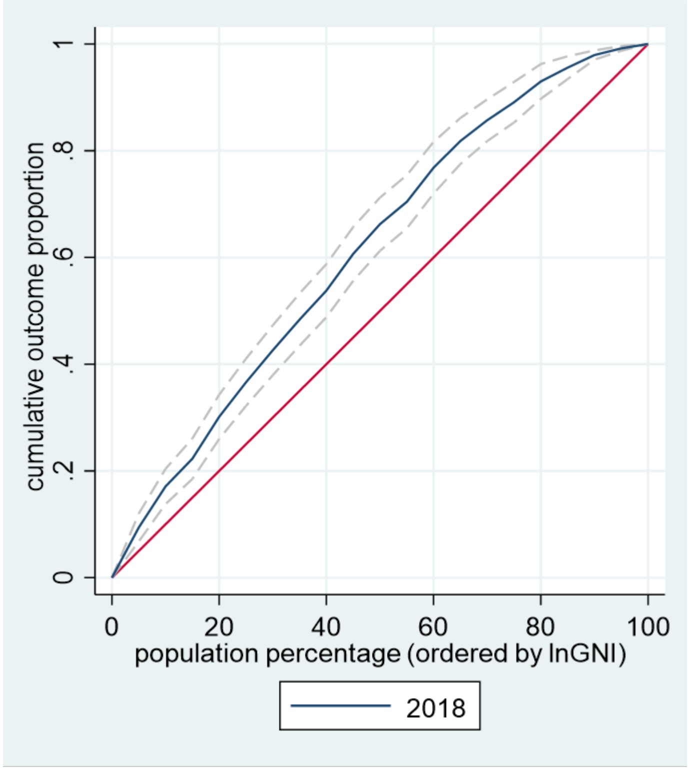

cumulative outcome proportion
0
.2
.4
.6
.8
1
0
20
40
60
80
100
population percentage (ordered by lnGNI)
2018

Figs. S102-119 Concentration curves of age-standardized DALY rates for non-rheumatic valvular heart disease from 2001 to 2018

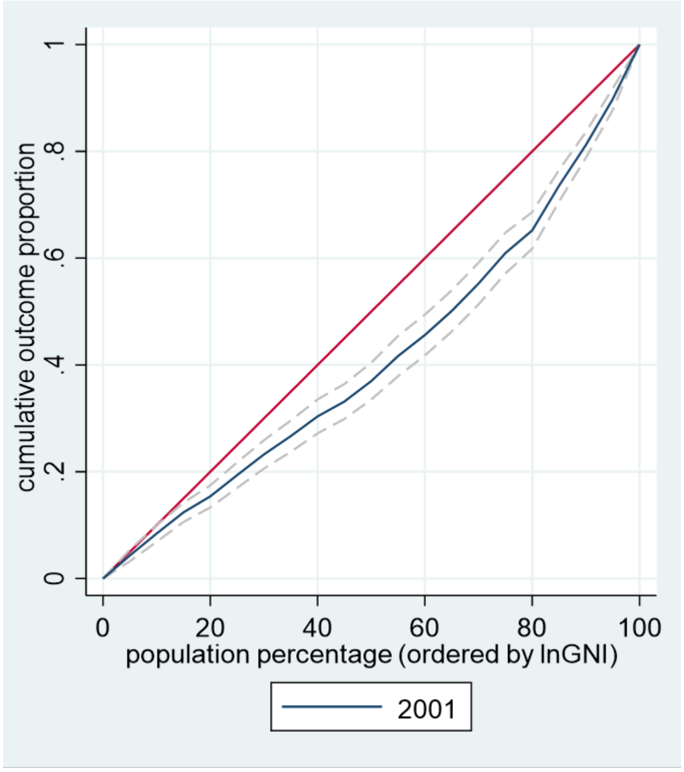

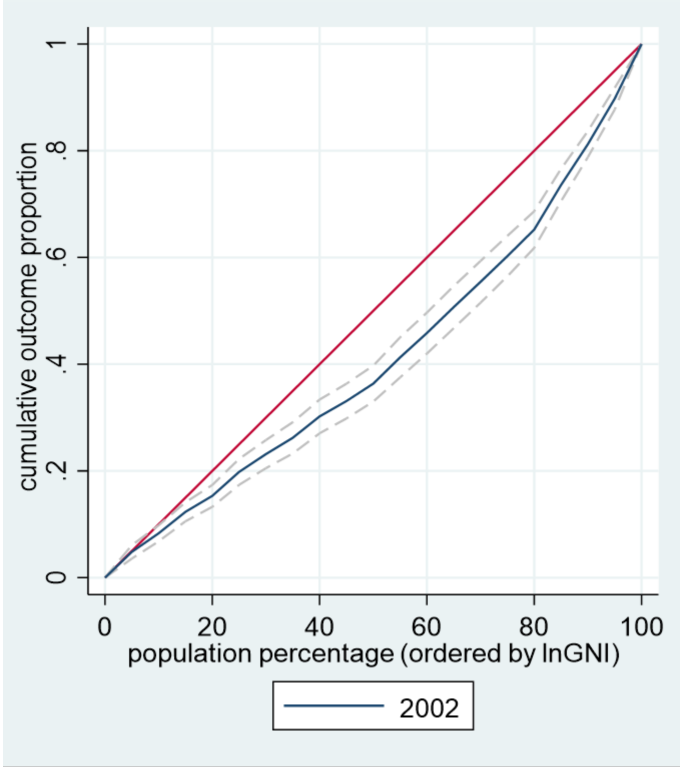

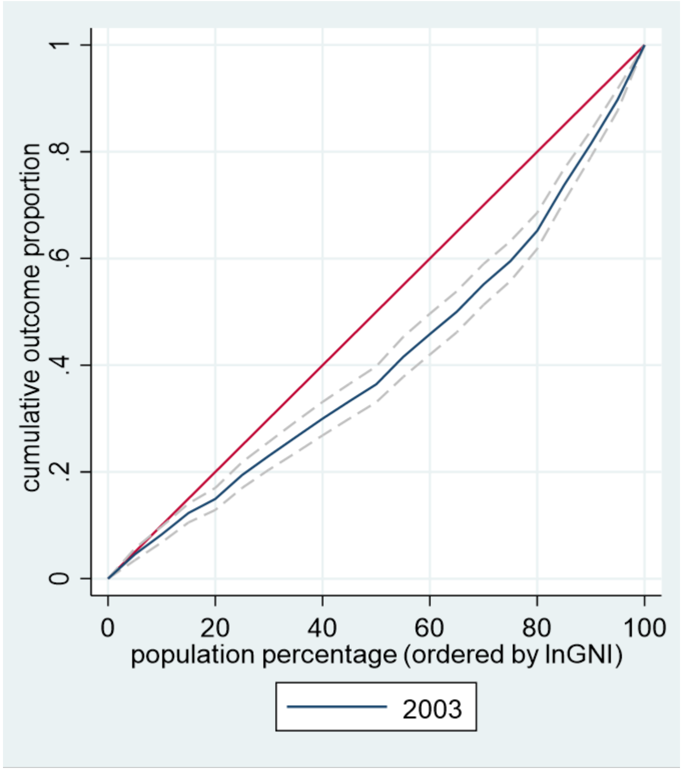
cumulative outcome proportion
0
.2
.4
.6
.8
1
0
20
40
60
80
100
population percentage (ordered by lnGNI)
2003

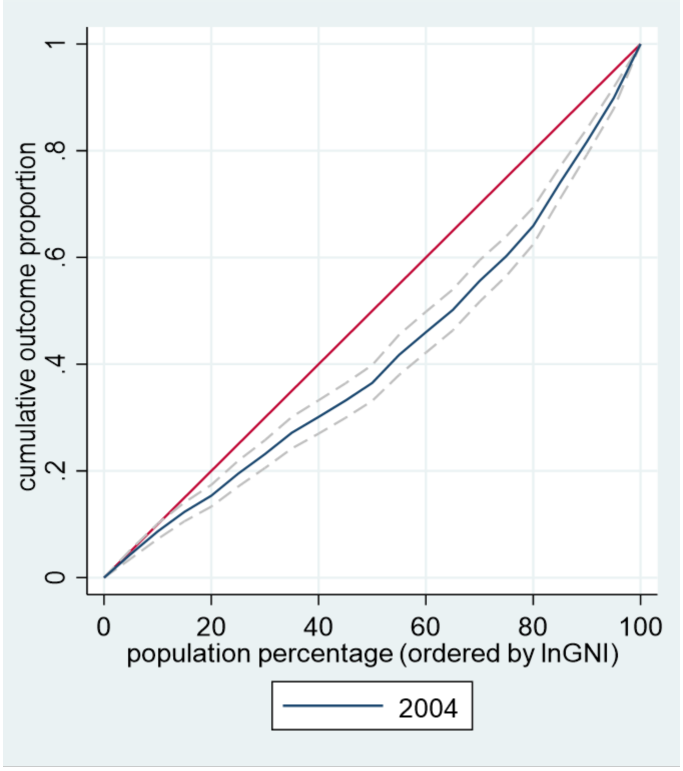
cumulative outcome proportion
0
.2
.4
.6
.8
1
0
20
40
60
80
100
population percentage (ordered by lnGNI)
2004

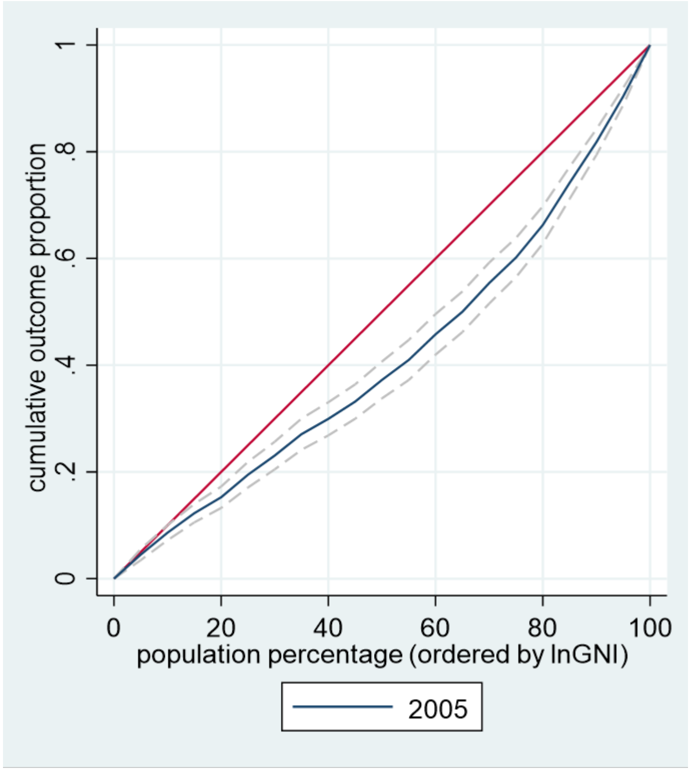

cumulative outcome proportion
0
.2
.4
.6
.8
1
0
20
40
60
80
100
population percentage (ordered by lnGNI)
2005

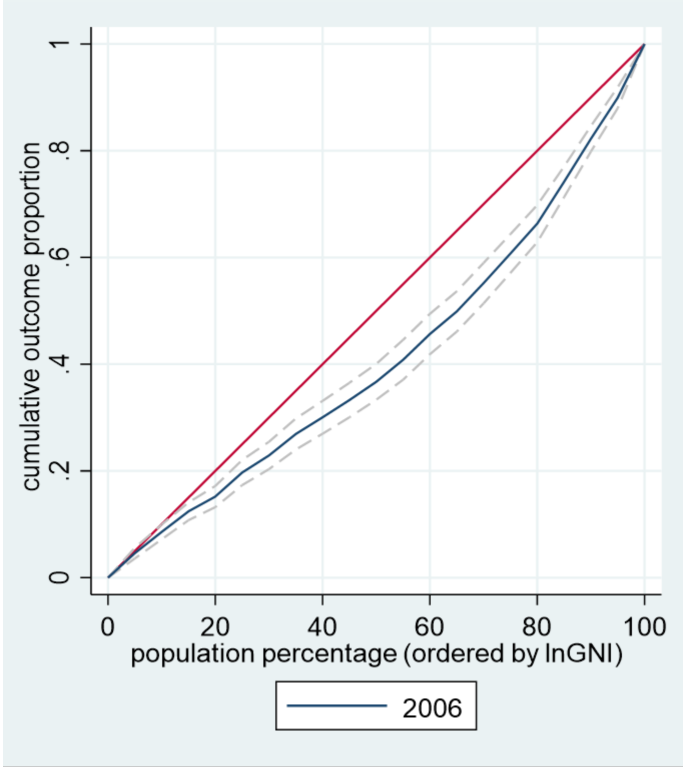

cumulative outcome proportion
0
.2
.4
.6
.8
1
0
20
40
60
80
100
population percentage (ordered by lnGNI)
2006

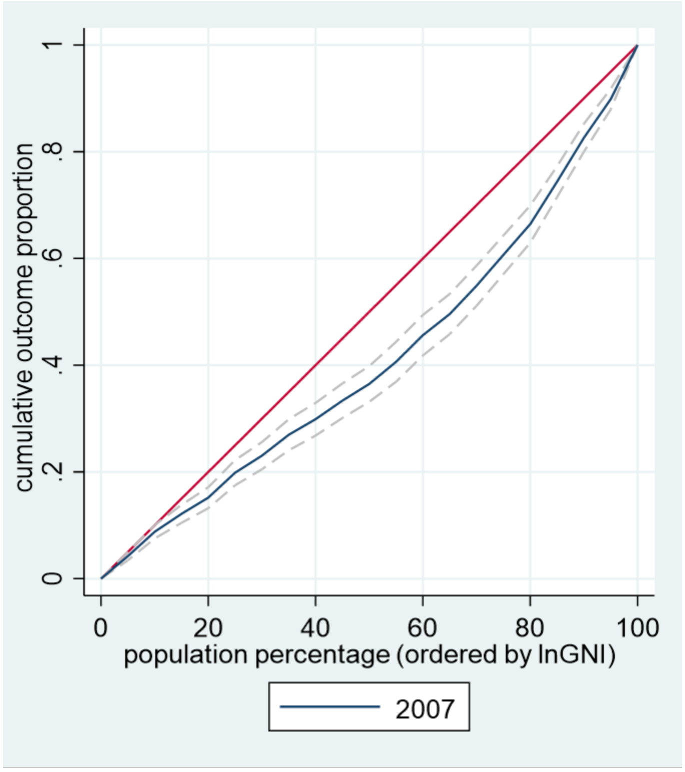

cumulative outcome proportion
0
.2
.4
.6
.8
1
0
20
40
60
80
100
population percentage (ordered by lnGNI)
2007

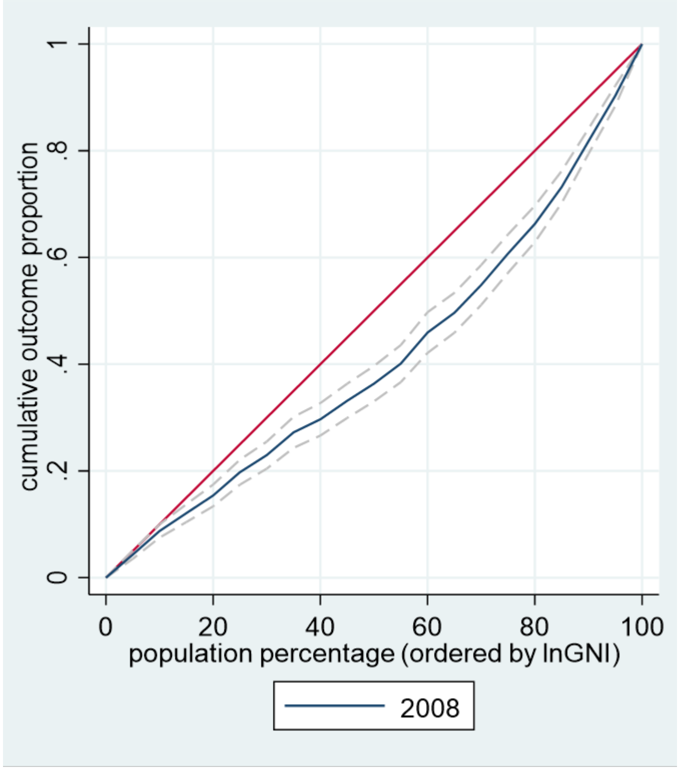

cumulative outcome proportion
0
.2
.4
.6
.8
1
0
20
40
60
80
100
population percentage (ordered by lnGNI)
2008

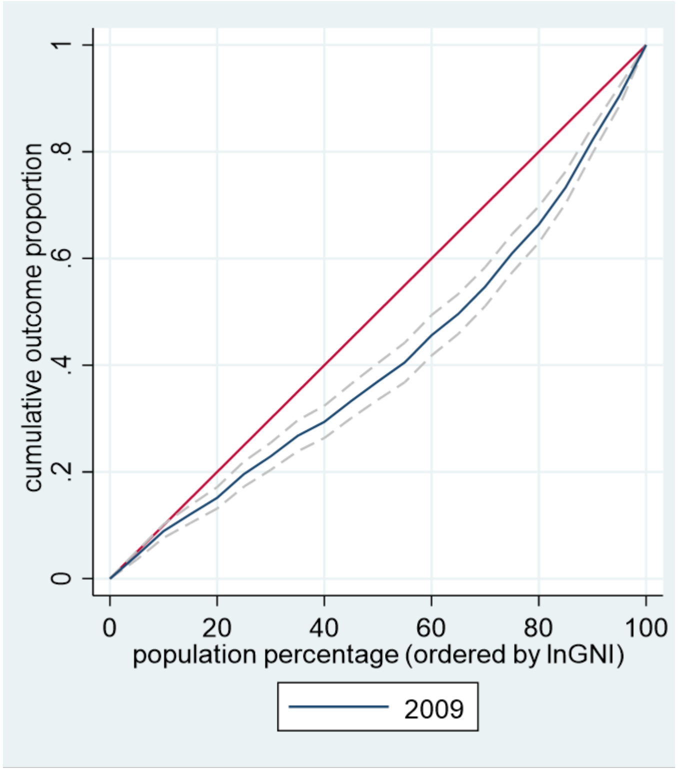

cumulative outcome proportion
0
.2
.4
.6
.8
1
0
20
40
60
80
100
population percentage (ordered by lnGNI)
2009

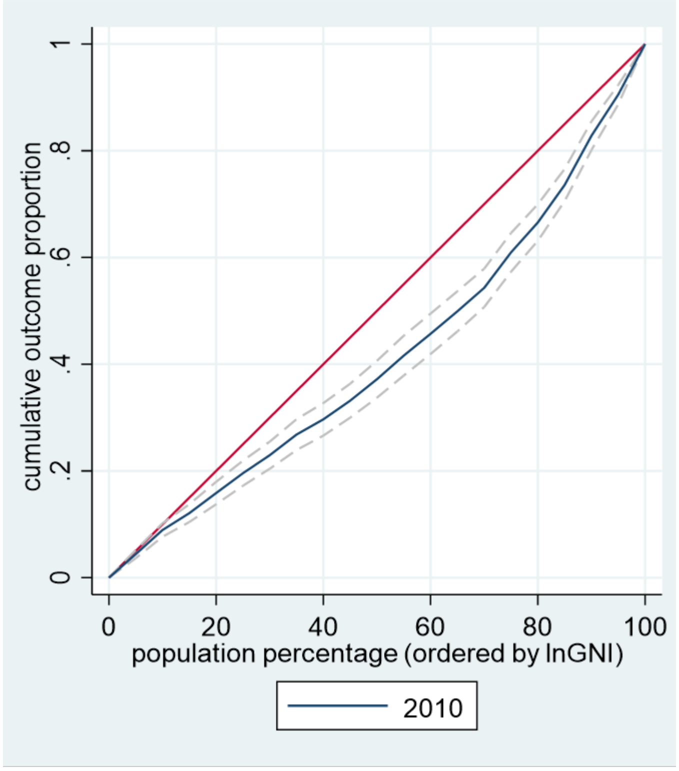

cumulative outcome proportion
0
.2
.4
.6
.8
1
0
20
40
60
80
100
population percentage (ordered by lnGNI)
2010

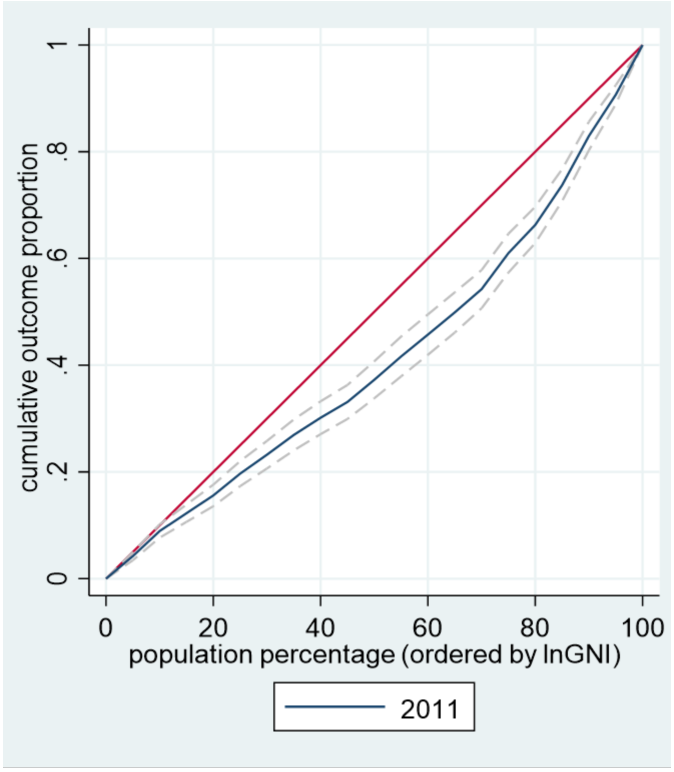
cumulative outcome proportion
0
.2
.4
.6
.8
1
0
20
40
60
80
100
population percentage (ordered by lnGNI)
2011

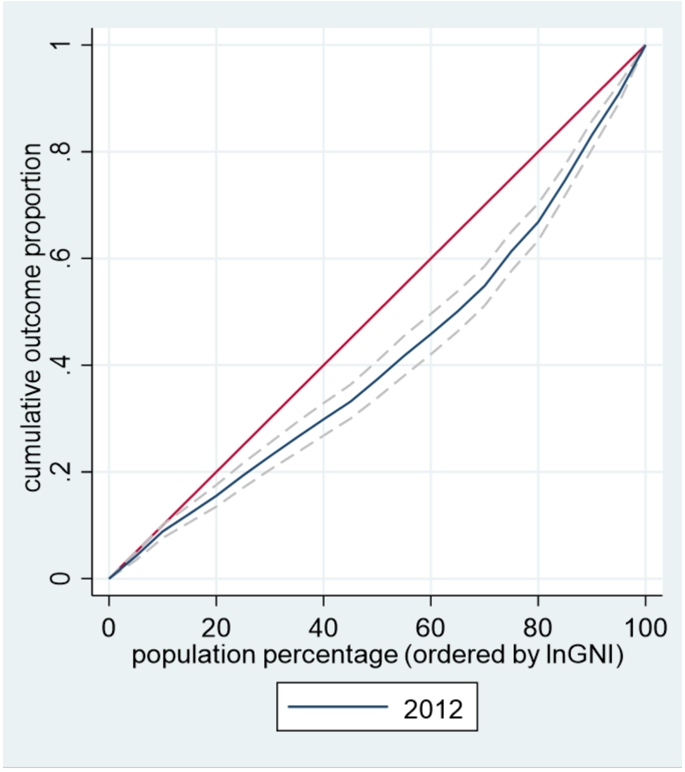
cumulative outcome proportion
0
.2
.4
.6
.8
1
0
20
40
60
80
100
population percentage (ordered by lnGNI)
2012

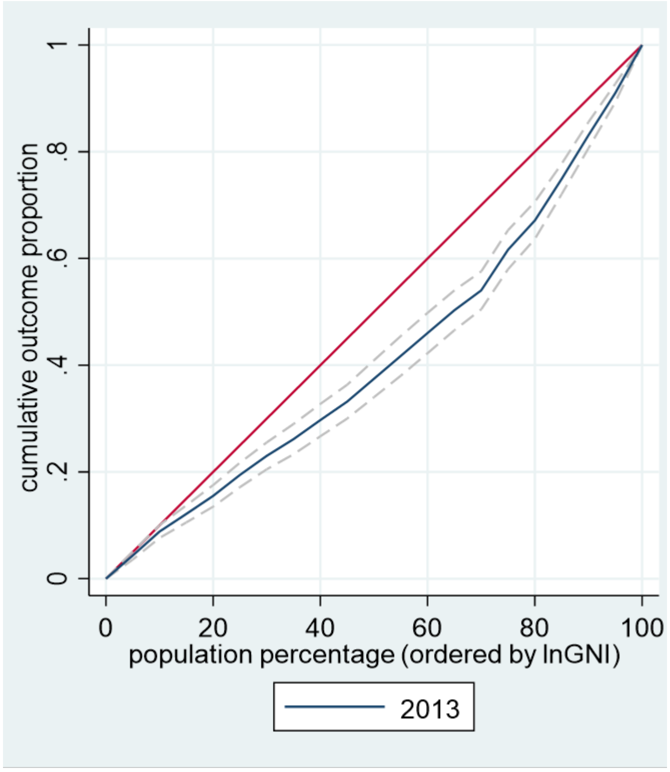
cumulative outcome proportion
0
.2
.4
.6
.8
1
0
20
40
60
80
100
population percentage (ordered by lnGNI)
2013

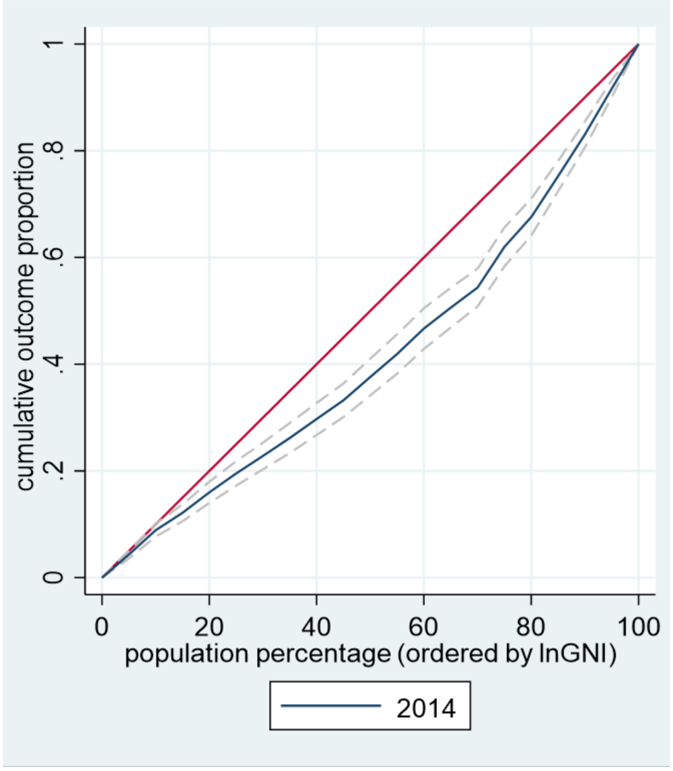
cumulative outcome proportion
0
.2
.4
.6
.8
1
0
20
40
60
80
100
population percentage (ordered by lnGNI)
2014

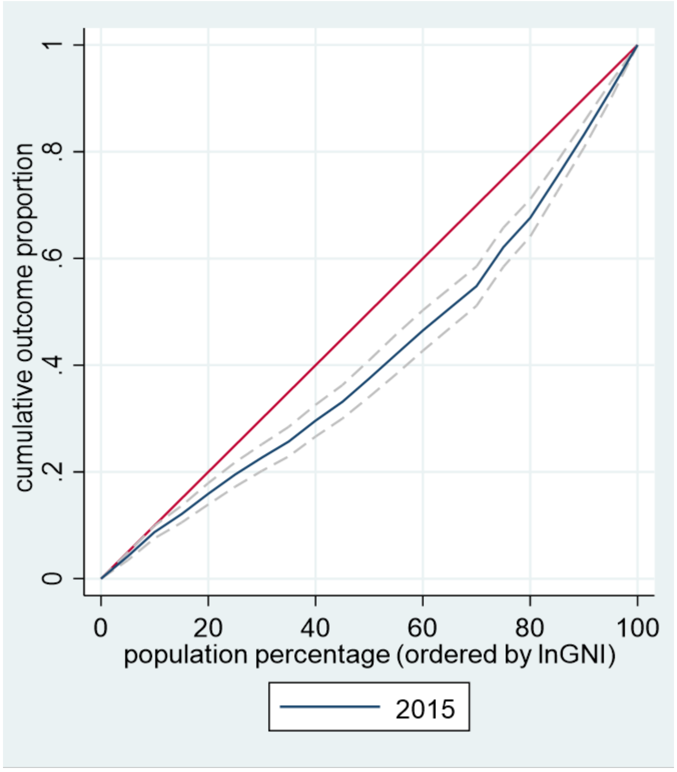
cumulative outcome proportion
0
.2
.4
.6
.8
1
0
20
40
60
80
100
population percentage (ordered by lnGNI)
2015

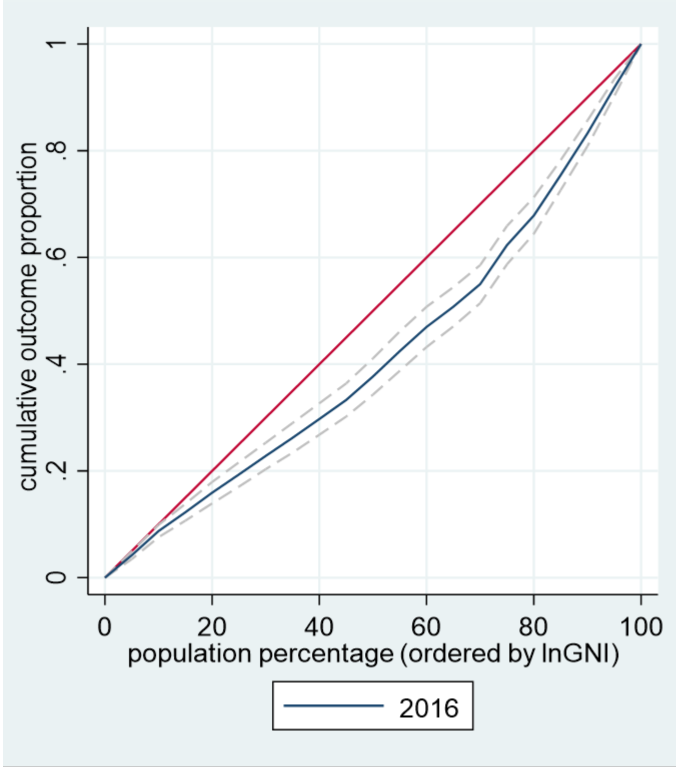
cumulative outcome proportion
0
.2
.4
.6
.8
1
0
20
40
60
80
100
population percentage (ordered by lnGNI)
2016

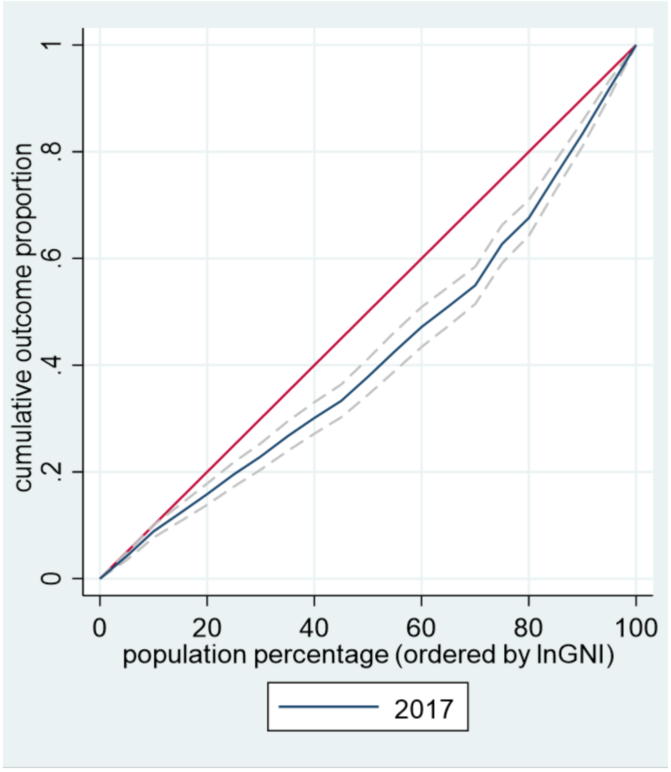

cumulative outcome proportion
0
.2
.4
.6
.8
1
0
20
40
60
80
100
population percentage (ordered by lnGNI)
2017

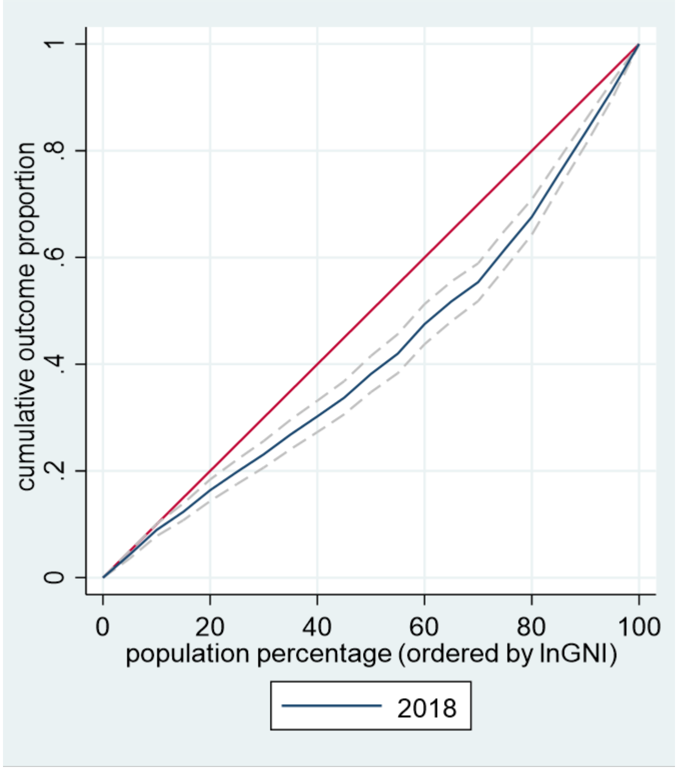

cumulative outcome proportion
0
.2
.4
.6
.8
1
0
20
40
60
80
100
population percentage (ordered by lnGNI)
2018

Figs. S120-137 Concentration curves of age-standardized DALY rates for cardiomyopathy and myocarditis from 2001 to 2018

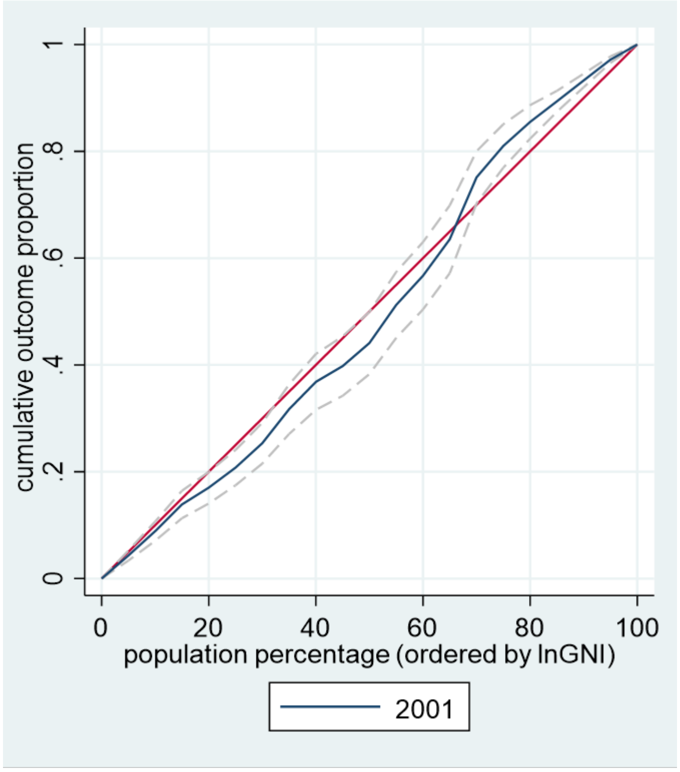

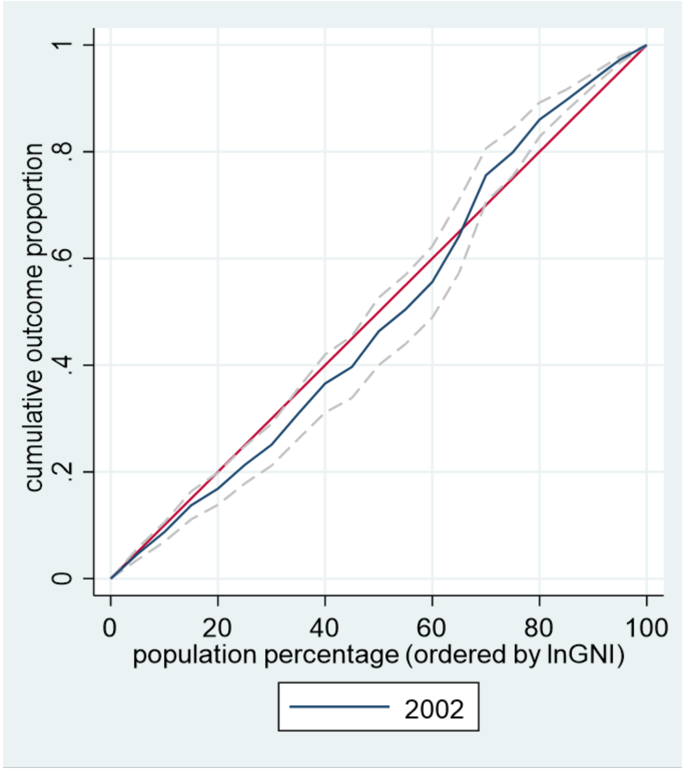

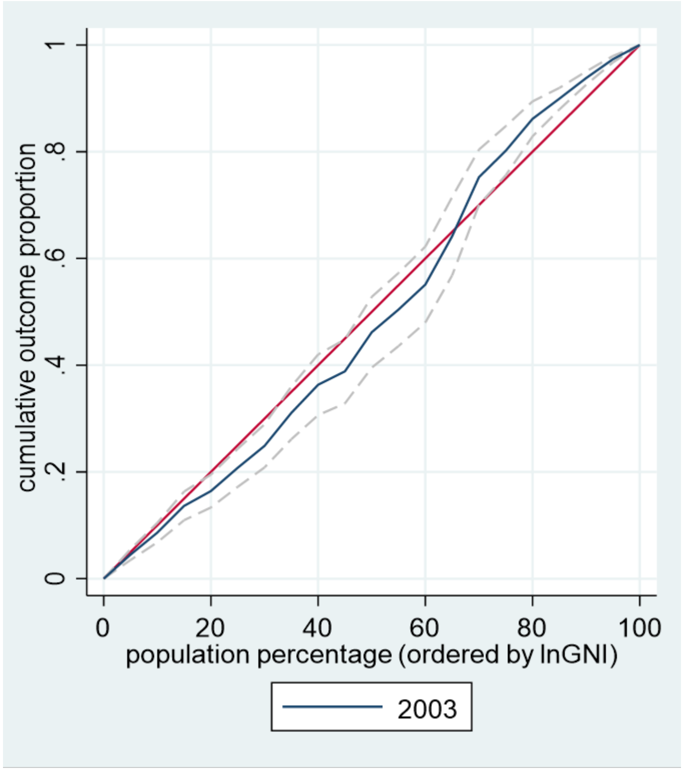
cumulative outcome proportion
0
.2
.4
.6
.8
1
0
20
40
60
80
100
population percentage (ordered by lnGNI)
2003

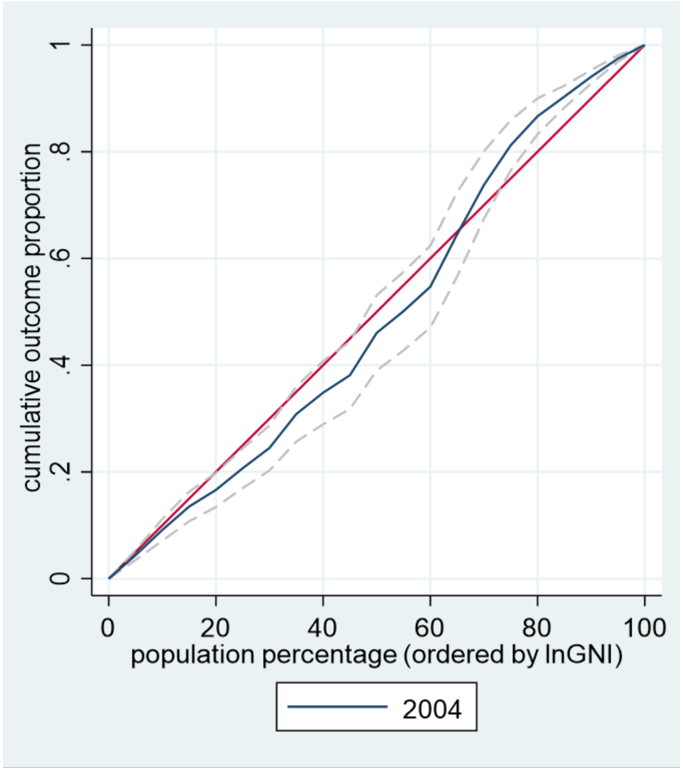
cumulative outcome proportion
0
.2
.4
.6
.8
1
0
20
40
60
80
100
population percentage (ordered by lnGNI)
2004

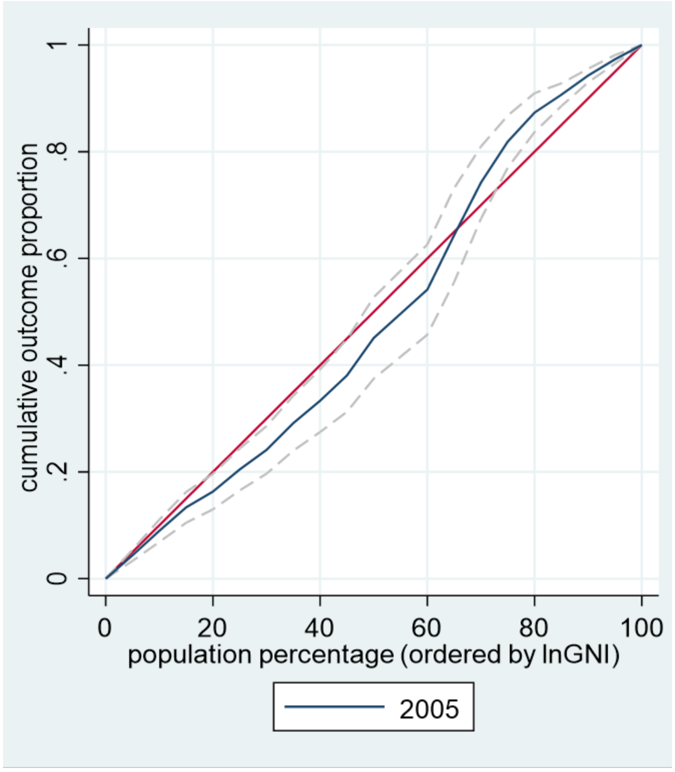

cumulative outcome proportion
0
.2
.4
.6
.8
1
0
20
40
60
80
100
population percentage (ordered by lnGNI)
2005

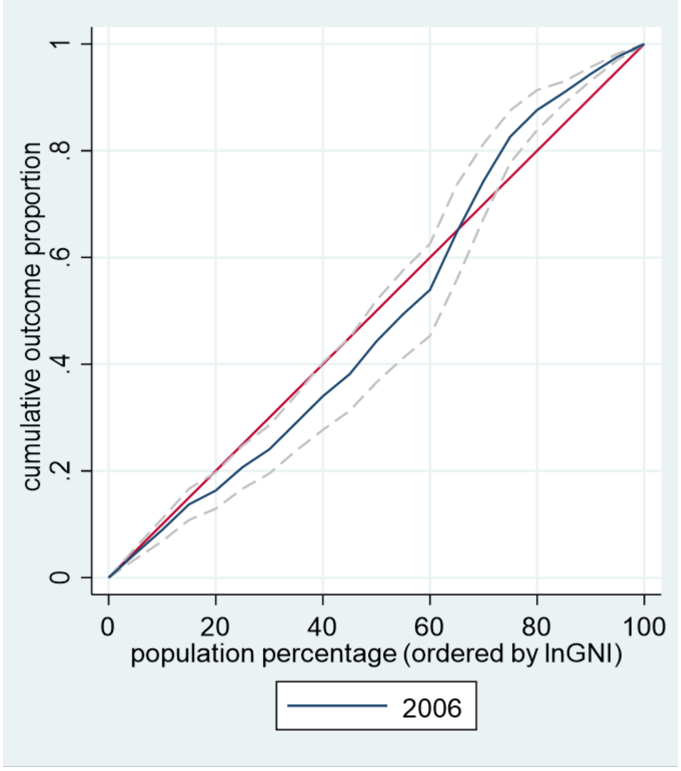

cumulative outcome proportion
0
.2
.4
.6
.8
1
0
20
40
60
80
100
population percentage (ordered by lnGNI)
2006

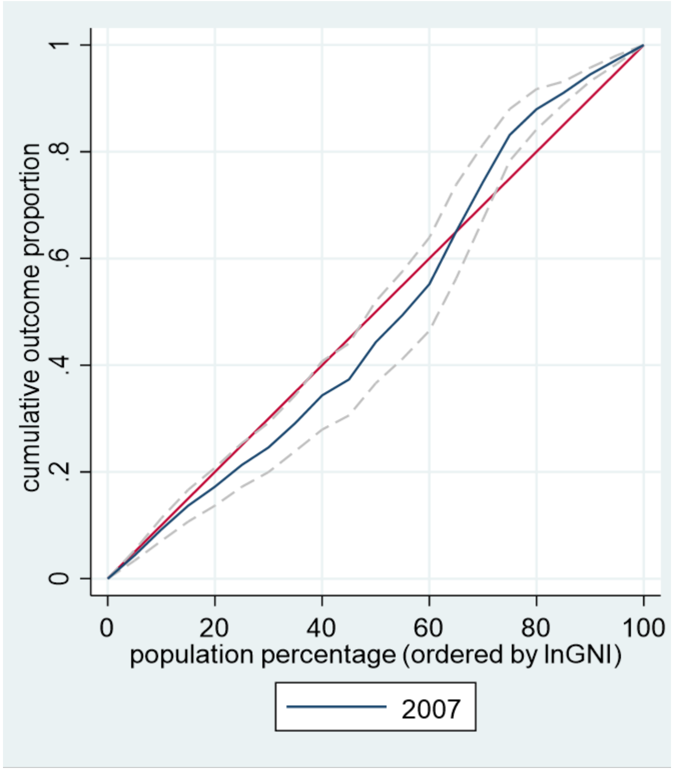

cumulative outcome proportion
0
.2
.4
.6
.8
1
0
20
40
60
80
100
population percentage (ordered by lnGNI)
2007

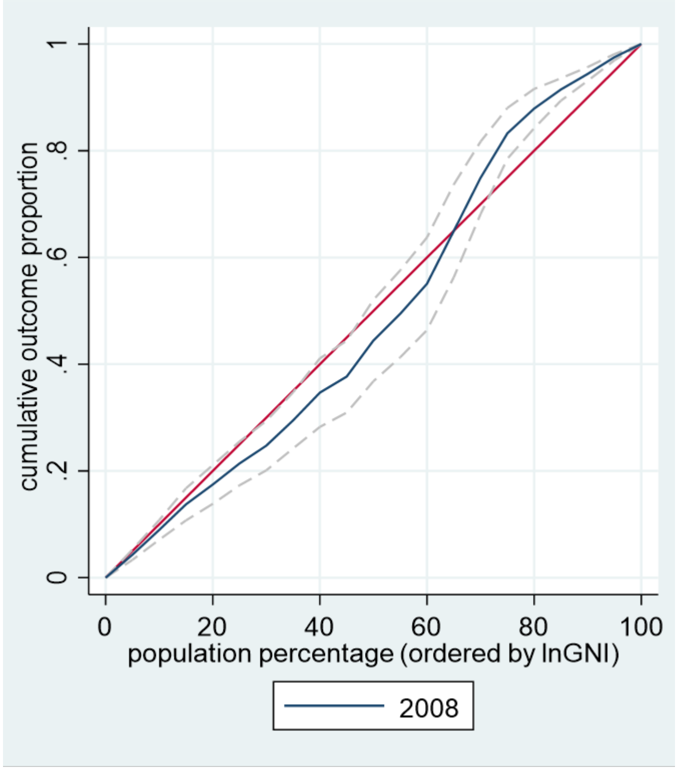

cumulative outcome proportion
0
.2
.4
.6
.8
1
0
20
40
60
80
100
population percentage (ordered by lnGNI)
2008

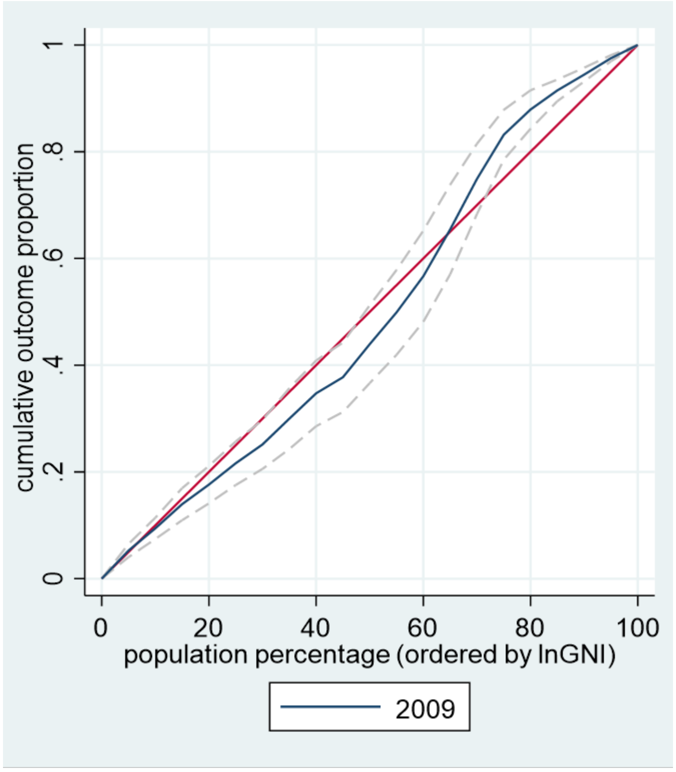

cumulative outcome proportion
0
.2
.4
.6
.8
1
0
20
40
60
80
100
population percentage (ordered by lnGNI)
2009

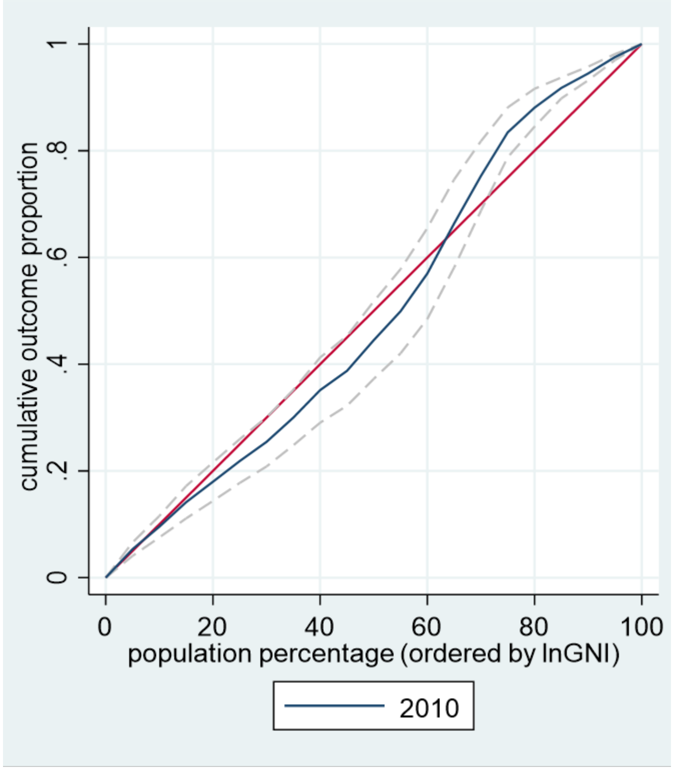

cumulative outcome proportion
0
.2
.4
.6
.8
1
0
20
40
60
80
100
population percentage (ordered by lnGNI)
2010

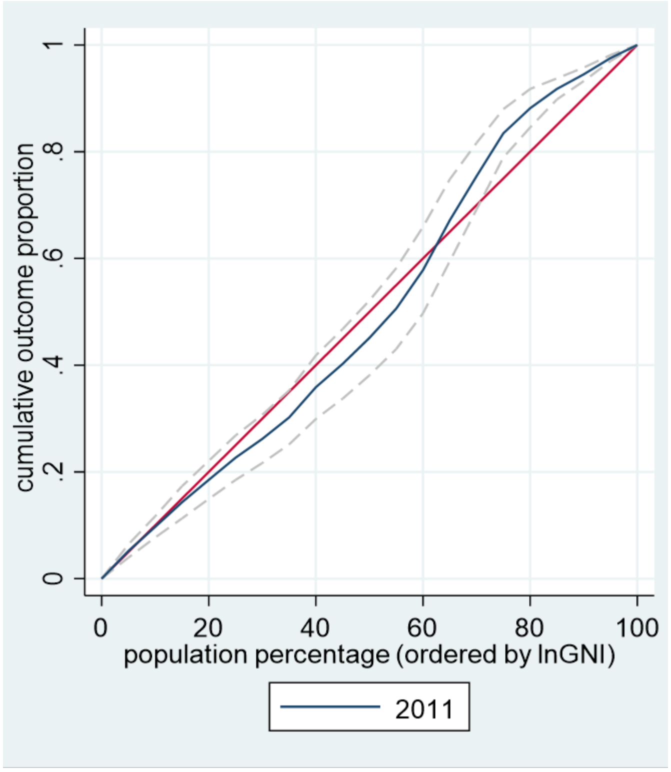
cumulative outcome proportion
0
.2
.4
.6
.8
1
0
20
40
60
80
100
population percentage (ordered by lnGNI)
2011

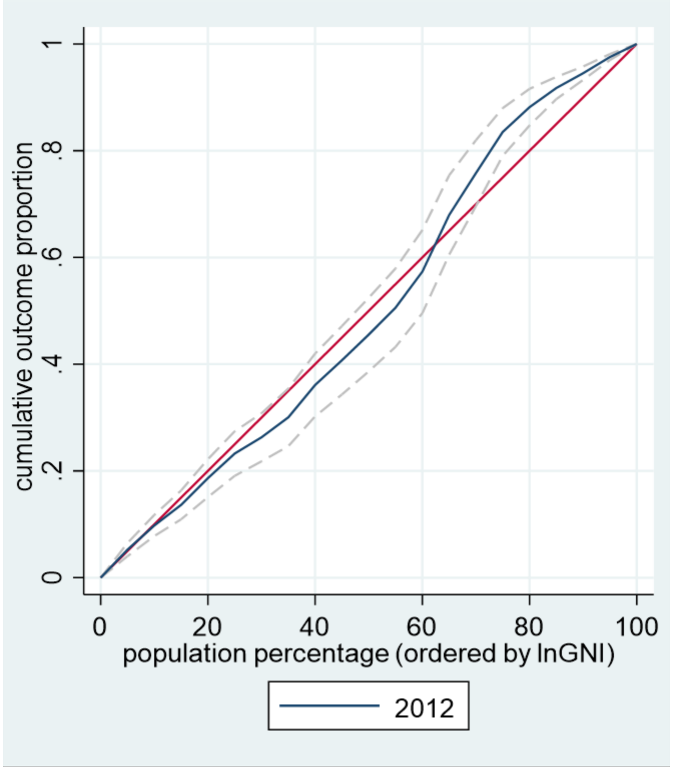
cumulative outcome proportion
0
.2
.4
.6
.8
1
0
20
40
60
80
100
population percentage (ordered by lnGNI)
2012

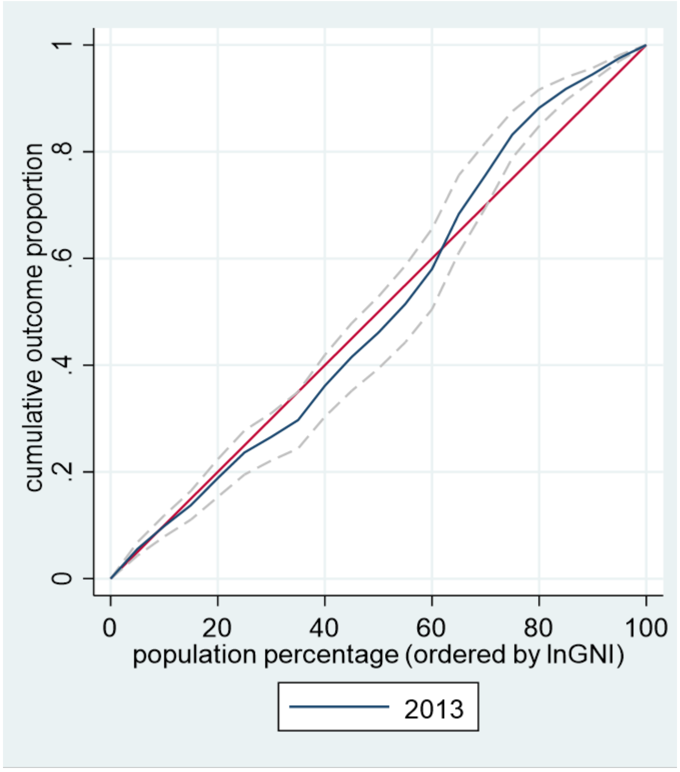

cumulative outcome proportion
0
.2
.4
.6
.8
1
0
20
40
60
80
100
population percentage (ordered by lnGNI)
2013

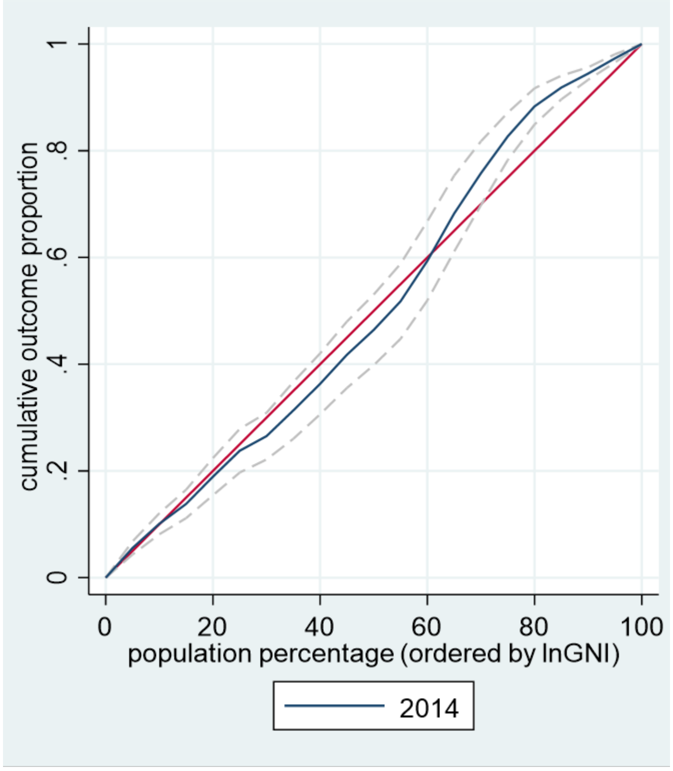

cumulative outcome proportion
0
.2
.4
.6
.8
1
0
20
40
60
80
100
population percentage (ordered by lnGNI)
2014

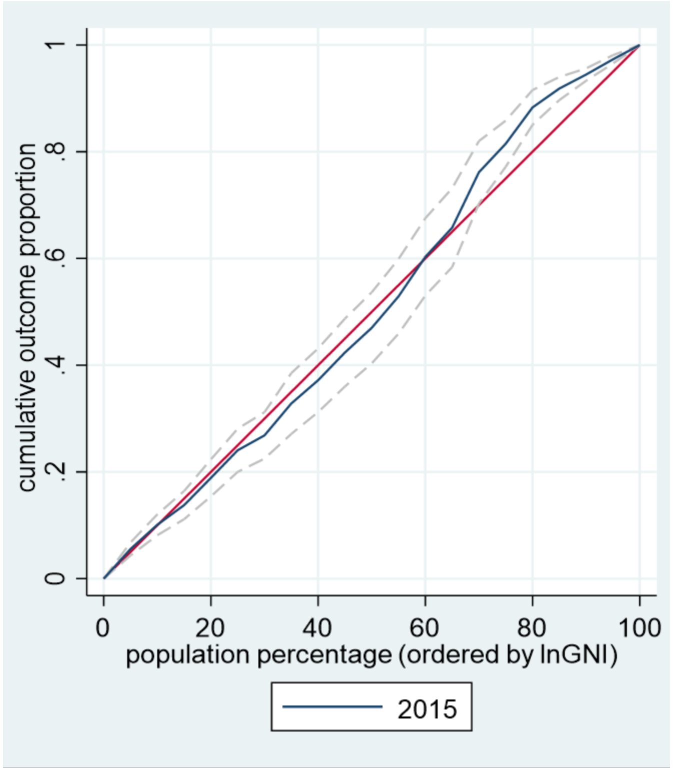

cumulative outcome proportion
0
.2
.4
.6
.8
1
0
20
40
60
80
100
population percentage (ordered by lnGNI)
2015

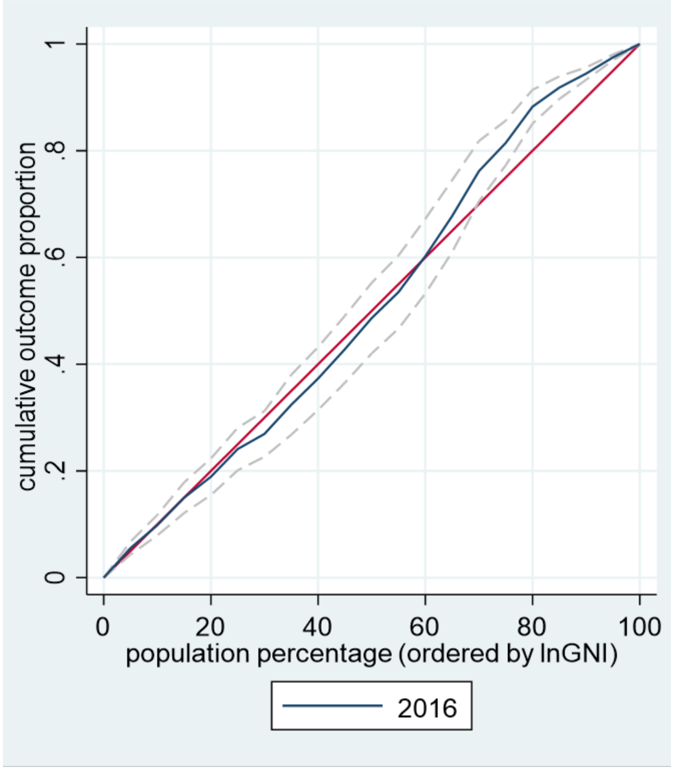

cumulative outcome proportion
0
.2
.4
.6
.8
1
0
20
40
60
80
100
population percentage (ordered by lnGNI)
2016

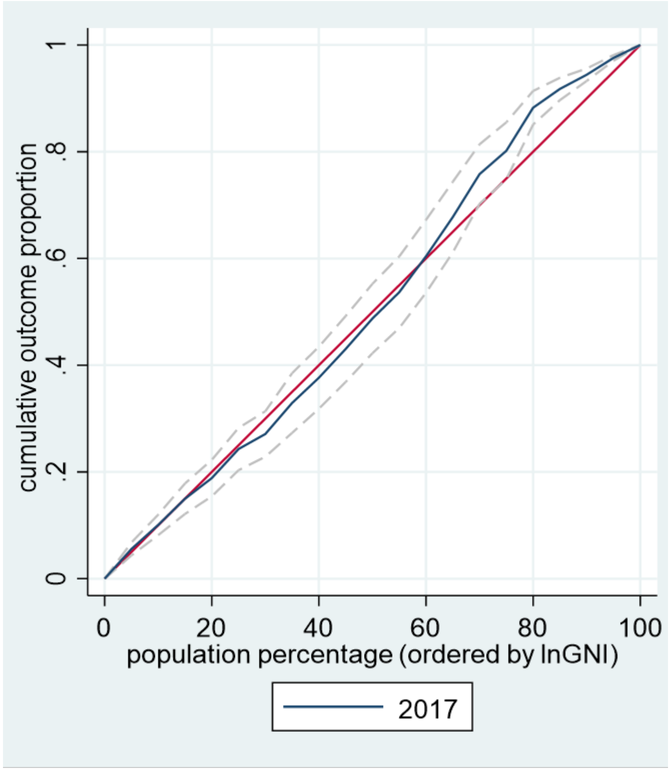
cumulative outcome proportion
0
.2
.4
.6
.8
1
0
20
40
60
80
100
population percentage (ordered by lnGNI)
2017

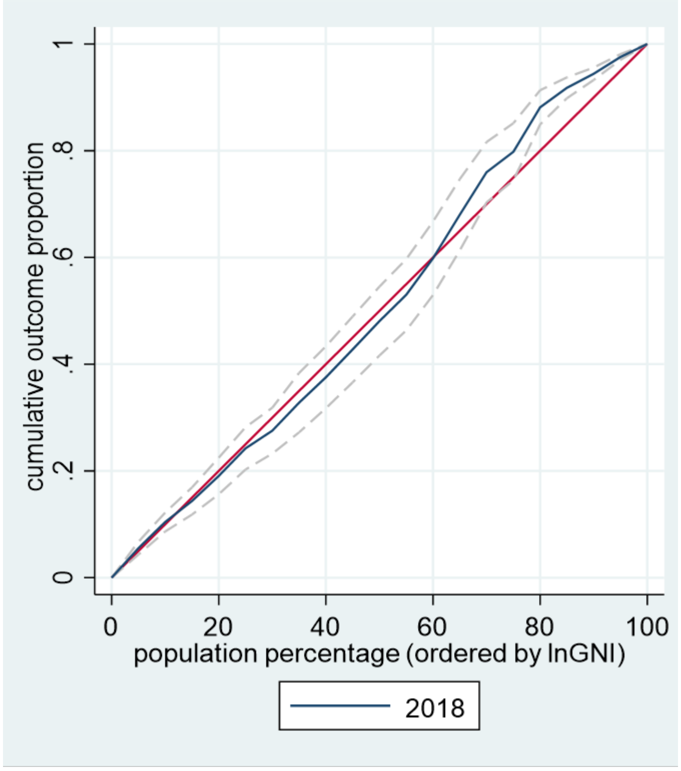
cumulative outcome proportion
0
.2
.4
.6
.8
1
0
20
40
60
80
100
population percentage (ordered by lnGNI)
2018

Figs. S138-155 Concentration curves of age-standardized DALY rates for atrial fibrillation and flutter from 2001 to 2018

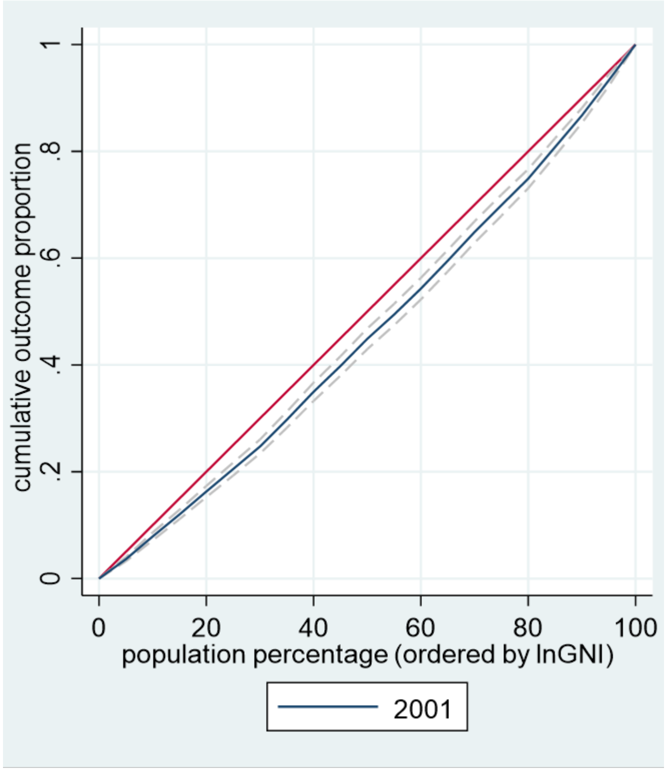

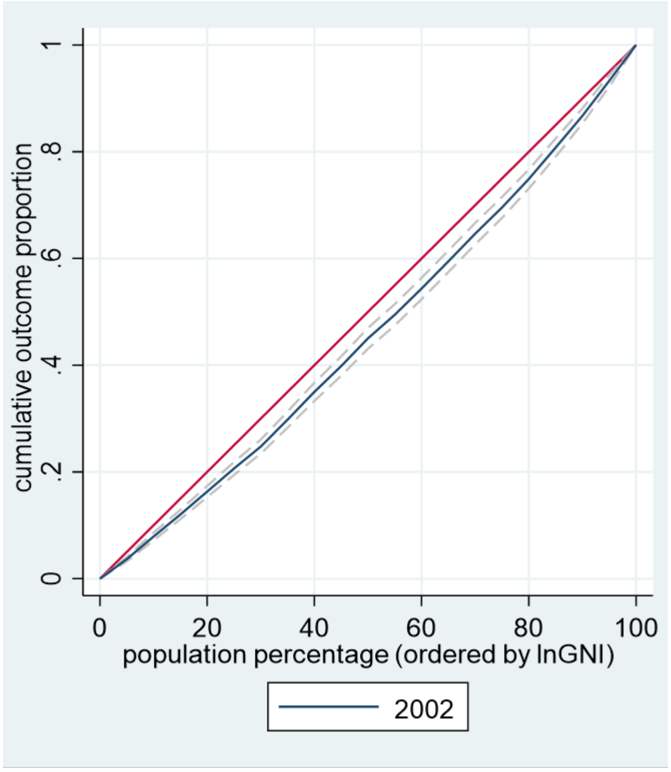

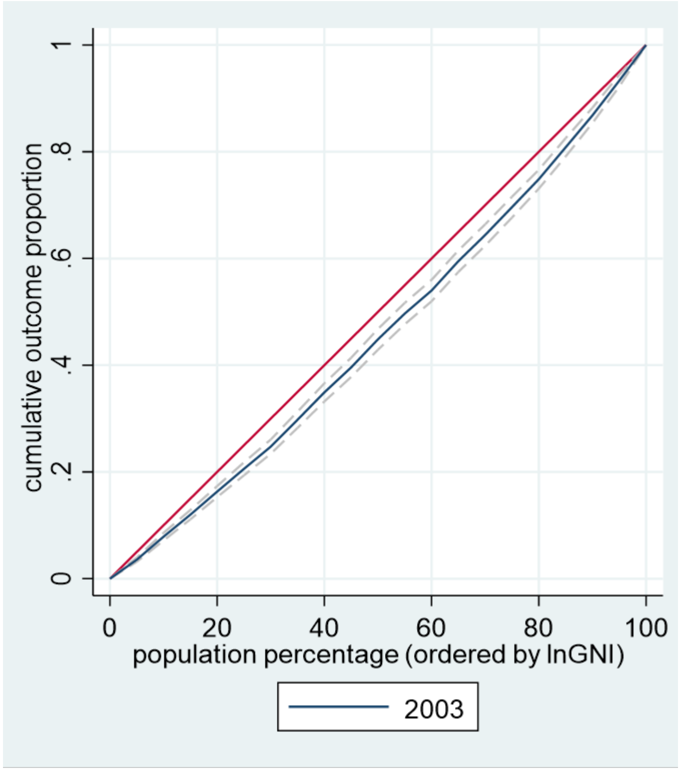

cumulative outcome proportion
0
.2
.4
.6
.8
1
0
20
40
60
80
100
population percentage (ordered by lnGNI)
2003

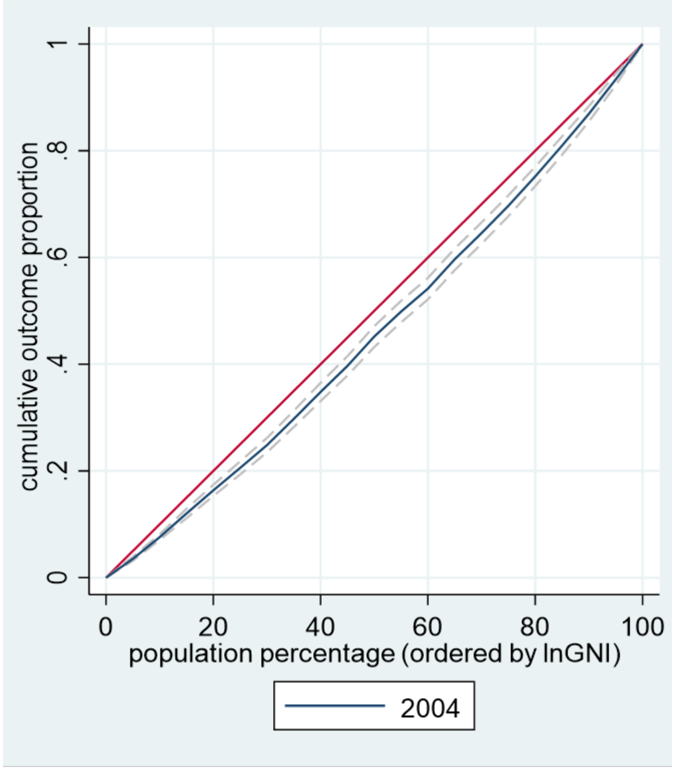

cumulative outcome proportion
0
.2
.4
.6
.8
1
0
20
40
60
80
100
population percentage (ordered by lnGNI)
2004

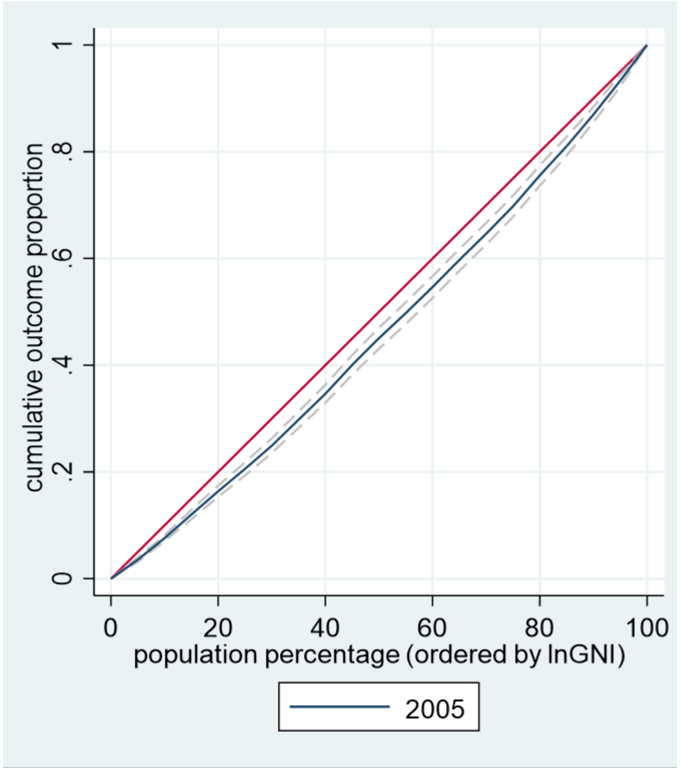

cumulative outcome proportion
0
.2
.4
.6
.8
1
0
20
40
60
80
100
population percentage (ordered by lnGNI)
2005

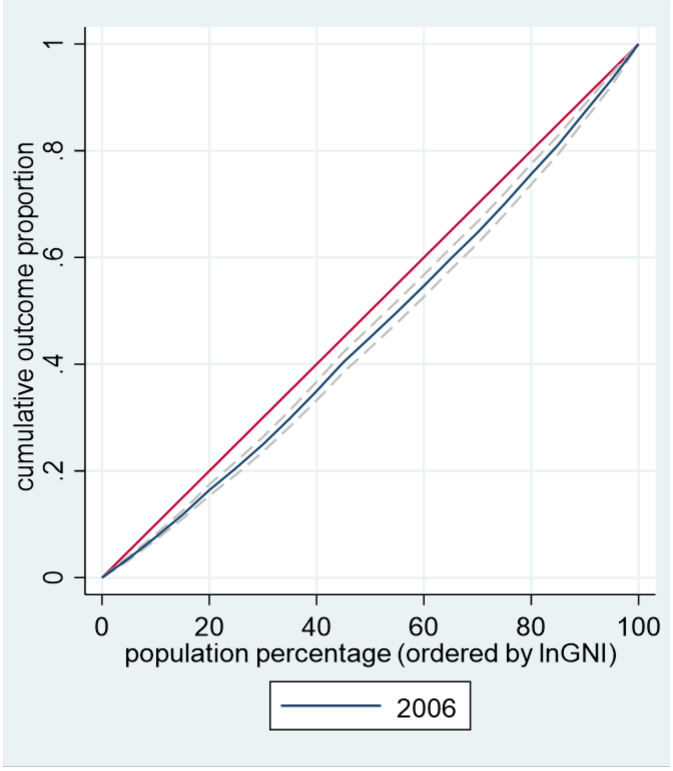

cumulative outcome proportion
0
.2
.4
.6
.8
1
0
20
40
60
80
100
population percentage (ordered by lnGNI)
2006

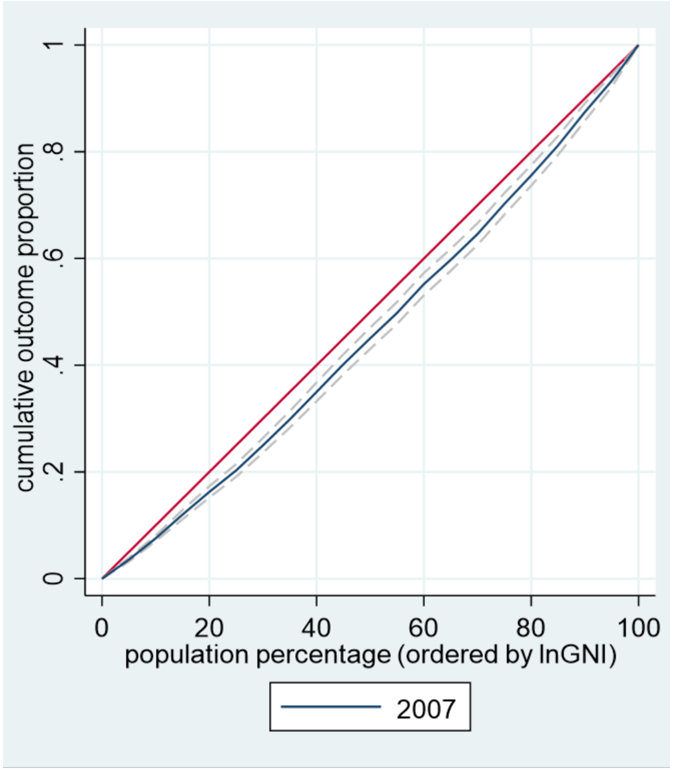
cumulative outcome proportion
0
.2
.4
.6
.8
1
0
20
40
60
80
100
population percentage (ordered by lnGNI)
2007

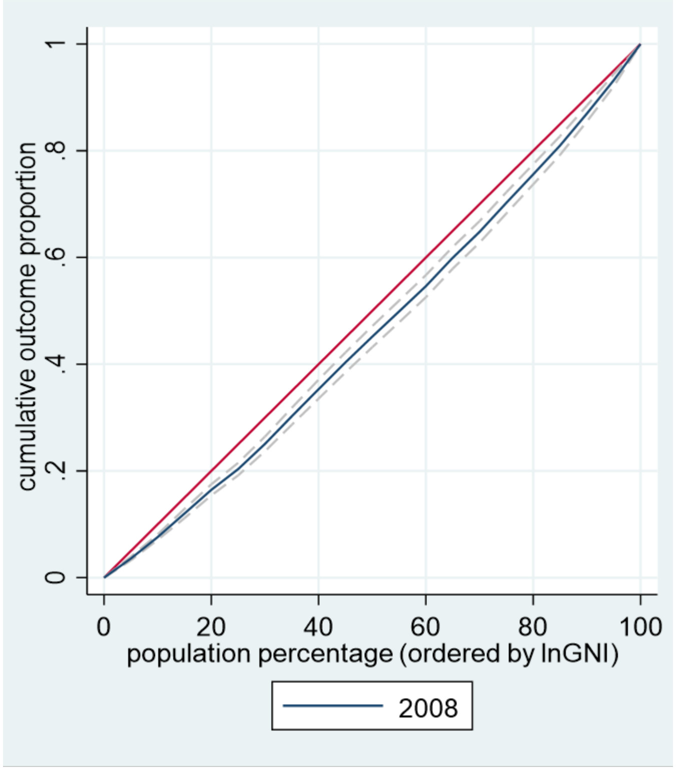
cumulative outcome proportion
0
.2
.4
.6
.8
1
0
20
40
60
80
100
population percentage (ordered by lnGNI)
2008

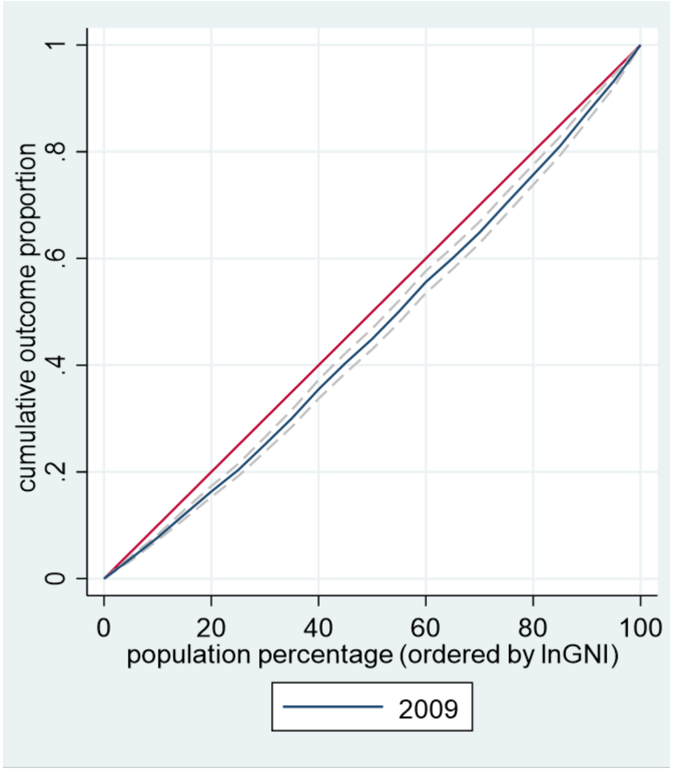
cumulative outcome proportion
0
.2
.4
.6
.8
1
0
20
40
60
80
100
population percentage (ordered by lnGNI)
2009

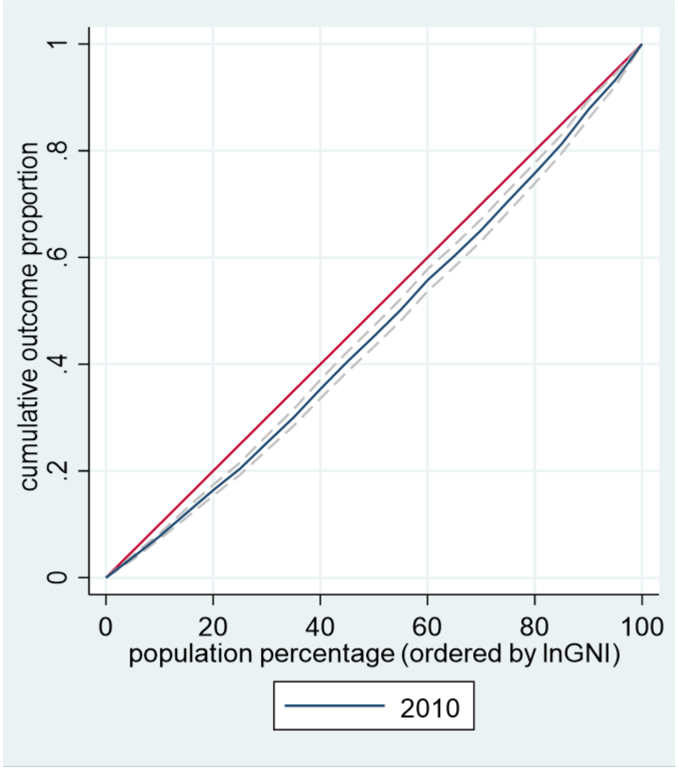
cumulative outcome proportion
0
.2
.4
.6
.8
1
0
20
40
60
80
100
population percentage (ordered by lnGNI)
2010

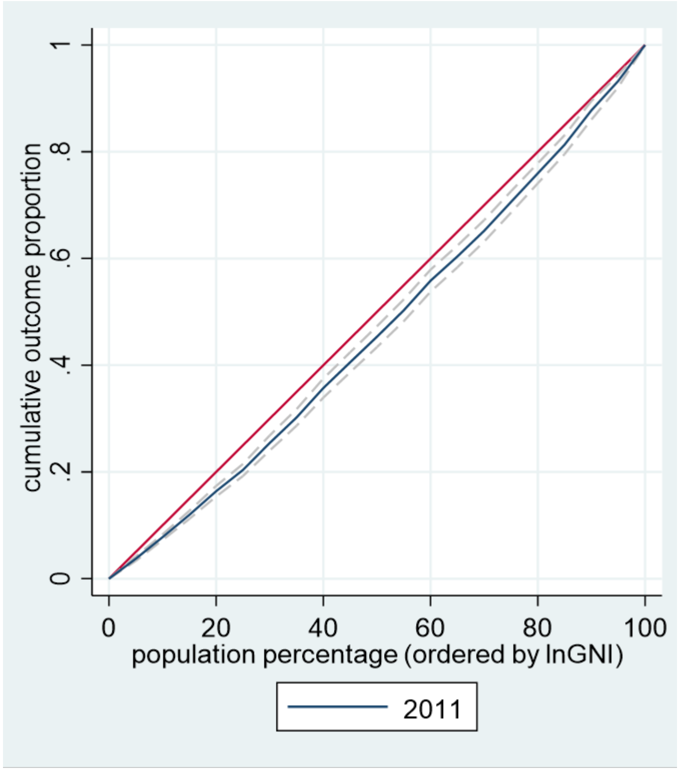
cumulative outcome proportion
0
.2
.4
.6
.8
1
0
20
40
60
80
100
population percentage (ordered by lnGNI)
2011

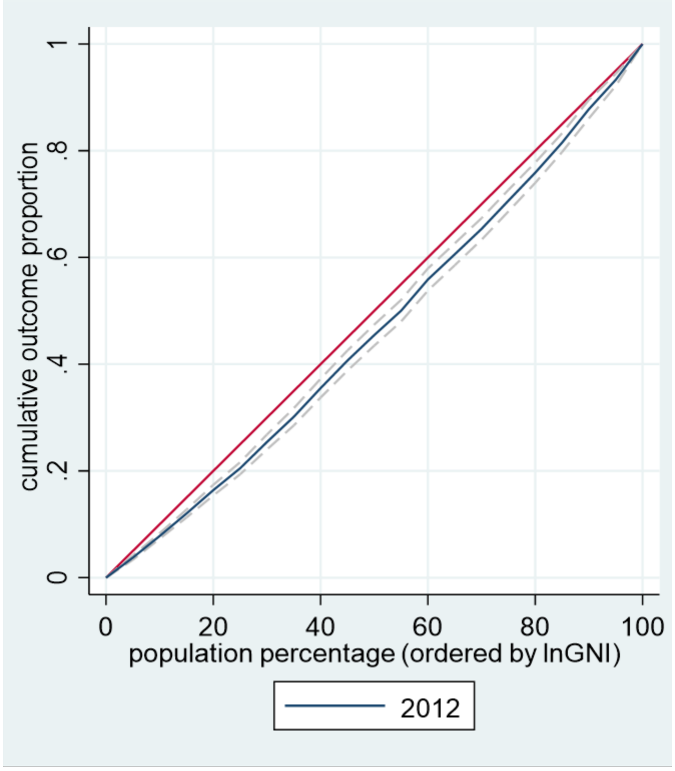
cumulative outcome proportion
0
.2
.4
.6
.8
1
0
20
40
60
80
100
population percentage (ordered by lnGNI)
2012

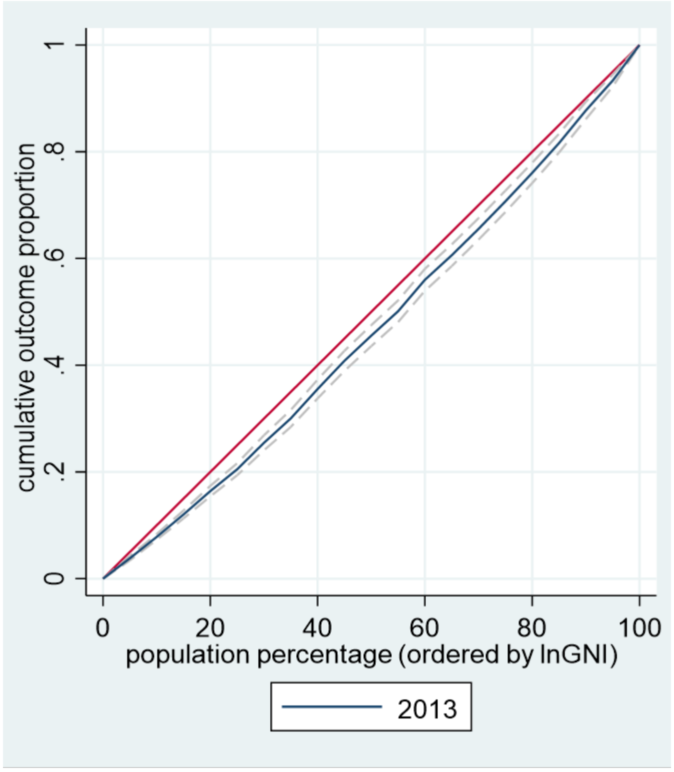

cumulative outcome proportion
0
.2
.4
.6
.8
1
0
20
40
60
80
100
population percentage (ordered by lnGNI)
2013

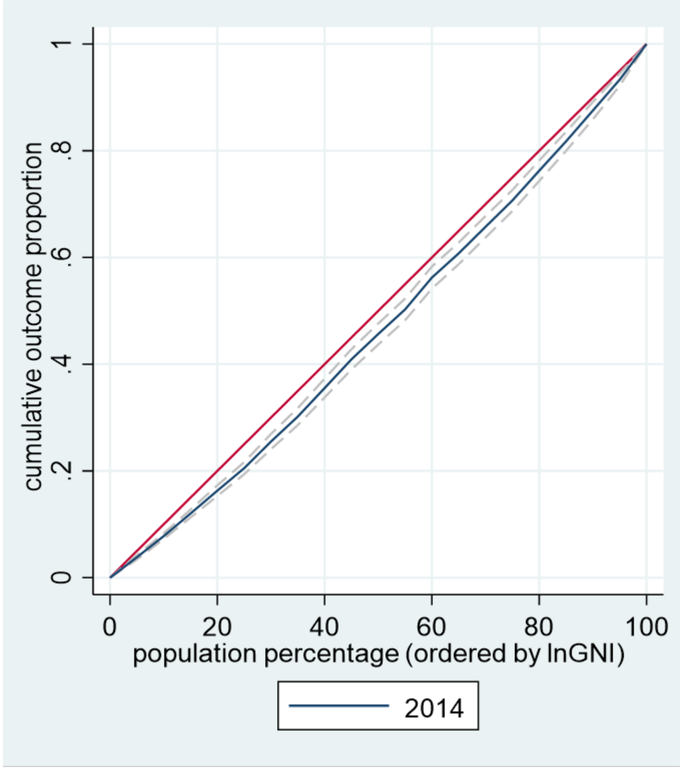

cumulative outcome proportion
0
.2
.4
.6
.8
1
0
20
40
60
80
100
population percentage (ordered by lnGNI)
2014

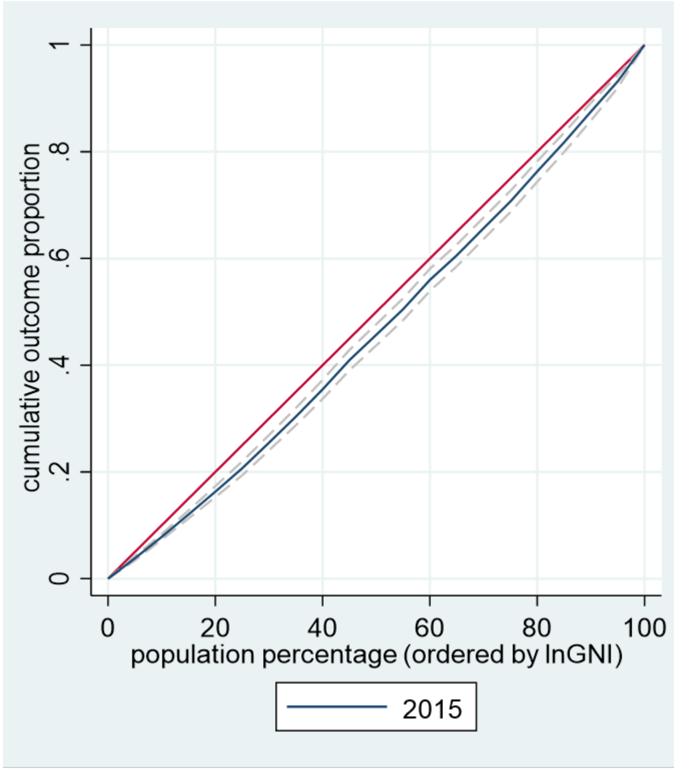
cumulative outcome proportion
0
.2
.4
.6
.8
1
0
20
40
60
80
100
population percentage (ordered by lnGNI)
2015

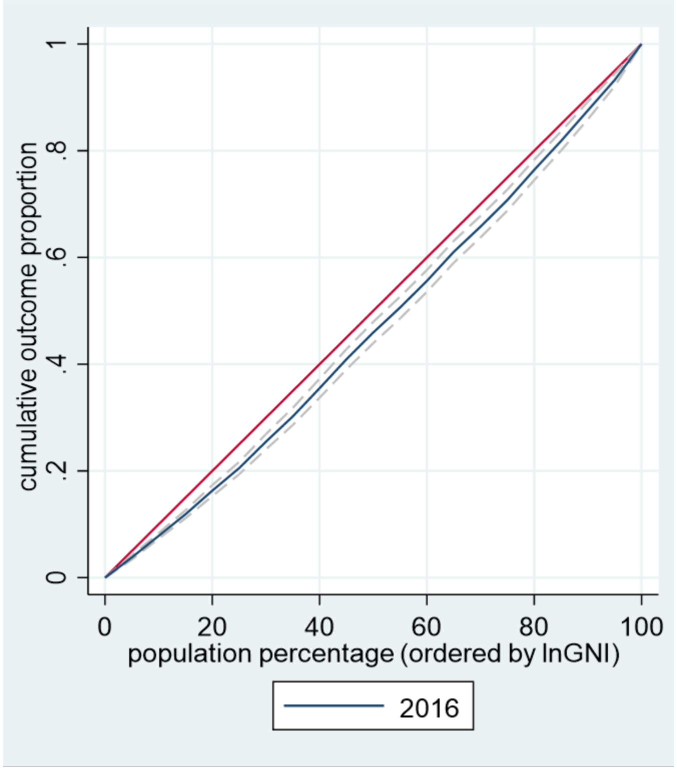
cumulative outcome proportion
0
.2
.4
.6
.8
1
0
20
40
60
80
100
population percentage (ordered by lnGNI)
2016

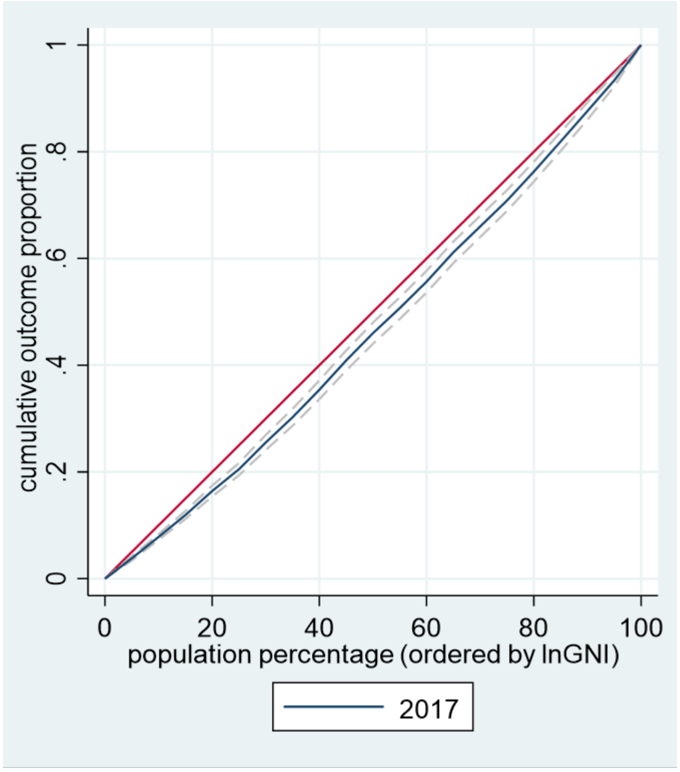
cumulative outcome proportion
0
.2
.4
.6
.8
1
0
20
40
60
80
100
population percentage (ordered by lnGNI)
2017

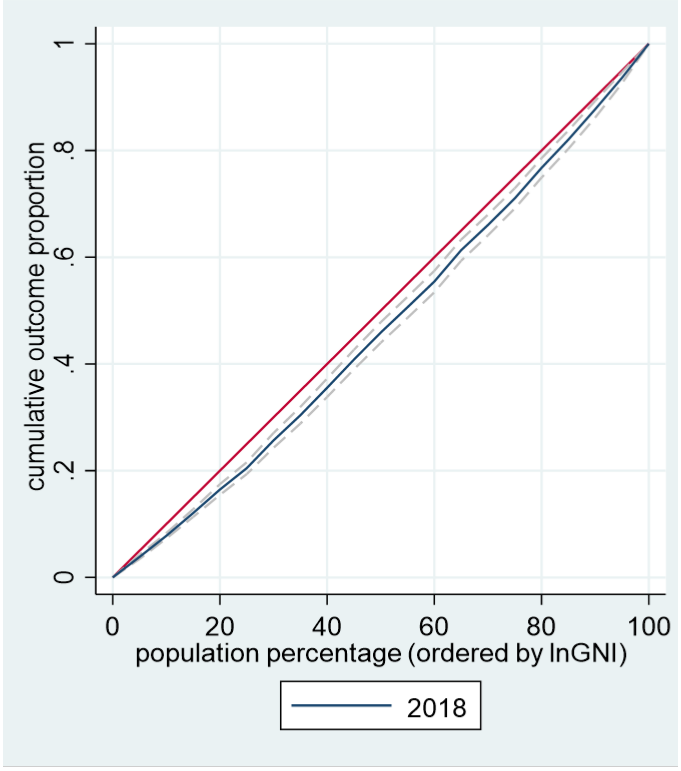
cumulative outcome proportion
0
.2
.4
.6
.8
1
0
20
40
60
80
100
population percentage (ordered by lnGNI)
2018

Figs. S156-173 Concentration curves of age-standardized DALY rates for aortic aneurysm from 2001 to 2018

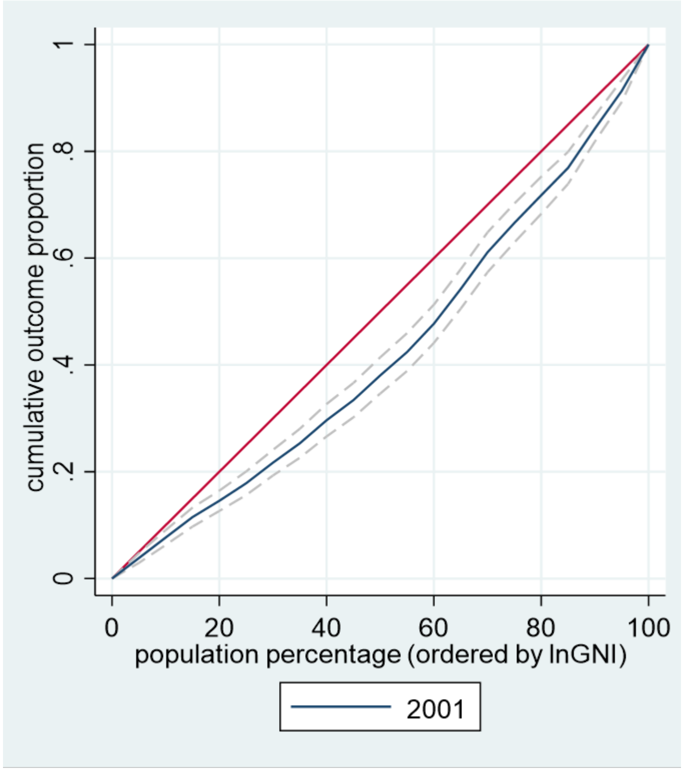

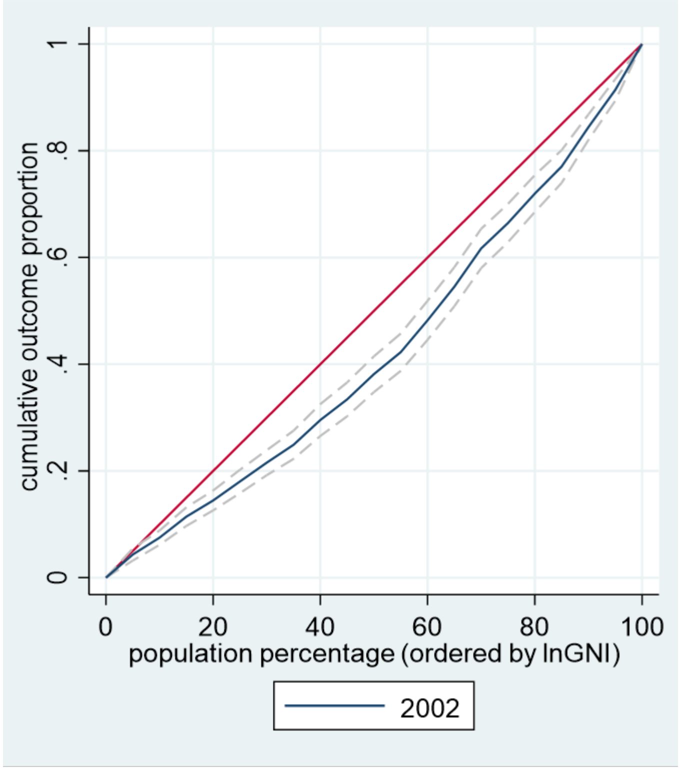

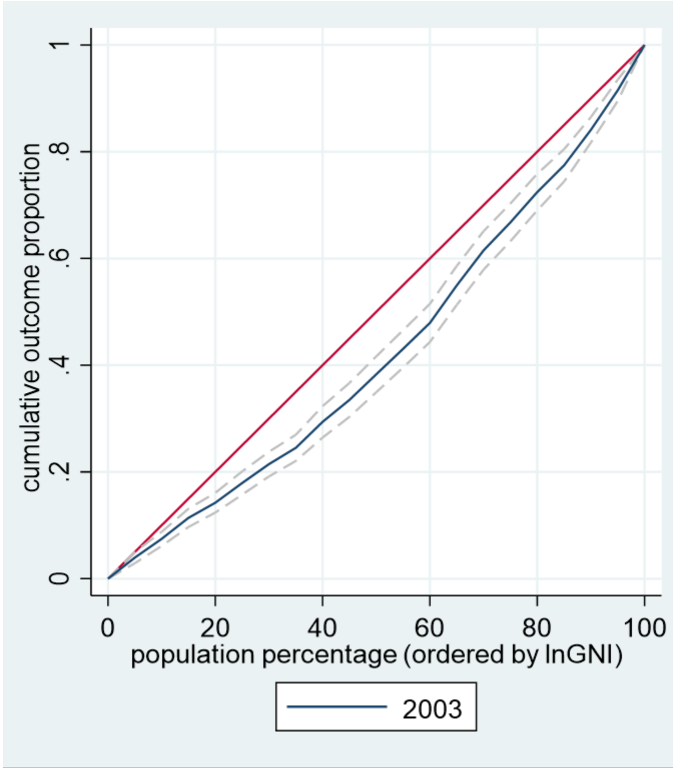

cumulative outcome proportion
0
.2
.4
.6
.8
1
0
20
40
60
80
100
population percentage (ordered by lnGNI)
2003

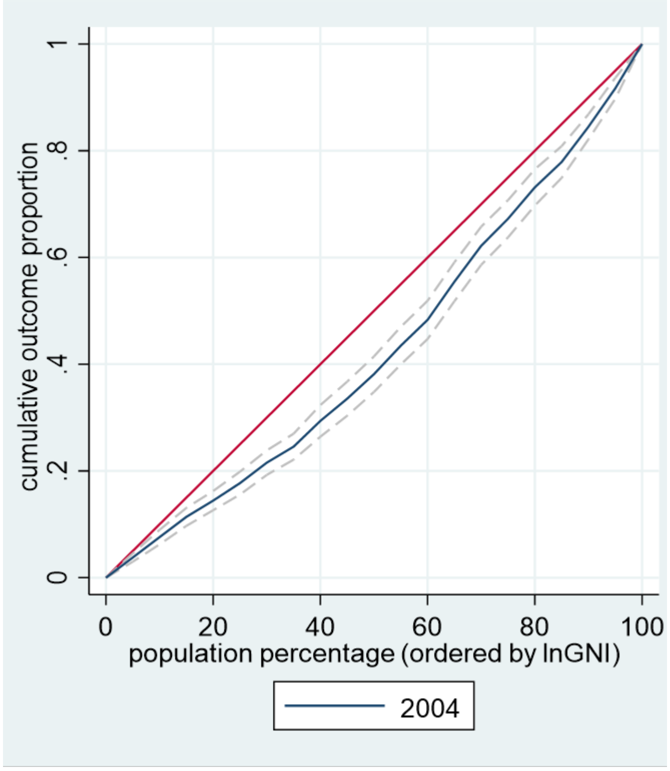

cumulative outcome proportion
0
.2
.4
.6
.8
1
0
20
40
60
80
100
population percentage (ordered by lnGNI)
2004

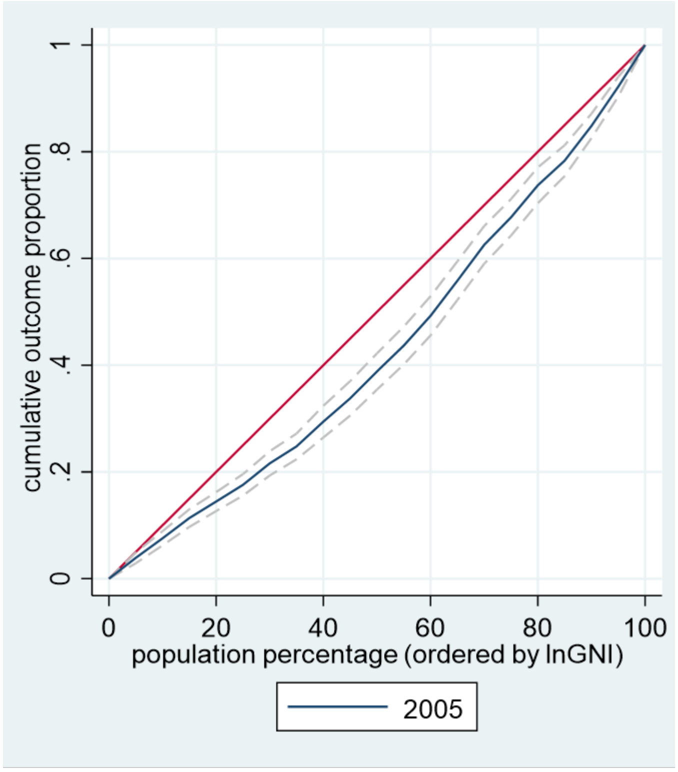

cumulative outcome proportion
0
.2
.4
.6
.8
1
0
20
40
60
80
100
population percentage (ordered by lnGNI)
2005

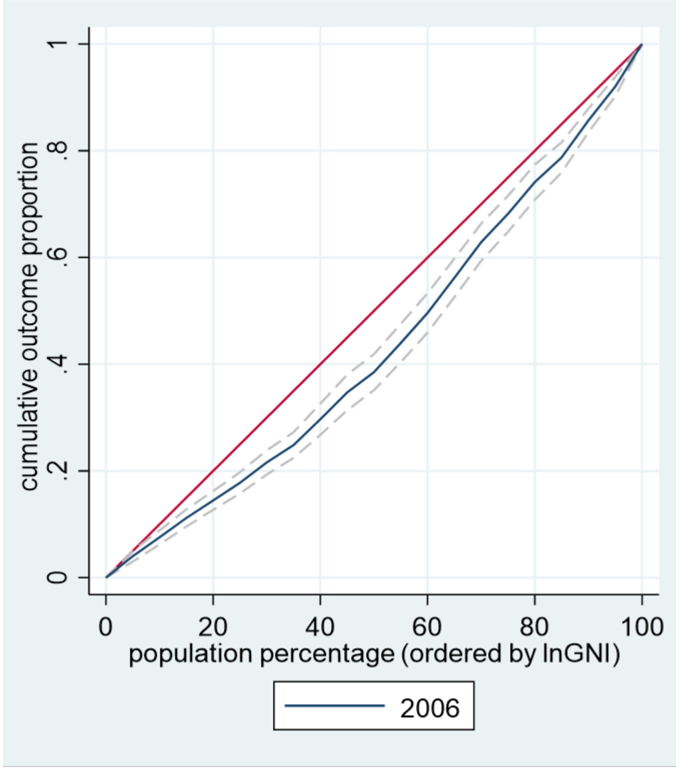

cumulative outcome proportion
0
.2
.4
.6
.8
1
0
20
40
60
80
100
population percentage (ordered by lnGNI)
2006

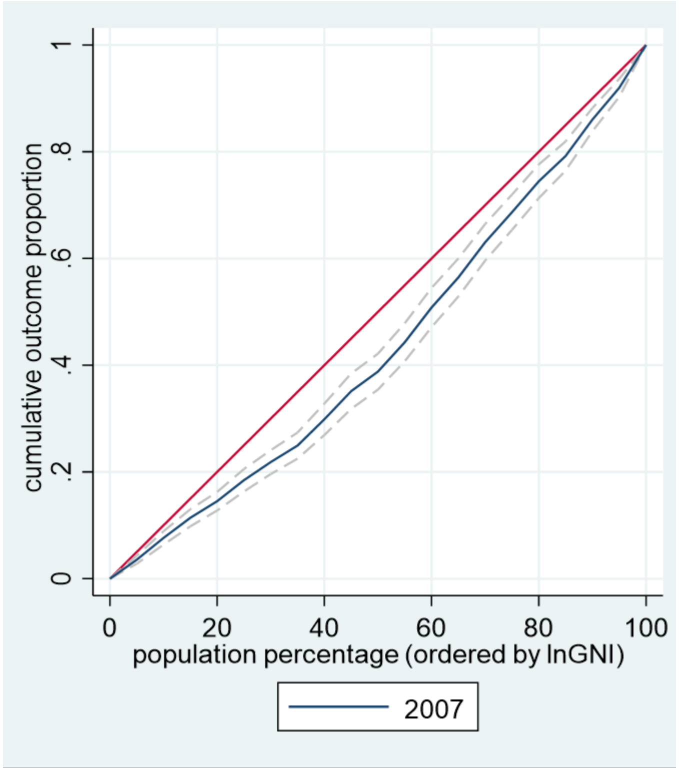
cumulative outcome proportion
0
.2
.4
.6
.8
1
0
20
40
60
80
100
population percentage (ordered by lnGNI)
2007

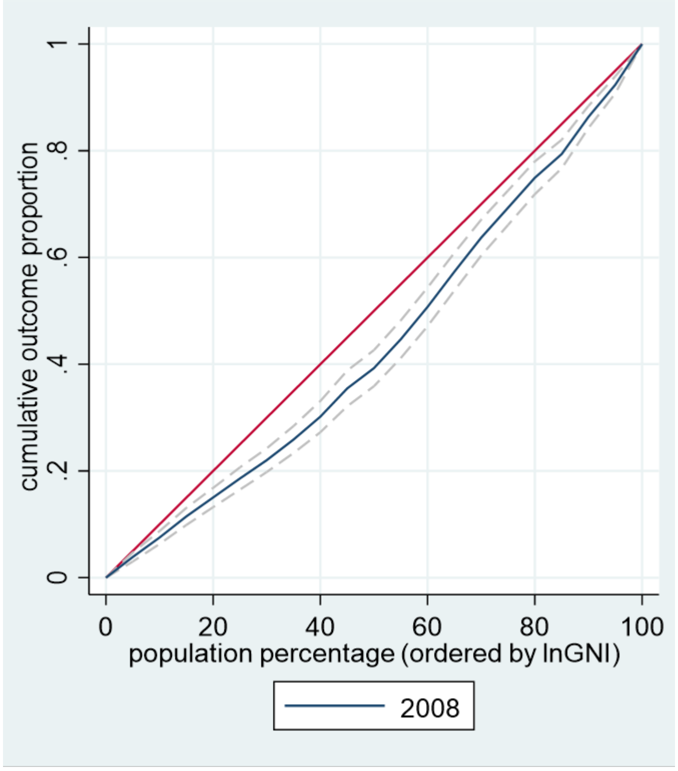
cumulative outcome proportion
0
.2
.4
.6
.8
1
0
20
40
60
80
100
population percentage (ordered by lnGNI)
2008

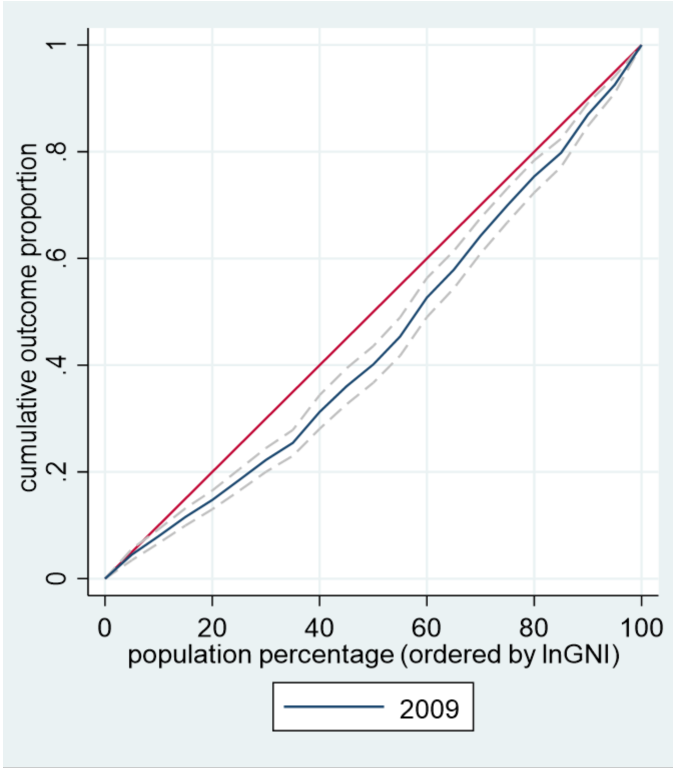
cumulative outcome proportion
0
.2
.4
.6
.8
1
0
20
40
60
80
100
population percentage (ordered by lnGNI)
2009

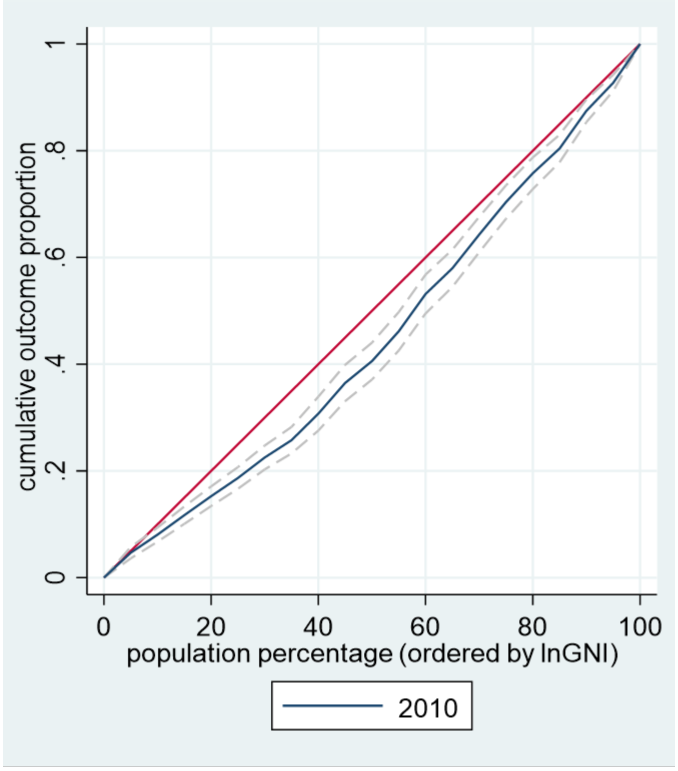
cumulative outcome proportion
0
.2
.4
.6
.8
1
0
20
40
60
80
100
population percentage (ordered by lnGNI)
2010

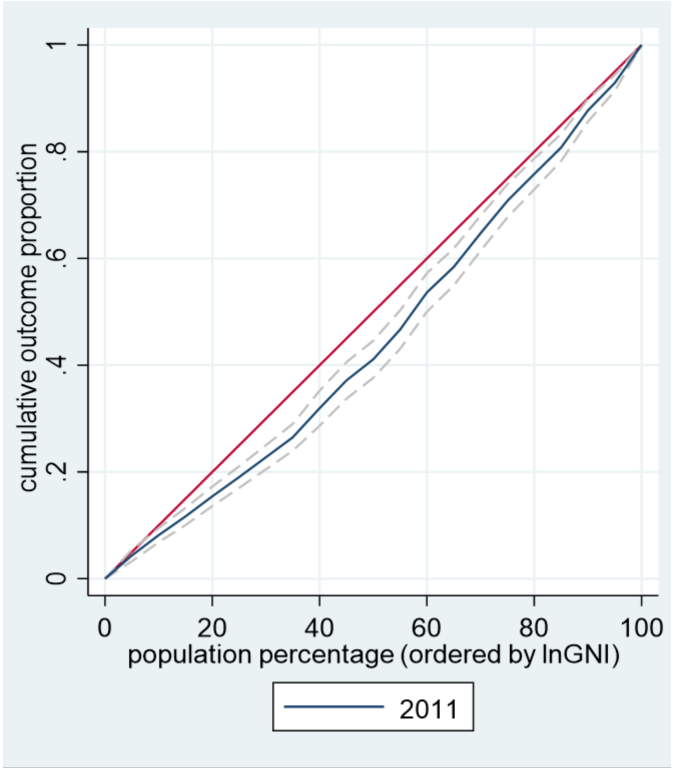

cumulative outcome proportion
0
.2
.4
.6
.8
1
0
20
40
60
80
100
population percentage (ordered by lnGNI)
2011

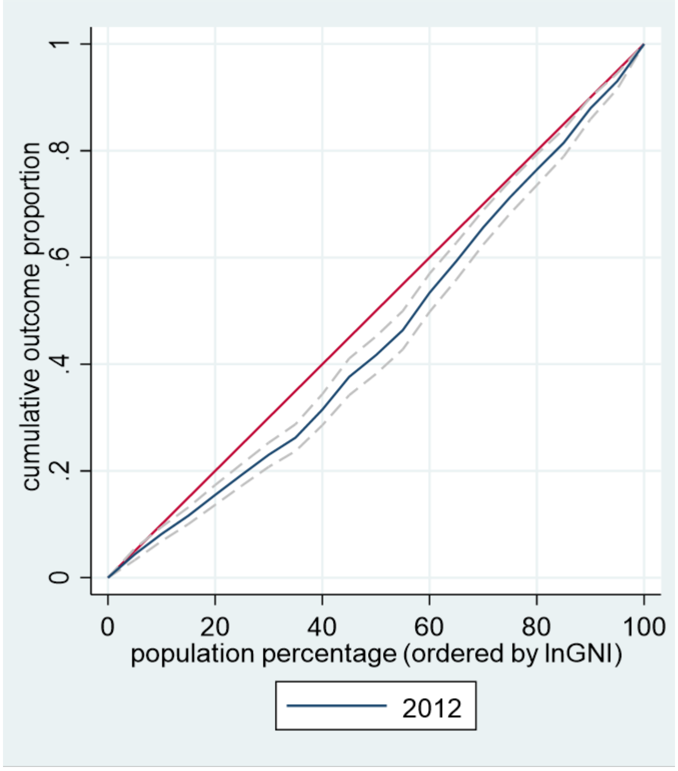

cumulative outcome proportion
0
.2
.4
.6
.8
1
0
20
40
60
80
100
population percentage (ordered by lnGNI)
2012

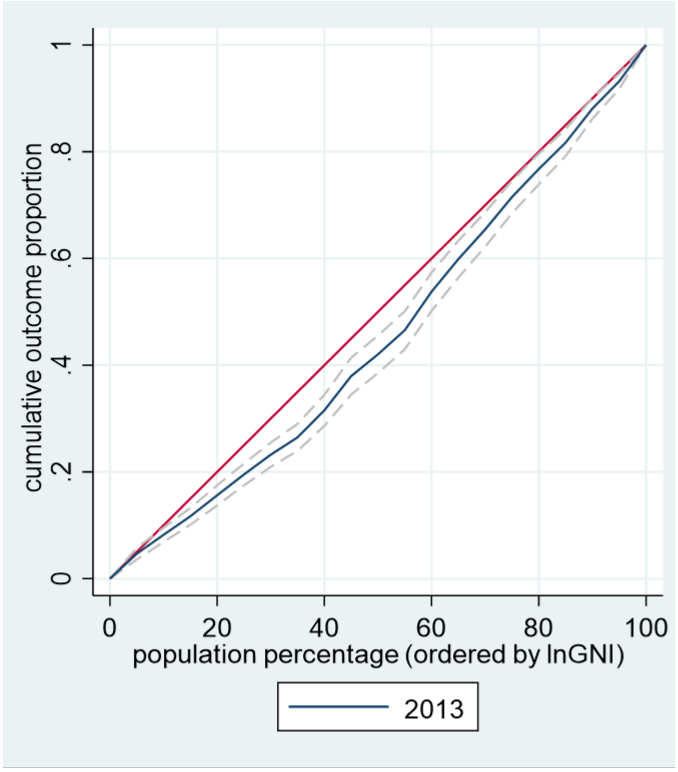

cumulative outcome proportion
0
.2
.4
.6
.8
1
0
20
40
60
80
100
population percentage (ordered by lnGNI)
2013

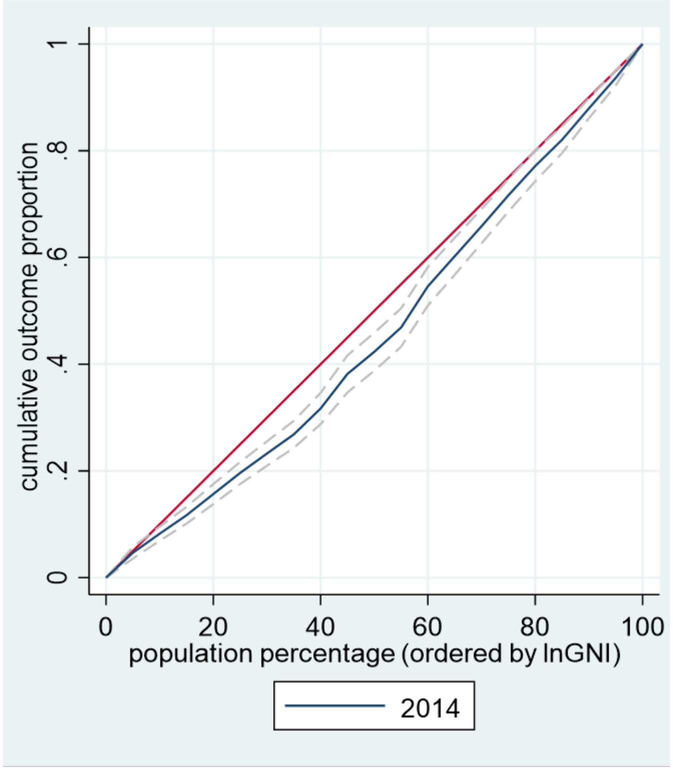

cumulative outcome proportion
0
.2
.4
.6
.8
1
0
20
40
60
80
100
population percentage (ordered by lnGNI)
2014

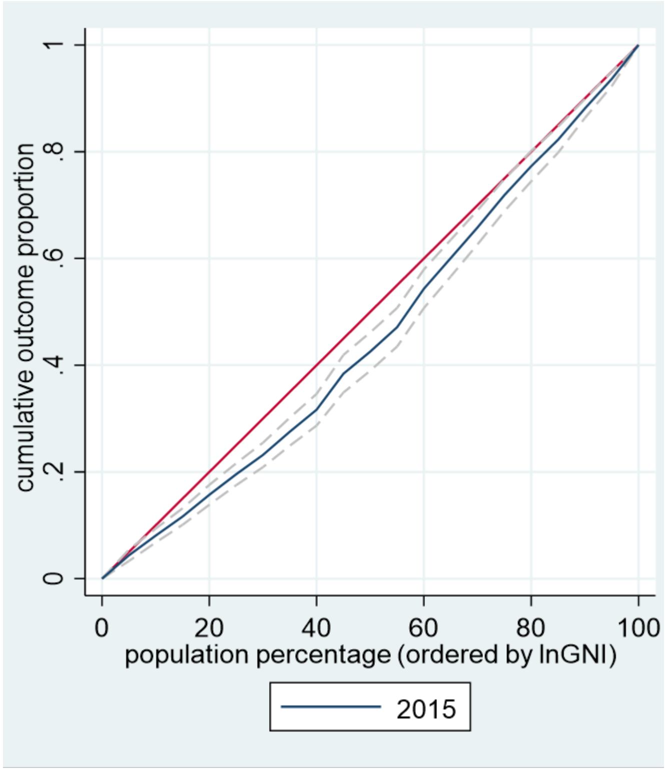
cumulative outcome proportion
0
.2
.4
.6
.8
1
0
20
40
60
80
100
population percentage (ordered by lnGNI)
2015

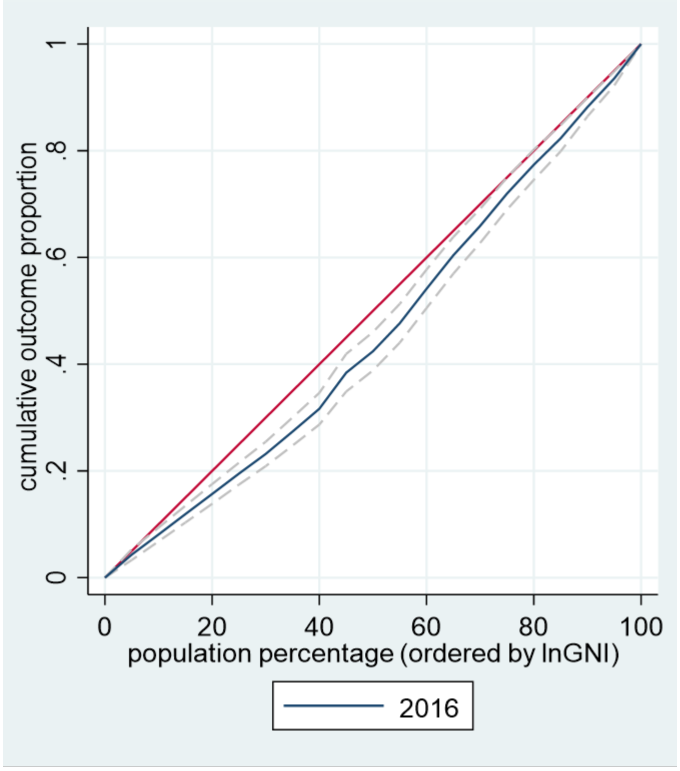
cumulative outcome proportion
0
.2
.4
.6
.8
1
0
20
40
60
80
100
population percentage (ordered by lnGNI)
2016

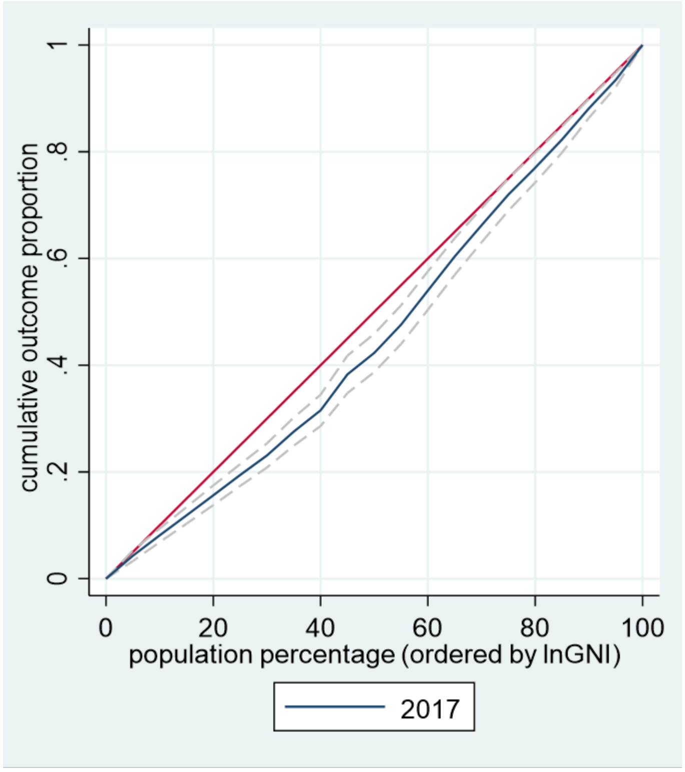
cumulative outcome proportion
0
.2
.4
.6
.8
1
0
20
40
60
80
100
population percentage (ordered by lnGNI)
2017

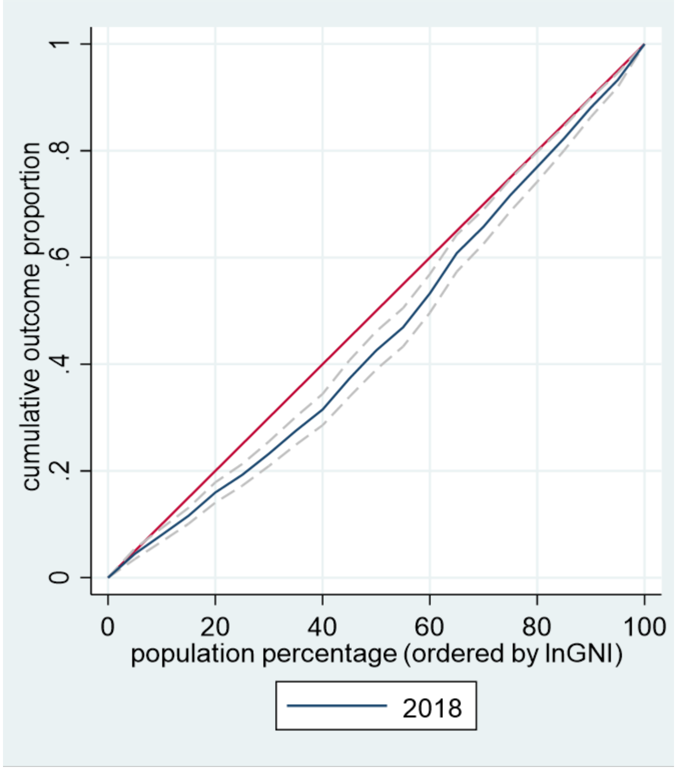
cumulative outcome proportion
0
.2
.4
.6
.8
1
0
20
40
60
80
100
population percentage (ordered by lnGNI)
2018

Figs. S174-191 Concentration curves of age-standardized DALY rates for peripheral artery disease from 2001 to 2018

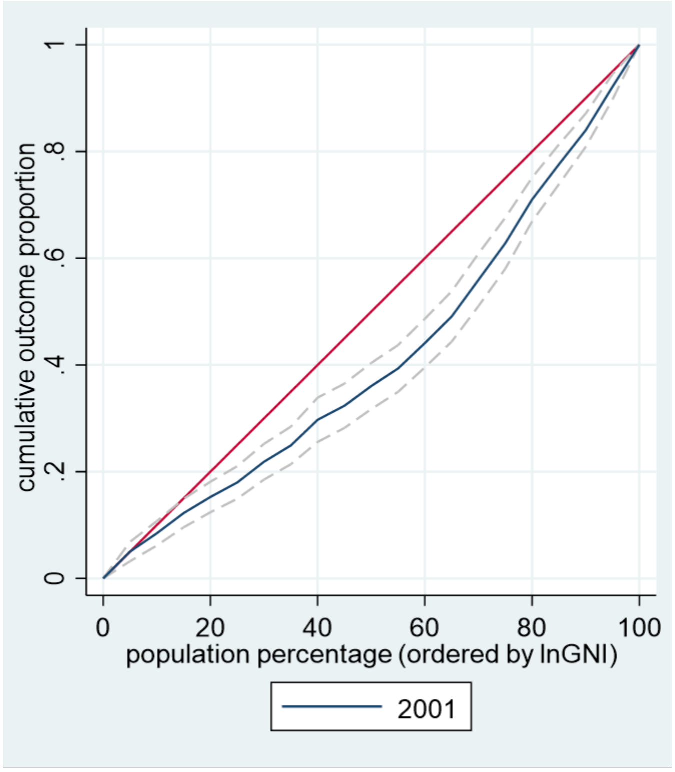

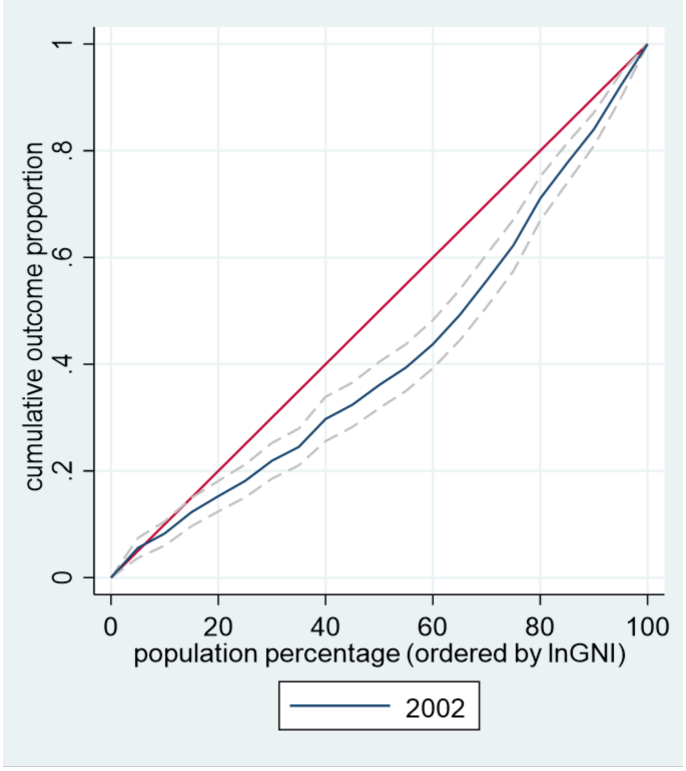

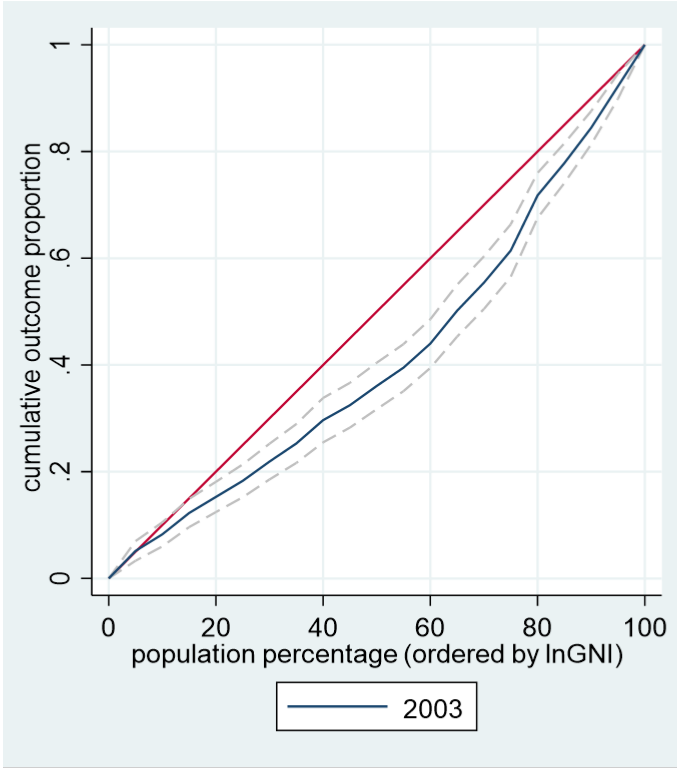
cumulative outcome proportion
0
.2
.4
.6
.8
1
0
20
40
60
80
100
population percentage (ordered by lnGNI)
2003

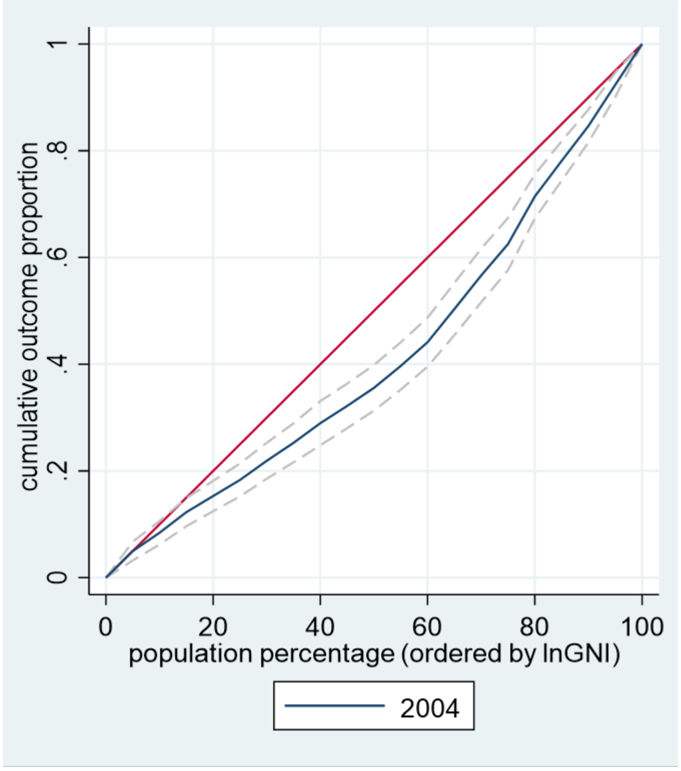
cumulative outcome proportion
0
.2
.4
.6
.8
1
0
20
40
60
80
100
population percentage (ordered by lnGNI)
2004

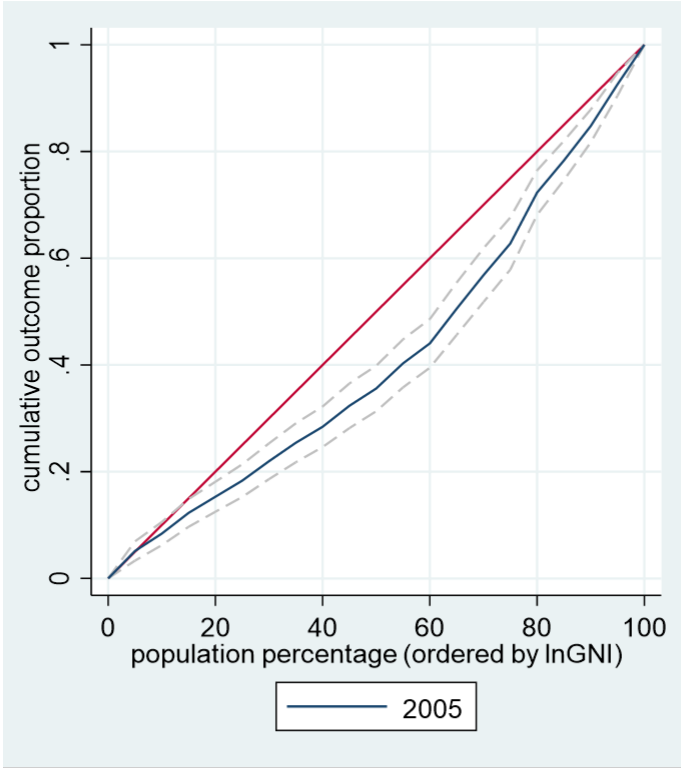
cumulative outcome proportion
0
.2
.4
.6
.8
1
0
20
40
60
80
100
population percentage (ordered by lnGNI)
2005

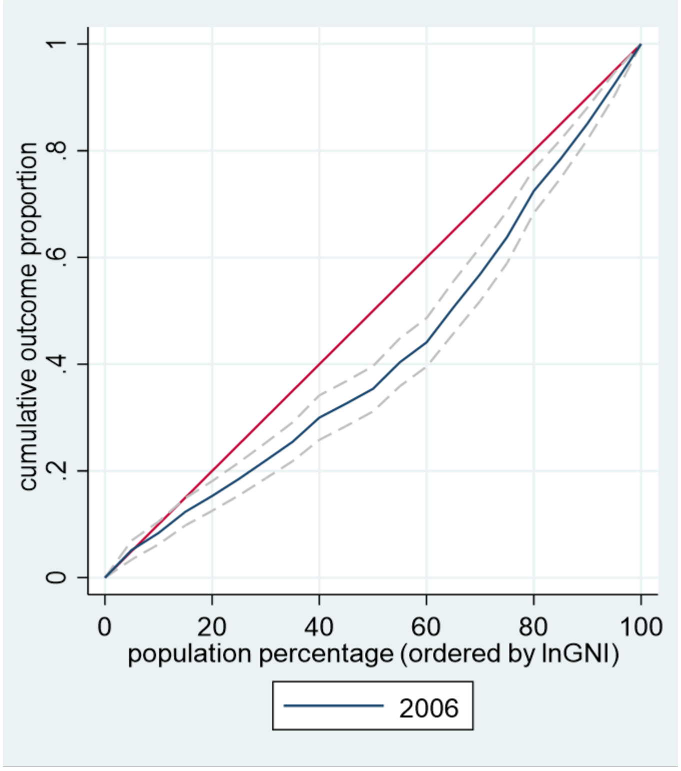
cumulative outcome proportion
0
.2
.4
.6
.8
1
0
20
40
60
80
100
population percentage (ordered by lnGNI)
2006

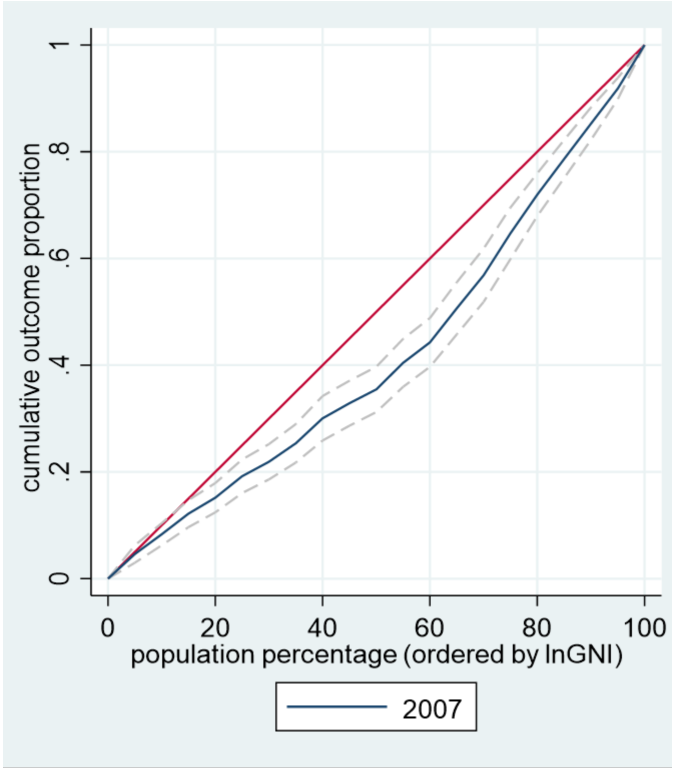
cumulative outcome proportion
0
.2
.4
.6
.8
1
0
20
40
60
80
100
population percentage (ordered by lnGNI)
2007

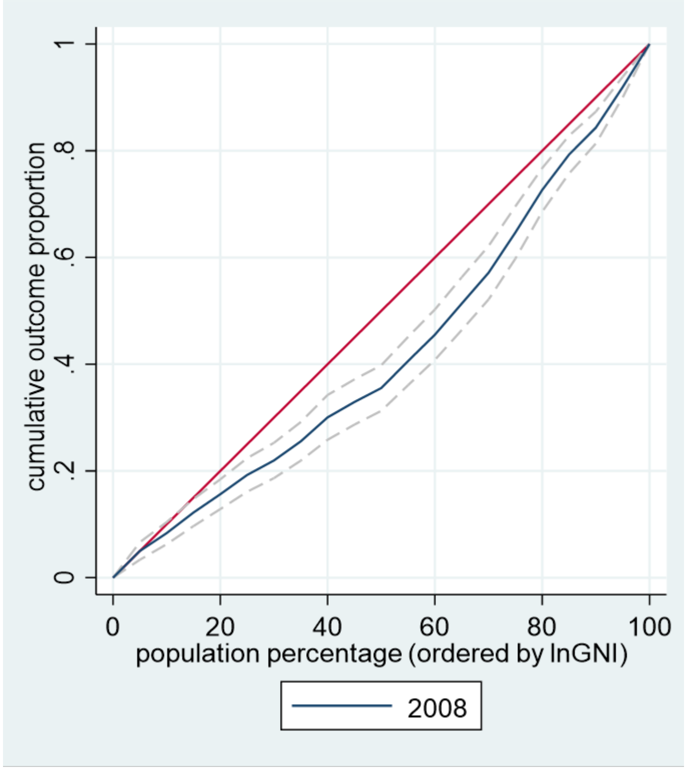
cumulative outcome proportion
0
.2
.4
.6
.8
1
0
20
40
60
80
100
population percentage (ordered by lnGNI)
2008

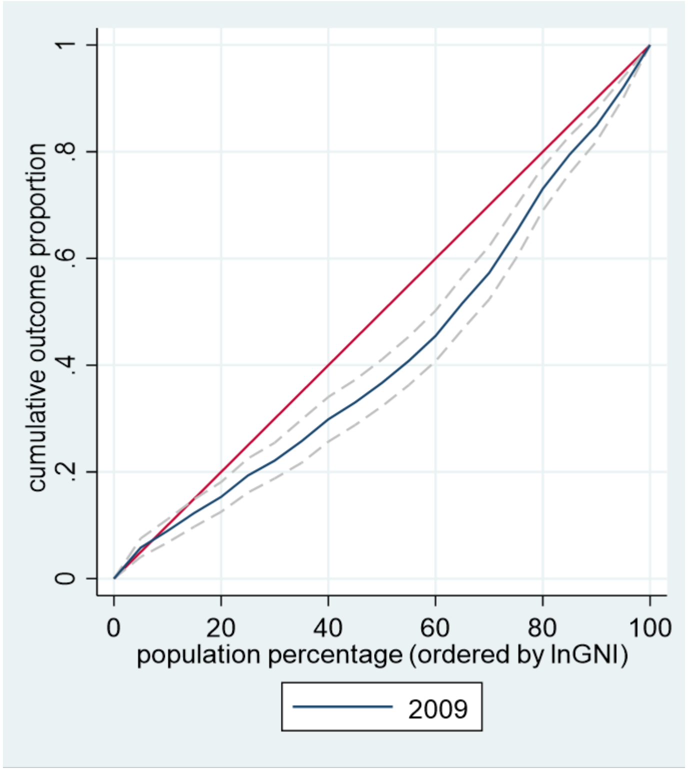

cumulative outcome proportion
0
.2
.4
.6
.8
1
0
20
40
60
80
100
population percentage (ordered by lnGNI)
2009

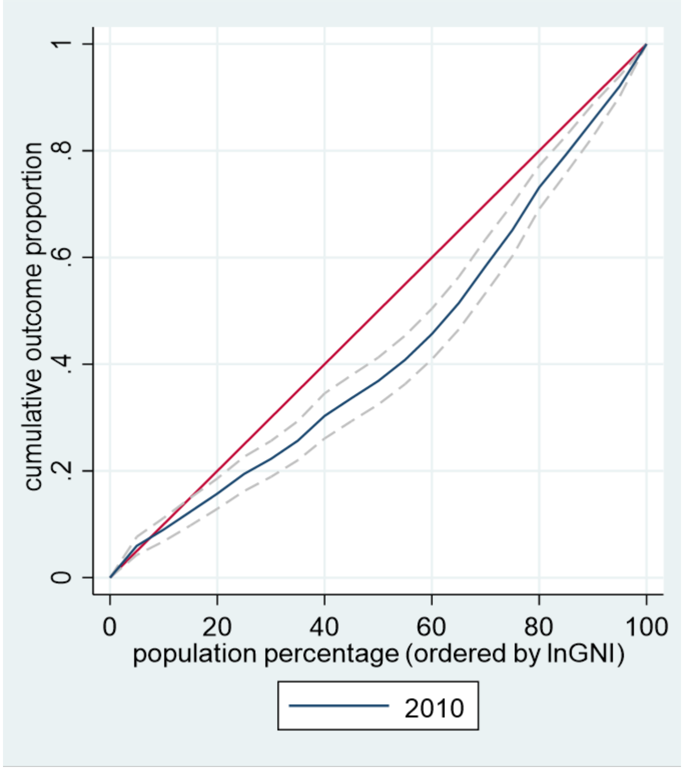

cumulative outcome proportion
0
.2
.4
.6
.8
1
0
20
40
60
80
100
population percentage (ordered by lnGNI)
2010

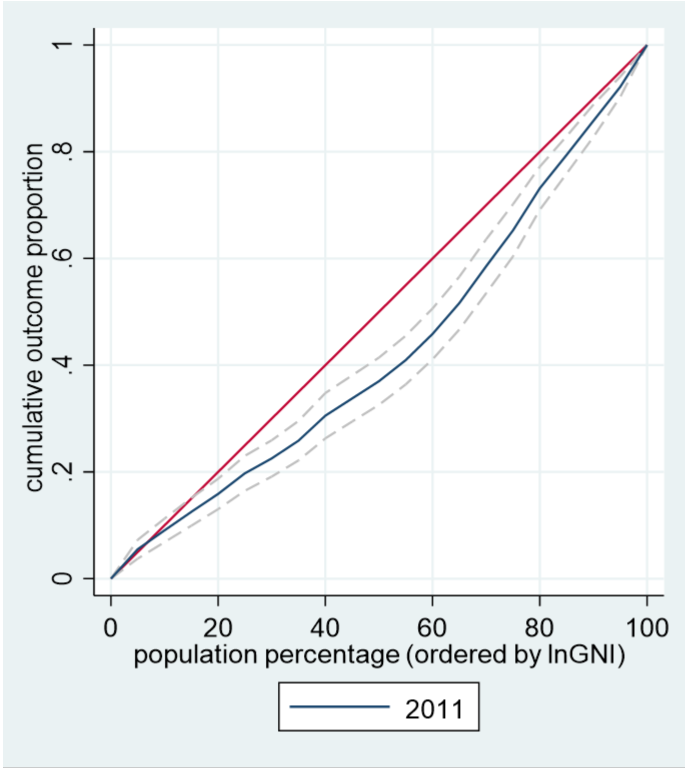
cumulative outcome proportion
0
.2
.4
.6
.8
1
0
20
40
60
80
100
population percentage (ordered by lnGNI)
2011

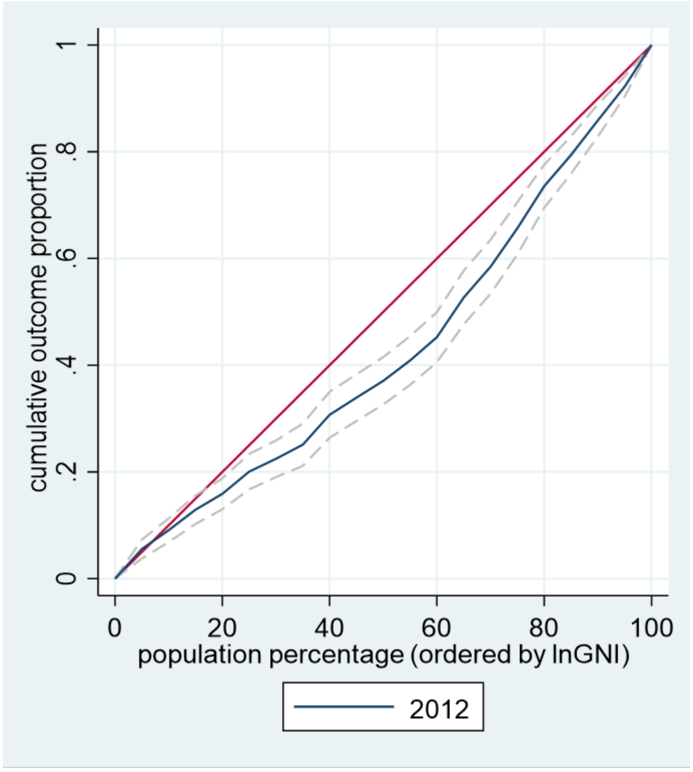
cumulative outcome proportion
0
.2
.4
.6
.8
1
0
20
40
60
80
100
population percentage (ordered by lnGNI)
2012

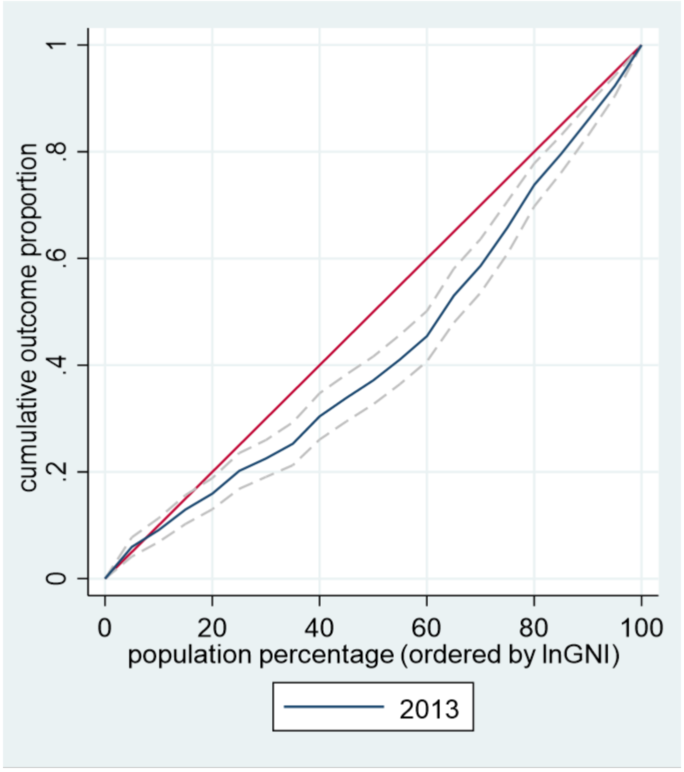
cumulative outcome proportion
0
.2
.4
.6
.8
1
0
20
40
60
80
100
population percentage (ordered by lnGNI)
2013

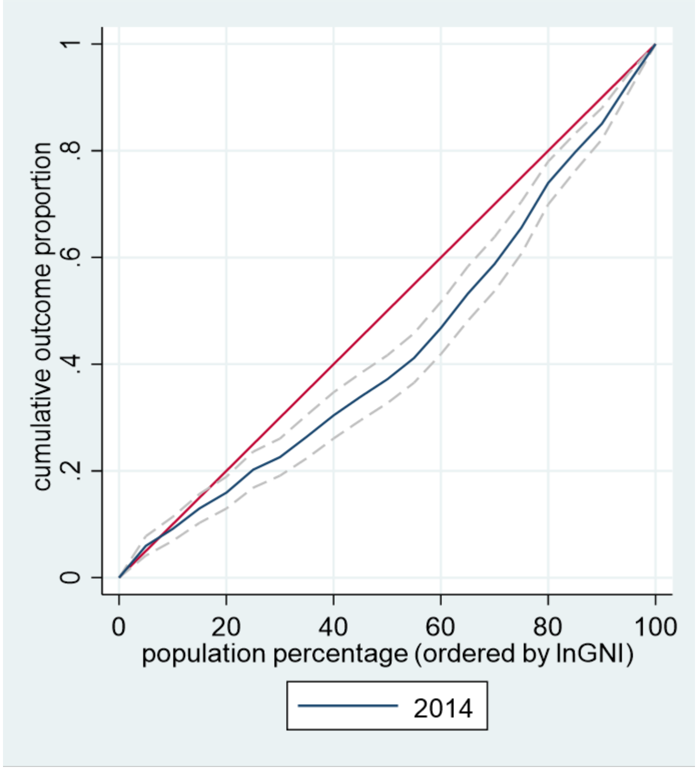
cumulative outcome proportion
0
.2
.4
.6
.8
1
0
20
40
60
80
100
population percentage (ordered by lnGNI)
2014

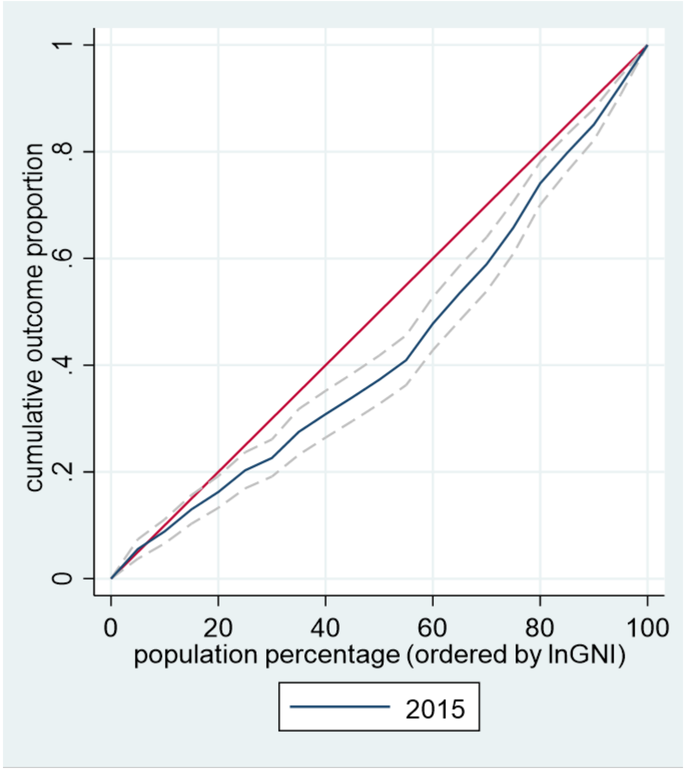
cumulative outcome proportion
0
.2
.4
.6
.8
1
0
20
40
60
80
100
population percentage (ordered by lnGNI)
2015

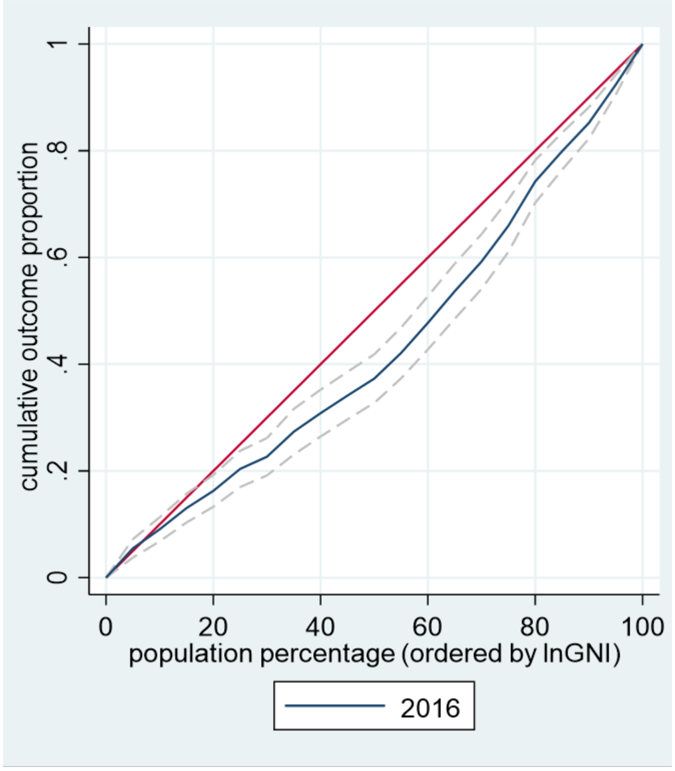
cumulative outcome proportion
0
.2
.4
.6
.8
1
0
20
40
60
80
100
population percentage (ordered by lnGNI)
2016

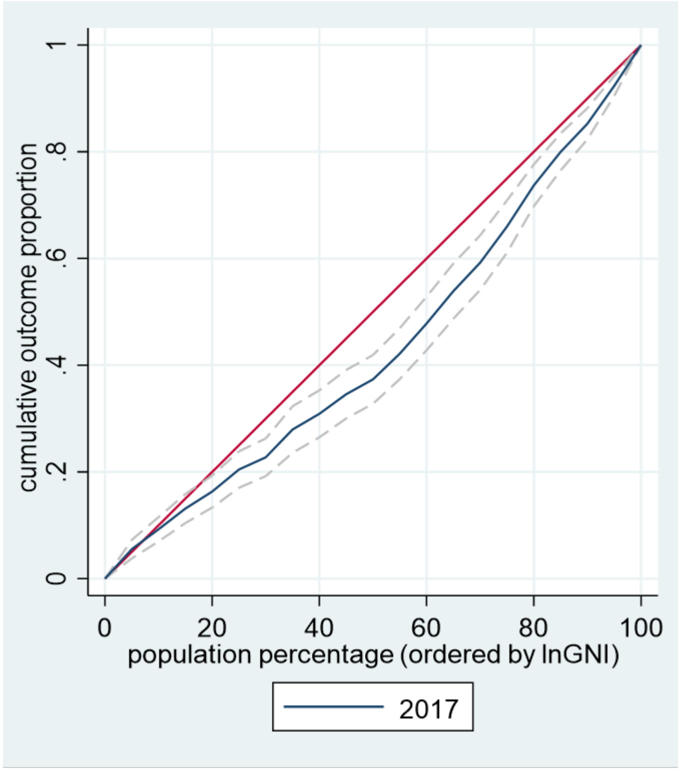

cumulative outcome proportion
0
.2
.4
.6
.8
1
0
20
40
60
80
100
population percentage (ordered by lnGNI)
2017

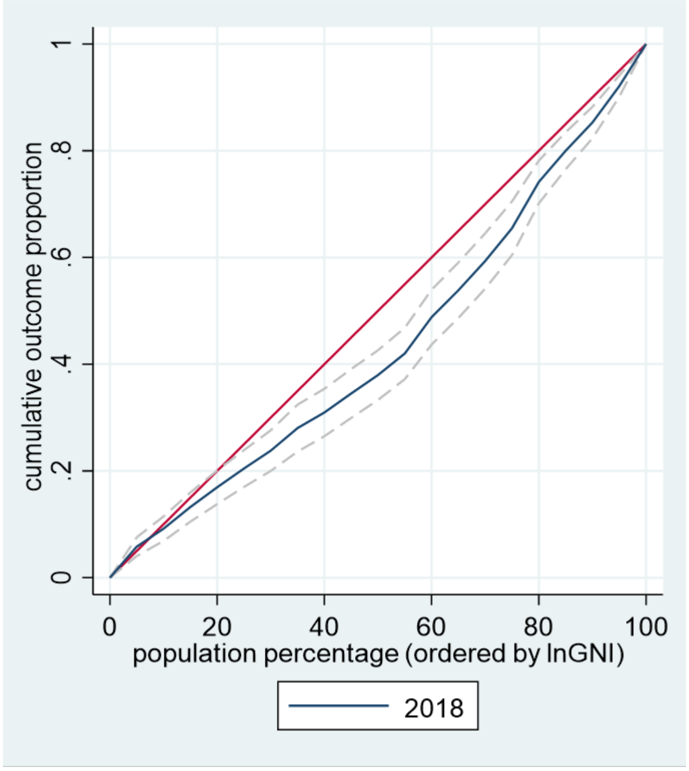

cumulative outcome proportion
0
.2
.4
.6
.8
1
0
20
40
60
80
100
population percentage (ordered by lnGNI)
2018

Figs. S192-209 Concentration curves of age-standardized DALY rates for endocarditis from 2001 to 2018

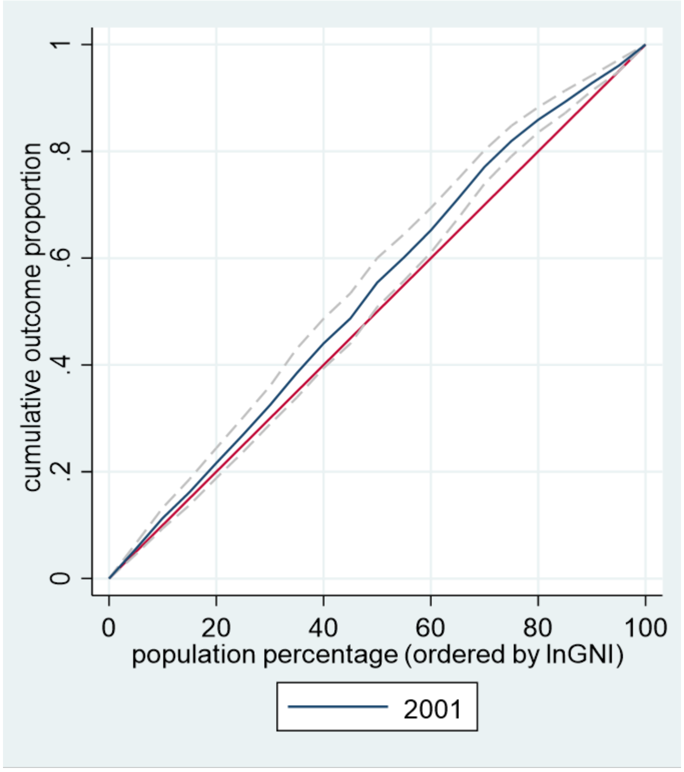

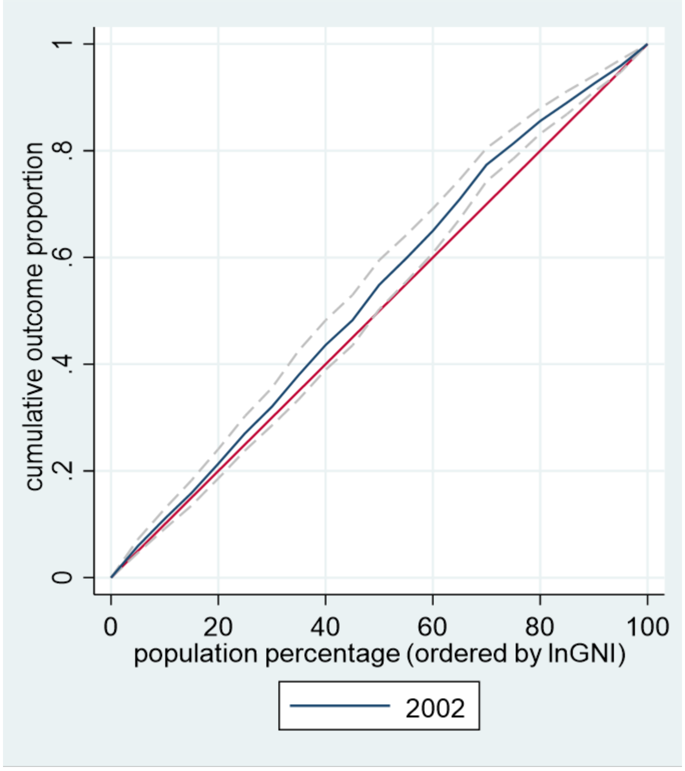

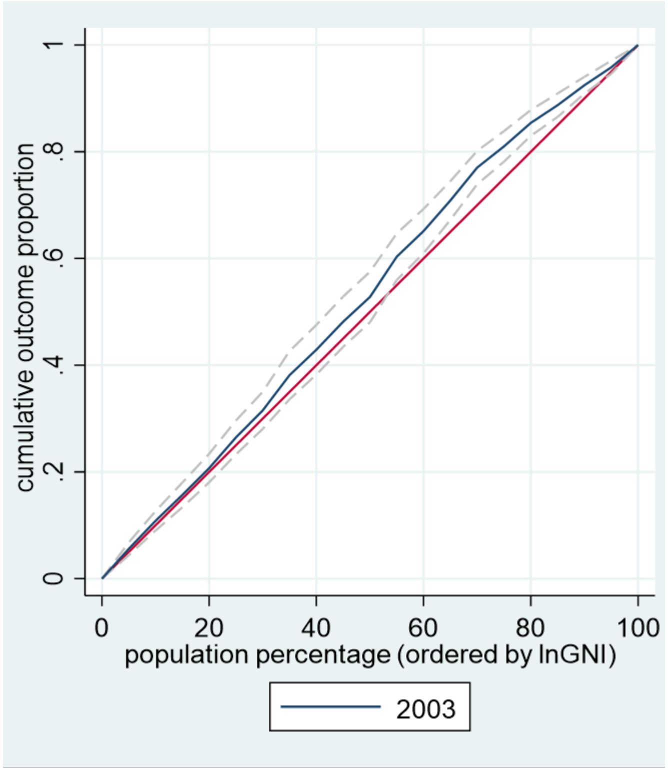
cumulative outcome proportion
0
.2
.4
.6
.8
1
0
20
40
60
80
100
population percentage (ordered by lnGNI)
2003

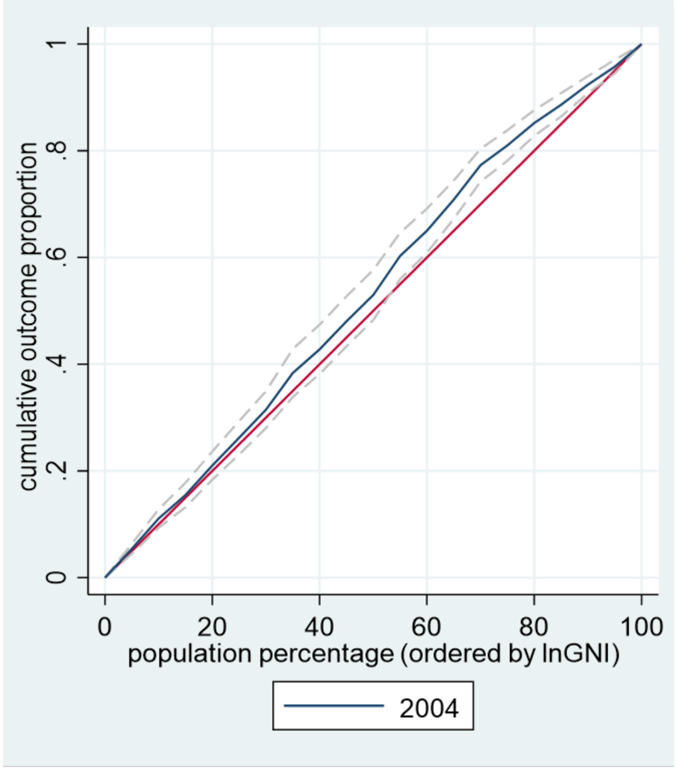
cumulative outcome proportion
0
.2
.4
.6
.8
1
0
20
40
60
80
100
population percentage (ordered by lnGNI)
2004

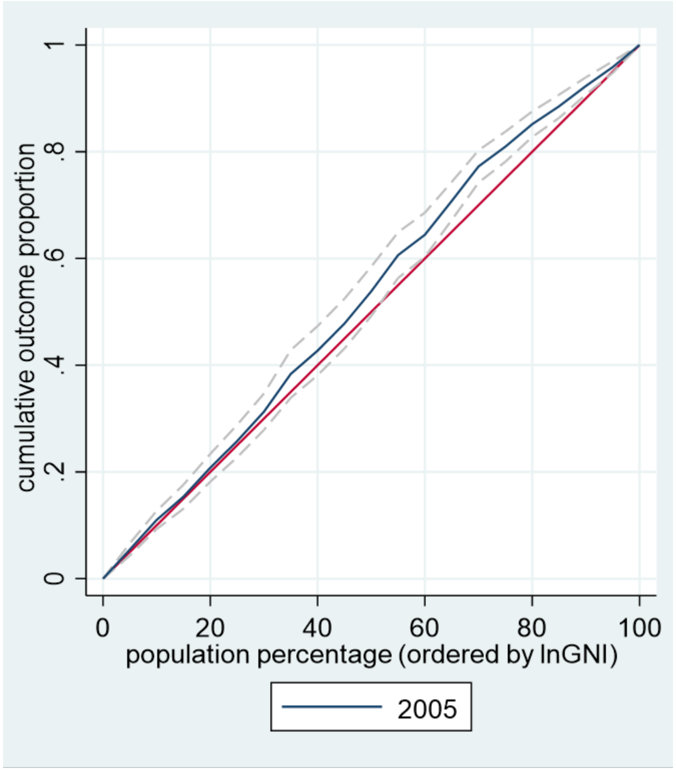
cumulative outcome proportion
0
.2
.4
.6
.8
1
0
20
40
60
80
100
population percentage (ordered by lnGNI)
2005

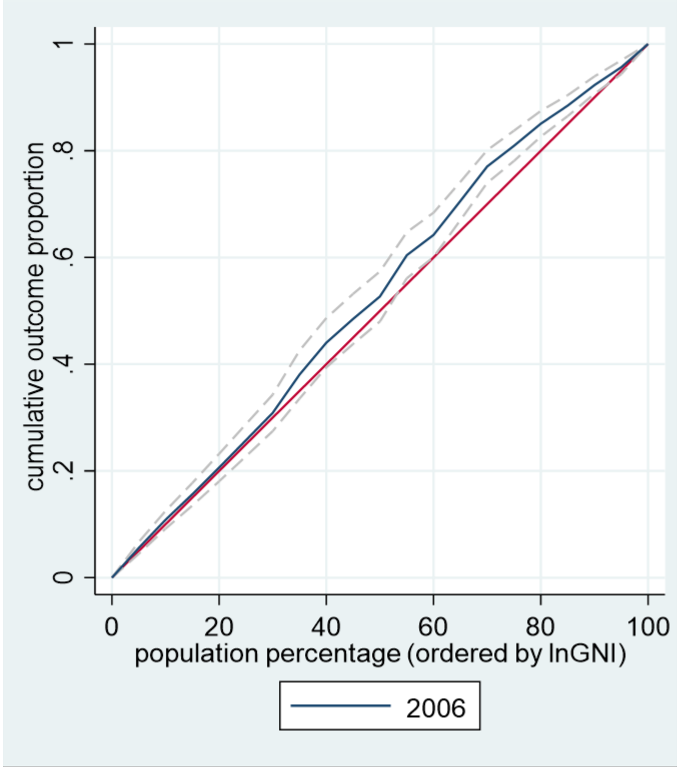
cumulative outcome proportion
0
.2
.4
.6
.8
1
0
20
40
60
80
100
population percentage (ordered by lnGNI)
2006

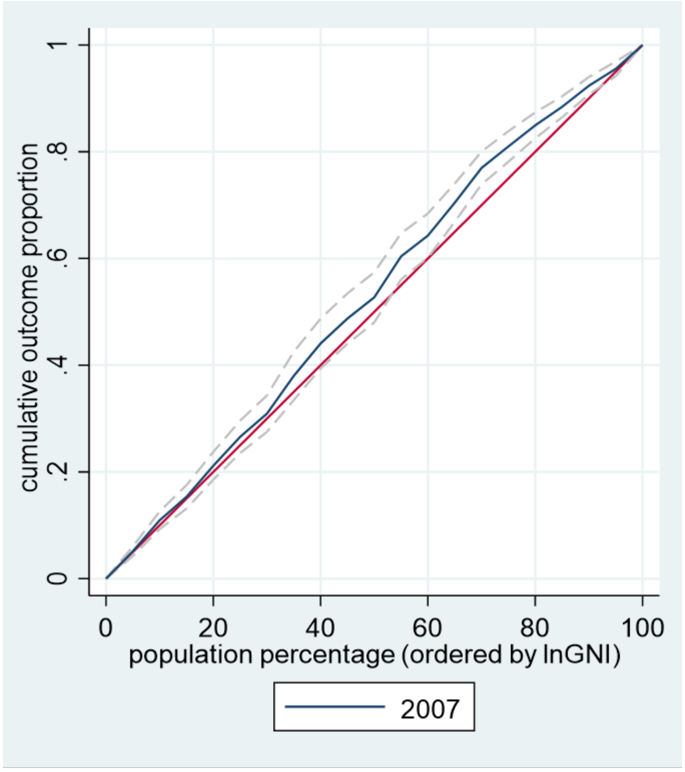

cumulative outcome proportion
0
.2
.4
.6
.8
1
0
20
40
60
80
100
population percentage (ordered by lnGNI)
2007

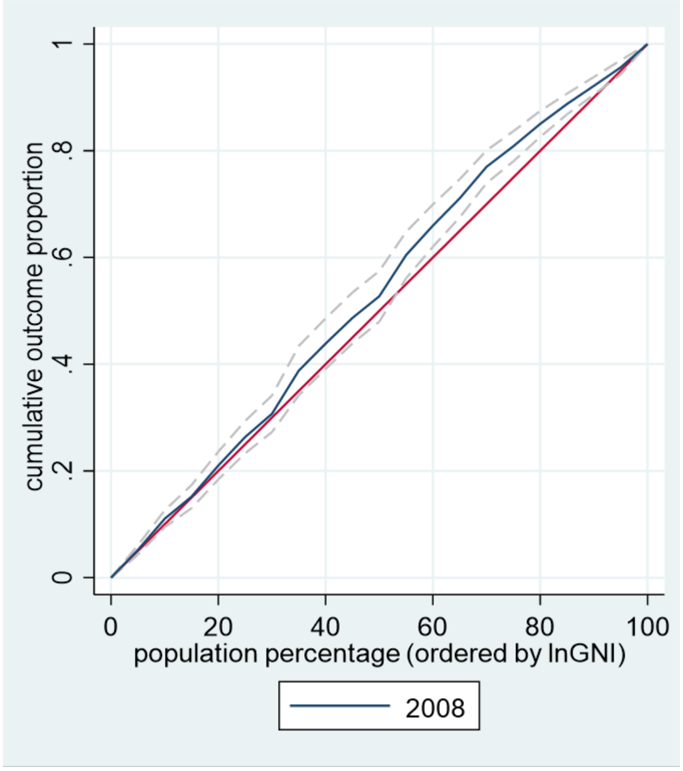

cumulative outcome proportion
0
.2
.4
.6
.8
1
0
20
40
60
80
100
population percentage (ordered by lnGNI)
2008

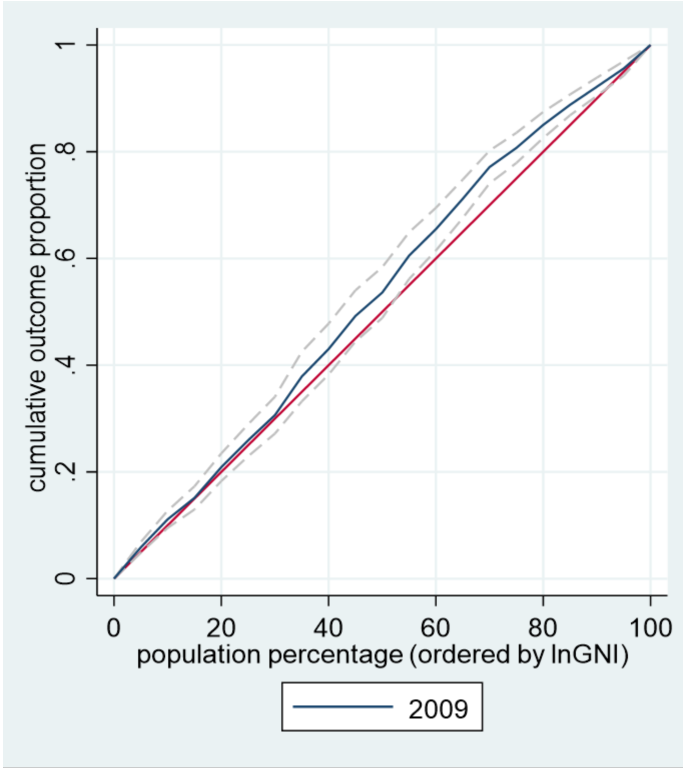
cumulative outcome proportion
0
.2
.4
.6
.8
1
0
20
40
60
80
100
population percentage (ordered by lnGNI)
2009

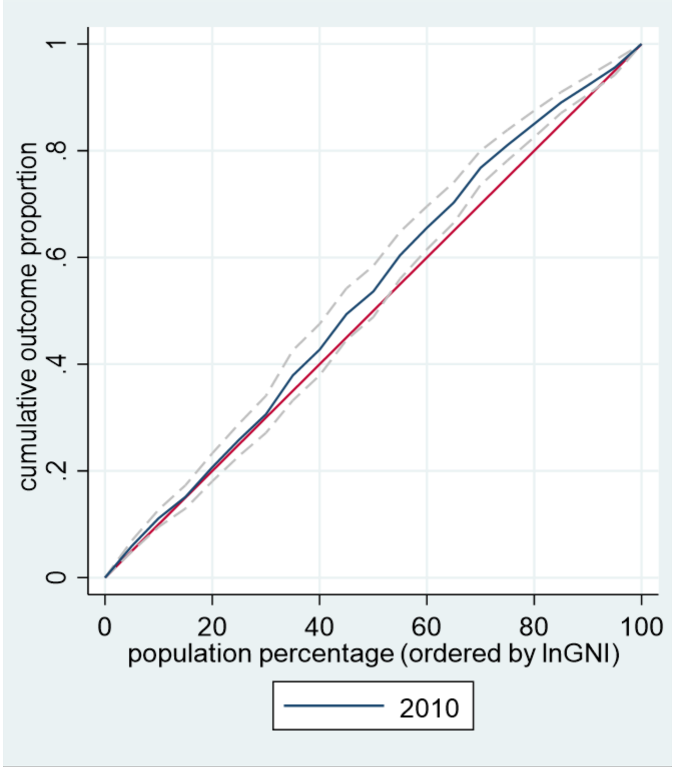
cumulative outcome proportion
0
.2
.4
.6
.8
1
0
20
40
60
80
100
population percentage (ordered by lnGNI)
2010

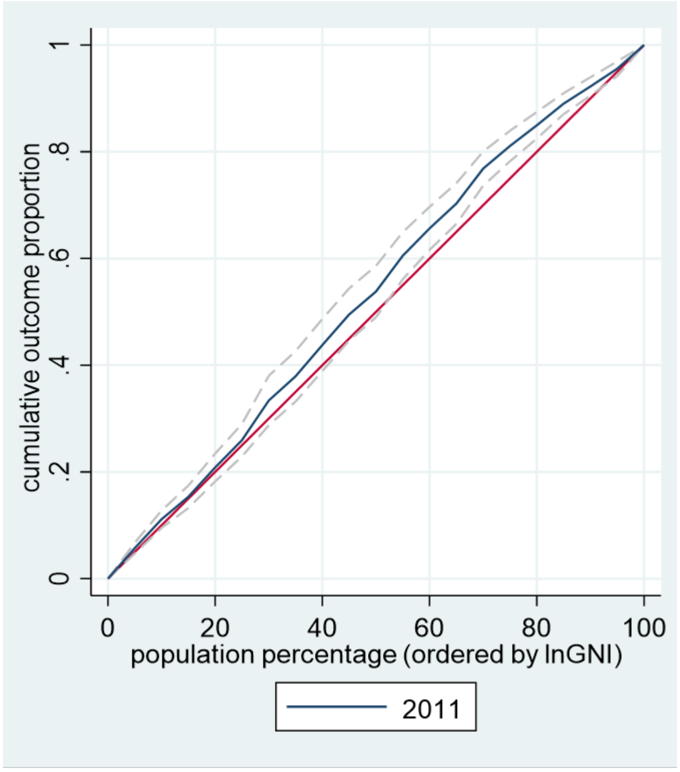
cumulative outcome proportion
0
.2
.4
.6
.8
1
0
20
40
60
80
100
population percentage (ordered by lnGNI)
2011

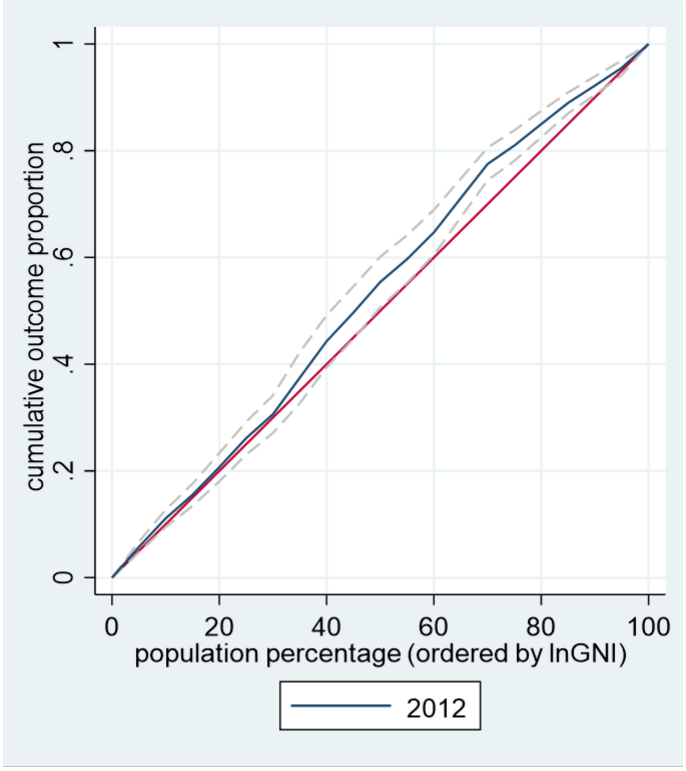
cumulative outcome proportion
0
.2
.4
.6
.8
1
0
20
40
60
80
100
population percentage (ordered by lnGNI)
2012

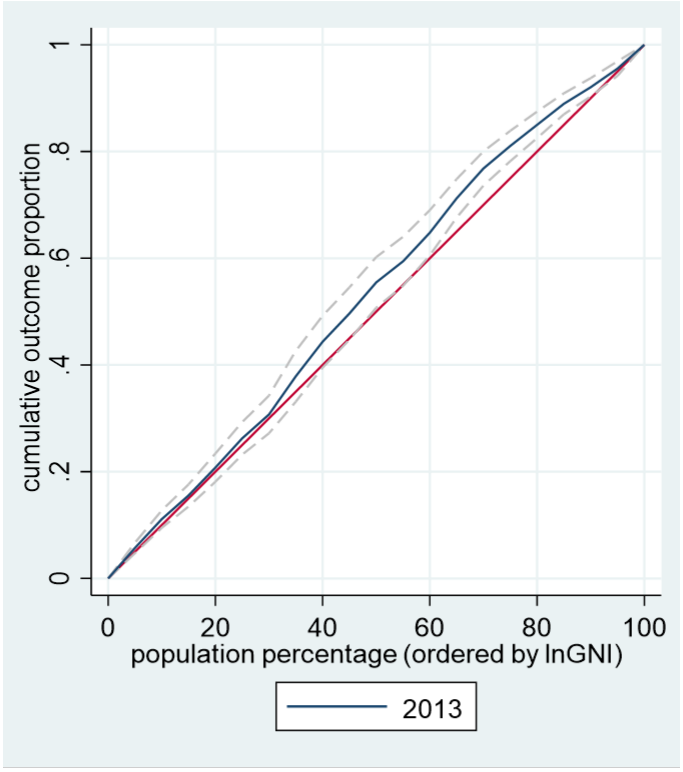

cumulative outcome proportion
0
.2
.4
.6
.8
1
0
20
40
60
80
100
population percentage (ordered by lnGNI)
2013

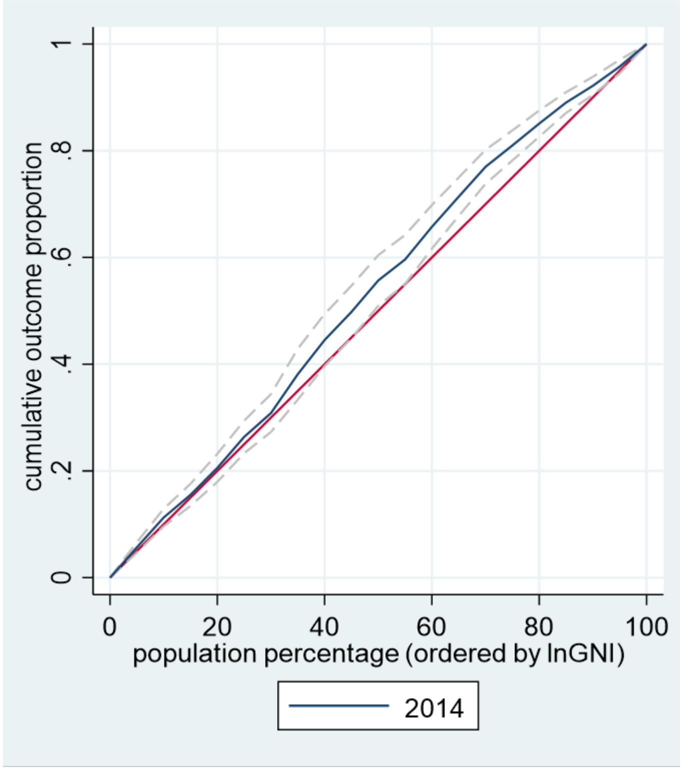

cumulative outcome proportion
0
.2
.4
.6
.8
1
0
20
40
60
80
100
population percentage (ordered by lnGNI)
2014

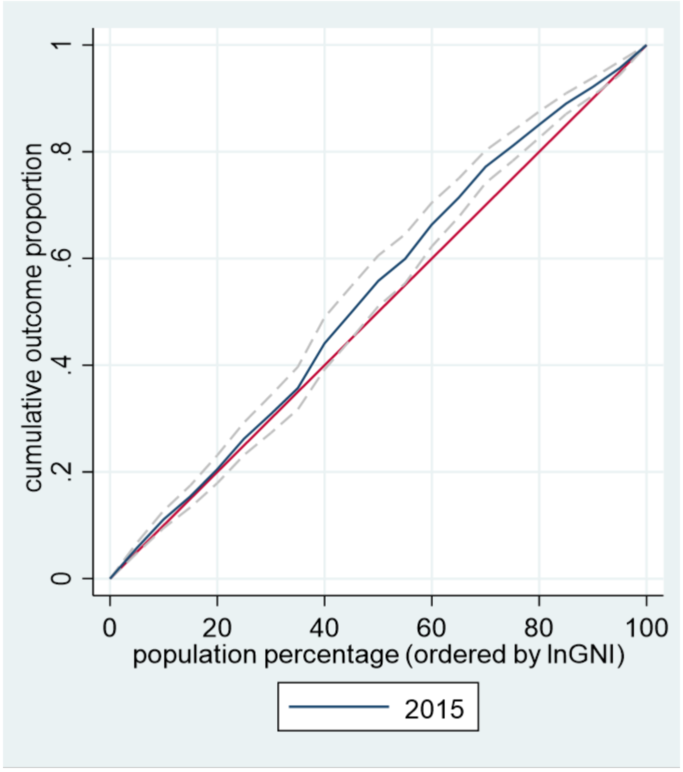
cumulative outcome proportion
0
.2
.4
.6
.8
1
0
20
40
60
80
100
population percentage (ordered by lnGNI)
2015

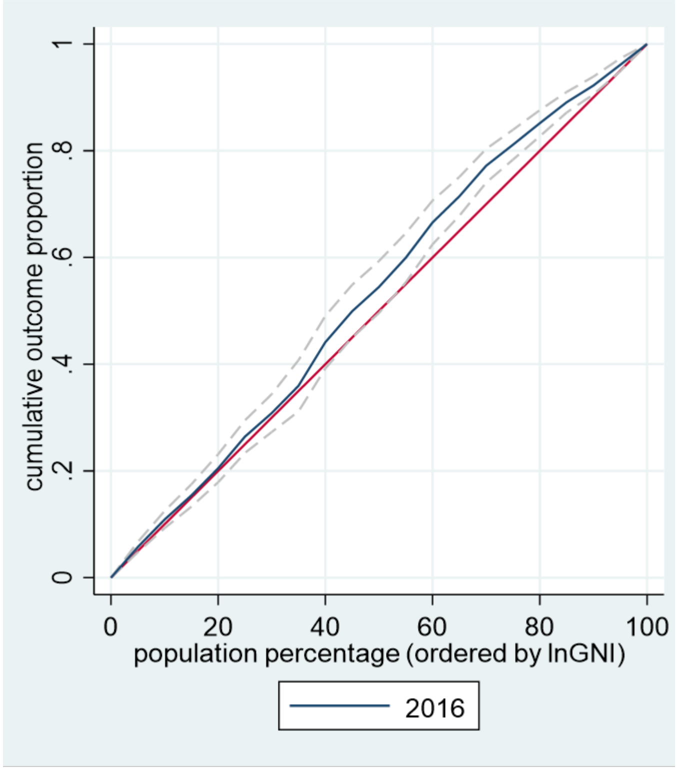
cumulative outcome proportion
0
.2
.4
.6
.8
1
0
20
40
60
80
100
population percentage (ordered by lnGNI)
2016

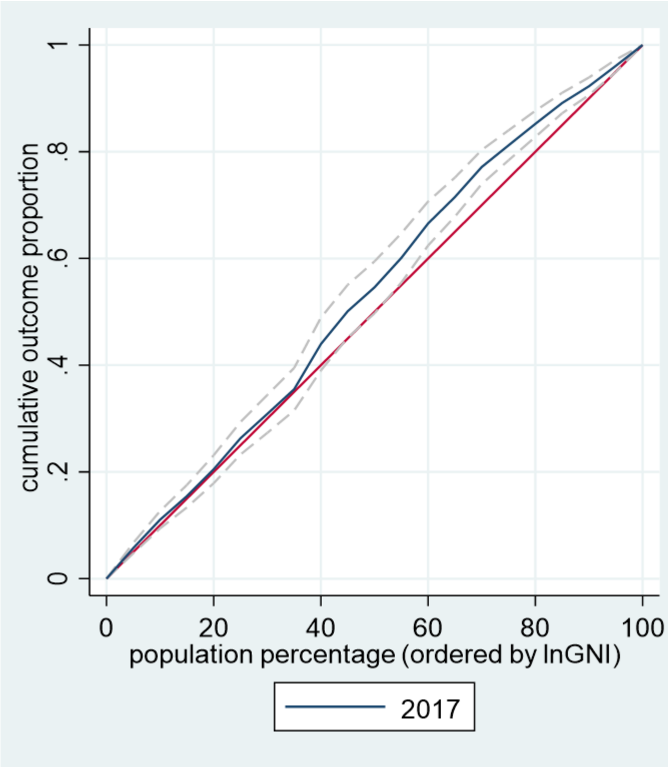

cumulative outcome proportion
0
.2
.4
.6
.8
1
0
20
40
60
80
100
population percentage (ordered by lnGNI)
2017

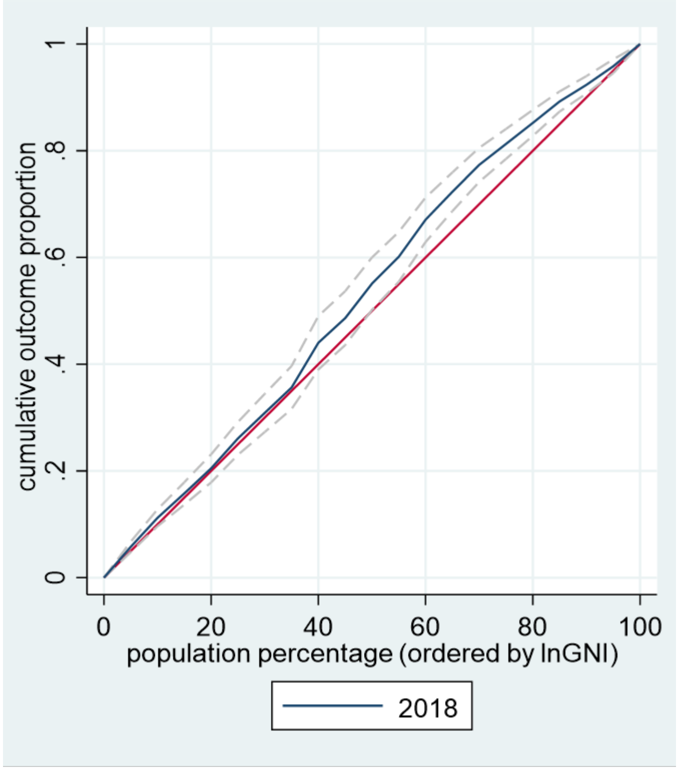

cumulative outcome proportion
0
.2
.4
.6
.8
1
0
20
40
60
80
100
population percentage (ordered by lnGNI)
2018

Figs. S210-227 Concentration curves of age-standardized DALY rates for other cardiovascular and circulatory diseases from 2001 to 2018

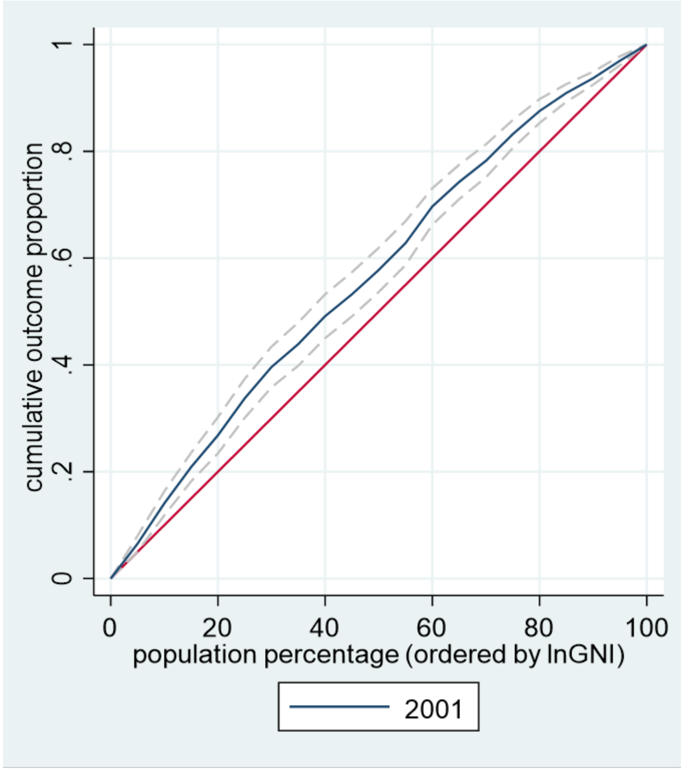

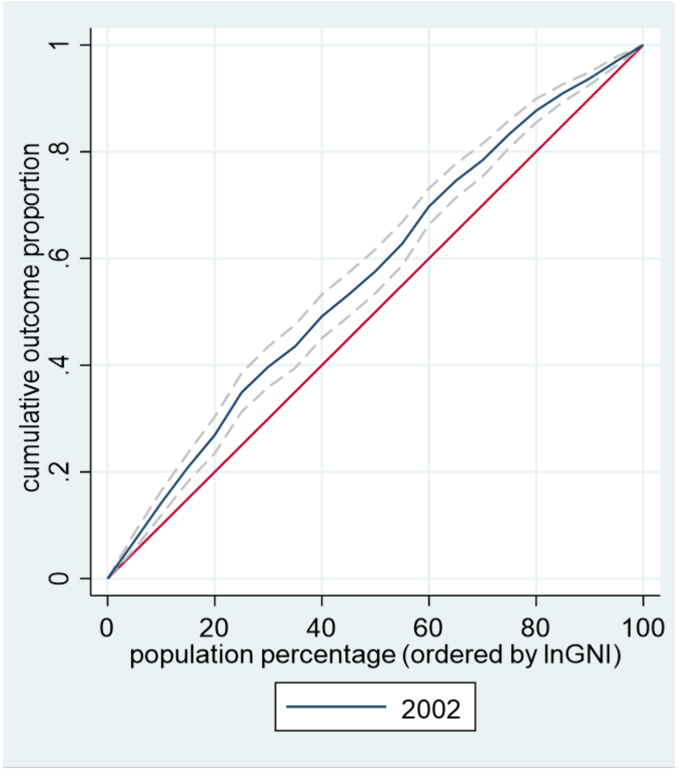

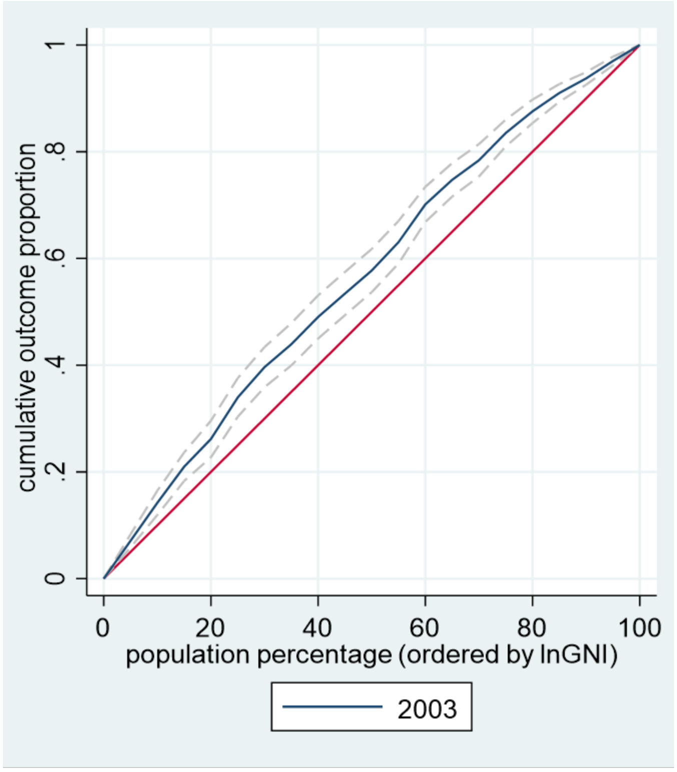

cumulative outcome proportion
0
.2
.4
.6
.8
1
0
20
40
60
80
100
population percentage (ordered by lnGNI)
2003

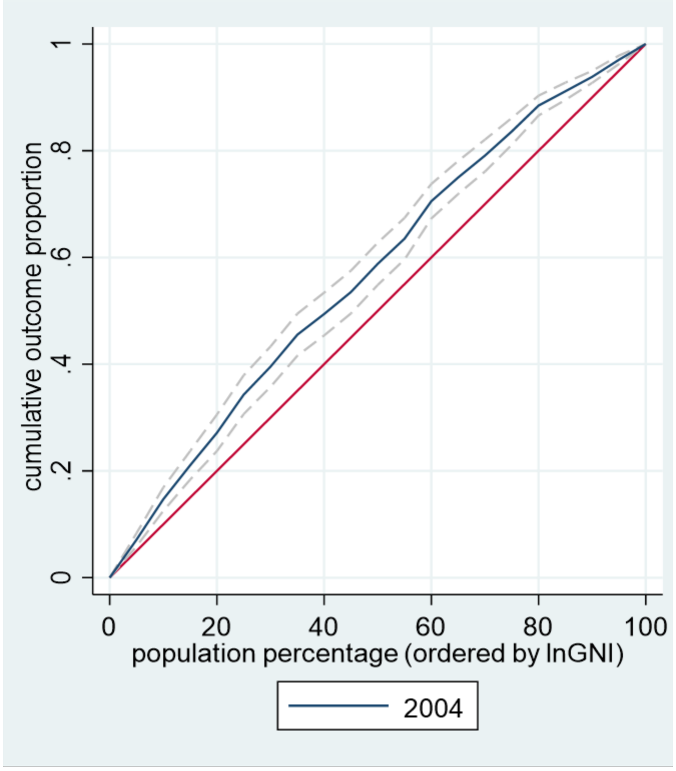

cumulative outcome proportion
0
.2
.4
.6
.8
1
0
20
40
60
80
100
population percentage (ordered by lnGNI)
2004

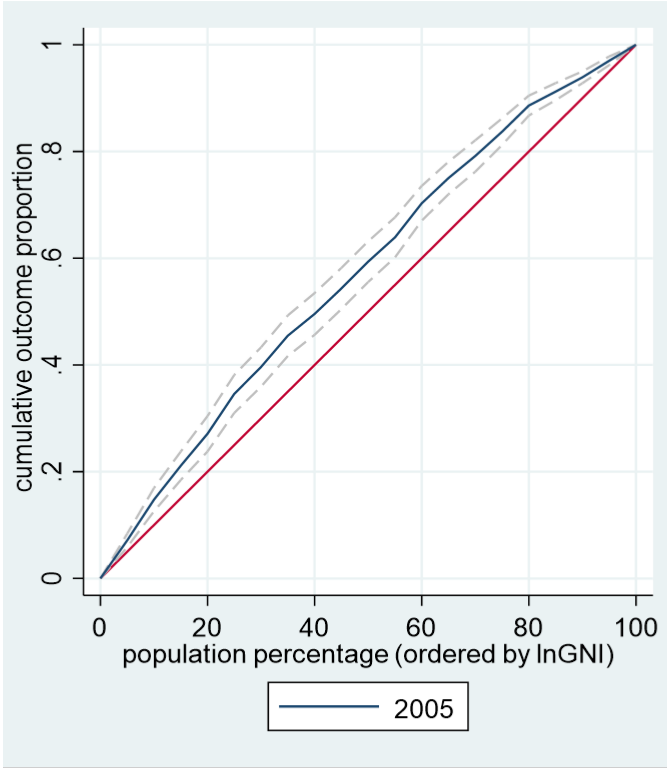

cumulative outcome proportion
0
.2
.4
.6
.8
1
0
20
40
60
80
100
population percentage (ordered by lnGNI)
2005

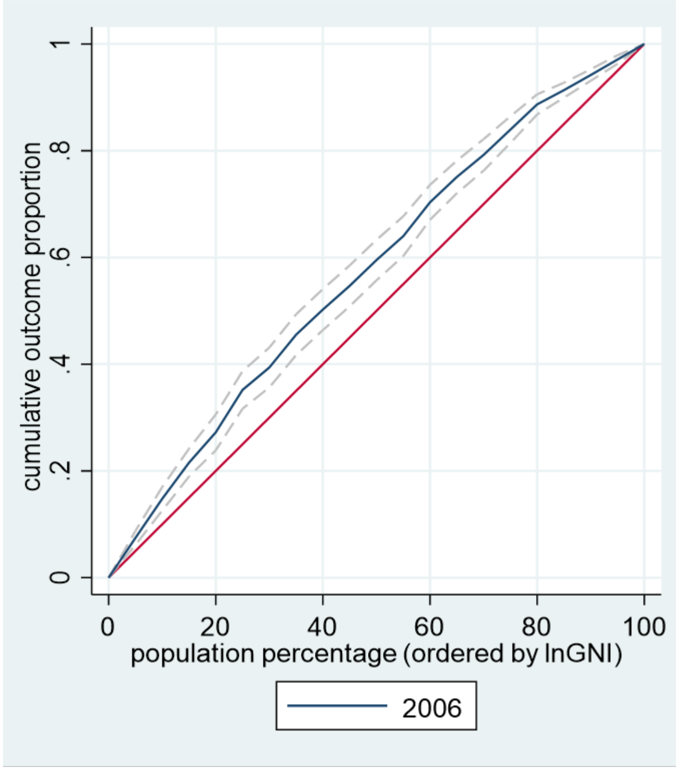

cumulative outcome proportion
0
.2
.4
.6
.8
1
0
20
40
60
80
100
population percentage (ordered by lnGNI)
2006

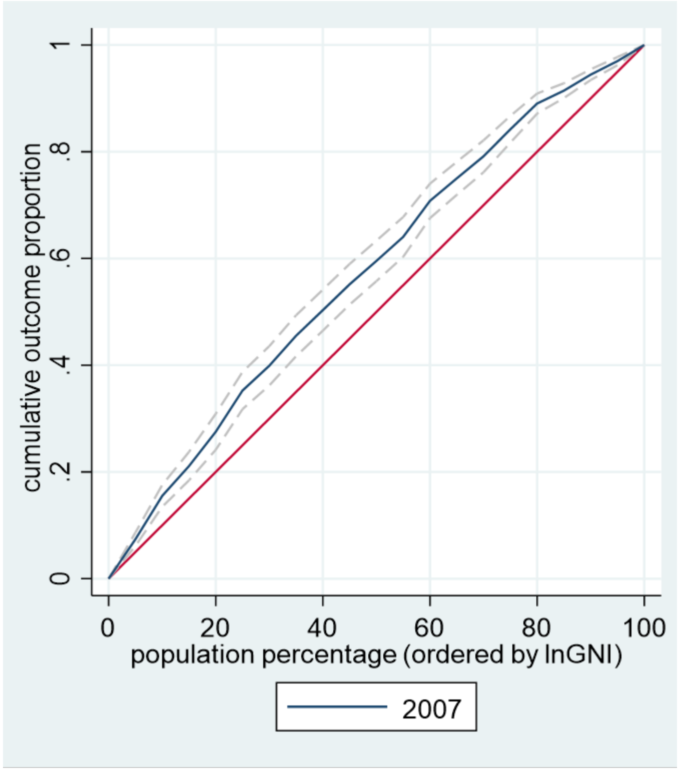
cumulative outcome proportion
0
.2
.4
.6
.8
1
0
20
40
60
80
100
population percentage (ordered by lnGNI)
2007

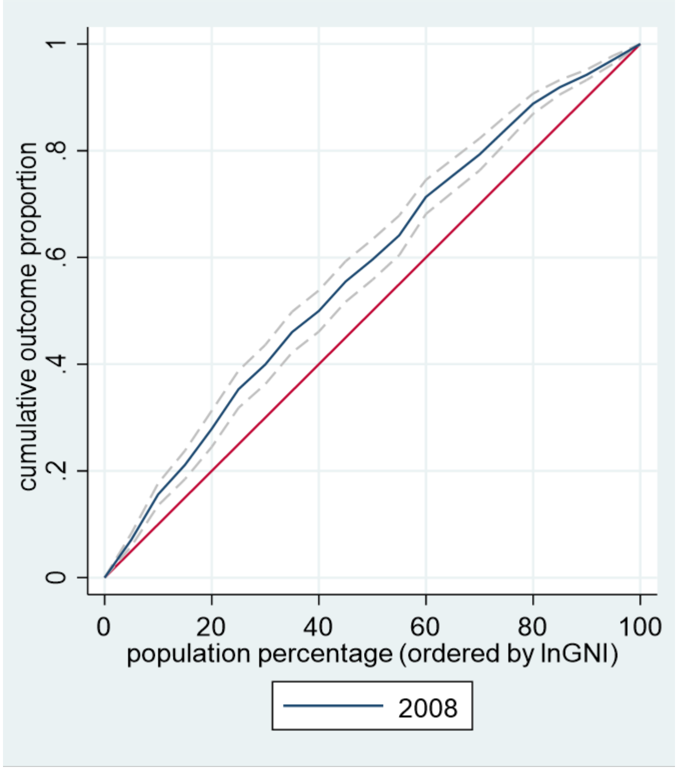
cumulative outcome proportion
0
.2
.4
.6
.8
1
0
20
40
60
80
100
population percentage (ordered by lnGNI)
2008

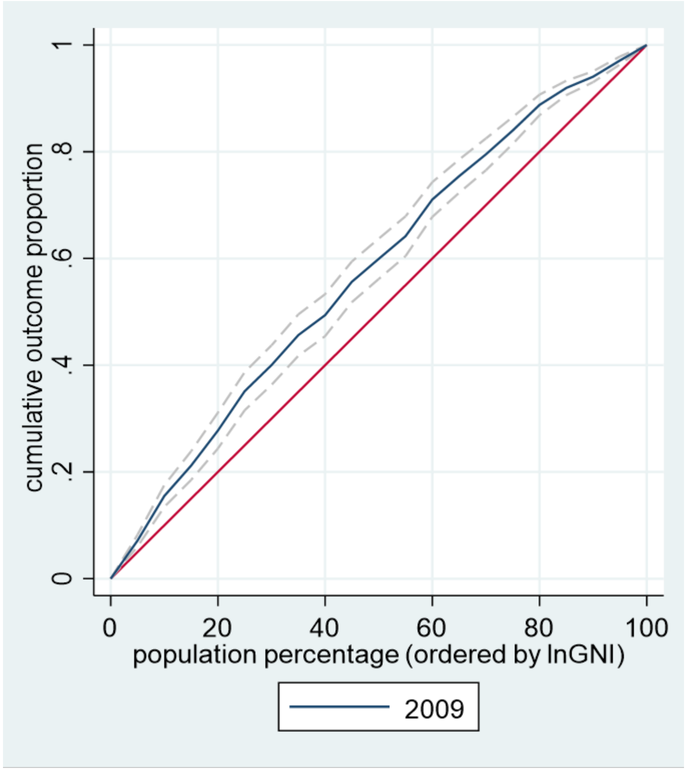
cumulative outcome proportion
0
.2
.4
.6
.8
1
0
20
40
60
80
100
population percentage (ordered by lnGNI)
2009

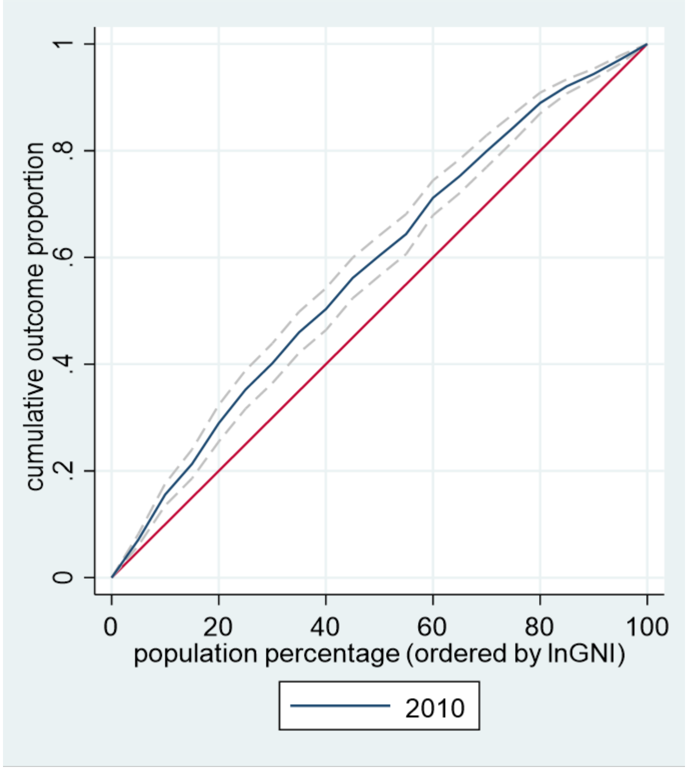
cumulative outcome proportion
0
.2
.4
.6
.8
1
0
20
40
60
80
100
population percentage (ordered by lnGNI)
2010

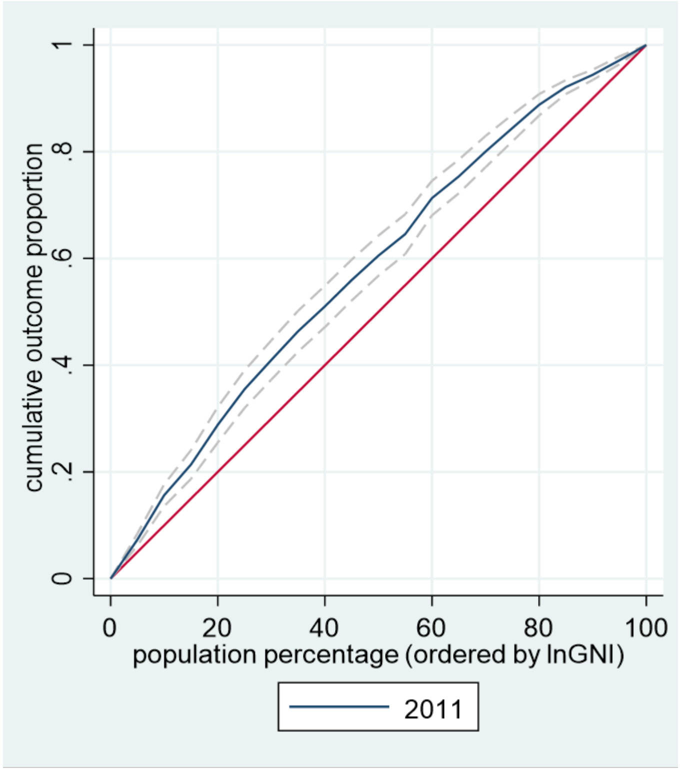
cumulative outcome proportion
0
.2
.4
.6
.8
1
0
20
40
60
80
100
population percentage (ordered by lnGNI)
2011

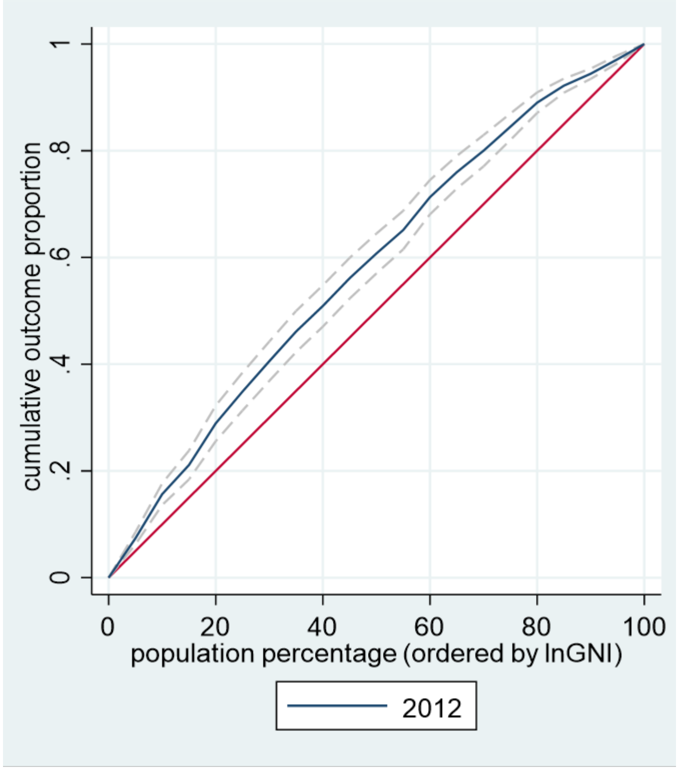
cumulative outcome proportion
0
.2
.4
.6
.8
1
0
20
40
60
80
100
population percentage (ordered by lnGNI)
2012

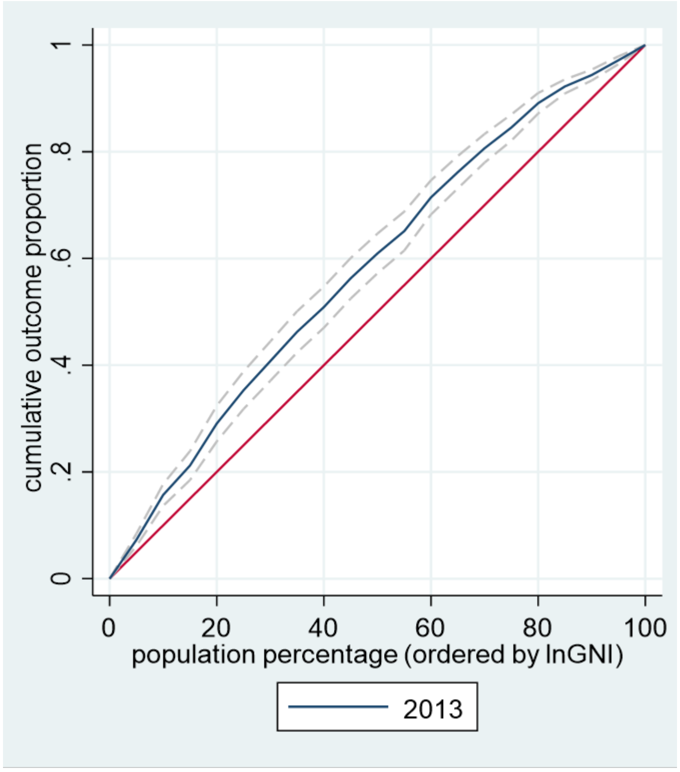
cumulative outcome proportion
0
.2
.4
.6
.8
1
0
20
40
60
80
100
population percentage (ordered by lnGNI)
2013

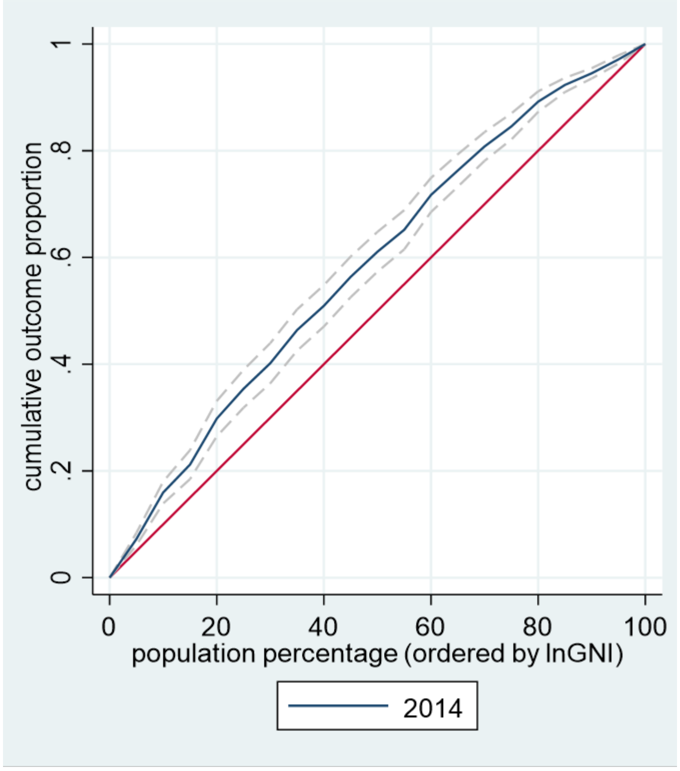
cumulative outcome proportion
0
.2
.4
.6
.8
1
0
20
40
60
80
100
population percentage (ordered by lnGNI)
2014

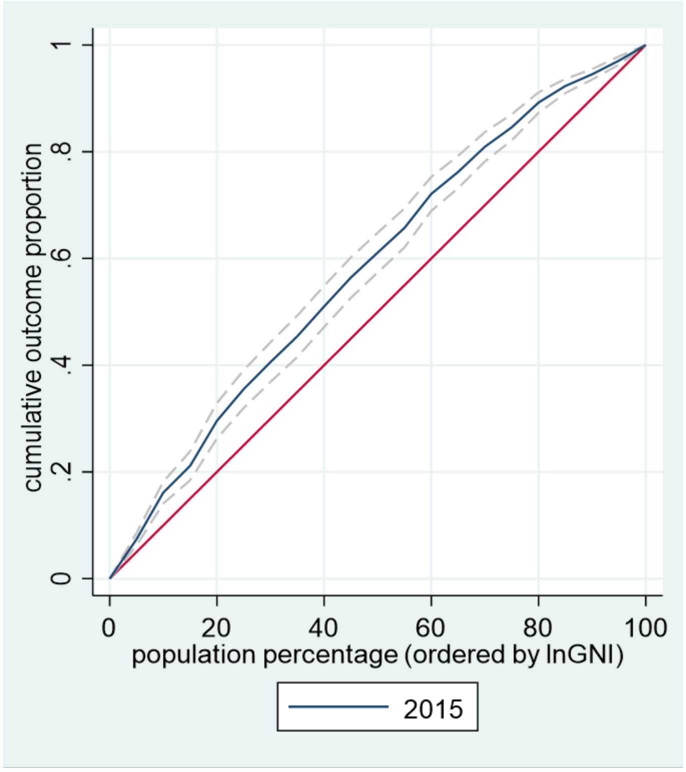
cumulative outcome proportion
0
.2
.4
.6
.8
1
0
20
40
60
80
100
population percentage (ordered by lnGNI)
2015

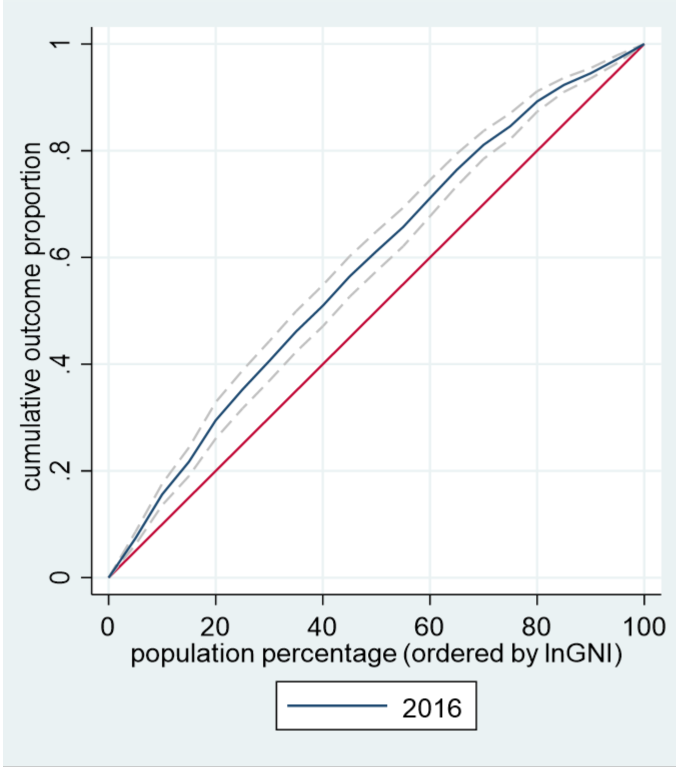
cumulative outcome proportion
0
.2
.4
.6
.8
1
0
20
40
60
80
100
population percentage (ordered by lnGNI)
2016

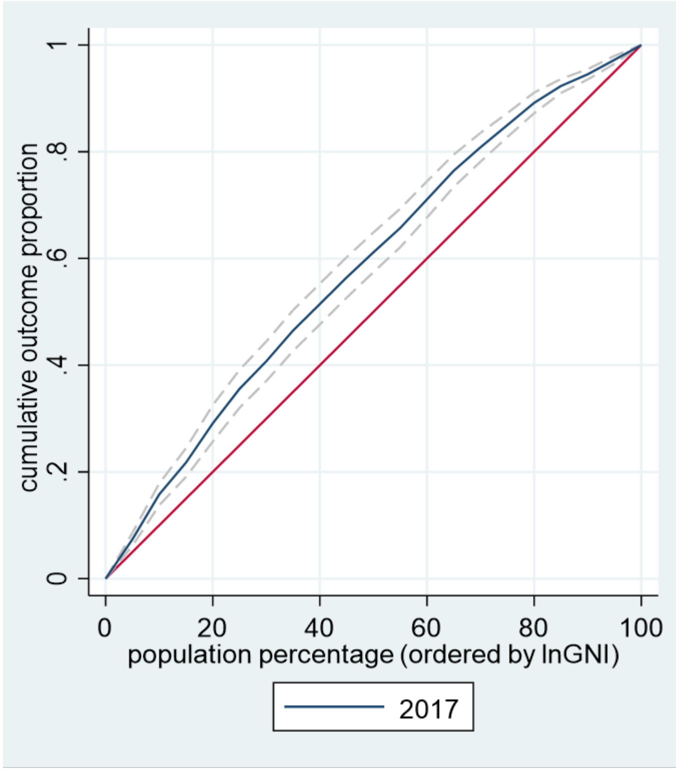

cumulative outcome proportion
0
.2
.4
.6
.8
1
0
20
40
60
80
100
population percentage (ordered by lnGNI)
2017

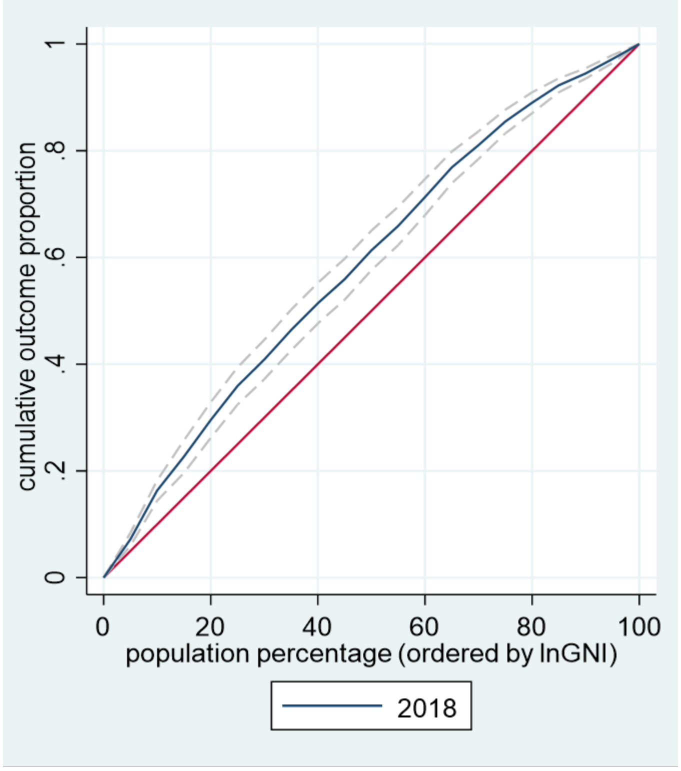

cumulative outcome proportion
0
.2
.4
.6
.8
1
0
20
40
60
80
100
population percentage (ordered by lnGNI)
2018

Table S1 Trends in concentration index of age-standardized DALY rates for rheumatic heart disease from 2000-2019

Segment	Period	APC/AAPC (%)	95% CI	*p*-value
Trend 1	2000–2012	–1.0*	–1.2 to –0.8	< 0.001
Trend 2	2012–2019	–0.5*	–1.0 to –0.0	0.036
Total	2000–2019	–0.4*	–0.6 to –0.2	< 0.001

APC=annual percentage change. AAPC=average annual percentage change. CI=confidence interval. *Significant at alpha=0.05.

Table S2 Trends in concentration index of age-standardized DALY rates for ischemic heart disease from 2000-2019

Segment	Period	APC/AAPC (%)	95% CI	*p*-value
Trend 1	2000–2002	–14.4*	–19.5 to –9.6	< 0.001
Trend 2	2002–2011	–9.4*	–9.9 to –8.9	< 0.001
Trend 3	2011–2015	–3.4*	–5.7 to –1.2	0.007
Trend 4	2015–2019	–0.1	–1.4 to 1.3	0.926
Total	2000–2019	–6.6*	–7.3 to –5.9	< 0.001

APC=annual percentage change. AAPC=average annual percentage change. CI=confidence interval. *Significant at alpha=0.05.

Table S3 Trends in concentration index of age-standardized DALY rates for stroke from 2000-2019

Segment	Period	APC/AAPC (%)	95% CI	*p*-value
Trend 1	2000–2011	–2.6*	–2.7 to –2.5	< 0.001
Trend 2	2011–2014	–1.0	–2.3 to 0.3	0.117
Trend 3	2014–2019	0.2	–0.1 to 0.5	0.179
Total	2000–2019	–1.6*	–1.8 to –1.4	< 0.001

APC=annual percentage change. AAPC=average annual percentage change. CI=confidence interval. *Significant at alpha=0.05.

Table S4 Trends in concentration index of age-standardized DALY rates for hypertensive heart disease from 2000-2019

Segment	Period	APC/AAPC (%)	95% CI	*p*-value
Trend 1	2000–2002	0.9	–0.3 to 2.1	0.113
Trend 2	2002–2006	0.1	–0.5 to 0.7	0.696
Trend 3	2006–2016	–0.4*	–0.5 to –0.3	< 0.001
Trend 4	2016–2019	–1.0*	–1.6 to –0.4	0.004
Total	2000–2019	–0.2*	–0.4 to –0.1	< 0.001

APC=annual percentage change. AAPC=average annual percentage change. CI=confidence interval. *Significant at alpha=0.05.

Table S5 Trends in concentration index of age-standardized DALY rates for non-rheumatic valvular heart disease from 2000-2019

Segment	Period	APC/AAPC (%)	95% CI	*p*-value
Trend 1	2000–2010	–0.1	–0.3 to 0.0	0.125
Trend 2	2010–2019	–1.2*	–1.4 to –1.0	< 0.001
Total	2000–2019	–0.6*	–0.7 to –0.5	< 0.001

APC=annual percentage change. AAPC=average annual percentage change. CI=confidence interval. *Significant at alpha=0.05.

Table S6 Trends in concentration index of age-standardized DALY rates for cardiomyopathy and myocarditis from 2000-2019

Segment	Period	APC/AAPC (%)	95% CI	*p*-value
Trend 1	2000–2007	12.7*	9.6 to 16.0	< 0.001
Trend 2	2007–2010	–39.1*	–45.2 to –32.2	< 0.001
Total 1	2000–2010	–6.3*	–9.0 to –3.4	< 0.001
Trend 3	2011–2015	–59.0*	–78.3 to –41.9	< 0.001
Trend 4	2015–2019	–2.5	–14.9 to 8.6	0.586
Total 2	2011–2019	–27.7*	–35.1 to –20.6	< 0.001

APC=annual percentage change. AAPC=average annual percentage change. CI=confidence interval. *Significant at alpha=0.05.

Table S7 Trends in concentration index of age-standardized DALY rates for atrial fibrillation and flutter from 2000-2019

Segment	Period	APC/AAPC (%)	95% CI	*p*-value
Trend 1	2000–2003	0.4	–0.8 to 1.6	0.462
Trend 2	2003–2008	–1.1*	–1.9 to –0.4	0.008
Trend 3	2008–2011	–2.5*	–4.8 to –0.1	0.040
Trend 4	2011–2019	–1.2*	–1.5 to –1.0	< 0.001
Total	2000–2019	–1.1*	–1.6 to –0.7	< 0.001

APC=annual percentage change. AAPC=average annual percentage change. CI=confidence interval. *Significant at alpha=0.05.

Table S8 Trends in concentration index of age-standardized DALY rates for aortic aneurysm from 2000-2019

Segment	Period	APC/AAPC (%)	95% CI	*p*-value
Trend 1	2000–2002	1.2	–1.8 to 4.2	0.401
Trend 2	2002–2006	–2.7*	–4.1 to –1.2	0.002
Trend 3	2006–2015	–5.1*	–5.4 to –4.8	< 0.001
Trend 4	2015–2019	2.1*	1.2 to 3.1	0.001
Total	2000–2019	–2.5*	–2.9 to –2.0	< 0.001

APC=annual percentage change. AAPC=average annual percentage change. CI=confidence interval. *Significant at alpha=0.05.

Table S9 Trends in concentration index of age-standardized DALY rates for peripheral artery disease from 2000-2019

Segment	Period	APC/AAPC (%)	95% CI	*p*-value
Trend 1	2000–2002	–0.0	–1.6 to 1.6	0.966
Trend 2	2002–2007	–1.1*	–1.6 to –0.6	0.001
Trend 3	2007–2010	–2.5*	–4.1 to –1.0	0.005
Trend 4	2010–2019	–1.8*	–1.9 to –1.6	< 0.001
Total	2000–2019	–1.5*	–1.8 to –1.3	< 0.001

APC=annual percentage change. AAPC=average annual percentage change. CI=confidence interval. *Significant at alpha=0.05.

Table S10 Trends in concentration index of age-standardized DALY rates for endocarditis from 2000-2019

Segment	Period	APC/AAPC (%)	95% CI	*p*-value
Trend 1	2000–2004	5.6*	4.1 to 7.0	< 0.001
Trend 2	2004–2019	–0.7*	–0.9 to –0.5	< 0.001
Total	2000–2019	0.7*	0.3 to 1.0	< 0.001

APC=annual percentage change. AAPC=average annual percentage change. CI=confidence interval. *Significant at alpha=0.05.

Table S11 Trends in concentration index of age-standardized DALY rates for other cardiovascular and circulatory diseases from 2000-2019

Segment	Period	APC/AAPC (%)	95% CI	*p*-value
Trend 1	2000–2013	–1.8*	–1.9 to –1.6	< 0.001
Trend 2	2013–2019	–0.6*	–1.1 to –0.0	0.040
Total	2000–2019	–1.4*	–1.6 to –1.2	< 0.001

APC=annual percentage change. AAPC=average annual percentage change. CI=confidence interval. *Significant at alpha=0.05.
